# Synthesis of Bicyclo[1.1.1]Pentane Z‐Substituted Enamides, Enol Ethers, and Vinyl Sulfides Using Iodine (III) Reagents

**DOI:** 10.1002/anie.202508404

**Published:** 2025-06-08

**Authors:** Najung Lee, Jonas Dechent, Elija Grinhagena, Jerome Waser

**Affiliations:** ^1^ Laboratory of Catalysis and Organic Synthesis Institute of Chemical Sciences and Engineering, École Polytechnique Fédérale de Lausanne Lausanne CH‐1015 Switzerland

**Keywords:** Alkenes, Bicyclo[1.1.1]pentanes, Bioisosteres, Hypervalent iodine reagents

## Abstract

Bicyclo[1.1.1]pentanes (BCPs) are well‐studied bioisosteres for *para*‐substituted benzene rings, and numerous methods for synthesizing 1,3‐difunctionalized BCPs have been developed. However, synthetic approaches to access vinyl BCP motifs remain limited, with only few reports on the synthesis of BCP‐olefins bearing heteroatom substituents. Herein, we present the synthesis of BCP‐substituted enamides, enol ethers, and vinyl sulfides through the sequential functionalization of ethynylbenziodoxolone (EBX) reagents containing a BCP scaffold. Stereoselective addition of a nucleophile generates heteroatomic Z‐vinylbenziodoxolone (VBX) reagents. The hypervalent iodine substituent of the VBX reagents serves then as a versatile platform for further arylation, vinylation, and trifluoromethylation. This method provides a modular synthesis of previously inaccessible heteroatom‐substituted vinyl BCPs.

## Graphical abstract

The sequential functionalization of novel BCP‐substituted ethynylbenziodoxolone (EBX) reagents into heteroatom‐ and BCP‐substituted alkenes is described. The addition of N, O, and S nucleophiles on the EBXs was stereoselective and generated heteroatomic Z‐vinylbenziodoxolone (VBX) reagents. The hypervalent iodine bond reacted under mild conditions to give access to *Z*‐substituted enamides, enol ethers, and vinyl sulfides.

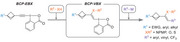

The search for novel bioisosteres with improved pharmacological properties, including binding affinity, water solubility, and reduced toxicity, has been the focus of intense research in the last decades. In particular, bicyclo[1.1.1]pentanes (BCPs) have attracted considerable attention as replacement for *para*‐substituted benzene rings due to their linear bond angle between substituents and their rigid structure.^[^ ^1^, ^2^, ^3^, ^4^, ^5^ ^]^ They often exhibit enhanced pharmacokinetic properties, such as increased solubility in water, improved membrane permeability, and reduced susceptibility to in vivo oxidation. Pharmacologically active drug molecules incorporating the BCP motif have been developed (Scheme 1a). Many of these new compounds such as γ‐secretase inhibitor **1** ^[^ ^1^ ^]^ and mutant IDH enzyme inhibitor **2** ^[^ ^6^ ^]^ bear a heteroatom in α‐position to the BCP, highlighting the importance of combining the rigid bioisostere with heteroatoms to interact with biological targets. Furthermore, vinyl‐BCP analogue **3** ^[^ ^7^ ^]^ of resveratrol exhibited improved in vivo pharmacokinetic properties compared to its parent compound, highlighting the positive effect of the bioisosteric replacement with the BCP scaffold in case of styrene derivatives.

**Scheme 1:**
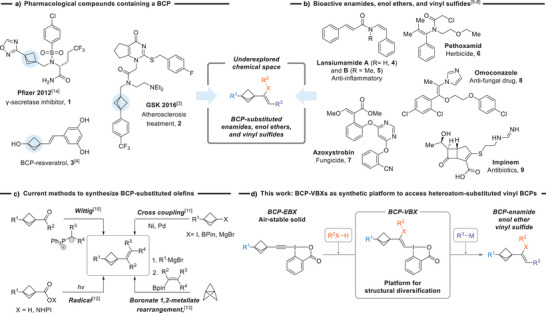
a) Examples of pharmaceutical molecules containing BCPs. b) Bioactive enamides, enol ethers, and vinyl sulfides. c) Current methods to synthesize vinyl BCPs. d) This work: Synthesis of Z‐substituted enamides, enol ethers, and vinyl sulfides from BCP‐EBXs.

Olefins bearing heteroatomic substituents, particularly enamides and enol ethers, are another class of valuable scaffolds used in the pharmaceutical and agrochemical industry (Scheme 1b). These compounds combine a heteroatom with a rigid alkene core, often bearing an aromatic ring as substituent, for example, bioactive compounds **4**–**8**.^[^ ^8^, ^9^, ^10^, ^11^, ^12^, ^13^ ^]^ Furthermore, they also serve as highly useful electron‐rich intermediates in organic synthesis.^[^ ^14^, ^15^, ^16^, ^17^ ^]^ Vinyl sulfides, sulfoxides, and sulfones have also emerged as attractive scaffolds in drug design, for example, the antibiotic Impinem **9**.^[^ ^18^, ^19^, ^20^, ^21^ ^]^ Despite recent advances in synthesizing 1,3‐disubstituted BCP derivatives,^[^ ^22^, ^23^, ^24^, ^25^ ^]^ general methods for preparing BCP‐substituted olefins remain scarce (Scheme 1c). Classical methods to access them include Wittig reactions,^[^ ^26^, ^27^, ^28^ ^]^ Suzuki‐ or Heck‐cross couplings,^[^ ^5^, ^29^, ^30^, ^31^ ^]^ and radical‐based processes.^[^ ^32^, ^33^ ^]^ In 2020, Aggarwal and co‐workers developed a method to synthesize vinyl‐BCPs via the 1,2‐metallate rearrangement of boronate complexes.^[^ ^34^ ^]^ While they successfully accessed 1,3‐difunctionalized BCP‐substituted olefins, the chemical space was largely limited to carbon‐based substituents with no example of heteroatoms on the double bond. In fact, there is only one reported example of a Wittig reaction to access a methoxy‐substituted vinyl‐BCP derivative.^[^ ^27^ ^]^ Therefore, there is an unmet need for general synthetic methods giving access to heteroatom‐substituted vinyl BCPs.

Since their introduction by Olofsson in 2016, VBX (vinylbenziodoxolone) reagents have led to the development of new transformations due to their unique reactivity as electrophilic alkene synthons.^[^ ^35^, ^36^ ^]^ Our group has previously reported the stereospecific synthesis of Z‐enamides and enol ethers utilizing VBX (vinylbenziodoxolone) reagents.^[^ ^37^ ^]^ The VBX reagents were obtained by stereoselective addition of sulfonamides and phenols on the corresponding EBX (ethynylbenziodoxolone) reagents. Herein, we report a stereoselective synthesis of BCP‐containing enamides, enol ethers, and vinyl sulfides using the corresponding newly developed EBX reagents bearing a BCP motif (Scheme 1d). These BCP‐EBX reagents are accessible in four to five steps from [1.1.1]propellane or a commercially available BCP derivative. Multiple functional groups were tolerated under the mild reaction conditions, enabling the modification of drug molecules and natural products. The benziodoxolone (BX) motif of the VBX reagents served as a versatile handle for introducing aryl, vinyl, and trifluoromethyl substituents.

First, four representative BCP‐EBXs bearing substituents with varying electronic properties (sulfone, methyl ester, phenyl, and benzyl) were synthesized (Scheme 2). The reagents were obtained from the silyl alkynes synthesized using different approaches. For the synthesis of sulfone‐substituted BCP‐EBX **EBX.1**, [1.1.1]propellane **10** was transformed into alkyne **12** by reaction with alkynyl sulfone **11** under energy‐transfer catalysis (Scheme 2a).^[^ ^38^ ^]^ Subsequent deprotection of the TIPS with TBAF and re‐protection with TMSCl gave TMS alkyne **14** in 78% yield over two steps. Unfortunately, it was not possible to directly achieve the light‐mediated sulfonyl alkynylation with a TMS protected alkynyl sulfone as we only observed degradation. Finally, the reaction between the TMS‐protected alkyne and HO‐BX **15** in the presence of TMSOTf resulted in BCP‐EBX **EBX.1** in 72% yield [31% overall yield from [1.1.1]propellane (**10**)]. The synthesis of methyl ester substituted **EBX.2** was achieved by a sequence of simple functional group transformations starting from commercially available BCP monoester **16** without the need for purification of the intermediates (Scheme 2b).^[^ ^39^ ^]^ For the coupling with HO‐BX **15**, more caution with the reaction time was required as decomposition was observed when the reaction proceeded for a longer period of time. The desired **EBX.2** was obtained in 78% yield (31% overall yield from commercial **16**). For phenyl and benzyl bearing BCP‐EBXs, we utilized the established strategy employing the addition of Grignard reagents to [1.1.1]propellane (**10**) to access BCP‐aldehydes **22** and **23**.^[^ ^40^ ^]^ The obtained aldehydes were then transformed into the corresponding alkynes via an Ohira‐Bestmann homologation with diazo compound **19**. The lower yields in this sequence are mostly due to the volatility of the intermediates. Unfortunately, the reaction between HO‐BX **15** and the TMS alkynes was not successful with Ph and Bn‐substituted BCP alkynes. We were still able to synthesize the desired BCP‐EBX **EBX.3** and **EBX.4** via a one‐pot procedure starting from 2‐iodobenzoic acid **28**,^[^ ^41^, ^42^ ^]^ albeit in lower yield compared to the other EBX derivatives (34% and 18%). We did not yet attempt to optimize the synthesis of these reagents, as the primary focus was the study of their reactivity.

**Scheme 2:**
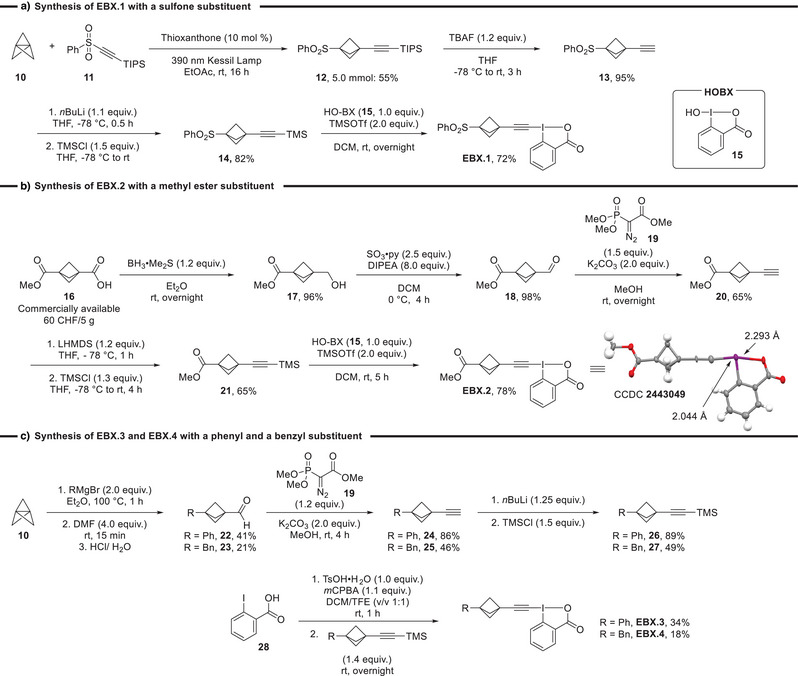
Synthesis of BCP‐EBX reagents **EBX.1–4**.

The crystal structure of methyl ester substituted **EBX.2** showed a characteristic near‐linear C─I─O bond around the iodine (III) atom with a bond angle of 169.16°.^[^ ^43^ ^]^ The C─I and I─O bond lengths were 2.044 and 2.293 Å, respectively, which are similar to other EBXs.^[^ ^44^ ^]^ Considering that BCP‐EBX reagents combine the energy of the strained BCP with the weak hypervalent bond, it was important to investigate their stability. DSC measurement of **EBX.2** revealed decomposition starting at 56 °C, but it took place progressively up to a temperature of 169 °C with a peak height of 0.37 W g^−1^ and an energy of 300 J g^−1^ (See Figure S3 in Supporting Information). A second more energy‐intensive decomposition was then observed in the range from 193°C to 324 °C (622 J g^−1^). These results indicate that the reagents should still be handled with care, even if the risk is lower than for other benziodoxolone reagents. No significant decomposition was observed upon storage in the fridge for 2 months for all EBX reagents.

With the newly synthesized BCP‐EBX reagents in hand, we then investigated the nucleophilic addition of sulfonamides, phenols, and thiols. We based the reaction conditions on the previous work of our group on other EBX reagents.^[^ ^37^ ^]^ Changing the solvent from ethanol to DCM was necessary due to the limited solubility of BCP‐EBXs in ethanol. Gratifyingly, the reaction with BCP‐EBXs proceeded smoothly with a catalytic amount of cesium carbonate in flasks open to air in DCM. First, we investigated the reaction of BCP‐EBXs with sulfonamides. With sulfone‐substituted **EBX.1**, N‐VBX **29a** bearing a tosyl sulfonamide was obtained in 86% yield, and the yield was conserved at a 1 mmol scale. The X‐ray crystal structure of **29a** confirmed the *Z* geometry of the double bond, which was consistent with our previous studies.^[^ ^37^ ^]^ Both a mesyl and a nosyl sulfonamides were tolerated to give VBXs **29b** and **29c** in 86% and 73% yield, respectively. A slight decrease in the yield was observed with an ester group on the BCP, giving **29d** in 71% yield. The addition of mesyl and nosyl sulfonamides gave **29e** and **29f** in 57% and 55% yield. EBX reagents bearing more electron donating phenyl and benzyl substituents gave the corresponding N‐VBX **29** **g** and **29** **h** in 63% and 61% yield, respectively. Overall, we observed decreased yields when the reaction was performed with EBXs with less electron‐withdrawing substituents. The same protocol could be applied to phenols as nucleophiles, including tyrosine, resulting in the formation of vinyl ethers **30a**‐**i** in 60%–85% yield (Scheme 3b). Compared to our previous work with other EBX reagents, the obtained yields of N‐VBX reagents were comparable (43%–94%).^[^ ^37^ ^]^ In comparison with the work of Itoh for the synthesis of N‐VBX reagents, the yields were within a similar range (42%–99%).^[^ ^45^ ^]^ For O‐VBX reagents, the obtained yields were generally comparable or even higher for most of the combinations tested in comparison to our previous report (23%–67%, one entry with 91% yield for O‐VBX)^[^ ^37^ ^]^ and to other reported literature for the synthesis of O‐VBXs.^[^ ^46^, ^47^, ^48^ ^]^ Therefore, our work demonstrates that the addition of nucleophiles proceeds with equal efficiency with a BCP substituent when compared to standard EBX reagents.

**Scheme 3:**
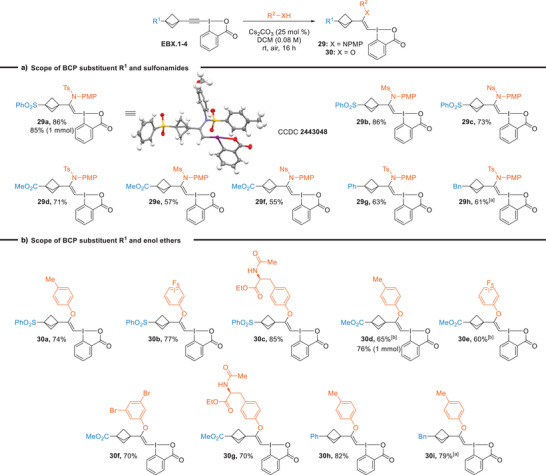
Scope of a) N‐ and b) O‐ VBXs. Reactions performed at a 0.3 mmol scale. ^[a]^ Reaction performed at a 0.15 mmol scale. ^[b]^ Reaction performed in a 0.008 M solution.

We then investigated the potential of modifying more complex drug molecules or natural products (Scheme 4). The functionalization of the sulfa drug sulfaphenazole was successful without any reaction on the free aromatic amine (**31**). Bioactive compounds bearing a phenol such as α‐tocopherol and estradiol were also tolerated (**32** and **33**). We exclusively observed the addition of phenol in presence of an aliphatic alcohol in the case of estradiol. This selectivity could originate from the deprotonation of the most acidic phenol proton. In the context of drug functionalization, we were also interested in extending the scope of nitrogen nucleophiles beyond sulfonamides. Gratifyingly, it was possible to modify the tetrazole heterocycle in the drug valsartan to give the corresponding VBX **34** in 46% yield. Although tetrazoles and carboxylic acids are considered very close in acidity and nucleophilicity, we did not observe any reaction on the latter.

**Scheme 4:**
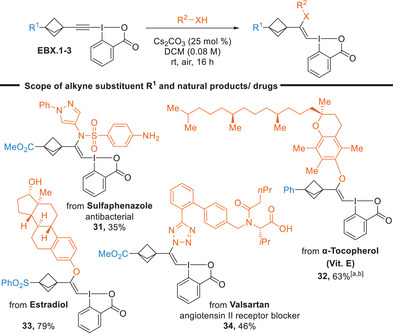
Scope of natural products and drugs. Reactions performed at a 0.3 mmol scale. ^a)^ Reaction performed at a 0.15 mmol scale. ^b)^ **32** was obtained as a 1:1 mixture of isomers, tentatively assigned as *E* and *Z* double bond isomers by NOE experiments, see Supporting Information for details.

Having successfully added harder O and N nucleophiles, we wondered if softer thiol nucleophiles could also be used to give S‐VBXs (Scheme 5). It was necessary to further optimize the reaction conditions in this case due to a competing side reaction resulting from the addition of a second equivalent of thiol to give vinyl disulfide **36** (See Tables S1 and S2). With the modified conditions (lower base loading, different solvent mixture and dilution), it was possible to achieve good yields also for this transformation. The addition of benzyl mercaptan to sulfone‐substituted **EBX.1** gave **35a** in 74% yield. The addition of 2‐bromothiol to **EBX.2** resulted in the corresponding VBX **35b** with a slightly lower yield of 53%. The addition of protected cysteine was also successful, albeit in lower yield (**35c**, 42%). The addition of thiophenol to phenyl‐BCP **EBX.3** and benzyl‐BCP **EBX.4** resulted in 74% yield of **35d** and 70% yield of **35e**.

**Scheme 5:**
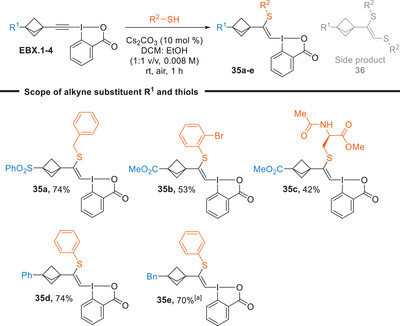
Scope of S‐VBXs. Reactions performed at a 0.3 mmol scale. ^a)^ Reaction performed at a 0.15 mmol scale.

With a wide range of functionalized BCP‐VBX reagents in hand, we then explored their transformation into substituted enamides, enol ethers, and vinyl sulfides by exploiting the high reactivity of the hypervalent iodine bond (Scheme 6). First, palladium‐catalyzed Stille and Suzuki couplings were investigated. The coupling of aryl and vinyl stannyl reagents was achieved at room temperature with O‐VBX reagents **30a** and **30d** to give **37a** and **37b** in 79% and 87% yield, respectively. With N‐VBX reagents, an increased catalyst loading and a higher temperature were needed for complete conversion, using conditions reported by Yoshikai and co‐workers.^[^ ^46^ ^]^ The corresponding enamides with vinyl or phenyl substituents were obtained in 57%–80% yield (**37c**‐**g**). Furthermore, it was possible to achieve selective Stille coupling with the BX motif in presence of an aryl bromide, giving vinyl sulfide **37** **h** in 78% yield. In all Stille coupling reactions, complete retention of the double bond geometry was observed with only *Z*‐ enamides and enol ethers as products. Suzuki coupling with the S‐VBX reagent **35a** gave product **37i** in 41% yield with a 4:1 *Z*:*E* ratio, showing partial isomerization in this case. Finally, we attempted trifluoromethylation with a copper (III) reagent, which had been used by our group with other VBXs.^[^ ^49^ ^]^ Despite the thermal sensitivity of the BCP‐VBX reagents, the transformation proceeded in 76%–89% yield at 120 °C to give products **38a**‐**c**. A slightly lower yield of 58% of **38d** was observed for the trifluoromethylation of S‐VBX **35c** bearing a cysteine substituent. BCP‐containing enamides, enol ethers, and vinyl sulfides could be further modified by reaction of the double bond (Scheme 7). Hydrogenation of the sterically hindered trisubstituted olefin α to a quaternary center was successful with simple palladium‐based heterogenous catalysts at an atmospheric pressure of hydrogen, giving **39** and **40** in 71% and 76% yield with the BCP core untouched. This allows to access α‐amino BCPs, which have been already used in the pharmaceutical industry (Scheme 1a), and α‐ether BCPs. Furthermore, the double bond could be transformed into epoxide **41** using *m*CBPA in quantitative yield. The epoxide then could be further treated with acetic acid to afford α‐acetoxy ketone **42** in 65% yield.^[^ ^50^ ^]^ The oxidation of vinyl sulfide **37** **h** with *m*CPBA resulted in the corresponding vinyl sulfone **43** in 93% yield.

**Scheme 6:**
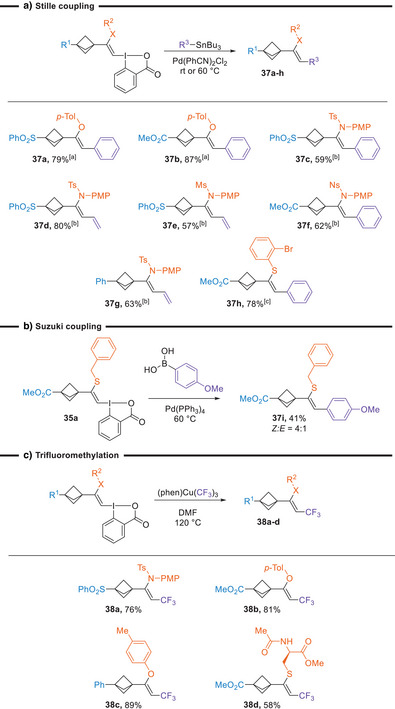
Functionalization of the VBX products. Reaction conditions: a). ^[a]^ 0.10 mmol VBX reagent, 0.20 mmol stannane, 5 mol% Pd(PhCN)_2_Cl_2_, 0.1 M in DMF, 16 h, rt. ^[b]^ 0.10 mmol VBX reagent, 0.20 mmol stannane, 10 mol% Pd(PhCN)_2_Cl_2_, 0.1 M in DMF, 16 h, 60 °C. ^[c]^ 0.10 mmol VBX reagent, 0.11 mmol stannane, 5 mol% Pd(PhCN)_2_Cl_2_, 0.1 M in DMF, 16 h, rt. **b)** 0.10 mmol VBX reagent, 0.2 mmol boronic acid, 0.3 mmol Cs_2_CO_3_, 10 mol % Pd(PPh_3_)_4_, 0.1 M in DMF:H_2_O (8:2 v/v), 16 h, 60 °C. c). Reaction conditions: 0.10 mmol VBX reagent, 0.15 mmol (phen)Cu(CF_3_)_3_, 0.08 M in DMF, 120 °C, 1 h.

**Scheme 7:**
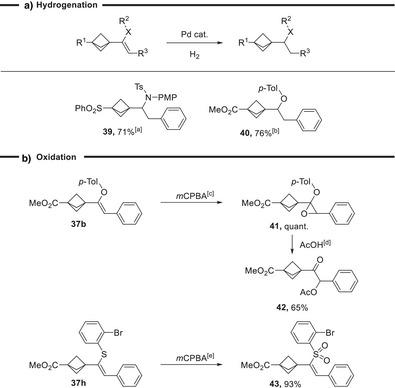
Functionalization of the double bond. a). ^[a]^ 0.10 mmol of enamide **37c**, 20 mol % Pd(OH)_2_, 1 atm H_2_, 0.1 M in MeOH, 45 °C, 16 h. ^b)^ 0.10 mmol of enol ether **37b**, 20 mol % Pd/C, 1 atm H_2_, 0.1 M in MeOH, 45 °C, 16 h. b). ^[c]^ 0.10 mmol of enol ether **37b**, 0.12 mmol of *m*CBPA, DCM/ NaHCO_3_, rt, 1 h. ^[d]^ 0.10 mmol of epoxide **41**, 0.5 mL of acetic acid, rt, 30 min. ^[e]^ 0.10 mmol of vinyl sulfide **37** **h**, 0.40 mmol of *m*CBPA, DCM, rt, 5 h.

In summary, we have reported the first synthesis of EBX reagents bearing a BCP substituent (BCP‐EBX **EBX.1–4**). Their transformation into the corresponding VBX reagents was successful in a stereoselective manner with a catalytic amount of base under mild reaction conditions for four representative classes of substituent on the BCP (sulfone, ester, phenyl, and benzyl). Nitrogen‐, oxygen‐, and sulfur‐substituted VBX reagents were obtained in high yield and were stable to column chromatography. Thanks to the high reactivity of the hypervalent iodine bond, the VBX reagents gave access to aryl, vinyl, and trifluoromethyl‐*Z*‐substituted enamides, enol ethers, and vinyl sulfides with high stereoselectivity, expanding the chemical space of BCP‐based libraries.

## Supporting Information

Supporting information is available as pdf file, including general methods, experimental procedures, compound characterization data, and copy of NMR spectra of new compounds. Raw data for compound characterization will be available free access at zenodo.org at https://doi.org/10.5281/zenodo.15525527 after final publication of the work. The authors have cited additional references within the Supporting Information.^[^ ^38^, ^51^, ^52^, ^53^, ^54^, ^55^ ^]^

## Conflict of Interests

The authors declare no conflict of interest.

## Supporting information



Supporting Infromation

## Data Availability

The data that support the findings of this study are available in the Supporting Information of this article.

## References

[1] *For selected examples on the use of BCP in medicinal chemistry*, see: A. F. Stepan , C. Subramanyam , I. V. Efremov , J. K. Dutra , T. J. O'Sullivan , K. J. DiRico , W. S. McDonald , A. Won , P. H. Dorff , C. E. Nolan , S. L. Becker , L. R. Pustilnik , D. R. Riddell , G. W. Kauffman , B. L. Kormos , L. Zhang , Y. Lu , S. H. Capetta , M. E. Green , K. Karki , E. Sibley , K. P. Atchison , A. J. Hallgren , C. E. Oborski , A. E. Robshaw , B. Sneed , C. J. O'Donnell , J. Med. Chem. 2012, 55, 3414–3424. 22420884 10.1021/jm300094u

[2] H. Ratni , K. Baumann , P. Bellotti , X. A. Cook , L. G. Green , T. Luebbers , M. Reutlinger , A. F. Stepan , W. Vifian , RSC Med. Chem. 2021, 12, 758–766. 34124674 10.1039/d1md00043h PMC8152580

[3] *For selected reviews of the use of BCP in medicinal chemistry*, see: Y. P. Auberson , C. Brocklehurst , M. Furegati , T. C. Fessard , G. Koch , A. Decker , L. La Vecchia , E. Briard , ChemMedChem 2017, 12, 590–598. 28319646 10.1002/cmdc.201700082

[4] P. K. Mykhailiuk , Org. Biomol. Chem. 2019, 17, 2839–2849. 30672560 10.1039/c8ob02812e

[5] Z. Fang , Q. Xu , X. Lu , N. Wan , W.‐L. Yang , Synthesis 2025, 57, 1171–1179.

[6] N. D. Measom , K. D. Down , D. J. Hirst , C. Jamieson , E. S. Manas , V. K. Patel , D. O. Somers , ACS Med. Chem. Lett. 2017, 8, 43–48. 28105273 10.1021/acsmedchemlett.6b00281 PMC5238484

[7] Y. L. Goh , Y. T. Cui , V. Pendharkar , V. A. Adsool , ACS Med. Chem. Lett. 2017, 8, 516–520. 28523103 10.1021/acsmedchemlett.7b00018 PMC5430408

[8] L. Song , G. Li , W. Guan , Z. Zeng , Y. Ou , T. Zhao , J. Li , D. He , X. Fang , Y. Zhang , J. Wu , R. Tong , H. Yao , Biomed. Pharmacother. 2023, 166, 115412. 37660652 10.1016/j.biopha.2023.115412

[9] H. Xu , T. Chen , L. Huang , Q. Shen , Z. Lian , Y. Shi , M.‐A. Ouyang , L. Song , Molecules 2018, 23, 1499. 29933580 10.3390/molecules23071499 PMC6099640

[10] S. A. Z. Ahmad , T. K. Jena , F. A. Khan , Chem. Asian J. 2021, 16, 1685–1702. 33979009 10.1002/asia.202100277

[11] R. Nofiani , K. de Mattos‐Shipley , K. E. Lebe , L.‐C. Han , Z. Iqbal , A. M. Bailey , C. L. Willis , T. J. Simpson , R. J. Cox , Nat. Commun. 2018, 9, 3940. 30258052 10.1038/s41467-018-06202-4 PMC6158276

[12] T. Itoyama , K. Uchida , H. Yamaguchi , S. Fujita , J. Antimicrob. Chemother. 1997, 40, 441–444. 9338501 10.1093/jac/40.3.441

[13] T. B. Poulsen , Acc. Chem. Res. 2021, 54, 1830–1842. 33660974 10.1021/acs.accounts.0c00851

[14] G. Stork , A. Brizzolara , H. Landesman , J. Szmuszkovicz , R. Terrell , J. Am. Chem. Soc. 1963, 85, 207–222.

[15] R. Matsubara , S. Kobayashi , Acc. Chem. Res. 2008, 41, 292–301. 18281949 10.1021/ar700098d

[16] K. Gopalaiah , H. B. Kagan , Chem. Rev. 2011, 111, 4599–4657. 21568332 10.1021/cr100031f

[17] T. Courant , G. Dagousset , G. Masson , Synthesis 2015, 47, 1799–1856.

[18] D. C. Meadows , J. Gervay‐Hague , Med. Res. Rev. 2006, 26, 793–814. 16788979 10.1002/med.20074

[19] R. Zhang , H. Ding , X. Pu , Z. Qian , Y. Xiao , Catalysts 2020, 10, 1339.

[20] R. Ahmadi , S. Emami , Eur. J. Med. Chem. 2022, 234, 114255. 35305462 10.1016/j.ejmech.2022.114255

[21] J. Tong , J. Shu , Y. Wang , Y. Qi , Y. Wang , Life. Sci. 2024, 352, 122904. 38986895 10.1016/j.lfs.2024.122904

[22] *For selected reviews* see: J. Kanazawa , M. Uchiyama , Synlett 2019, 30, 1–11.

[23] X. Ma , L. N. Pham , Asian J. Org. Chem. 2020, 9, 8–22.

[24] B. R. Shire , E. A. Anderson , JACS Au 2023, 3, 1539–1553. 37388694 10.1021/jacsau.3c00014 PMC10301682

[25] I. Sánchez‐Sordo , S. Barbeira‐Arán , M. Fañanás‐Mastral , Org. Chem. Front. 2024, 11, 916–928. 38298565 10.1039/d3qo01631e PMC10825854

[26] W. Huang , S. Keess , G. A. Molander , Chem. Sci. 2022, 13, 11936–11942. 36320918 10.1039/d2sc05100a PMC9580470

[27] R. Bychek , P. K. Mykhailiuk , Angew. Chem. Int. Ed. 2022, 61, e202205103. 10.1002/anie.202205103 PMC9401599 35638404

[28] H. Liu , Z. Fu , X. Li , S. Yu , Green Chem. 2024, 27, 256–263.

[29] M. Messner , S. I. Kozhushkov , A. de Meijere , Eur. J. Org. Chem. 2000, 2000, 1137–1155.

[30] M. Kondo , J. Kanazawa , T. Ichikawa , T. Shimokawa , Y. Nagashima , K. Miyamoto , M. Uchiyama , Angew. Chem. Int. Ed. 2020, 59, 1970–1974. 10.1002/anie.201909655 31603274

[31] V. Srinivasu , K. Pal , S. Giri , D. Sureshkumar , Org. Lett. 2024, 26, 10328–10333. 39582174 10.1021/acs.orglett.4c03913

[32] H. Cao , H. Jiang , H. Feng , J. M. C. Kwan , X. Liu , J. Wu , J. Am. Chem. Soc. 2018, 140, 16360–16367. 30412399 10.1021/jacs.8b11218

[33] J. Brals , T. M. McGuire , A. J. B. Watson , Angew. Chem. Int. Ed 2023, 62, e202310462. 10.1002/anie.202310462 PMC10952440 37622419

[34] S. Yu , C. Jing , A. Noble , V. K. Aggarwal , Angew. Chem. Int. Ed. 2020, 59, 3917–3921. 10.1002/anie.201914875 31912941

[35] E. Stridfeldt , A. Seemann , M. J. Bouma , C. Dey , A. Ertan , B. Olofsson , Chem. ‐ Eur. J. 2016, 22, 16066–16070. 27629653 10.1002/chem.201603955

[36] N. Declas , G. Pisella , J. Waser , Helv. Chim. Acta 2020, 103, e2000191.

[37] P. Caramenti , N. Declas , R. Tessier , M. D. Wodrich , J. Waser , Chem. Sci. 2019, 10, 3223–3230. 30996905 10.1039/c8sc05573d PMC6430016

[38] Z. Wu , Y. Xu , H. Zhang , X. Wu , C. Zhu , Chem. Commun. 2021, 57, 6066–6069. 10.1039/d1cc02249k 34037006

[39] S. O. Kokhan , Y. B. Valter , A. V. Tymtsunik , I. V. Komarov , O. O. Grygorenko , Eur. J. Org. Chem. 2017, 2017, 6450–6456.

[40] D. Lasányi , D. Máth , G. L. Tolnai , J. Org. Chem. 2022, 87, 2393–2401. 35050600 10.1021/acs.joc.1c02267

[41] M. J. Bouma , B. Olofsson , Chem. ‐ Eur. J. 2012, 18, 14242–14245. 23033155 10.1002/chem.201202977

[42] T. M. Milzarek , N. P. Ramirez , X.‐Y. Liu , J. Waser , Chem. Commun. 2023, 59, 12637–12640. 10.1039/d3cc04525k 37791867

[43] Deposition numbers2443049 (for EBX.2) and 2443048 (for 29a) contain the supplementary crystallographic data for this paper. These data are provided free of chargefrom the Cambridge Crystallographic Data Centre .

[44] E. L.e Du , N. P. Ramirez , S. Nicolai , R. Scopelliti , F. Fadaei‐Tirani , M. D. Wodrich , D. P. Hari , J. Waser , Helv. Chim. Acta 2023, 106, e202200175.

[45] D. Shimbo , A. Shibata , M. Yudasaka , T. Maruyama , N. Tada , B. Uno , A. Itoh , Org. Lett. 2019, 21, 9769–9773. 31742414 10.1021/acs.orglett.9b03990

[46] W. Ding , J. Chai , C. Wang , J. Wu , N. Yoshikai , J. Am. Chem. Soc. 2020, 142, 8619–8624. 32362119 10.1021/jacs.0c04140

[47] J. Chai , W. Ding , J. Wu , N. Yoshikai , Chem. Asian J. 2020, 15, 2166–2169. 32506821 10.1002/asia.202000653

[48] M. Li , W. Li , C.‐D. Lin , J.‐H. Wang , L.‐R. Wen , J. Org. Chem. 2019, 84, 6904–6915. 31084019 10.1021/acs.joc.9b00659

[49] T. M. Milzarek , J. Waser , Angew. Chem. Int. Ed. 2023, 62, e202306128. 10.1002/anie.202306128 37311164

[50] N. Declas , J. Waser , Angew. Chem. Int. Ed. 2020, 59, 18256–18260. 10.1002/anie.202006707 32542955

[51] P. Palamini , J. Borrel , M. Djaïd , M. Delattre , J. Waser , Org. Lett. 2023, 25, 7535–7539. 37801735 10.1021/acs.orglett.3c02869

[52] L. Gnägi , S. V. Martz , D. Meyer , R. M. Schärer , P. Renaud , Chem. ‐ Eur. J. 2019, 25, 11646–11649. 31359455 10.1002/chem.201903392

[53] U. Kloeckner , B. J. Nachtsheim , Chem. Commun. 2014, 50, 10485. 10.1039/c4cc04738a 25068377

[54] K. Liu , G. Wang , Z.‐W. Zhang , Y.‐Y. Shi , Z.‐S. Ye , Org. Lett. 2022, 24, 6489–6493. 36069728 10.1021/acs.orglett.2c02201

[55] K. Endo , H. Ube , M. Shionoya , J. Am. Chem. Soc. 2020, 142, 407–416. 31804816 10.1021/jacs.9b11099

