# Supplementary material for: Synthesis of Bicyclo[1.1.1]Pentane Z‐Substituted Enamides, Enol Ethers, and Vinyl Sulfides Using Iodine (III) Reagents
Source: Angew Chem Int Ed Engl. 2025 Jun 8;64(32):e202508404. doi: 10.1002/anie.202508404 (PMC12322621; doi:10.1002/anie.202508404)

Synthesis of Bicyclo[1.1.1]pentane Z-Substituted Enamides, Enol Ethers, and Vinyl Sulfides Using Iodine (III) Reagents

Najung Lee, Jonas Dechent, Elija Grinhagena and Jerome Waser*

Laboratory of Catalysis and Organic Synthesis, Institute of Chemical Sciences and Engineering, Ecole Polytechnique Fédérale de Lausanne, EPFL SB ISIC LCSO, BCH 4306, 1015 Lausanne, Switzerland.

jerome.waser@epfl.ch

Table of Contents

1. Materials and Methods 3
2. Preparation of starting materials 4
   2.1. Preparation of hypervalent iodine precursor 4
   2.2. Preparation of [1.1.1]propellane (10) 4
   2.3. Preparation of 11 5
   2.4. Preparation of EBX reagents 5
   2.5. Preparation of sulfonamides 13
3. Optimization of the synthesis of VBX reagents 15
4. Scope of N-VBX, N-VBX, and S-VBXs 17
   4.1. General Procedure GP2 for the synthesis of N-VBX and O-VBX 17
   4.2. General Procedure GP3 for the synthesis of S-VBX 29
5. Further functionalization employing VBXs 31
   5.1. C-C bond formation 31
   5.2. Hydrogenation 39
   5.3. Oxidation 40
6. Crystal structure 43
7. DSC experiment of EBX.2 47
8. Spectra of novel compounds 48

1. Materials and Methods

All reactions were carried out in oven dried glassware under an atmosphere of nitrogen, unless stated otherwise. Reactions requiring heating were carried out using DrySyn heating block. For quantitative flash chromatography, technical grade solvents were used. For flash chromatography for analysis, HPLC grade solvents from Sigma-Aldrich were used. THF, CH_3CN, Et_2O, CH_2Cl_2 and toluene were dried by passage over activated alumina under nitrogen atmosphere (H_2O content <10 ppm, Karl-Fischer titration). The solvents were degassed through Freeze-Pump-Thaw method when mentioned. All chemicals were purchased from Acros, Aldrich, Fluka, VWR, Aplichem, or Merck and used as such unless otherwise stated. Chromatographic purification was performed as flash chromatography using Macherey-Nagel silica 40-63, 60 Å, with the solvents indicated as eluent under 0.1-0.5 bar pressure. TLC was performed on Merck silica gel 60 F254 TLC glass plates or aluminium plates and visualized with UV light, permanganate stain, CAN stain, or *p*-anisaldehyde stain. Melting points were measured on a Büchi B-540 melting point apparatus using open glass capillaries, the data is uncorrected. ^{1}H-NMR spectra were recorded on a Brucker DPX-400 400 MHz spectrometer in $CDCl_3$, DMSO-d_6 CD_3OD, C_6D_6 and CD_2Cl_2, all signals are reported in ppm with the internal chloroform signal at 7.26 ppm, the internal DMSO signal at 2.50 ppm the internal methanol signal at 3.30 ppm, the internal dichloromethane signal at 5.30 ppm as standard. The data is being reported as (s = singlet, d = doublet, t = triplet, q = quadruplet, qi = quintet, m = multiplet or unresolved, br = broad signal, app = apparent, coupling constant(s) in Hz, integration, interpretation).^{13}C-NMR spectra were recorded with 1H-decoupling on a Brucker DPX-400 100 MHz spectrometer in $CDCl_3$, DMSO-d_6, CD_3OD or CD_2Cl_2, all signals are reported in ppm with the internal chloroform signal at 77.0 ppm, the internal DMSO signal at 39.5 ppm, the internal methanol signal at 49.0 ppm and the internal dichloromethane signal at 54.0 ppm as standard. ^{19}F-NMR spectra were recorded with {^{1}H} decoupling on a Bruker DPX-400 376 MHz spectrometer in chloroform-d. IR spectra were recorded on an Alpha-P Bruker FT-IR Spectrometer. Absorbance frequencies are reported in reciprocal centimeters (cm^{-1}) with indicated relative intensities: s (strong, 0-33% T); m (medium, 34-66% T); w (weak, 67-100% T). High resolution mass spectrometric measurements were performed by the mass spectrometry service of ISIC at the EPFL on a MICROMASS (ESI) Q-TOF Ultima API. For medium pressure liquid chromatography (MPLC) the BÜCHI Pure C-810 Flash system was used together with Reverleris® Reverse Phase (RP) C18 columns (Grace) using UV-detection at 220 nm, 254 nm, and 280 nm. The eluent system consisted of A = H_2O + 0.05% TFA, B = MeCN + 0.05% TFA. The purification method used the following elution gradient: 0–25 min 5% to 95% B, 25–30 min with a flow rate of 20 or 36 mL/min. Light mediated reactions were performed with the Schlenk flask irradiated by Kessil lamp at a distance of ~4 cm from the bottom of the reaction vessel. X-ray analyses of compounds **EBX.2** and **29a** were performed by Dr. F. Tirani and Dr. R. Scopelliti at the EPF Lausanne.

2. Preparation of starting materials

2.1. Preparation of hypervalent iodine precursor

1-Hydroxy-1,2-benziodoxol-3-(1H)-one (HO-BX, 15)

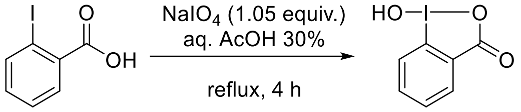

Following a reported literature,[1] $NaIO_4$ (9.0 g, 42 mmol, 1.0 equiv.) and 2-iodobenzoic acid (9.9 g, 40 mmol, 1.0 equiv.) were suspended in a mixture of AcOH (18 mL) and water (42 mL). The mixture was vigorously stirred and refluxed for 4 h. The reaction mixture was then diluted with cold water (50 mL) and allowed to cool to 21 °C protected from light. The crude product was collected by filtration, washed on the filter with ice water (3 x 25 mL) and acetone (3 x 25 mL), and air-dried in the dark to give the pure product as a white solid (10 g, 39 mmol, 98%).

^{1}H NMR (400 MHz, DMSO) δ 8.01 (dd, *J* = 7.5, 1.5 Hz, 1H, Ar*H*), 7.96 (ddd, *J* = 8.5, 7.1, 1.5 Hz, 1H, Ar*H*), 7.84 (dd, *J* = 8.2, 1.0 Hz, 1H, Ar*H*), 7.70 (td, *J* = 7.3, 1.0 Hz, 1H, Ar*H*).

^{13}C NMR (101 MHz, DMSO) δ 167.7, 134.5, 131.5, 131.1, 130.4, 126.3, 120.4.

All spectroscopic data in accordance with the literature.[1]

2.2. Preparation of [1.1.1]propellane (10)

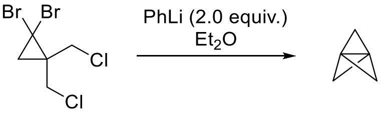

Following a reported literature procedure,[2] a 250 mL 2-neck flask charged with a stirring bar was flame-dried under reduced pressure and purged with nitrogen. To the flask was added 1,1-dibromo-2,2-bis(chloromethyl)cyclopropane (8.9 g, 30 mmol, 1.0 equiv.). The flask was then purged with nitrogen 3 times, and Et_2O (25 mL) was added. The flask was cooled to −45 °C (dry ice/ MeCN bath). Phenyllithium (32 mL, 2.0 M in Bu_2O, 60 mmol, 2.0 equiv.) was added dropwise over 30 min with a syringe pump. After complete addition, the orange-coloured solution was stirred for 5 min at −45 °C then warmed up to 0 °C and further stirred. An orange-coloured heterogenous mixture was formed. After 2 h, the reaction flask was warmed up to rt, and a distillation head was flushed with nitrogen. The distillation started at 200 mbar while the receiver flask was immersed in – 78 °C (dry ice/ acetone bath). The pressure was gradually lowered to 25 mbar, and the distillation was stopped after approx. 30 min. The flask was filled with nitrogen and stored at -20 °C before use.

Determination of the Yield of XX. CH_2Br_2 (10.0 µL, 0.144 mmol) was added as an internal standard to an aliquot (0.2 mL) of the solution, and the concentration was determined by the relative integration ratio between the internal standard (using the singlet at δ 4.9 (2H) ppm) and [1.1.1]propellane peak (using the singlet at δ 1.95 (6H) ppm). The typical concentration ranged from 0.60 to 0.77 M.

^{1}H NMR (400 MHz, $CDCl_3$) δ 1.95 (s, 6H).

^{13}C NMR (101 MHz, $CDCl_3$) δ 74.0, 0.9.

---

[1] P. Palamini, J. Borrel, M. Djaïd, M. Delattre, J. Waser*, Org. Lett*. **2023**, *25*, 7535–7539.

[2] D. Lasányi, D. Máth, G. L. Tolnai, *J. Org. Chem*. **2022**, *87*, 2393–2401.

Spectroscopic data in accordance with the literature.[2]

2.3. Preparation of 11

Triisopropyl((phenylsulfonyl)ethynyl)silane (11)

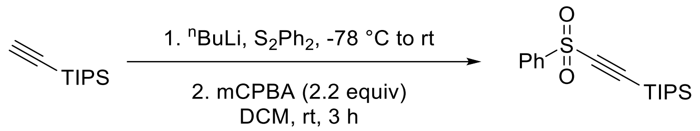

Following a reported literature procedure,[3] TIPS acetylene (3.7 g, 4.5 mL, 20 mmol) was dissolved in dry THF (20 mL) and the solution was cooled to -78 °C. *n*-BuLi (2.5 M in hexane, 8.0 mmol, 1.0 equiv.) was added dropwise at -78 °C and the mixture was stirred for 30 min at this temperature. Diphenyldisulfide (4.6 g, 21 mmol, 1.1 equiv.) in dry THF (10 mL) was slowly added at –78 °C. After being stirred at –78 °C for 30 min, the reaction mixture was allowed to warm up to rt and stirred overnight. The reaction mixture was cooled to 0 °C, stirred for further 10 min and subsequently treated with distilled water (10 mL). The reaction mixture was diluted with Et_2O (20 mL). The layers were separated and the aqueous layer was extracted twice with Et_2O (20 mL). The combined organic layer was washed with aq. NaOH (0.1 M, 3 x 15 mL), dist. water (3 x 20 mL) and brine (10 mL). The organic layer was dried over Na_2SO_4, concentrated and the obtained oil was dried under high vacuum.
The residue was dissolved in DCM (25mL) without purification and *m*-CPBA (12.0 g, 70-75% stabilized in water, 2.5 equiv.) was dissolved in dry DCM (150 mL) and was added into the mixture solution dropwise at 0 °C. The progress of the reaction was monitored by TLC. (approx. 3-4 hours). Upon completion, sat. $NaHCO_3$ (42 mL) was added to the solution and the reaction mixture was stirred for 1 h until no gas was produced. Then the mixture was extracted with DCM (3×15 mL) and the organic layer was washed by sat. $NaHCO_3$, brine, dried (Na_2SO_4) and concentrated *in vacuo*. The crude was purified by flash column chromatography (3→ 10% Et_2O in pentane) to give sulfone **11** as a colorless, viscous oil (4.8 g, 15 mmol, 75% yield).
$\mathbf{R_f}$ = 0.47 (10% Et_2O/pentane).
^{1}H NMR (400 MHz, $CDCl_3$) δ 8.04 – 8.00 (m, 2H, Ar*H*), 7.71 – 7.62 (m, 1H, Ar*H*), 7.62 – 7.53 (m, 2H, Ar*H*), 1.18 – 1.05 (m, 3H, SiC*H*), 1.02 (d, *J* = 5.9 Hz, 18H, SiCH(CH_3)$_2$).
^{13}C NMR (101 MHz, $CDCl_3$) δ 142.3, 134.2, 129.4, 127.3, 101.1, 100.8, 18.2, 11.0.
All spectroscopic data in accordance with the literature.[3]

2.4. Preparation of EBX reagents

2.4.1. Preparation of EBX.1

Triisopropyl((3-(phenylsulfonyl)bicyclo[1.1.1]pentan-1-yl)ethynyl)silane (12)

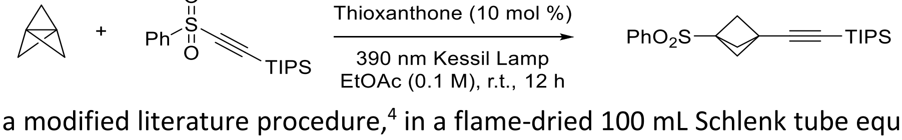

Following a modified literature procedure,[4] in a flame-dried 100 mL Schlenk tube equipped with a stirring bar, thioxanthone (106 mg, 0.500 mmol, 10 mol %) was charged under air. Then, the Schlenk tube was evacuated and re-filled with nitrogen three times. After that, the alkynyl sulfone

[3] L. Gnägi, S. V. Martz, D. Meyer, R. M. Schärer, P. Renaud, *Chem. Eur. J.* **2019**, *25*, 11646–11649.
[4] Z. Wu, Y. Xu, H. Zhang, X. Wu, C. Zhu, *Chem. Commun.* **2021**, *57*, 6066–6069.

(3.2 g, 10 mmol, 2.0 equiv.) and dry ethyl acetate (50 mL, 0.1 M) were added, followed by [1.1.1]propellane (8.05 mL, 5.00 mmol, 1.00 equiv., 0.62 M solution in Et_2O). The reaction mixture was then irradiated for 16 h under vigorous stirring at 52 W 390 nm using a Kessil lamp placed at approximately 3 cm distance. Room temperature was maintained using a fan. The crude mixture was evaporated under vacuum, then purified by flash column chromatography (2 → 20% Et_2O in pentane) to yield the product **12** as yellow oil which solidified into a white solid upon standing (1.1 g, 2.8 mmol, 55% yield).
R$_f$ = 0.28 (10% Et_2O/pentane).
^{1}H NMR (400 MHz, $CDCl_3$) δ 7.86 – 7.81 (m, 2H, Ar*H*), 7.68 – 7.63 (m, 1H, Ar*H*), 7.59 – 7.53 (m, 2H, Ar*H*), 2.29 (s, 6H, H_{BCP}), 1.01 (d, *J* = 2.4 Hz, 21H, H_{TIPS}).
^{13}C NMR (101 MHz, $CDCl_3$) δ 136.8, 133.9, 129.3, 128.7, 103.4, 84.1, 55.0, 52.9, 28.7, 18.6, 11.2.
All spectroscopic data in accordance with the literature.[4]

1-Ethynyl-3-(phenylsulfonyl)bicyclo[1.1.1]pentane (13)

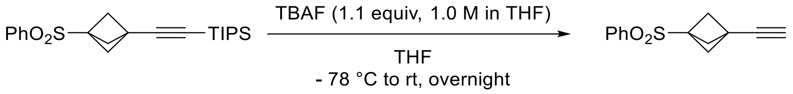

To a solution of TIPS silane-BCP **12** (1.8 g, 4.6 mmol, 1.0 equiv.) in THF (45 mL) at -78 °C, TBAF (5.1 mL, 1.0 M in THF, 5.1 mmol, 1.1 equiv.) was added dropwise then stirred at room temperature for further 3 hours. H_2O was added to quench the reaction. After stirring for 5 min at 0 °C, the resulting aqueous mixture was extracted with Et_2O (50 mL x 3). The combined organic layers were washed with water then dried over Na_2SO_4, and concentrated under reduced pressure. The crude mixture was purified by flash column chromatography (7% → 30% Et_2O in pentane) to yield the desired product **13** (1.0 g, 4.4 mmol, 95% yield) as a white amorphous solid.
R$_f$ = 0.32 (30% Et_2O/pentane).
^{1}H NMR (400 MHz, $CDCl_3$) δ 7.86 – 7.81 (m, 2H, Ar*H*), 7.70 – 7.64 (m, 1H, Ar*H*), 7.60 – 7.54 (m, 2H, Ar*H*), 2.30 (s, 6H, H_{BCP}), 2.17 (s, 1H, C≡C*H*).
^{13}C NMR (101 MHz, $CDCl_3$) δ 136.6, 134.1, 129.4, 128.7, 79.9, 70.9, 54.6, 53.0, 27.9.
HRMS (ESI/QTOF) m/z: $[M + Na]^+$ Calcd for $C_{13}H_{12}NaO_2S^+$ 255.0450; Found 255.0442.

Trimethyl((3-(phenylsulfonyl)bicyclo[1.1.1]pentan-1-yl)ethynyl)silane (14)

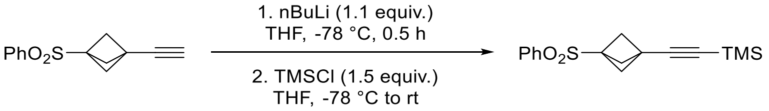

Under a nitrogen atmosphere, 1-ethynyl-3-(phenylsulfonyl)bicyclo[1.1.1]pentane **13** (0.12 g, 0.50 mmol, 1.0 equiv.) was dissolved in dry THF (5 mL) and cooled down to -78 °C. Then, *n*-BuLi (0.34 mL, 1.6 M in hexanes, 0.55 mmol, 1.1 equiv.) was added dropwise to the solution, and the reaction mixture was stirred at -78 °C for 1 h, and chlorotrimethylsilane (82 mg, 95 µL, 0.75 mmol, 1.5 equiv.) was added. The resulting solution was stirred at -78 °C for 1 h. The mixture was quenched with sat. NH_4Cl (10 mL), and the aqueous layer was extracted with EtOAc (2 × 7 mL). The combined organic layers were dried over Na_2SO_4, and concentrated in vacuo. The crude product was purified by flash column chromatography (pentane → 10% EtOAc in pentane) to yield the product **14** (0.13 g, 0.41 mmol, 82% yield) as a white solid.
R$_f$ = 0.17 (5% EtOAc/pentane).

^{1}H NMR (400 MHz, $CDCl_3$) δ 7.88 – 7.80 (m, 2H, Ar*H*), 7.69 – 7.62 (m, 1H, Ar*H*), 7.59 – 7.53 (m, 2H, Ar*H*), 2.28 (s, 6H, H_{BCP}), 0.12 (s, 9H, $Si(CH_3)_3$).
^{13}C NMR (101 MHz, $CDCl_3$) δ 136.7, 134.0, 129.3, 128.7, 101.5, 87.9, 54.8, 53.0, 28.6, -0.1.
HRMS (ESI/QTOF) m/z: $[M + Ag]^+$ Calcd for $C_{16}H_{20}AgO_2SSi^+$ 410.9999; Found 410.9998.

1-((3-(Phenylsulfonyl)bicyclo[1.1.1]pentan-1-yl)ethynyl)-1λ^3-benzo[d][1,2]iodaoxol-3(1H)-one (EBX.1)

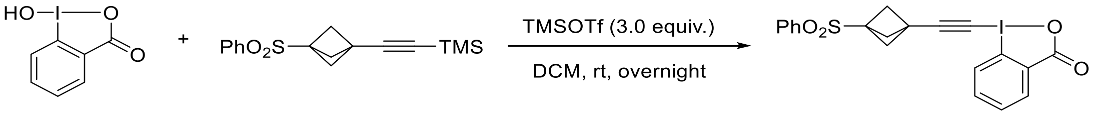

The reaction was conducted in dry glassware under nitrogen atmosphere. To a solution of 1-hydroxy-1λ^3-benzo[*d*][1,2]iodaoxol-3(1*H*)-one **15** (0.30 g, 1.1 mmol, 0.95 equiv.) in dry DCM (6 mL), was added trimethylsilyl trifluoromethanesulfonate (0.67 g, 0.55 mL, 3.6 mmol, 3.0 equiv.) and the reaction was stirred at room temperature for 1 h, before adding trimethyl((3-(phenylsulfonyl)bicyclo[1.1.1]pentan-1-yl)ethynyl)silane **14** (0.37 g, 1.2 mmol, 1.0 equiv.) in dry DCM (6 mL) The reaction was stirred overnight and then quenched with sat. aq. $NaHCO_3$ (15 mL) over 10 min. The layers were separated and the aqueous layer was extracted with DCM (3 x 15 mL). The combined organic layers were washed with sat. aq. $NaHCO_3$ (2 x 15 mL) and brine (20 mL), filtered over celite and dried over Na_2SO_4, filtered, and concentrated in vacuo. The remaining brown solid was triturated with pure n-hexane (3 x 8 mL) using ultrasound. The n-hexane was carefully removed using a syringe. The remaining solid was dried in high vacuum to give **EBX.1** (0.41 g, 0.86 mmol, 72% yield) as a light brown solid. For characterization, the compound can be purified by flash column chromatography (1 → 10% MeOH in DCM).
R_f = 0.23 (5% MeOH/ DCM).
m.p. 107.5–109.3 °C (melt).
^{1}H NMR (400 MHz, $CDCl_3$) δ 8.40 – 8.32 (m, 1H, Ar*H*), 8.06 – 8.00 (m, 1H, Ar*H*), 7.88 – 7.83 (m, 2H, Ar*H*), 7.77 – 7.73 (m, 2H, Ar*H*), 7.72 – 7.67 (m, 1H, Ar*H*), 7.63 – 7.56 (m, 2H, Ar*H*), 2.44 (s, 6H, H_{BCP}).
^{13}C NMR (101 MHz, $CDCl_3$) δ 166.5, 136.3, 135.1, 134.3, 132.7, 131.8, 131.3, 129.5, 128.7, 126.3, 115.7, 102.9, 55.2, 53.2, 45.2, 28.5.
HRMS (ESI/QTOF) m/z: $[M + H]^+$ Calcd for $C_{20}H_{16}IO_4S^+$ 478.9809; Found 478.9815.
IR (ν_{max}, cm^{-1}) 2934 (w), 2923 (w), 1685 (w), 1679 (w), 1664 (m), 1649 (s), 1644 (s), 1619 (s), 1612 (m), 1604 (m).

2.4.2. Preparation of EBX.2

Methyl 3-(hydroxymethyl)bicyclo[1.1.1]pentane-1-carboxylate (17)

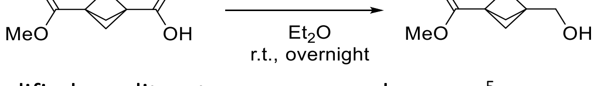

Following a modified literature procedure, [5] commercially available methoxycarbonylbicyclo[1.1.1]pentane-1-carboxylic acid **16** (1.8 g, 10 mmol, 1.0 equiv.) was dissolved in dry Et_2O (57 mL) under nitrogen atmosphere. The solution was cooled to 0 °C, and $BH_3{\cdot}Me_2S$ complex (0.91 g, 6.0 mL, 12 mmol, 2 M in THF, 1.2 equiv.) was added dropwise. After 1

[5] S. O. Kokhan, Y. B. Valter, A. V. Tymtsunik, I. V. Komarov, O. O. Grygorenko, *Eur. J. Org. Chem.* **2017**, *2017*, 6450–6456.

h at 0 °C, the solution was allowed to reach room temperature and stirring was continued overnight. The excess borane was quenched by a careful addition of MeOH (10 mL), and the mixture was concentrated *in vacuo*. Water was added to the residue, and the mixture was extracted with Et_2O (3 x 40 mL). the combined organic layers were dried over anhydrous Na_2SO_4, filtered, and concentrated *in vacuo* to give **17** (1.5 g, 9.6 mmol, 96% yield).
^{1}H NMR (400 MHz, $CDCl_3$) δ 3.68 (s, 3H, OC*H*$_3$), 3.63 (s, 2H, C*H*$_2$), 2.00 (s, 6H, H_{BCP}).
^{13}C NMR (101 MHz, $CDCl_3$) δ 170.7, 62.8, 51.8, 50.3, 40.3, 38.2.
All spectroscopic data in accordance with the literature.[5]

Methyl 3-((trimethylsilyl)ethynyl)bicyclo[1.1.1]pentane-1-carboxylate (21)

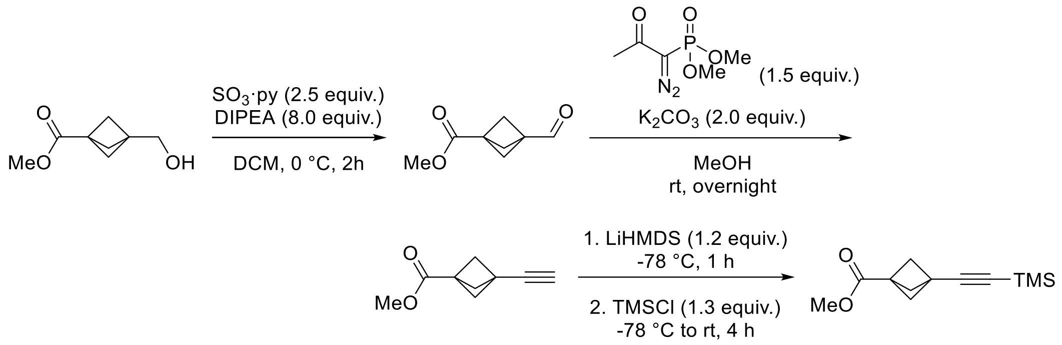

Methyl 3-((trimethylsilyl)ethynyl)bicyclo[1.1.1]pentane-1-carboxylate was prepared over 3 steps. Methyl 3- (hydroxymethyl)bicyclo[1.1.1]pentane-1-carboxylate **17** (1.6 g, 10 mmol, 1.0 equiv.) was dissolved in dry DCM (30 mL) and dry DIPEA (14 mL, 80 mmol, 8.0 equiv.) and the mixture was cooled to 0 °C, followed by the addition of a solution of SO_3·pyridine complex (4.0 g, 25 mmol, 2.5 equiv.) in dry DMSO (15 mL) over 15 min while keeping the temperature below 25 °C. After 2 h at 0 °C, aqueous 1 N aq. HCl (60 mL) was added over 5 min. After layer separation, the aqueous layer was reextracted with DCM (3 x 50 mL), and the combined organic layers were washed sequentially with sat. aq. $NaHCO_3$, water, and brine (70 mL each). The combined organic layers were dried over anhydrous Na_2SO_4, filtered, and concentrated in *vacuo* to yield the crude product **18** as yellow oil (1.5 g, 9.8 mmol, 98% yield), which was used to the next step without further purification.

^{1}H NMR (400 MHz, $CDCl_3$) δ 9.60 (s, 1H, C*H*O), 3.70 (s, 3H, OC*H*$_3$), 2.32 (s, 6H, H_{BCP}).

A flame-dried round-bottom flask was charged with potassium carbonate (2.7 g, 20 mmol, 2.0 equiv.) and set under vacuum for 30 min, and filled with nitrogen. The crude methyl 3-formylbicyclo[1.1.1]pentane-1-carboxylate **18** was dissolved in dry methanol (35 mL) and added to the flask under stirring. Then, 1-diazo-1- [methoxy(methyl)phosphoryl]oxypropan-2-one **19** (2.0 g, 1.6 mL, 11 mmol, 1.1 equiv.) was set under vacuum and refilled with nitrogen 3 times, then dissolved in dry MeOH (18 mL) and added dropwise to the reaction mixture. The reaction mixture was stirred at room temperature overnight. Then, Et_2O (50 mL) and water (50 mL) were added. The layers were separated, and the aqueous layer was extracted with Et_2O (3 × 50 mL). The combined organic layers were washed with water (3 x 100 mL) and dried over anhydrous Na_2SO_4. The solution was concentrated in vacuo to yield the crude product **20** as bright yellow solid (0.95 g, 6.3 mmol, 65% yield), which was used directly to the next step without purification.

^{1}**H NMR** (400 MHz, $CDCl_3$) δ 3.65 (s, 3H, OC*H$_3$*), 2.31 (s, 6H, H_{BCP}), 2.10 (s, 1H, C≡C*H*).

† The product is a volatile solid, therefore putting under high vacuum for a prolonged period might lead to loss of the product.

To a solution of terminal alkyne **20** (0.95 g, 6.3 mmol, 1.0 equiv.) in dry THF (63 mL) was added LiHMDS (8.2 mL, 8.2 mmol, 1.0 M solution in THF, 1.3 equiv.) at -78 °C under nitrogen atmosphere. After stirring for 1 h, TMSCl (1.1 mL, 8.8 mmol, 1.3 equiv.) was added dropwise to the reaction mixture. Then it was gradually warmed up to room temperature and stirred for further 4 h (the reaction progress was monitored by TLC). The reaction was quenched by sat. NH_4Cl. The layers were separated, then the aqueous layer was extracted with DCM (3 x 40 mL). The combined organic layers were washed with water (40 mL) and brine (40 mL), dried over Na_2SO_4, filtered, and concentrated in *vacuo* to afford the desired product **21** as a white solid (905 mg, 4.07 mmol, 65% yield).

^{1}**H NMR** (400 MHz, $CDCl_3$) δ 3.66 (s, 3H, OC*H$_3$*), 2.31 (s, 6H, H_{BCP}), 0.15 (s, 9H, Si*(CH$_3$)$_3$*).
^{13}**C NMR** (101 MHz, $CDCl_3$) δ 170.0, 104.1, 85.5, 56.1, 51.9, 39.7, 28.9, 0.1.
HRMS (Sicrit plasma/LTQ-Orbitrap) m/z: $[M + H]^+$ Calcd for $C_{12}H_{19}O_2Si^+$ 223.1149; Found 223.1146.

Methyl 3-((3-oxo-1λ^3-benzo[d][1,2]iodaoxol-1(3H)-yl)ethynyl)bicyclo[1.1.1]pentane-1-carboxylate (EBX.2)

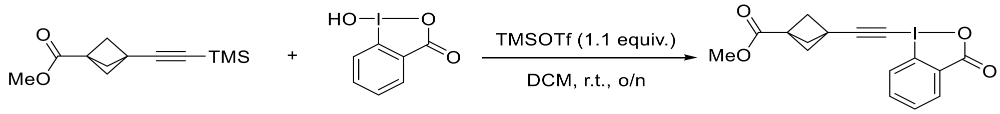

The reaction was conducted in dry glassware under nitrogen atmosphere. To a solution of 1-hydroxy-1λ^3-benzo[*d*][1,2]iodaoxol-3(1*H*)-one **15** (1.0 g, 3.8 mmol, 1.0 equiv) in dry DCM (18 mL), was added trimethylsilyl trifluoromethanesulfonate (2.3 g, 1.9 mL, 11 mmol, 3.0 equiv.) and the reaction was stirred at room temperature for 1 h, before adding methyl 3-((trimethylsilyl)ethynyl)bicyclo[1.1.1]pentane-1-carboxylate **21** (0.84 g, 3.8 mmol, 1.0 equiv.) in dry DCM (18 mL). The reaction was stirred for 5 h and then quenched with sat. aq. $NaHCO_3$ (50 mL) over 10 min. The layers were separated and the aqueous layer was extracted with DCM (3 x 50 mL). The combined organic layers were washed with sat. aq. $NaHCO_3$ (2 x 50 mL) and brine (60 mL), filtered over celite and dried over Na_2SO_4, filtered, and concentrated in vacuo. The remaining brown solid was triturated with pure n-hexane (5 x 5 mL) using ultrasound. The n-hexane was carefully removed using a syringe. The crude was then purified by flash column chromatography (0 → 3% MeOH in DCM) to afford **EBX.2** as a brown solid (1.2 g, 3.0 mmol, 78% yield).
R$_f$ = 0.35 (4% MeOH/DCM).
m.p. 178 °C (decomp.)
^{1}**H NMR** (400 MHz, $CDCl_3$) δ 8.32 (dd, *J* = 7.1, 2.0 Hz, 1H, Ar*H*), 8.11 – 8.05 (m, 1H, Ar*H*), 7.78 – 7.67 (m, 2H, Ar*H*), 3.65 (s, 2H, OC*H$_3$*), 2.42 (d, *J* = 1.5 Hz, 6H, H_{BCP}).
^{13}**C NMR** (101 MHz, $CDCl_3$) δ 169.0, 166.6, 135.0, 132.6, 131.8, 131.4, 126.3, 115.7, 105.8, 56.4, 52.1, 42.4, 40.1, 28.8.
IR (ν_{max}, cm^{-1}) 3509 (w), 3448 (w), 1699 (w), 1695 (w), 1689 (w), 1684 (w), 1679 (w), 1669 (m), 1664 (m), 1628 (s), 1619 (m), 1601 (w).

HRMS (Sicrit plasma/LTQ-Orbitrap) m/z: $[M + H]^+$ Calcd for $C_{16}H_{14}IO_4^+$ 396.9931; Found 396.9930.

2.4.3. Preparation of EBX.3 and EBX.4

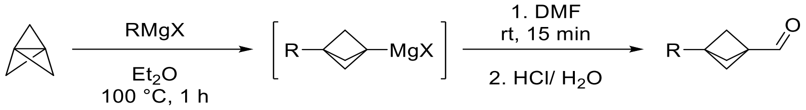

Following a reported literature procedure,[2] an oven dried 20 mL vial was loaded with Mg (340 mg, 14.0 mmol, 4.0 equiv.), a piece of iodine, and a stirring bar. The vial was evacuated and refilled with nitrogen 3 times. Then, Et_2O (10 mL) was added, followed by 1/3 of the aryl bromide (10 mmol, 2.0 equiv.). The reaction was briefly stirred until the exothermic reaction started. Upon reflux, the rest of the aryl bromide was added dropwise over 30 min. After the complete addition of aryl bromide, the reaction was heated to 40 °C for 2 h. The Grignard reagent was transferred to a 100 mL Ace pressure flask via a syringe, and [1.1.1]propellane (1.0 equiv., 0.7-0.9 M in Et_2O) was added. The flask was stirred for 1 h at 100 °C, and it was cooled down to rt. Anhydrous DMF (3.1 mL, 40 mmol, 8.0 equiv.) was added dropwise very slowly with intensive stirring. Once the addition was completed, the reaction was stirred for further 15 min at rt. Then, it was poured into 130 mL of 1:3 HCl (37%):H_2O. The aqueous layer was extracted with EtOAc (3 x 130 mL), washed with sat. $NaHCO_3$ (130 mL), H_2O (130 mL), brine (130 mL), and dried over Na_2SO_4. The solvents were evaporated under reduced pressure, and the crude product was purified by flash column chromatography on silica gel.

3-Phenylbicyclo[1.1.1]pentane-1-carbaldehyde (22)

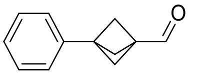

Synthesized using phenyl bromide (1.6 g, 1.1 mL, 10 mmol) to afford the desired product as a yellow oil (0.36 g, 2.1 mmol, 41% yield).
R_f = 0.46 (10% EtOAc/pentane); visualisation by *p*-anisaldehyde.
^{1}H NMR (400 MHz, $CDCl_3$) δ 9.69 (s, 1H, C*HO*), 7.37 – 7.30 (m, 2H, Ar*H*), 7.29 – 7.21 (m, 3H, Ar*H*), 2.32 (s, 6H, H_{BCP}).
^{13}C NMR (101 MHz, $CDCl_3$) δ 199.3, 139.8, 128.5, 127.2, 126.1, 52.3, 43.4, 42.7.
All spectroscopic data in accordance with the literature.[2]

3-Benzylbicyclo[1.1.1]pentane-1-carbaldehyde (23)

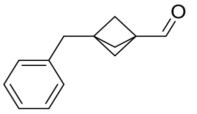

Synthesized using benzyl bromide (1.7 g, 1.2 mL, 10 mmol) to afford the desired product (0.19 g, 1.1 mmol, 21% yield). The crude product was purified by flash column chromatography, but it was not possible to separate out from some impurity.
R_f = 0.48 (10% EtOAc/pentane); visualisation by *p*-anisaldehyde.
^{1}H NMR (400 MHz, $CDCl_3$) δ 9.45 (s, 1H, C*HO*), 7.26 – 7.17 (m, 3H, Ar*H*), 7.03 – 6.99 (m, 2H, Ar*H*), 2.73 (s, 2H, $PhCH_2$), 1.78 (s, 6H, H_{BCP}).
^{13}C NMR (101 MHz, $CDCl_3$) δ 199.2, 138.6, 129.0, 128.5, 127.6, 50.7, 50.4, 41.3, 39.1.

HRMS (Sicrit plasma/LTQ-Orbitrap) m/z: $[M + H]^+$ Calcd for $C_{13}H_{15}O^+$ 187.1117; Found 187.1117.

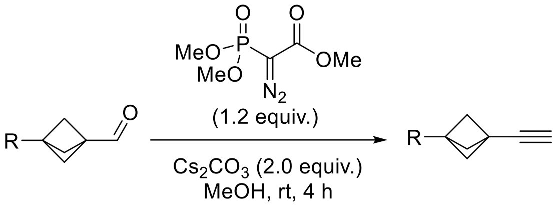

A flame-dried two neck round-bottom flask was charged with K_2CO_3 (2.0 equiv.) and the atmosphere was purged with N_2 (x 3). The corresponding bicyclopentyl aldehyde was measured out into an oven-dried vial and was dissolved in anhydrous MeOH (0.25 M), and added to K_2CO_3. Dimethyl (1-diazo-2-oxopropyl)phosphonate (1.2 equiv.) was also measured out into an oven-dried vial, purged with N_2 (x 3), dissolved in anhydrous MeOH (0.5 M), and then added to the reaction mixture in a dropwise manner. The reaction was then stirred at rt. The progress was monitored by TLC. After the reaction was completed, the reaction mixture was extracted with EtOAc (x 3). The combined organic layers were washed with H_2O, dried over Na_2SO_4, filtered and the solvent was removed under reduced pressure.

1-Ethynyl-3-phenylbicyclo[1.1.1]pentane (24)

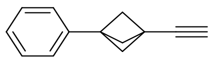

Synthesized using 3-phenylbicyclo[1.1.1]pentane-1-carbaldehyde (0.24 g, 1.4 mmol) to afford the desired product as yellow oil (0.20 g, 1.2 mmol, 86% yield). The crude product was used directly for the next step without purification.

^{1}H NMR (400 MHz, $CDCl_3$) δ 7.33 – 7.27 (m, 2H, Ar*H*), 7.25 – 7.17 (m, 3H, Ar*H*), 2.34 (s, 6H, H_{BCP}), 2.15 (s, 1H, C≡C*H*).

^{13}C NMR (101 MHz, $CDCl_3$) δ 139.8, 128.2, 126.8, 126.0, 83.2, 68.1, 56.3, 44.0, 27.1.

All spectroscopic data in accordance with the literature.[2]

1-Benzyl-3-ethynylbicyclo[1.1.1]pentane (25)

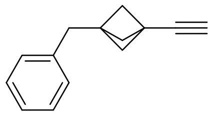

Synthesized using 3-benzylbicyclo[1.1.1]pentane-1-carbaldehyde to afford the desired product as a yellow oil (0.17 g, 0.92 mmol, 46% yield). The crude product was purified by flash column chromatography, but it was not possible to separate out from some impurity.

R$_f$ = 0.56 (2% Et_2O/pentane).

^{1}H NMR (400 MHz, $CDCl_3$) δ 7.38 – 7.27 (m, 2H, Ar*H*), 7.23 – 7.17 (m, 1H, Ar*H*), 7.09 – 7.04 (m, 2H, Ar*H*), 2.73 (s, 2H, PhC*H$_2$*), 2.06 (s, 1H, C≡C*H*), 1.89 (s, 6H, HBCP).

C NMR analyzed by correlation with 2D NMR

^{13}C NMR (101 MHz, $CDCl_3$) δ 138.9, 128.7, 128.4, 126.9, 70.6, 67.7, 54.6, 42.6, 38.7, 28.5.

HRMS (APCI/QTOF) m/z: $[M + Ag]^+$ Calcd for $C_{14}H_{14}Ag^+$ 289.0141; Found 289.0147.

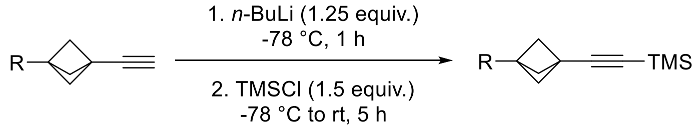

Under nitrogen atmosphere, BCP alkyne (1.0 equiv.) was dissolved in dry THF (0.1 M) and cooled down to -78 °C. Then, *n*-BuLi (2.5 M in hexanes, 1.25 equiv.) was added dropwise to the solution, and the reaction mixture was stirred at -78 °C for 1 h, and chlorotrimethylsilane (1.5 equiv.) was added. The resulting solution was stirred at -78 °C for 1 h then warmed up to rt then stirred for further 5 h. The mixture was quenched with sat. NH_4Cl and the aqueous layer was extracted with EtOAc (x 3). The combined organic layers were dried over Na_2SO_4, filtered and concentrated in vacuo.

Trimethyl((3-phenylbicyclo[1.1.1]pentan-1-yl)ethynyl)silane (26)

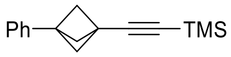

Synthesized from 1-ethynyl-3-phenylbicyclo[1.1.1]pentane (0.20 g, 1.2 mmol) to afford the desired product as a white solid (0.26 g, 1.1 mmol, 89% yield). The crude product was used directly to the next step without purification.

^{1}H NMR (400 MHz, $CDCl_3$) δ 7.32 – 7.27 (m, 2H, Ar*H*), 7.25 – 7.15 (m, 3H, Ar*H*), 2.33 (s, 6H, H_{BCP}), 0.18 (s, 9H, Si*(CH$_3$)$_3$*).

^{13}C NMR (101 MHz, $CDCl_3$) δ 140.2, 128.3, 126.9, 126.1, 105.5, 84.7, 56.7, 44.0, 28.0, 0.2.

HRMS (Sicrit plasma/LTQ-Orbitrap) m/z: $[M + H]^+$ Calcd for $C_{16}H_{21}Si^+$ 241.1407; Found 241.1406.

((3-Benzylbicyclo[1.1.1]pentan-1-yl)ethynyl)trimethylsilane (27)

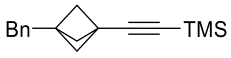

Synthesized using 1-benzyl-3-ethynylbicyclo[1.1.1]pentane (0.32 g, 1.7 mmol) to afford the desired product as a white solid (0.21 g, 0.84 mmol, 49% yield). The crude product was purified by flash column chromatography (pentane → 5% Et_2O in pentane).

R_f = 0.19 (pentane).

^{1}H NMR (400 MHz, $CDCl_3$) δ 7.30 – 7.23 (m, 2H, Ar*H*), 7.22 – 7.15 (m, 1H, Ar*H*), 7.08 – 7.03 (m, 2H, Ar*H*), 2.71 (s, 2H, PhC*H$_2$*), 1.87 (s, 6H, H_{BCP}), 0.12 (s, 9H, Si*(CH$_3$)$_3$*).

^{13}C NMR (101 MHz, $CDCl_3$) δ 139.0, 129.0, 128.4, 126.1, 105.7, 84.1, 54.9, 42.5, 38.8, 29.3, 0.2.

HRMS (Sicrit plasma/LTQ-Orbitrap) m/z: $[M]^+$ Calcd for $C_{17}H_{22}Si^+$ 254.1485; Found 254.1485.

Synthesis of EBX.3 and EBX.4

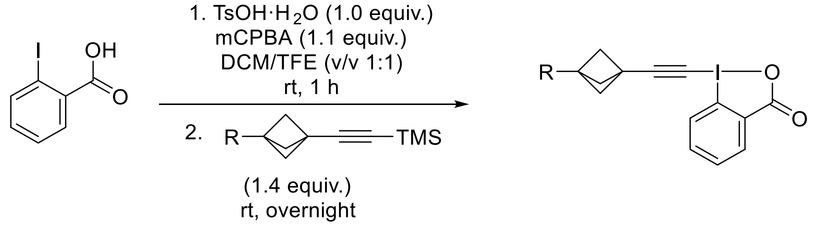

Following a slightly modified procedure,[6] 2-iodobenzoic acid **28** (1.0 equiv.), *p*-toluenesulfonic acid monohydrate (1.0 equiv.) and *m*CPBA (70 wt%, 1.1 equiv.) were dissolved in DCM and 2,2,2-trifluoroethanol (1:1 v/v, 0.25 M). The mixture was stirred at room temperature under nitrogen for 1 hour, after which BCP alkyne (1.4 equiv.) was added in one portion. The reaction mixture was stirred for 14 h at room temperature, filtered and concentrated in vacuo. The resulting oil

[6] (a) M. J. Bouma, B. Olofsson, *Chem. Eur. J.* **2012**, *18*, 14242–14245. (b) T. M. Milzarek, N. P. Ramirez, X.-Y. Liu, J. Waser, *Chem. Commun.* **2023**, 59, 12637–12640.

was redissolved in DCM and under vigorous stirring, saturated aq. $NaHCO_3$ was added. The mixture was stirred for 1 h. Then the layers were separated, and the aqueous layer was extracted with DCM (x 3). The combined organic layers were dried over Na_2SO_4, filtered and concentrated *in vacuo*. The crude product was purified by flash column chromatography (DCM → 5% MeOH in DCM).

1-((3-Phenylbicyclo[1.1.1]pentan-1-yl)ethynyl)-1λ³-benzo[*d*][1,2]iodaoxol-3(1*H*)-one (EBX.3)

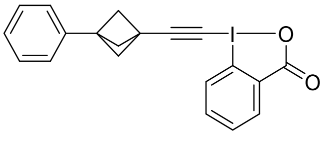

Synthesized using trimethyl((3-phenylbicyclo[1.1.1]pentan-1-yl)ethynyl)silane (0.17 g, 0.70 mmol) to afford the desired product as pale-yellow solid (71 mg, 92 µmol, 34% yield).
R_f = 0.24 (5% MeOH in DCM).
m.p. 88.5–90.2 °C (melt).
^{1}H NMR (400 MHz, $CDCl_3$) δ 8.44 – 8.37 (m, 1H, Ar*H*), 8.20 – 8.13 (m, 1H, Ar*H*), 7.85 – 7.73 (m, 2H, Ar*H*), 7.37 – 7.29 (m, 2H, Ar*H*), 7.30 – 7.24 (m, 1H, Ar*H*), 7.22 – 7.17 (m, 2H, Ar*H*), 2.48 (s, 6H, H_{BCP}).
^{13}C NMR (101 MHz, $CDCl_3$) δ 166.6, 139.0, 135.0, 132.6, 131.7, 131.5, 128.5, 127.4, 126.3, 126.1, 115.8, 107.4, 57.1, 44.7, 41.2, 28.0.
IR ($ν_{max}$, cm^{-1}) 2992 (w), 2983 (w), 2972 (w), 2968 (w), 2966 (w), 2961 (w), 2910 (w), 2871 (w), 2149 (w), 1705 (w), 1701 (w), 1696 (w), 1688 (w), 1685 (w), 1679 (w), 1665 (m), 1649 (s), 1623 (s), 1605 (m).
HRMS (Sicrit plasma/LTQ-Orbitrap) m/z: $[M + H]^+$ Calcd for $C_{20}H_{16}IO_2^+$ 415.0190; Found 415.0189.

1-((3-Benzylbicyclo[1.1.1]pentan-1-yl)ethynyl)-1λ³-benzo[*d*][1,2]iodaoxol-3(1*H*)-one (EBX.4)

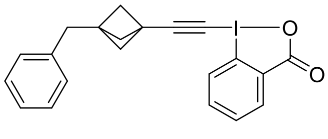

Synthesized using ((3-benzylbicyclo[1.1.1]pentan-1-yl)ethynyl)trimethylsilane (0.47 g, 1.8 mmol) to afford the desired product as a light orange solid(0.14 g, 0.33 mmol, 18% yield).
R_f = 0.25 (5% MeOH in DCM).
m.p. 58.4–64.1 °C (melt).
^{1}H NMR (400 MHz, $CDCl_3$) δ 8.45 – 8.34 (m, 1H, Ar*H*), 8.11 – 8.03 (m, 1H, Ar*H*), 7.79 – 7.67 (m, 2H, Ar*H*), 7.33 – 7.27 (m, 2H, Ar*H*), 7.25 – 7.20 (m, 1H, Ar*H*), 7.10 – 7.05 (m, 2H, Ar*H*), 2.78 (s, 2H, PhC*H₂*), 2.03 (s, 6H, H_{BCP}).
^{13}C NMR (101 MHz, $CDCl_3$) δ 166.6, 138.4, 134.9, 132.6, 131.7, 131.5, 129.0, 128.6, 126.4, 126.2, 115.8, 107.6, 55.2, 43.3, 40.4, 38.5, 29.3.
IR ($ν_{max}$, cm^{-1}) 2991 (w), 2979 (w), 2969 (w), 2966 (w), 2961 (w), 2928 (w), 2924 (w), 2910 (w), 2904 (w), 2871 (w), 2140 (w), 1685 (w), 1679 (w), 1648 (s), 1643 (s), 1622 (s), 1606 (m).
HRMS (ESI/QTOF) m/z: $[M + Na]^+$ Calcd for $C_{21}H_{17}INaO_2^+$ 451.0165; Found 451.0183.

2.5. Preparation of sulfonamides

General procedure 1 for the preparation of sulfonamides

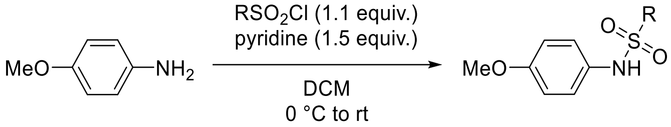

Sulfonamides were synthesized according to a reported procedure.[7] Under nitrogen atmosphere, *p*-anisidine (1.0 equiv.) and dry pyridine (1.5 equiv.) were dissolved in DCM (0.5 M) at 0 °C. Then, the corresponding sulfonyl chloride (1.1 equiv.) was slowly added, and the reaction mixture was warmed to room temperature. The conversion of the starting material was checked by TLC. After the reaction was completed, the reaction was quenched by water (2 x volume of solvent). The aqueous layer was extracted with DCM (x 3). The combined organic layers were dried over Na_2SO_4, filtered, and concentrated under reduced pressure. The crude material was purified by flash column chromatography (pentane/ EtOAc) to afford the desired sulfonamides.

***N*-(4-methoxyphenyl)-4-methylbenzenesulfonamide**

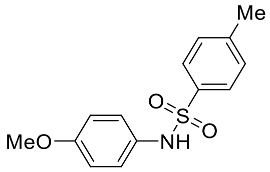

Synthesized following GP1 using anisidine (0.31 g, 0.28 mL, 2.5 mmol), tosyl chloride (0.52 g, 2.8 mmol) and pyridine (0.30 g, 0.30 mL, 3.8 mmol) to afford the desired product (0.56 g, 2.0 mmol, 81% yield) as a pale orange solid.

$\mathbf{R_f}$ = 0.52 (30% EtOAC/ pentane).

^{1}H NMR (400 MHz, $CDCl_3$) δ 7.57 (d, *J* = 8.3 Hz, 2H, Ar*H*), 7.24 – 7.18 (m, 2H, Ar*H*), 6.99 – 6.92 (m, 2H, Ar*H*), 6.80 – 6.72 (m, 2H, Ar*H*), 3.76 (s, 3H, OC*H*$_3$), 2.39 (s, 3H, ArC*H*$_3$).

^{13}C NMR (101 MHz, $CDCl_3$) δ 158.2, 143.8, 136.2, 129.7, 128.9, 127.5, 125.8, 114.6, 55.6, 21.7.

All spectroscopic data in accordance with the literature.[8]

***N*-(4-methoxyphenyl)methanesulfonamide**

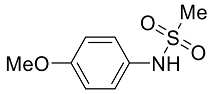

Synthesized following GP1 using anisidine (0.62 g, 5.0 mmol), methanesulfonyl chloride (0.63 g, 0.43 mL, 5.5 mmol) and pyridine (0.59 g, 0.61 mL, 7.5 mmol) to afford the desired product (0.88 g, 3.2 mmol, 63% yield) as a pale purple solid.

$\mathbf{R_f}$ = 0.25 (30% EtOAC/ pentane).

^{1}H NMR (400 MHz, $CDCl_3$) δ 7.23 – 7.15 (m, 2H, Ar*H*), 6.92 – 6.84 (m, 2H, Ar*H*), 3.83 – 3.78 (m, 3H, OC*H*$_3$), 2.95 (s, 3H, SO_2C*H*$_3$).

^{13}C NMR (101 MHz, $CDCl_3$) δ 158.3, 129.1, 125.0, 115.0, 55.7, 39.0.

All spectroscopic data in accordance with the literature.[7]

***N*-(4-methoxyphenyl)-4-nitrobenzenesulfonamide**

[7] U. Kloeckner, B. J. Nachtsheim, *Chem. Commun.* **2014**, *50*, 10485–10487.

[8] K. Liu, G. Wang, Z.-W. Zhang, Y.-Y. Shi, Z.-S. Ye, *Org. Lett.* **2022**, *24*, 6489–6493.

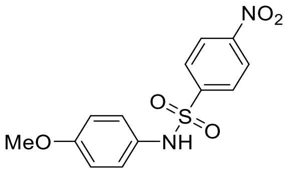

460

Synthesized following GP1 using anisidine (0.62 g, 5.0 mmol), 4-nitrobenzenesulfonyl chloride chloride (1.2 g, 5.5 mmol) and pyridine (0.59 g, 0.61 mL, 7.5 mmol) to afford the desired product (0.81 g, 2.6 mmol, 52% yield) as a pale orange solid.
R$_f$ = 0.24 (30% EtOAC/ pentane).
^{1}H NMR (400 MHz, DMSO) δ 10.24 (s, 1H, N*H*), 8.40 – 8.32 (m, 2H, Ar*H*), 7.94 – 7.86 (m, 2H, Ar*H*), 7.02 – 6.93 (m, 2H, Ar*H*), 6.86 – 6.78 (m, 2H, Ar*H*), 3.67 (s, 3H, OC*H$_3$*).
^{13}C NMR (101 MHz, DMSO) δ 157.0, 149.7, 144.9, 129.2, 128.3, 124.6, 124.1, 114.5, 55.2.
All spectroscopic data in accordance with the literature.[9]

3. Optimization of the synthesis of VBX reagents

In a glass vial, the correspondent sulfonamide or phenol (0.300 mmol, 1.00 equiv.) was dissolved in 3.75 mL of DCM (0.08 M). Cs_2CO_3 was added and the mixture stirred vigorously for 5 min. Then the corresponding EBX was added in one portion (0.300 mmol, 1.00 equiv.) and the reaction was left stirring for 12 h. After the reaction was stopped, DCM was removed under reduced pressure. The NMR yield was determined by addition of dibromomethane (6.9 µL, 0.10 mmol) using the singlet at δ 4.9 (2H) ppm and integrating the vinyl H at δ 6.88 (s, 1H).

Table S1. Optimization of N-VBX formation[a]

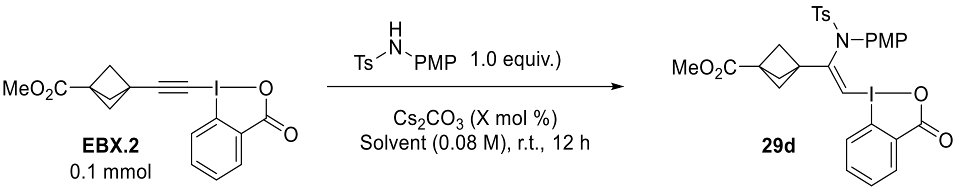

Entry	Solvent	Base / mol %	Conversion / %	Yield % 29d
1	EtOH	10	29	7
2	THF	10	Full	Decomposition to iodobenzoic acid
3	DCM	0	0	0
4	DCM	10	40	9
**5**	**DCM**	**25**	**Full**	**93(86)**[b]
6	DCM	50	Full	50

[a] **EBX.2** (0.100 mmol), sulfonamide (0.100 mmol), Cs_2CO_3 (XX mol%), and solvent (0.08 M) at 25 °C. NMR yield given, calculated using 0.1 mmol of dibromomethane as internal standard. [b] Isolated yield.

In a round bottomed flask, the correspondent thiol (0.100 mmol, 1.00 equiv.) was dissolved in 12.5 mL of EtOH: DCM (1:1 v/v, 0.08 M). Cs_2CO_3 was added and the mixture stirred vigorously for

[9] K. Endo, H. Ube, M. Shionoya, *J. Am. Chem. Soc.* **2020**, *142*, 407–416.

5 min. Then the corresponding EBX was added in one portion (0.100 mmol, 1.00 equiv.) and the flask was sealed with a teflon cap. The reaction was left stirring for 1 h. The reaction was stopped, the solvent removed under reduced pressure. The crude was redissolved in DCM and sat. $NaHCO_3$ was added. The layers were separated, and the aqueous layer was extracted with DCM (3 x 3 mL). The combined organic layers were washed with sat. $NaHCO_3$ (3 mL), H_2O (3 mL), brine (3 mL), and dried over Na_2SO_4. The solvent was removed under reduced pressure. The NMR yield was determined by addition of dibromomethane (6.9 µL, 0.10 mmol) and integrating the vinyl H at δ 7.02 (s, 1H). The NMR yield of the side product **36** was determined by integrating the vinyl H at δ 6.76 (s, 1H).

Table S2. Optimization of S-VBX formation[a]

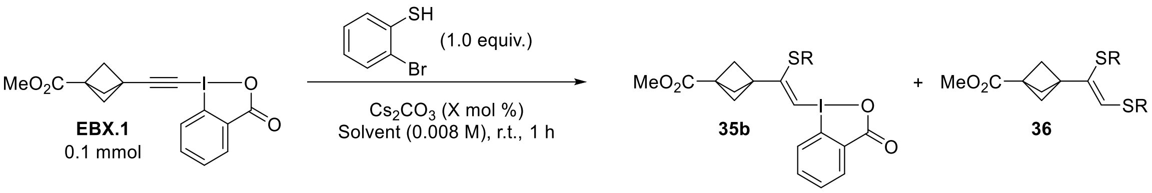

Entry	Base / mol %	Solvent	Additive	Yield % 35b	Yield % 36
1	0	EtOH	-	37	28
2	10	EtOH	-	33	53
3	0	EtOH	BHT (1.0 equiv.)	53	24
4	0	DCM	-	37	3
5	0	*i*PrOH	-	22	10
6	10	EtOH	BHT (1.0 equiv.)	50	35
7	10	DCM	-	23	8
8	10	EtOH	BHT (1.5 equiv.)	54	25
9	0	EtOH/ DCM 1:1	-	37	15
**10**	**10**	**EtOH/ DCM 1:1**	**-**	**56**	**33**
11[b]	10	EtOH/ DCM 1:1	-	27	41
12	10	EtOH/ DCM 1:1	BHT (1.0 equiv.)	54	29
13	10	EtOH/ DCM 1:4	-	39	33
14[c]	10	EtOH/ DCM 1:1	-	36	33

[a] **EBX.2** (0.100 mmol), 2-bromothiophenol (0.100 mmol), Cs_2CO_3 (XX mol%), and solvent (0.008 M) at 25 °C. NMR yield given, calculated using 0.1 mmol of dibromomethane as internal standard. [b] Solvent concentration 0.08 M. [c]Reaction conducted at -50 °C

4. Scope of N-VBX, N-VBX, and S-VBXs

4.1. General Procedure GP2 for the synthesis of N-VBX and O-VBX

In a glass vial, the correspondent sulfonamide or phenol (0.300 mmol, 1.00 equiv.) was dissolved in 3.75 mL of DCM (0.08 M). Cs_2CO_3 (24.4 mg, 25 mol%, 25.0 µmol) was added and the mixture stirred vigorously for 5 min. Then the corresponding EBX was added in one portion (0.300 mmol, 1.00 equiv.) and the reaction was left stirring for 12 h. After the reaction was stopped, DCM was removed under reduced pressure and the crude purified via column chromatography using DCM:MeOH (20:1) as eluent.

(Z)-*N*-(4-methoxyphenyl)-4-methyl-N-(2-(3-oxo-1λ³-benzo[*d*][1,2]iodaoxol-1(3*H*)-yl)-1-(3-(phenylsulfonyl)bicyclo[1.1.1]pentan-1-yl)vinyl)benzenesulfonamide (29a)

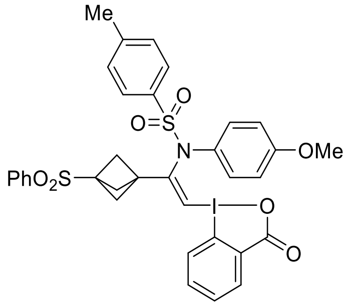

Starting from **EBX.1** (144 mg, 0.300 mmol), **29a** (194 mg, 0.257 mmol, 86% yield) was obtained, as a light-yellow solid.
1 mmol scale reaction: Starting from **EBX.1** (478 mg, 1.00 mmol), **29a** (639 mg, 0.846 mmol, 85% yield) was obtained, as a light-yellow solid.
R_f = 0.25 (5% MeOH/ DCM).
m.p. 112.5–130.3 °C (decomp.).
^{1}H NMR (400 MHz, $CDCl_3$) δ 8.41 (dt, *J* = 6.8, 1.8 Hz, 1H, Ar*H*), 7.81 – 7.75 (m, 2H, Ar*H*), 7.72 – 7.53 (m, 8H, Ar*H*), 7.31 – 7.27 (m, 2H, Ar*H*), 7.03 – 6.95 (m, 2H, Ar*H*), 6.92 – 6.89 (m, 1H, vinyl*H*), 6.76 – 6.70 (m, 2H, Ar*H*), 3.75 (s, 3H, OC*H₃*), 2.43 (s, 3H, ArC*H₃*), 2.03 (d, *J* = 9.2 Hz, 6H, HBCP).
^{13}C NMR (101 MHz, $CDCl_3$) δ 166.9 (*C*=O), 159.8 (C_{Ar}-OMe), 151.4 (N*C*=CHI), 145.9 (C_{Ar}), 137.1 (C_{Ar}), 136.2 (C_{Ar}), 135.5 (C_{Ar}), 134.3 (C_{Ar}), 134.0 (C_{Ar}), 133.8 (C_{Ar}), 133.2 (C_{Ar}), 133.0 (C_{Ar}), 131.1 (C_{Ar}), 130.8 (C_{Ar}), 130.3 (C_{Ar}), 130.3 (C_{Ar}), 129.7 (C_{Ar}), 129.5 (C_{Ar}), 128.7 (C_{Ar}), 128.5 (C_{Ar}), 128.3 (C_{Ar}), 125.7 (C_{Ar}), 115.2 (C_{Ar}), 115.1 (C_{Ar}), 109.1 (NC=*C*HI), 55.7 (O*C*H₃), 53.4 (C_{BCP}), 51.1 (*C*SO₂Ph), 41.7 (NC*C*), 21.9 (C_{Ar}-*C*H₃).
IR (ν_{max}, cm^{-1}) 3066 (w), 3061 (w), 3057 (w), 3051 (w), 3047 (w), 3033 (w), 3027 (w), 3022 (w), 3018 (w), 3013 (w), 3004 (w), 2997 (w), 2974 (w), 2969 (w), 2924 (w), 2916 (w), 1642 (m), 1622 (s), 1601 (m).
HRMS (nanochip-ESI/LTQ-Orbitrap) m/z: $[M + H]^+$ Calcd for $C_{34}H_{31}INO_7S_2^+$ 756.0581; Found 756.0573.

(Z)-N-(4-methoxyphenyl)-N-(2-(3-oxo-1λ³-benzo[*d*][1,2]iodaoxol-1(3*H*)-yl)-1-(3-(phenylsulfonyl)bicyclo[1.1.1]pentan-1-yl)vinyl)methanesulfonamide (29b)

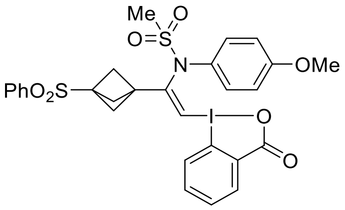

Starting from **EBX.1** (144 mg, 0.300 mmol), **29b** (176 mg, 0.259 mmol, 86% yield) was obtained, as a light-yellow solid.

R$_f$ = 0.25 (5% MeOH/ DCM).

m.p. 173.3–191.1 °C (melt).

^{1}H NMR (400 MHz, $CDCl_3$) δ 8.47 – 8.38 (m, 1H, Ar*H*), 7.84 – 7.78 (m, 2H, Ar*H*), 7.72 – 7.62 (m, 3H, Ar*H*), 7.61 – 7.53 (m, 2H, Ar*H*), 7.39 – 7.33 (m, 1H, Ar*H)*, 7.25 – 7.18 (m, 2H, Ar*H*), 6.93 (s, 1H, vinyl*H*), 6.90 – 6.81 (m, 2H, Ar*H*), 3.80 (s, 3H, SO_2CH_3), 3.13 (s, 3H, OCH_3), 2.14 (s, 6H, HBCP).

^{13}C NMR (101 MHz, $CDCl_3$) δ 166.7, 159.8, 150.2, 136.3, 134.3, 134.0, 133.8, 133.4, 131.3, 130.0, 129.5, 128.7, 126.8, 125.9, 115.7, 114.8, 109.6, 55.8, 53.2, 51.1, 41.6, 39.9.

IR (ν_{max}, cm^{-1}) 3020 (w), 3016 (w), 3003 (w), 2999 (w), 2935 (w), 2931 (w), 2921 (w), 2916 (w), 1642 (m), 1622 (s), 1606 (m).

HRMS (ESI/QTOF) m/z: $[M + H]^+$ Calcd for $C_{28}H_{27}INO_7S_2^+$ 680.0268; Found 680.0291.

(Z)-*N*-(4-methoxyphenyl)-4-nitro-*N*-(2-(3-oxo-1λ^3-benzo[*d*][1,2]iodaoxol-1(3*H*)-yl)-1-(3-(phenylsulfonyl)bicyclo[1.1.1]pentan-1-yl)vinyl)benzenesulfonamide (29c)

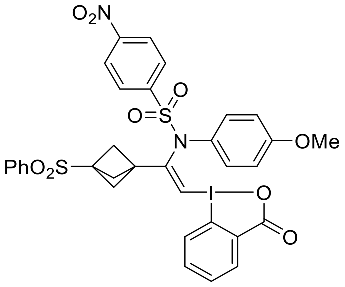

Starting from **EBX.1** (144 mg, 0.300 mmol), **29c** (172 mg, 0.219 mmol, 73% yield) was obtained, as a bright orange solid.

R$_f$ = 0.17 (5% MeOH/ DCM).

m.p. 153.1–170.1 °C (melt).

^{1}H NMR (400 MHz, $CDCl_3$) δ 8.38 – 8.27 (m, 3H, Ar*H*), 7.97 – 7.90 (m, 2H, Ar*H*), 7.80 – 7.75 (m, 2H, Ar*H*), 7.71 – 7.66 (m, 1H, Ar*H*), 7.63 – 7.53 (m, 4H, Ar*H*), 7.30 – 7.24 (m, 1H, Ar*H*), 7.07 (s, 1H, vinyl*H*), 7.02 – 6.96 (m, 2H, Ar*H*), 6.78 – 6.72 (m, 2H, Ar*H*), 3.75 (s, 3H, OCH_3), 2.09 (s, 6H, HBCP).

^{13}C NMR (101 MHz, $CDCl_3$) δ 166.8, 160.3, 150.9, 150.9, 144.0, 136.1, 134.4, 134.0, 133.6, 133.1, 131.3, 129.6, 129.6, 129.2, 128.7, 128.7, 125.9, 124.8, 115.5, 115.2, 110.2, 55.7, 53.4, 51.1, 41.7.

IR (ν_{max}, cm^{-1}) 3101 (w), 3060 (w), 3054 (w), 3020 (w), 3010 (w), 3004 (w), 1650 (m), 1642 (m), 1623 (s), 1606 (m).

HRMS (ESI/QTOF) m/z: $[M + H]^+$ Calcd for $C_{33}H_{28}IN_2O_9S_2^+$ 787.0275; Found 787.0295.

Methyl (Z)-3-(1-((N-(4-methoxyphenyl)-4-methylphenyl)sulfonamido)-2-(3-oxo-1λ^3-benzo[*d*][1,2]iodaoxol-1(3*H*)-yl)vinyl)bicyclo[1.1.1]pentane-1-carboxylate (29d)

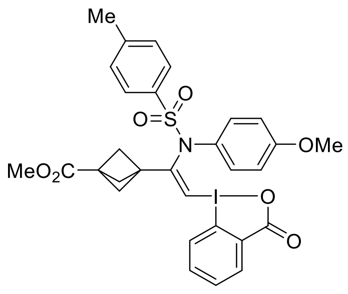

Starting from **EBX.2** (119 mg, 0.300 mmol), **29d** (144 mg, 0.214 mmol, 71% yield) was obtained, as a brown solid. The reaction was conducted in DCM (0.008 M). Purified in deactivated silica; decomposition upon silica gel was observed.

R$_f$ = 0.25 (5% MeOH/ DCM).

m.p. 109.2–111.3 °C (melt).

^{1}H NMR (400 MHz, $CDCl_3$) δ 8.51 – 8.43 (m, 1H, Ar*H*), 7.72 – 7.62 (m, 4H, Ar*H*), 7.47 – 7.38 (m, 1H, Ar*H*), 7.36 – 7.29 (m, 2H, Ar*H*), 7.06 – 7.00 (m, 2H, Ar*H*), 6.96 (s, 1H, vinyl*H*), 6.81 – 6.71 (m, 2H, Ar*H*), 3.75 (s, 3H, OCH_3), 3.63 (s, 3H, OCH_3), 2.45 (s, 3H, ArCH_3), 2.03 (s, 6H, H_{BCP}).

^{13}C NMR (101 MHz, $CDCl_3$) δ 169.2, 167.4, 159.7, 153.2, 145.6, 136.1, 133.9, 133.4, 131.1, 130.6, 130.3, 128.6, 128.3, 125.9, 115.4, 115.0, 107.0, 55.6, 54.5, 52.0, 42.5, 37.5, 21.9.

IR (ν_{max}, cm^{-1}) 3066 (w), 3061 (w), 3057 (w), 3022 (w), 3018 (w), 3013 (w), 3004 (w), 2997 (w), 1642 (m), 1622 (s), 1601 (m).

HRMS (nanochip-ESI/LTQ-Orbitrap) m/z: $[M + H]^+$ Calcd for $C_{30}H_{29}INO_7S^+$ 674.0704; Found 674.0733.

Methyl (Z)-3-(1-(*N*-(4-methoxyphenyl)methylsulfonamido)-2-(3-oxo-1λ^3-benzo[*d*][1,2]iodaoxol-1(3*H*)-yl)vinyl)bicyclo[1.1.1]pentane-1-carboxylate (29e)

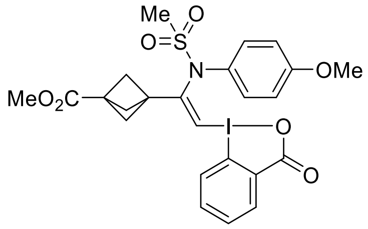

Starting from **EBX.2** (119 mg, 0.300 mmol), **29e** (103 mg, 0.172 mmol, 57% yield) was obtained, as a brown solid. Purified in deactivated silica; decomposition upon silica gel.

R$_f$ = 0.23 (5% MeOH/ DCM).

m.p. 147.0–153.7 °C (decomp).

^{1}H NMR (400 MHz, $CDCl_3$) δ 8.51 – 8.40 (m, 1H, Ar*H*), 7.75 – 7.61 (m, 2H, Ar*H*), 7.49 – 7.40 (m, 1H, Ar*H*), 7.34 – 7.27 (m, 2H, Ar*H*), 6.91 (s, 1H, vinyl*H*), 6.89 – 6.77 (m, 2H, Ar*H*), 3.79 (s, 3H, COOCH_3), 3.65 (s, 3H ArOCH_3), 3.17 (s, 3H, SO_2CH_3), 2.16 (s, 6H, H_{BCP}).

^{13}C NMR (101 MHz, $CDCl_3$) δ 169.2, 166.8, 159.7, 152.1, 134.1, 133.9, 133.4, 131.2, 130.4, 127.2, 125.9, 115.5, 114.9, 107.8, 55.7, 54.3, 52.1, 42.5, 40.3, 37.5.

One carbon not resolved

IR (ν_{max}, cm^{-1}) 3004 (w), 2957 (w), 2928 (w), 2882 (w), 1731 (m), 1606 (m), 1519 (s).

HRMS (ESI/QTOF) m/z: $[M + H]^+$ Calcd for $C_{24}H_{25}INO_7S^+$ 598.0391; Found 598.0405.

Methyl (*Z*)-3-(1-((*N*-(4-methoxyphenyl)-4-nitrophenyl)sulfonamido)-2-(3-oxo-1λ^3-benzo[*d*][1,2]iodaoxol-1(3*H*)-yl)vinyl)bicyclo[1.1.1]pentane-1-carboxylate (29f)

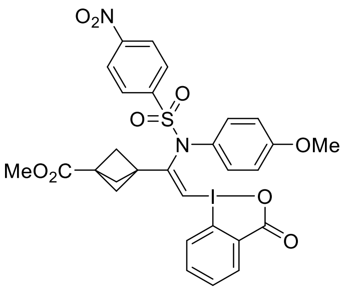

Starting from **EBX.2** (119 mg, 0.300 mmol), **29f** (117 mg, 0.166 mmol, 55% yield) was obtained, as a brown solid. Purified in deactivated silica; decomposition upon silica gel.

R$_f$ = 0.21 (5% MeOH/ DCM).

m.p. 149.1–152.4 °C (melt).

^{1}H NMR (400 MHz, $CDCl_3$) δ 8.59 – 8.42 (m, 1H, Ar*H*), 8.42 – 8.31 (m, 2H, Ar*H*), 8.04 – 7.87 (m, 2H, Ar*H*), 7.72 – 7.59 (m, 2H, Ar*H*), 7.40 – 7.28 (m, 1H, Ar*H*), 7.04 – 6.94 (m, 3H, Ar*H* + vinyl*H*), 6.82 – 6.67 (m, 2H, Ar*H*), 3.77 (s, 3H, ArOCH_3), 3.64 (s, 3H, OCH_3), 2.06 (s, 6H, H_{BCP}).

^{13}C NMR (101 MHz, $CDCl_3$) δ 168.9, 166.9, 160.3, 152.6, 150.9, 144.6, 134.0, 133.9, 133.4, 131.3, 129.6, 129.6, 128.9, 125.7, 124.7, 115.3, 115.3, 108.6, 55.7, 54.5, 52.1, 46.1, 42.6, 37.6.

IR (ν_{max}, cm^{-1}) 3108 (w), 3065 (w), 3002 (w), 2925 (w), 1726 (m), 1640 (m), 1623 (m), 1532 (m), 1506 (m).

HRMS (ESI/QTOF) m/z: $[M + H]^+$ Calcd for $C_{29}H_{26}IN_2O_9S^+$ 705.0398; Found 705.0413.

(Z)-*N*-(4-methoxyphenyl)-4-methyl-*N*-(2-(3-oxo-1λ^3-benzo[*d*][1,2]iodaoxol-1(3*H*)-yl)-1-(3-phenylbicyclo[1.1.1]pentan-1-yl)vinyl)benzenesulfonamide (29g)

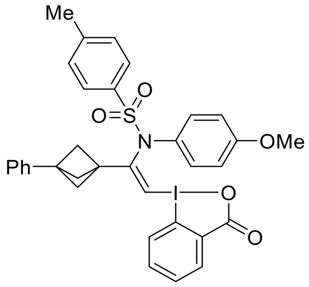

Starting from **EBX.3** (124 mg, 0.300 mmol), **29g** (131 mg, 0.189 mmol, 63% yield) was obtained, as a pale-yellow solid.

R$_f$ = 0.25 (5% MeOH/ DCM).

m.p. 121.0–131.2 °C (melt).

^{1}H NMR (400 MHz, $CDCl_3$) δ 8.48 (dd, *J* = 5.9, 3.3 Hz, 1H, Ar*H*), 7.76 – 7.69 (m, 2H, Ar*H*), 7.69 – 7.62 (m, 2H, Ar*H*), 7.48 – 7.40 (m, 1H, Ar*H*), 7.36 – 7.29 (m, 2H, Ar*H*), 7.30 – 7.24 (m, 2H, Ar*H*), 7.24 – 7.17 (m, 1H, Ar*H*), 7.10 – 7.03 (m, 4H, Ar*H*), 6.94 (s, 1H, vinyl*H*), 6.79 – 6.72 (m, 2H, Ar*H*), 3.75 (s, 3H, OCH_3), 2.44 (s, 3H, CH_3), 2.02 (s, 6H, HBCP).

^{13}C NMR (101 MHz, $CDCl_3$) δ 167.0, 159.6, 154.4, 145.4, 138.8, 136.2, 134.4, 133.6, 133.3, 130.9, 130.9, 130.2, 128.8, 128.4, 128.4, 127.2, 126.0, 125.7, 115.3, 114.8, 106.4, 55.6, 55.2, 41.8, 41.7, 21.8.

IR (ν_{max}, cm^{-1}) 3028 (w), 3022 (w), 3007 (w), 2991 (w), 2985 (w), 2971 (w), 2966 (w), 2956 (w), 2907 (w), 1674 (w), 1641 (s), 1622 (s), 1613 (s), 1607 (s).

HRMS (ESI/QTOF) m/z: $[M + Na]^+$ Calcd for $C_{34}H_{30}INNaO_5S^+$ 714.0782; Found 714.0803.

(*Z*)-*N*-(1-(3-benzylbicyclo[1.1.1]pentan-1-yl)-2-(3-oxo-1λ^3-benzo[*d*][1,2]iodaoxol-1(3*H*)-yl)vinyl)-*N*-(4-methoxyphenyl)-4-methylbenzenesulfonamide (29h)

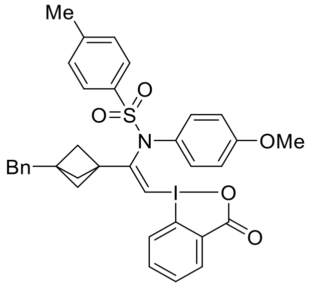

Starting from **EBX.4** (64.2 mg, 0.150 mmol), **29h** (65.0 mg, 92.1 µmol, 61% yield) was obtained, as a light-yellow solid.
R$_f$ = 0.22 (5% MeOH/ DCM).
m.p. 90.7–101.5 °C (melt).
^{1}H NMR (400 MHz, CDCl$_3$) δ 8.48 (dd, *J* = 7.4, 1.9 Hz, 1H, Ar*H*), 7.70 – 7.54 (m, 4H, Ar*H*), 7.35 – 7.27 (m, 5H, Ar*H*), 7.24 – 7.17 (m, 1H, Ar*H*), 7.03 – 6.96 (m, 4H, Ar*H*), 6.74 (s, 1H, vinyl*H*), 6.73 – 6.68 (m, 2H, Ar*H*), 3.75 (s, 3H, OC*H$_3$*), 2.69 (s, 2H, C*H$_2$*Ph), 2.43 (s, 3H, C*H$_3$*), 1.61 (s, 6H, H_{BCP}).
^{13}C NMR (101 MHz, CDCl$_3$) δ 167.0, 159.6, 154.9, 145.3, 138.4, 136.1, 134.3, 133.6, 133.3, 130.9, 130.9, 130.1, 129.0, 128.8, 128.5, 128.4, 126.3, 125.7, 115.2, 114.8, 105.9, 55.6, 53.4, 43.3, 40.2, 38.3, 21.8.
IR (ν_{max}, cm^{-1}) 3057 (w), 3026 (m), 2964 (m), 2925 (m), 2909 (m), 2871 (m), 2841 (w), 1637 (s), 1621 (s).
HRMS (APCI/QTOF) m/z: [M + H]$^+$ Calcd for $C_{35}H_{33}INO_5S^+$ 706.1119; Found 706.1151.

(*Z*)-1-(2-(3-(phenylsulfonyl)bicyclo[1.1.1]pentan-1-yl)-2-(p-tolyloxy)vinyl)-1λ^3-benzo[*d*][1,2]iodaoxol-3(1*H*)-one (30a)

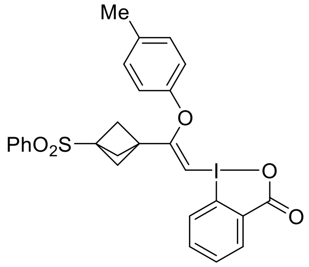

Starting from **EBX.1** (144 mg, 0.300 mmol), **30a** (130 mg, 0.222 mmol, 74% yield) was obtained, as a dark brown solid.
R$_f$ = 0.38 (10% MeOH/ DCM).
m.p. 119.9–130.4 °C (melt).
^{1}H NMR (400 MHz, CDCl$_3$) δ 8.39 – 8.32 (m, 1H, H_{Ar}), 7.82 – 7.75 (m, 2H, H_{Ar}), 7.71 – 7.64 (m, 1H, H_{Ar}), 7.61 – 7.52 (m, 4H, H_{Ar}), 7.44 – 7.38 (m, 1H, H_{Ar}), 7.08 – 7.02 (m, 2H, H_{Ar}), 6.77 – 6.71 (m, 2H, H_{Ar}), 6.05 (s, 1H, H_{vinyl}), 2.28 (s, 3H, H_{Me}), 2.13 (s, 6H, H_{BCP}).
^{13}C NMR (101 MHz, CDCl$_3$) δ 166.7, 164.1, 152.3, 136.2, 135.6, 134.3, 133.6, 133.6, 133.0, 130.9, 130.8, 129.5, 128.7, 125.4, 118.6, 114.2, 53.5, 51.8, 38.5, 20.9.
One aromatic carbon not resolved.
IR (ν_{max}, cm^{-1}) 3076 (w), 3073 (w), 3066 (w), 3060 (w), 3051 (w), 3016 (w), 3008 (w), 2921 (w), 1657 (w), 1647 (m), 1642 (m), 1618 (s), 1601 (s).

HRMS (nanochip-ESI/LTQ-Orbitrap) m/z: [M + H]$^+$ Calcd for $C_{34}H_{31}INO_7S_2^+$ 756.0581; Found 756.0573.

(*Z*)-1-(2-(perfluorophenoxy)-2-(3-(phenylsulfonyl)bicyclo[1.1.1]pentan-1-yl)vinyl)-1λ^3-benzo[*d*][1,2]iodaoxol-3(1*H*)-one (30b)

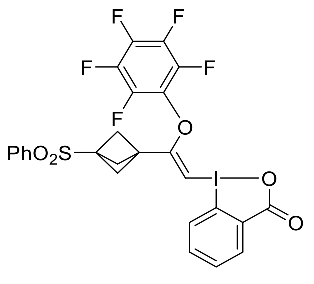

Starting from **EBX.1** (144 mg, 0.300 mmol), **30b** (153 mg, 0.231 mmol, 77% yield) was obtained, as a light brown amorphous solid.
R$_f$ = 0.21 (5% MeOH/ DCM).
^{1}H NMR (400 MHz, $CDCl_3$) δ 8.42 – 8.34 (m, 1H, Ar*H*), 7.88 – 7.80 (m, 2H, Ar*H*), 7.75 – 7.68 (m, 1H, Ar*H*), 7.66 – 7.54 (m, 4H, Ar*H*), 7.39 (dd, *J* = 7.7, 1.4 Hz, 1H, Ar*H*), 6.15 – 6.02 (m, 1H, vinyl*H*), 2.22 (s, 6H, H_{BCP}).
^{13}C NMR (101 MHz, $CDCl_3$) δ 166.3, 162.6, 135.7, 134.5, 133.8, 133.1, 131.2, 131.1, 129.6, 128.7, 114.0, 86.1, 53.2, 51.3, 37.9.
6 aromatic carbons are not resolved due to coupling with F.
^{19}F NMR (376 MHz, $CDCl_3$) δ -154.7 (d, *J* = 17.5 Hz), -155.6 (t, *J* = 21.9 Hz), -159.4 (dd, *J* = 21.8, 17.2 Hz).
IR ($ν_{max}$, cm^{-1}) 2976 (w), 2970 (w), 2931 (w), 2921 (w), 1658 (w), 1648 (m), 1641 (m), 1621 (m), 1618 (m), 1604 (s).
HRMS (ESI/QTOF) m/z: [M + H]$^+$ Calcd for $C_{26}H_{17}F_5IO_5S^+$ 662.9756; Found 662.9766.

(*Z*)-1-(2-(Tyrosine)-2-(3-(phenylsulfonyl)bicyclo[1.1.1]pentan-1-yl)vinyl)-1λ^3-benzo[*d*][1,2]iodaoxol-3(1*H*)-one (30c)

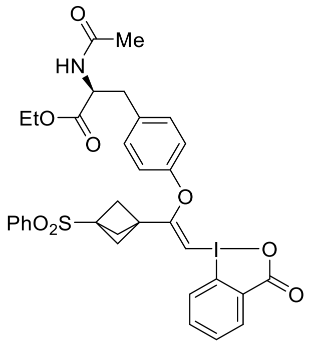

Starting from **EBX.2** (144 mg, 0.300 mmol), **30c** (185 mg, 0.254 mmol, 85% yield) was obtained, as a brown solid.
R$_f$ = 0.20 (10% MeOH/ DCM).
m.p. 140.7–148.2 °C (melt).
^{1}H NMR (400 MHz, $CDCl_3$) δ 8.39 – 8.28 (m, 1H, N*H*), 7.83 – 7.75 (m, 2H, Ar*H*), 7.72 – 7.64 (m, 1H, Ar*H*), 7.63 – 7.52 (m, 4H, Ar*H*), 7.44 – 7.37 (m, 1H, Ar*H*), 7.05 (d, *J* = 8.5 Hz, 2H, Ar*H*), 6.81 (d, *J* = 8.5 Hz, 2H, Ar*H*), 6.39 (d, *J* = 7.5 Hz, 1H, Ar*H*), 6.04 (s, 1H, vinyl*H*), 4.76 (dt, *J* = 7.5, 6.0 Hz, 1H, C=OC*H*NH), 4.18 – 4.05 (m, 2H, OC*H$_2$*CH$_3$), 3.15 (dd, *J* = 14.0, 5.9 Hz, 1H, CHC*H$_2$*Ar), 3.03 (dd, *J* =

14.0, 6.0 Hz, 1H, CHCH_2Ar), 2.16 (s, 6H, HBCP), 1.97 (s, 3H, C=OCH_3), 1.22 (t, *J* = 7.1 Hz, 3H, OCH_2CH_3).
^{13}C NMR (101 MHz, $CDCl_3$) δ 171.4, 170.1, 166.7, 164.0, 153.6, 136.2, 134.3, 133.7, 133.6, 133.1, 131.4, 131.0, 129.5, 128.7, 125.4, 119.0, 114.4, 85.1, 61.8, 53.4, 53.3, 51.7, 38.8, 37.1, 23.2, 14.3.
One carbon not resolved.
IR (ν_{max}, cm^{-1}) 3289 (w), 3063 (w), 2932 (w), 1739 (m), 1607 (s), 1505 (m).
HRMS (APCI/QTOF) m/z: $[M + Na]^+$ Calcd for $C_{33}H_{32}INNaO_8S^+$ 752.0786; Found 752.0809.

Methyl (Z)-3-(2-(3-oxo-1λ^3-benzo[*d*][1,2]iodaoxol-1(3*H*)-yl)-1-(*p*-tolyloxy)vinyl)bicyclo[1.1.1]pentane-1-carboxylate (30d)

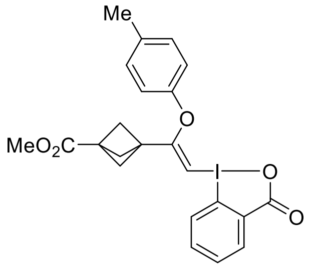

Starting from **EBX.2** (119 mg, 0.300 mmol), **30d** (98.0 mg, 0.194 mmol, 65% yield) was obtained, as a brown solid. The reaction was conducted in DCM (0.008 M).
1 mmol scale reaction: Starting from **EBX.2** (396 mg, 1.00 mmol), **XX** (381 mg, 0.756 mmol, 76% yield) was obtained. The reaction was conducted in DCM (0.08 M).
R_f = 0.25 (5% MeOH/ DCM).
m.p. 102.2–108.6 °C (decomp).
^{1}H NMR (400 MHz, $CDCl_3$) δ 8.48 – 8.39 (m, 1H, Ar*H*), 7.65 (m, 2H, Ar*H*), 7.56 – 7.48 (m, 1H, Ar*H*), 7.09 (d, *J* = 8.6 Hz, 2H, Ar*H*), 6.81 (d, *J* = 8.5 Hz, 2H, Ar*H*), 5.94 (s, 1H, vinyl*H*), 3.64 (s, 3H, OCH_3), 2.31 (s, 3H, ArCH_3), 2.15 (s, 6H, H_{BCP}).
^{13}C NMR (101 MHz, $CDCl_3$) δ 169.0, 166.7, 166.4, 152.5, 135.5, 133.7, 133.6, 133.2, 131.0, 130.6, 125.2, 119.0, 114.1, 83.2, 54.6, 52.1, 39.4, 38.5, 20.9.
IR (ν_{max}, cm^{-1}) 3001 (w), 2992 (w), 2988 (w), 2972 (w), 2957 (w), 2912 (w), 2880 (w), 1744 (m), 1720 (m), 1648 (w), 1637 (m), 1618 (s), 1600 (s).
HRMS (ESI/QTOF) m/z: $[M + H]^+$ Calcd for $C_{23}H_{22}IO_5^+$ 505.0506; Found 505.0522.

Methyl (*Z*)-3-(2-(3-oxo-1λ^3-benzo[*d*][1,2]iodaoxol-1(3*H*)-yl)-1-(perfluorophenoxy)vinyl)bicyclo[1.1.1]pentane-1-carboxylate (30e)

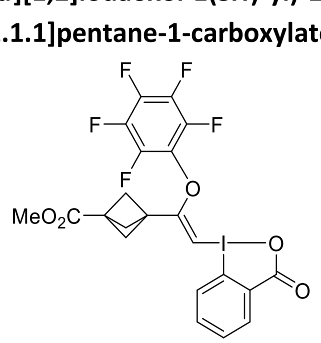

Starting from **EBX.2** (119 mg, 0.300 mmol), **(30e)** (104 mg, 0.179 mmol, 60% yield) was obtained, as a brown solid. The reaction was conducted in DCM (0.008 M).
R_f = 0.22 (5% MeOH/ DCM).

m.p. 109.7–122.2 °C (melt).

^{1}H NMR (400 MHz, $CDCl_3$) δ 8.46 – 8.39 (m, 1H, Ar*H*), 7.71 – 7.61 (m, 2H, Ar*H*), 7.54 – 7.42 (m, 1H, Ar*H*), 6.07 (d, *J* = 3.1 Hz, 1H, vinyl*H*), 3.68 (s, 3H, OC*H₃*), 2.22 (s, 6H, H_{BCP}).

^{13}C NMR (101 MHz, $CDCl_3$) δ 168.4, 166.7, 164.6, 133.9, 133.3, 133.2, 131.2, 125.5, 114.0, 84.1, 54.4, 52.3, 38.7, 38.1.

C-F carbons not resolved.

^{19}F NMR (376 MHz, $CDCl_3$) δ -154.6 (d, *J* = 19.4 Hz), -156.4 (t, *J* = 23.1 Hz), -160.2 (dd, *J* = 24.8, 18.9 Hz).

IR (ν_{max}, cm^{-1}) 3017 (w), 3012 (w), 2994 (w), 2976 (w), 2950 (w), 2930 (w), 2917 (w), 1752 (w), 1748 (w), 1720 (w), 1648 (w), 1642 (w), 1621 (m), 1601 (m).

HRMS (ESI/QTOF) m/z: $[M + H]^+$ Calcd for $C_{22}H_{15}F_5IO_5^+$ 580.9879; Found 580.9907.

Methyl (*Z*)-3-(1-(3,5-dibromophenoxy)-2-(3-oxo-1λ³-benzo[*d*][1,2]iodaoxol-1(3*H*)-yl)vinyl)bicyclo[1.1.1]pentane-1-carboxylate (30f)

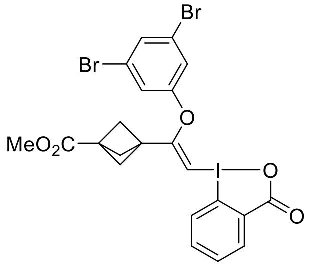

Starting from **EBX.2** (119 mg, 0.300 mmol), **30f** (137 mg, 0.211 mmol, 70% yield) was obtained, as a brown amorphous solid.

R_f = 0.43 (10% MeOH/ DCM).

^{1}H NMR (400 MHz, $CDCl_3$/ MeOD) δ 8.38 – 8.26 (m, 1H, Ar*H*), 7.67 – 7.57 (m, 2H, Ar*H*), 7.52 – 7.43 (m, 1H, Ar*H*), 7.44 – 7.37 (m, 1H, Ar*H*), 7.11 – 7.01 (m, 2H, Ar*H*), 6.26 (s, 1H, vinyl*H*), 3.63 (s, 3H, OC*H₃*), 2.20 (s, 6H, H_{BCP}).

^{13}C NMR (101 MHz, $CDCl_3$/ MeOD) δ 164.7, 155.5, 134.5, 133.2, 131.1, 131.0, 126.2, 123.6, 120.8, 114.6, 87.3, 54.6, 52.1, 38.9, 38.5.

One C=O carbon not resolved.

IR (ν_{max}, cm^{-1}) 3046 (w), 2995 (w), 2962 (w), 2922 (w), 2853 (w), 1734 (m), 1606 (w), 1595 (w), 1564 (m).

HRMS (ESI/QTOF) m/z: $[M + H]^+$ Calcd for $C_{22}H_{18}Br_2IO_5^+$ 646.8560; Found 646.8560.

Methyl (*Z*)-3-(1-(4-(2-tyrosine)-2-(3-oxo-1λ³-benzo[*d*][1,2]iodaoxol-1(3*H*)-yl)vinyl)bicyclo[1.1.1]pentane-1-carboxylate (30g)

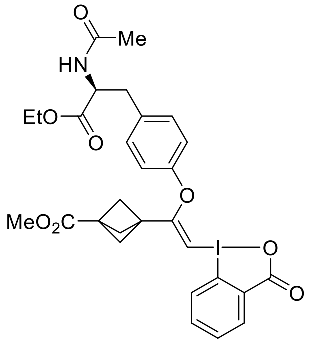

Starting from **EBX.2** (119 mg, 0.300 mmol), **30g** (135 mg, 0.209 mmol, 70% yield) was obtained, as a brown solid.

R$_f$ = 0.29 (10% MeOH/ DCM).

m.p. 102.4–115.6 °C (melt).

^{1}H NMR (400 MHz, $CDCl_3$) δ 8.44 – 8.36 (m, 1H, N*H*), 7.69 – 7.60 (m, 2H, Ar*H*), 7.52 – 7.44 (m, 1H, Ar*H*), 7.08 (d, *J* = 8.5 Hz, 2H, Ar*H*), 6.85 (d, *J* = 8.5 Hz, 2H, Ar*H*), 6.33 (d, *J* = 7.6 Hz, 1H, Ar*H*), 5.98 (s, 1H, vinyl*H*), 4.78 (dd, *J* = 7.7, 6.0 Hz, 1H, CN*H*CH$_2$), 4.22 – 4.05 (m, 2H, OCH_2CH$_3$), 3.64 (s, 3H, OCH_3), 3.15 (dd, *J* = 14.0, 6.0 Hz, 1H, CNHCH_2), 3.05 (dd, *J* = 14.0, 6.0 Hz, 1H, CNHCH_2), 2.17 (s, 6H, H_{BCP}), 1.96 (s, 3H, COCH_3), 1.22 (t, *J* = 7.2 Hz, 3H, OCH$_2$CH_3).

^{13}C NMR (101 MHz, $CDCl_3$) δ 171.5, 170.0, 169.0, 166.7, 166.2, 153.7, 134.0, 133.7, 133.7, 133.2, 131.2, 131.0, 125.3, 119.3, 114.2, 83.2, 61.7, 54.5, 53.4, 52.0, 39.5, 38.3, 37.2, 23.2, 14.3.

IR ($ν_{max}$, cm^{-1}) 3275 (w), 3065 (w), 2995 (w), 2925 (w), 1733 (s), 1605 (s), 1504 (m).

HRMS (ESI/QTOF) m/z: $[M + H]^+$ Calcd for $C_{29}H_{31}INO_8^+$ 648.1089; Found 648.1097.

(Z)-1-(2-(3-phenylbicyclo[1.1.1]pentan-1-yl)-2-(p-tolyloxy)vinyl)-1λ^3-benzo[*d*][1,2]iodaoxol-3(1*H*)-one (30h)

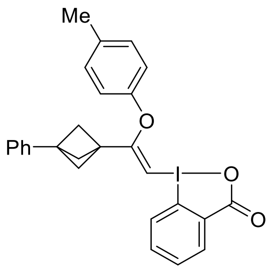

Starting from **EBX.3** (124 mg, 0.300 mmol), **30h** (128 mg, 0.245 mmol, 82% yield) was obtained, as a yellow amorphous solid.

R$_f$ = 0.23 (5% MeOH/ DCM).

^{1}H NMR (400 MHz, $CDCl_3$) δ 8.49 – 8.42 (m, 1H, Ar*H*), 7.68 – 7.61 (m, 2H, Ar*H*), 7.60 – 7.53 (m, 1H, Ar*H*), 7.32 – 7.26 (m, 2H, Ar*H*), 7.25 – 7.19 (m, 1H, Ar*H*), 7.15 – 7.07 (m, 4H, Ar*H*), 6.89 – 6.82 (m, 2H, Ar*H*), 6.00 (s, 1H, vinyl*H*), 2.31 (s, 3H, ArCH_3), 2.17 (s, 6H, H_{BCP}).

^{13}C NMR (101 MHz, $CDCl_3$) δ 167.8, 166.6, 152.7, 138.9, 135.2, 133.9, 133.5, 133.1, 130.8, 130.5, 128.4, 127.2, 126.0, 125.2, 119.0, 114.1, 82.5, 55.3, 42.8, 38.7, 20.9.

IR ($ν_{max}$, cm^{-1}) 3057 (w), 3029 (w), 2981 (w), 2912 (w), 2873 (w), 1602 (s), 1558 (m), 1504 (s).

HRMS (ESI/QTOF) m/z: $[M + H]^+$ Calcd for $C_{27}H_{24}IO_3^+$ 523.0765; Found 523.0775.

(Z)-1-(2-(3-benzylbicyclo[1.1.1]pentan-1-yl)-2-(p-tolyloxy)vinyl)-1λ^3-benzo[*d*][1,2]iodaoxol-3(1*H*)-one (30i)

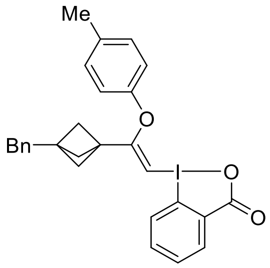

Starting from **EBX.4** (64.2 mg, 0.150 mmol), **30i** (62.1 mg, 0.119 mmol, 79% yield) was obtained, as a light orange solid.

R_f = 0.23 (5% MeOH/ DCM).
m.p. 73.3–87.4 °C (melt).
^{1}H NMR (400 MHz, $CDCl_3$) δ 8.48 – 8.38 (m, 1H, Ar*H*), 7.66 – 7.56 (m, 2H, Ar*H*), 7.50 – 7.42 (m, 1H, Ar*H*), 7.31 – 7.26 (m, 2H, Ar*H*), 7.25 – 7.16 (m, 1H, Ar*H*), 7.12 – 6.99 (m, 4H, Ar*H*), 6.79 – 6.73 (m, 2H, Ar*H*), 5.82 (s, 1H, vinyl*H*), 2.72 (s, 2H, PhC*H₂*), 2.31 (s, 3H, ArC*H₃*), 1.73 (s, 6H, H_{BCP}).
^{13}C NMR (101 MHz, $CDCl_3$) δ 168.0, 166.6, 152.8, 138.4, 134.9, 133.8, 133.4, 133.2, 130.8, 130.4, 129.0, 128.5, 126.4, 125.0, 118.8, 114.1, 82.2, 53.4, 41.4, 40.1, 38.4, 20.9.
IR (ν_{max}, cm^{-1}) 2983 (m), 2905 (m), 2877 (m), 1630 (s), 1605 (s), 1584 (m), 1556 (m).
HRMS (APCI/QTOF) m/z: $[M + H]^+$ Calcd for $C_{28}H_{26}IO_3^+$ 537.0921; Found 537.0942.

Natural product entries

Methyl (Z)-3-(1-sulfaphenazole)-2-(3-oxo-1λ^3-benzo[*d*][1,2]iodaoxol-1(3*H*)-yl)vinyl)bicyclo[1.1.1]pentane-1-carboxylate (31)

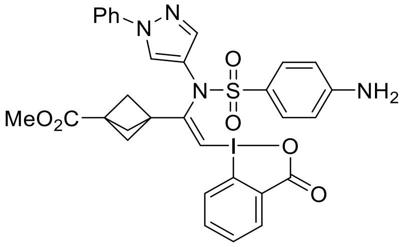

Starting from **EBX.2** (119 mg, 0.300 mmol) and sulfaphenazole (94.3 mg, 0.300 mmol), **31** (74.3 mg, 10.5 µmol, 35% yield) was obtained, as a light amorphous brown solid.
R_f = 0.19 (10% MeOH/ DCM).
^{1}H NMR (400 MHz, $CDCl_3$/ MeOD) δ 8.27 – 8.16 (m, 1H, Ar*H*), 8.10 – 8.01 (m, 1H, Ar*H*), 7.60 (t, *J* = 7.3 Hz, 1H, Ar*H*), 7.46 (td, *J* = 7.8, 1.7 Hz, 1H, Ar*H*), 7.40 – 7.35 (m, 2H, Ar*H*), 7.15 – 7.02 (m, 3H, Ar*H*), 6.98 – 6.88 (m, 4H, Ar*H*), 6.49 (d, *J* = 3.8 Hz, 1H, vinyl*H*), 6.44 – 6.31 (m, 2H, Ar*H*), 3.25 (s, 3H, OCH_3), 2.07 (d, *J* = 11.9 Hz, 6H, H_{BCP}).
^{13}C NMR (101 MHz, $CDCl_3$/ MeOD) δ 168.9, 168.6, 160.8, 151.6, 150.4, 145.2, 134.1, 133.8, 132.8, 132.6, 131.0, 130.7, 129.1, 128.9, 127.9, 127.0, 126.0, 115.8, 113.8, 106.6, 100.6, 54.1, 52.1, 41.2, 36.9.
IR (ν_{max}, cm^{-1}) 2990 (m), 2957 (s), 2952 (s), 2947 (s), 2925 (s), 2918 (s), 2910 (s), 2900 (s), 2856 (m), 1732 (m), 1619 (m), 1598 (s), 1539 (s).
HRMS (ESI/QTOF) m/z: $[M + Na]^+$ Calcd for $C_{31}H_{27}IN_4NaO_6S^+$ 733.0588; Found 733.0595.

1-((Z)-2-(3-phenylbicyclo[1.1.1]pentan-1-yl)-2-α-tocopherol-1λ^3-benzo[*d*][1,2]iodaoxol-3(1*H*)-one (32)

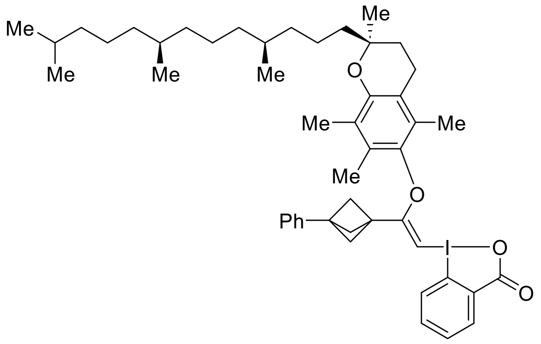

Starting from **EBX.3** (62.1 mg, 0.150 mmol) and α-tocopherol (64.6 mg, 68.0 µL, 0.150 mmol), **32** (80.1 mg, 94.8 µmol, 63% yield) was obtained, as a yellow sticky solid.
R$_f$ = 0.29 (10% MeOH/ DCM).
m.p. 78.6–91.4 °C (melt).
2 isomers were observed (1: 1.12 ratio) from the NMR. ^{1}H-^{1}H NOESY experiment potentially suggests E/Z isomers. High temperature NMR was also conducted, but no significant difference was observed. See Section 8 (SI 79) for further explanation on ^{1}H-^{1}H NOESY analysis. We highlight that this compound was the only example where we observed 2 isomers of VBX following the general procedure. The NMR in $CDCl_3$ didn't show significant difference in the chemical shifts between two isomers other than vinyl proton, so they were integrated together.
^{1}H NMR (400 MHz, $CDCl_3$) δ 8.52 – 8.41 (m, 1H, Ar*H*), 7.63 (dtd, *J* = 7.0, 3.9, 1.6 Hz, 3H, Ar*H*), 7.32 – 7.26 (m, 2H, Ar*H*), 7.25 – 7.18 (m, 1H, Ar*H*), 7.10 (td, *J* = 7.9, 1.3 Hz, 2H, Ar*H*), 5.58 (d, *J* = 14.6 Hz, 1H, vinyl*H*), 2.61 – 2.50 (m, 2H, C*H$_2$*), 2.10 (s, 3H, ArC*H$_3$*), 2.06 (d, *J* = 4.3 Hz, 6H, H_{BCP}), 2.00 (s, 3H, ArC*H$_3$*), 1.96 (s, 3H, ArC*H$_3$*), 1.86 – 1.74 (m, 2H, C*H$_2$*), 1.58 – 1.45 (m, 4H, C*H*), 1.43 – 1.31 (m, 4H, C*H$_2$*), 1.30 – 1.17 (m, 9H, C*H* + C*H$_2$* + C*H$_3$*), 1.16 – 0.97 (m, 7H, C*H* + C*H$_2$* + C*H$_3$*), 0.88 – 0.77 (m, 12H, C*H$_3$*).
^{13}C NMR (101 MHz, $CDCl_3$) δ 169.6, 169.1, 166.7, 150.1, 149.9, 143.8, 143.5, 138.8, 138.8, 134.2, 134.1, 133.2, 133.2, 133.1, 130.8, 130.7, 128.5, 127.6, 127.6, 127.3, 126.0, 125.9, 125.8, 125.1, 124.0, 123.7, 118.4, 118.1, 114.0, 113.9, 75.6, 75.6, 75.6, 72.9, 54.9, 54.8, 42.1, 42.0, 40.5, 40.4, 39.5, 39.2, 39.2, 39.0, 38.7, 37.8, 37.7, 37.5, 37.5, 37.4, 37.4, 37.3, 32.9, 32.9, 32.9, 32.8, 31.3, 28.1, 24.9, 24.6, 24.6, 24.2, 23.2, 23.1, 22.9, 22.8, 21.2, 21.0, 20.8, 20.7, 19.9, 19.8, 19.8, 19.8, 19.7, 19.7, 19.7, 13.4, 13.3, 12.5, 12.4, 11.9.
IR (ν_{max}, cm^{-1}) 3062 (w), 3028 (w), 2950 (s), 2926 (s), 2867 (m), 2849 (m), 1637 (m), 1603 (s), 1560 (m).
HRMS (ESI/QTOF) m/z: [M + H]$^+$ Calcd for $C_{49}H_{66}IO_4^+$ 845.4000; Found 845.4016.

1-((*Z*)-2-Estradiol)-2-(3-(phenylsulfonyl)bicyclo[1.1.1]pentan-1-yl)vinyl)-1λ^3-benzo[*d*][1,2]iodaoxol-3(1*H*)-one (33)

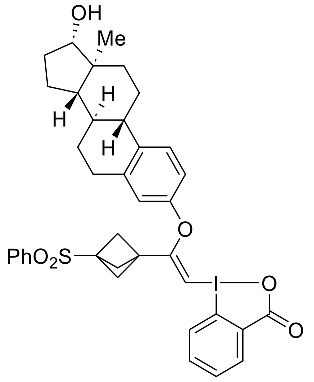

Starting from **EBX.1** (144 mg, 0.30 mmol) and estradiol (81.7 mg, 0.300 mmol), **33** (178 mg, 0.237 mmol, 79% yield) was obtained as a brown amorphous solid.
R$_f$ = 0.37 (10% MeOH/ DCM).
^{1}H NMR (400 MHz, $CDCl_3$) δ 8.42 – 8.31 (m, 1H, Ar*H*), 7.84 – 7.76 (m, 2H, Ar*H*), 7.72 – 7.64 (m, 1H, Ar*H*), 7.63 – 7.52 (m, 4H, Ar*H*), 7.45 – 7.36 (m, 1H, Ar*H*), 7.15 (d, *J* = 8.5 Hz, 1H, Ar*H*), 6.65 – 6.57 (m, 1H, Ar*H*), 6.57 – 6.49 (m, 1H, Ar*H*), 6.03 (s, 1H, vinyl*H*), 3.72 (t, *J* = 8.5 Hz, 1H, C*H*OH), 2.79 – 2.62 (m, 2H, C*H$_2$*), 2.27 – 2.20 (m, 1H, C*H*), 2.18 (s, 6H, H_{BCP}), 2.15 – 2.02 (m, 2H, C*H$_2$*), 1.99

– 1.91 (m, 1H, C*H*), 1.87 – 1.78 (m, 1H, C*H*), 1.72 – 1.61 (m, 1H, C*H*), 1.53 – 1.42 (m, 2H, CH_2), 1.41 – 1.22 (m, 4H, CH_2), 1.21 – 1.05 (m, 1H, C*H*), 0.79 (s, 3H, CH_3).

^{13}C NMR (101 MHz, $CDCl_3$) δ 166.6, 164.2, 152.3, 139.4, 138.1, 136.3, 134.3, 133.6, 133.1, 130.9, 129.5, 128.7, 127.2, 125.3, 118.4, 115.4, 114.3, 85.5, 81.9, 53.4, 51.8, 50.1, 44.0, 43.3, 38.7, 38.5, 36.7, 30.7, 29.6, 27.0, 26.3, 23.2, 11.2.

IR (ν_{max}, cm^{-1}) 3065 (w), 2925 (m), 2867 (m), 1607 (s), 1588 (m), 1561 (m).

HRMS (ESI/QTOF) m/z: $[M + H]^+$ Calcd for $C_{38}H_{40}IO_6S^+$ 751.1585; Found 751.1576.

(Z)-*N*-(3-(phenylsulfonyl)bicyclo[1.1.1]pentan-1-yl)vinyl)-*N*-valsartan-1λ^3-benzo[*d*][1,2]iodaoxol-3(1*H*)-one (34)

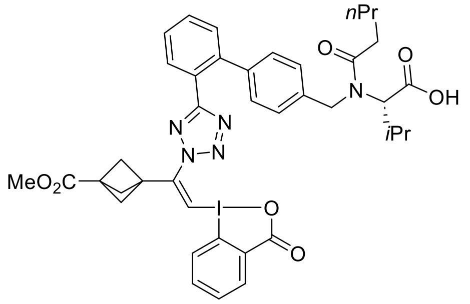

Starting from **EBX.2** (119 mg, 0.300 mmol) and commercially available valsartan (131 mg, 0.300 mmol), **34** was obtained as a brown solid (114 mg, 0.137 mmol, 46% yield).

R_f = 0.41 (10% MeOH/ DCM). Purified in silica gel using solvents with 0.1% AcOH.

m.p. 64.8–78.4 °C (melt).

Mixture of rotamers observed

^{1}H NMR (400 MHz, $CDCl_3$) δ 8.42 – 8.26 (m, 1H, Ar*H*), 8.04 (td, *J* = 7.5, 1.5 Hz, 1H, Ar*H*), 7.65 – 7.53 (m, 3H, Ar*H*), 7.52 – 7.30 (m, 4H, Ar*H*), 7.26 – 7.13 (m, 3H, Ar*H*), 6.65 – 6.42 (m, 1H, vinyl*H*), 4.76 (dd, *J* = 68.3, 16.5 Hz, 1H, ArCH_2N), 4.35 (dd, *J* = 29.4, 16.5 Hz, 1H, ArCH_2N), 4.15 (d, *J* = 11.0 Hz, 1H, NC*H*COOH), 3.75 (d, *J* = 2.1 Hz, 3H, OCH_3), 2.70 – 2.58 (m, 6H, H_{BCP}), 2.50 – 2.10 (m, 2H, NCOC$H_2$$CH_2CH_2CH_3$), 1.73 – 1.62 (m, 1H, NCOCH_2C$H_2$$CH_2CH_3$), 1.59 – 1.45 (m, 1H, NCOCH_2C$H_2$$CH_2CH_3$), 1.42 – 1.33 (m, 1H, NCHC*H*$(CH3)_2$), 1.33 – 1.14 (m, 2H, NCOCH_2CH_2C$H_2$$CH_3$), 1.00 – 0.91 (m, 3H NCO$CH_2CH_2CH_2$CH_3), 0.90 – 0.73 (m, 6H, CH_3).

^{13}C NMR (101 MHz, $CDCl_3$) δ 176.2, 174.9, 174.1, 172.5, 172.5, 170.3, 169.2, 169.1, 169.0, 164.4, 164.2, 143.2, 142.9, 142.6, 141.8, 139.9, 138.2, 138.1, 136.6, 134.5, 134.3, 134.0, 133.9, 133.1, 133.1, 132.2, 131.9, 131.5, 131.1, 131.0, 130.7, 130.5, 129.7, 129.0, 128.2, 127.7, 127.4, 126.8, 126.0, 125.9, 123.8, 123.5, 116.1, 115.9, 95.4, 94.4, 66.2, 54.2, 54.2, 52.2, 52.2, 46.0, 40.4, 40.3, 39.0, 39.0, 34.0, 33.5, 29.8, 28.2, 27.7, 27.3, 27.3, 22.7, 22.4, 20.8, 19.8, 19.5, 18.8, 18.5, 14.1, 13.9.

NMR was also taken at 363 K in DMSO to demonstrate the existence of rotamers.

^{1}H NMR (400 MHz, DMSO) δ 8.18 – 8.11 (m, 1H, Ar*H*), 7.81 (dd, *J* = 7.7, 1.4 Hz, 1H, Ar*H*), 7.76 – 7.65 (m, 3H, Ar*H*), 7.63 (ddd, *J* = 7.1, 3.3, 1.3 Hz, 1H, Ar*H*), 7.55 (td, *J* = 7.6, 1.4 Hz, 1H, Ar*H*), 7.51 – 7.41 (m, 2H, Ar*H* + vinyl*H*), 7.23 – 7.15 (m, 2H, Ar*H*), 7.09 (d, *J* = 8.3 Hz, 2H, Ar*H*), 4.69 – 4.41 (m, 3H, NC*H*COOH), 3.69 (s, 3H, OCH_3), 2.38 (s, 6H, H_{BCP}), 2.28 – 2.09 (m, 2H NCOC$H_2$$CH_2CH_2CH_3$), 1.50 (s, 3H, NCO$CH_2CH_2$$CH_2CH_3$ + NCHC*H*$(CH3)_2$), 1.33 – 1.16 (m, 2H, NCOCH_2CH_2C$H_2$$CH_3$), 1.03 – 0.89 (m, 3H, NCO$CH_2CH_2CH_2$CH_3), 0.87 – 0.70 (m, 6H, CH_3).

^{13}C NMR (101 MHz, DMSO) δ 190.7, 173.2, 173.1, 171.0, 167.8, 167.3, 140.0, 139.4, 136.9, 133.4, 131.7, 130.0, 129.5, 129.2, 128.1, 127.6, 127.0, 126.7, 92.9, 53.1, 52.3, 50.8, 33.3, 32.2, 27.3, 26.5, 21.2, 20.3, 19.4, 18.5, 13.0.

4 aromatic carbons were not resolved.
IR (ν_{max}, cm^{-1}) 2969 (m), 2954 (m), 2949 (m), 2939 (m), 2925 (m), 2918 (m), 1742 (m), 1724 (s), 1706 (m), 1657 (m), 1641 (s), 1621 (s), 1618 (s).
HRMS (ESI/QTOF) m/z: $[M + H]^+$ Calcd for $C_{40}H_{43}IN_5O_7^+$ 832.2202; Found 832.2205.

4.2. General Procedure GP3 for the synthesis of S-VBX

In a round bottomed flask, the correspondent thiol (0.30 mmol, 1.00 equiv.) was dissolved in 37.5 mL of EtOH: DCM (1:1 v/v, 0.08 M). Cs_2CO_3 (9.8 mg, 10 mol%, 10.0 µmol) was added and the mixture stirred vigorously for 5 min. Then the corresponding EBX was added in one portion (0.30 mmol, 1.0 equiv.) and the flask was sealed with a teflon cap. The reaction was left stirring for 1 h. The reaction was stopped, the solvent removed under reduced pressure. The crude was redissolved in DCM and sat. $NaHCO_3$ was added. The layers were separated, and aqueous layer was extracted with DCM (3 x 10 mL). The combined organic layers were washed with sat. $NaHCO_3$ (10 mL), H_2O (10 mL), brine (10 mL), and dried over Na_2SO_4. The solvent was removed under reduced pressure, and the crude purified via column chromatography using DCM:MeOH (20:1) as eluent.

(Z)-1-(2-(benzylthio)-2-(3-(phenylsulfonyl)bicyclo[1.1.1]pentan-1-yl)vinyl)-1λ³-benzo[*d*][1,2]iodaoxol-3(1*H*)-one (35a)

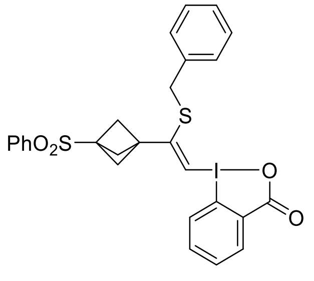

Starting from **EBX.1** (144 mg, 0.300 mmol), **35a** (133 mg, 0.221 mmol, 74% yield) was obtained, as a light-yellow solid.
R_f = 0.35 (10% MeOH/ DCM).
m.p. 75.4–81.2 °C (melt).
^{1}H NMR (400 MHz, $CDCl_3$) δ 8.36 (dd, *J* = 7.5, 1.8 Hz, 1H, Ar*H*), 7.93 – 7.86 (m, 2H, Ar*H*), 7.77 – 7.70 (m, 1H, Ar*H*), 7.66 – 7.60 (m, 2H, Ar*H*), 7.58 (tt, *J* = 7.4, 0.9 Hz, 1H, Ar*H*), 7.50 (m, 1H, Ar*H*), 7.29 – 7.23 (m, 2H, Ar*H*), 7.22 – 7.16 (m, 1H, Ar*H*), 7.14 – 7.08 (m, 3H, Ar*H* + vinyl*H*), 7.04 (dd, *J* = 8.1, 1.0 Hz, 1H, Ar*H*), 3.88 (s, 2H, SCH_2Ar), 2.38 (s, 6H, H_{BCP}).
^{13}C NMR (101 MHz, $CDCl_3$) δ 166.6, 150.9, 136.4, 135.9, 134.4, 133.6, 133.5, 133.2, 130.9, 129.6, 129.5, 129.3, 128.8, 128.7, 125.5, 118.8, 114.4, 53.1, 51.4, 43.8, 39.4.
IR (ν_{max}, cm^{-1}) 3006 (w), 2967 (w), 2923 (w), 1611 (s), 1519 (m).
HRMS (nanochip-ESI/LTQ-Orbitrap) m/z: $[M + H]^+$ Calcd for $C_{27}H_{24}IO_4S_2^+$ 603.0155; Found 603.0182.

Methyl (Z)-3-(1-((2-bromophenyl)thio)-2-(3-oxo-1λ³-benzo[*d*][1,2]iodaoxol-1(3*H*)-yl)vinyl)bicyclo[1.1.1]pentane-1-carboxylate (35b)

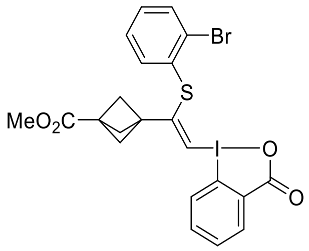

Starting from **EBX.2** (119 mg, 0.300 mmol), **35b** (93.2 mg, 0.159 mmol, 53% yield) was obtained, as a brown amorphous solid.

$\mathbf{R_f}$ = 0.27 (10% MeOH/DCM).

^{1}H NMR (400 MHz, $CDCl_3$) δ 8.45 – 8.38 (m, 1H, Ar*H*), 7.67 – 7.60 (m, 2H, Ar*H*), 7.57 (dd, *J* = 7.8, 1.5 Hz, 1H, Ar*H*), 7.48 (dd, *J* = 7.6, 1.8 Hz, 1H, Ar*H*), 7.45 – 7.39 (m, 1H, Ar*H*), 7.28 – 7.23 (m, 1H, Ar*H*), 7.19 (td, *J* = 7.6, 1.8 Hz, 1H, Ar*H*), 7.02 (s, 1H, vinyl*H*), 3.63 (s, 3H, O*Me*), 2.09 (s, 6H, H_{BCP}).

^{13}C NMR (101 MHz, $CDCl_3$) δ 169.4, 166.8, 156.4, 134.8, 134.0, 133.7, 133.7, 133.2, 131.7, 131.0, 131.0, 128.4, 128.2, 125.8, 114.9, 107.7, 54.5, 52.0, 44.4, 37.1.

IR (ν_{max}, cm^{-1}) 3005 (w), 2992 (w), 2917 (w), 1745 (w), 1729 (w), 1724 (w), 1719 (w), 1648 (w), 1637 (m), 1618 (s), 1600 (s).

HRMS (nanochip-ESI/LTQ-Orbitrap) m/z: $[M + H]^+$ Calcd for $C_{22}H_{19}BrIO_4S^+$ 584.9227; Found 584.9237.

Methyl (S,Z)-3-(cysteine)-2-(3-oxo-1λ^3-benzo[*d*][1,2]iodaoxol-1(3*H*)-yl)vinyl)bicyclo[1.1.1]pentane-1-carboxylate (35c)

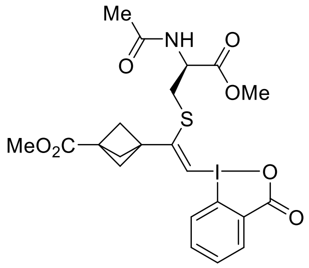

Starting from **EBX.2** (119 mg, 0.300 mmol), **35c** (73.0mg, 0.127 mmol, 42% yield) was obtained, as a brown solid.

$\mathbf{R_f}$ = 0.25 (10% MeOH/DCM).

m.p. 111.2–120.5 °C (melt).

^{1}H NMR (400 MHz, $CDCl_3$) δ 8.43 – 8.38 (m, 1H, N*H*), 7.61 (pd, *J* = 7.2, 1.6 Hz, 3H, Ar*H*), 7.31 – 7.26 (m, 1H, Ar*H*), 6.96 (s, 1H, vinyl*H*), 4.68 (td, *J* = 7.5, 4.1 Hz, 1H, NC*H*C=O), 3.73 (s, 3H, O*Me*), 3.73 (s, 3H, O*Me*), 3.59 (dd, *J* = 14.0, 4.1 Hz, 1H, SC*H₂*), 3.23 (dd, *J* = 14.0, 7.7 Hz, 1H, SC*H₂*), 2.44 (s, 6H, H_{BCP}), 2.04 (s, 3H, C=OC*H₃*).

^{13}C NMR (101 MHz, $CDCl_3$) δ 171.1, 170.7, 169.4, 167.4, 154.8, 133.9, 133.9, 133.3, 131.0, 125.9, 114.7, 111.5, 54.9, 53.2, 52.5, 52.2, 44.2, 37.5, 35.2, 23.3.

IR (ν_{max}, cm^{-1}) 3255 (w), 2989 (w), 2952 (w), 2918 (w), 2880 (w), 1728 (m), 1606 (s), 1552 (m).

HRMS (ESI/QTOF) m/z: $[M + H]^+$ Calcd for $C_{22}H_{25}INO_7S^+$ 574.0391; Found 574.0397.

(Z)-1-(2-(3-phenylbicyclo[1.1.1]pentan-1-yl)-2-(phenylthio)vinyl)-1λ^3-benzo[*d*][1,2]iodaoxol-3(1*H*)-one (35d)

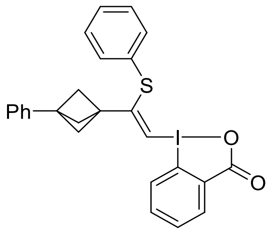

Starting from **EBX.3** (124 mg, 0.300 mmol), **35d** (116 mg, 0.221 mmol, 74% yield) was obtained, as a white solid.
R$_f$ = 0.29 (10% MeOH/DCM).
m.p. 103.8–110.9 °C (melt).
^{1}H NMR (400 MHz, $CDCl_3$) δ 8.51 – 8.41 (m, 1H, Ar*H*), 7.71 – 7.63 (m, 2H, Ar*H*), 7.50 – 7.44 (m, 1H, Ar*H*), 7.43 – 7.37 (m, 2H, Ar*H*), 7.33 (m, 3H, Ar*H*), 7.31 – 7.26 (m, 2H), 7.25 – 7.19 (m, 1H, Ar*H*), 7.14 – 7.09 (m, 2H, Ar*H*), 6.95 (s, 1H, vinyl*H*), 2.12 (s, 6H, H_{BCP}).
^{13}C NMR (101 MHz, $CDCl_3$) δ 166.6, 158.8, 139.1, 133.9, 133.6, 133.4, 132.6, 131.0, 131.0, 129.8, 129.3, 128.4, 127.1, 126.1, 125.5, 114.8, 106.1, 55.2, 43.9, 41.4.
IR (ν_{max}, cm^{-1}) 3059 (m), 3026 (m), 2977 (m), 2908 (m), 2871 (m), 1599 (s), 1556 (m).
HRMS (ESI/QTOF) m/z: [M + H]$^+$ Calcd for $C_{26}H_{22}IO_2S^+$ 525.0380; Found 525.0384.

(Z)-1-(2-(3-benzylbicyclo[1.1.1]pentan-1-yl)-2-(phenylthio)vinyl)-1λ^3-benzo[*d*][1,2]iodaoxol-3(1*H*)-one (35e)

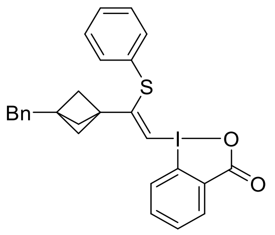

Starting from **EBX.4** (64.2 mg, 0.150 mmol), **35e** (56.6 mg, 0.105 mmol, 70% yield) was obtained, as a light-yellow solid.
R$_f$ = 0.31 (10% MeOH/DCM).
m.p. 74.4–85.9 °C (melt).
^{1}H NMR (400 MHz, $CDCl_3$) δ 8.54 – 8.41 (m, 1H, Ar*H*), 7.72 – 7.56 (m, 2H, Ar*H*), 7.38 – 7.34 (m, 1H, Ar*H*), 7.33 – 7.26 (m, 7H, Ar*H*), 7.24 – 7.16 (m, 1H, Ar*H*), 7.05 – 6.98 (m, 2H, Ar*H*), 6.78 (s, 1H, vinyl*H*), 2.73 (s, 2H, PhC*H$_2$*), 1.69 (s, 6H, H_{BCP}).
^{13}C NMR (101 MHz, $CDCl_3$) δ 166.6, 158.9, 138.6, 133.8, 133.6, 133.5, 132.4, 131.2, 131.0, 129.7, 129.1, 129.0, 128.5, 126.3, 125.3, 114.8, 105.8, 53.3, 45.4, 40.0, 38.4.
IR (ν_{max}, cm^{-1}) 2999 (w), 2994 (w), 2991 (w), 2979 (m), 2972 (m), 2962 (w), 2902 (w), 1632 (m), 1618 (s), 1612 (s).
HRMS (ESI/QTOF) m/z: [M + H]$^+$ Calcd for $C_{27}H_{24}IO_2S^+$ 539.0536; Found 539.0542.

5. Further functionalization employing VBXs

5.1. C-C bond formation

General Procedure GP4 for the C-C bond formation of O-VBX reagents

Following a reported procedure,[10] O-VBX (0.10 mmol, 1.0 equiv.), $Pd(PhCN)_2Cl_2$ (1.9 mg, 5.0 µmol, 5 mol%) and commercially available stannane (0.20 mmol, 2.0 equiv.) were added to a flame-dried vial. The sealed vial was purged with N_2 (x 3). Dry DMF (1.0 mL, 0.1 M) was added under a nitrogen atmosphere and the reaction was left stirring at room temperature for 16 hours. Then the reaction was stopped, EtOAc (10 mL) was added and the organic layer was washed with NaCl (3x30 mL). The solvent was removed under reduced pressure and the crude purified via column chromatography.

(Z)-1-(2-phenyl-1-(p-tolyloxy)vinyl)-3-(phenylsulfonyl)bicyclo[1.1.1]pentane (37a)

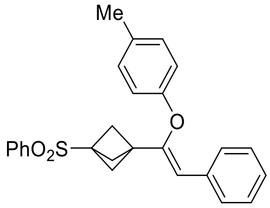

Starting from O-VBX **30a** (59 mg, 0.10 mmol) and commercially available tributyl(phenyl)stannane (65 µL, 0.20 mmol, 2.0 equiv.), **37a** (33 mg, 79 µmol, 79% yield) was obtained as a white amorphous solid.
R_f = 0.33 (15% EtOAc/pentane).
^{1}H NMR (400 MHz, $CDCl_3$) δ 7.84 – 7.79 (m, 2H, Ar*H*), 7.70 – 7.62 (m, 1H, Ar*H*), 7.59 – 7.52 (m, 2H, Ar*H*), 7.50 – 7.45 (m, 2H, Ar*H*), 7.26 – 7.19 (m, 2H, Ar*H*), 7.20 – 7.14 (m, 1H, Ar*H*), 7.08 – 7.02 (m, 2H, Ar*H*), 6.88 – 6.81 (m, 2H, Ar*H*), 5.88 (s, 1H, vinyl*H*), 2.29 (s, 3H, ArC*H*$_3$), 2.09 (s, 6H, H_{BCP}).
^{13}C NMR (101 MHz, $CDCl_3$) δ 153.7, 147.8, 136.8, 133.9, 133.9, 132.5, 130.3, 129.3, 128.7, 128.6, 127.7, 116.9, 116.6, 52.6, 51.4, 40.3, 20.8.
One carbon not resolved.
IR (ν_{max}, cm^{-1}) 3076 (w), 3073 (w), 3066 (w), 3060 (w), 3051 (w), 3016 (w), 3008 (w), 2921 (w), 1657 (w), 1647 (m), 1642 (m), 1618 (s), 1601 (s).
HRMS (APCI/QTOF) m/z: $[M + Na]^+$ Calcd for $C_{27}H_{23}INaO_5S^+$ 609.0203; Found 609.0217.

Methyl (Z)-3-(2-phenyl-1-(p-tolyloxy)vinyl)bicyclo[1.1.1]pentane-1-carboxylate (37b)

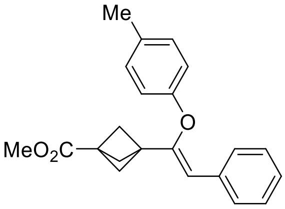

Starting from O-VBX **30d** (50 mg, 0.10 mmol) and commercially available tributyl(phenyl)stannane (65 µL, 0.20 mmol, 2.0 equiv.), **37b** (29 mg, 87 µmol, 87% yield) was obtained as a yellow oil.
R_f = 0.38 (5% EtOAc/pentane).
^{1}H NMR (400 MHz, $CDCl_3$) δ 7.56 – 7.50 (m, 2H, Ar*H*), 7.28 – 7.20 (m, 2H, Ar*H*), 7.19 – 7.13 (m, 1H, Ar*H*), 7.10 – 7.03 (m, 2H, Ar*H*), 6.95 – 6.88 (m, 2H, Ar*H*), 5.92 (s, 1H, vinyl*H*), 3.64 (s, 3H, OC*H*$_3$), 2.29 (s, 3H, ArC*H*$_3$), 2.10 (s, 6H, H_{BCP}).

[10] W. Ding, J. Chai, C. Wang, J. Wu, N. Yoshikai, *J. Am. Chem. Soc.* **2020**, 142, 8619–8624.

^{13}C NMR (101 MHz, $CDCl_3$) δ 170.3, 154.0, 149.7, 134.5, 132.2, 130.2, 128.6, 127.3, 116.9, 115.7, 53.7, 51.8, 41.1, 37.6, 20.8.
One aromatic carbon not resolved.
IR (ν_{max}, cm^{-1}) 3029 (w), 2993 (w), 2920 (w), 2881 (w), 1734 (m), 1652 (w), 1506 (s).
HRMS (Sicrit plasma/LTQ-Orbitrap) m/z: $[M + H]^+$ Calcd for $C_{22}H_{23}O_3^+$ 335.1642; Found 335.1641.

General Procedure GP5 for the C-C bond formation of N-VBX reagents
Following a reported procedure,[11] N-VBX (0.10 mmol, 1.0 equiv.), $Pd(PhCN)_2Cl_2$ (3.8 mg, 10 µmol, 10 mol%) and commercially available stannane (0.20 mmol, 2. 0 equiv.) were added to a flame-dried vial. The sealed vial was purged with N_2 (x 3). Dry DMF (1.0 mL, 0.1 M) was added under nitrogen atmosphere and the reaction was stirred at 60 °C for 16 hours. Then the reaction was stopped, EtOAc (10 mL) was added and the organic layer was washed with NaCl (3x30 mL). The solvent was removed under reduced pressure and the crude purified via column chromatography.

(Z)-N-(4-methoxyphenyl)-4-methyl-N-(2-phenyl-1-(3-(phenylsulfonyl)bicyclo[1.1.1]pentan-1-yl)vinyl)benzenesulfonamide (37c)

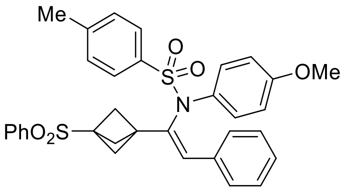

Starting from N-VBX **29a** (151 mg, 0.200 mmol) and commercially available tributyl(phenyl)stannane (131 µL, 0.400 mmol, 2.0 equiv.), **37c** (68.7 mg, 0.117 mmol, 59% yield) was obtained as white solid. it was not possible to separate out from some impurity.
R$_f$ = 0.25 (40% EtOAc/pentane).
^{1}H NMR (400 MHz, $CDCl_3$) δ 7.86 – 7.80 (m, 2H, Ar*H*), 7.69 – 7.62 (m, 1H, Ar*H*), 7.59 – 7.52 (m, 2H, Ar*H*), 7.41 – 7.30 (m, 4H, Ar*H*), 7.25 – 7.19 (m, 3H, Ar*H*), 7.16 – 7.11 (m, 2H, Ar*H*), 7.03 (d, *J* = 8.2 Hz, 2H, Ar*H*), 6.76 – 6.71 (m, 2H, Ar*H*), 6.43 (s, 1H, vinyl*H*), 3.76 (s, 3H, OCH_3), 2.33 (s, 3H, CH_3), 2.16 (s, 6H, H_{BCP}).
^{13}C NMR (101 MHz, $CDCl_3$) δ 157.8, 143.9, 136.9, 136.7, 135.0, 134.0, 133.9, 132.9, 131.8, 129.3, 129.2, 128.9, 128.8, 128.6, 128.5, 128.2, 125.6, 114.5, 55.5, 52.9, 50.9, 42.9, 21.6.
IR (ν_{max}, cm^{-1}) 3063 (w), 3021 (w), 3009 (w), 2960 (m), 2923 (m), 2854 (w), 1601 (w), 1506 (m).
HRMS (ESI/QTOF) m/z: $[M + H]^+$ Calcd for $C_{33}H_{32}NO_5S_2^+$ 586.1716; Found 586.1709.

(Z)-N-(4-methoxyphenyl)-4-methyl-N-(1-(3-(phenylsulfonyl)bicyclo[1.1.1]pentan-1-yl)buta-1,3-dien-1-yl)benzenesulfonamide (37d)

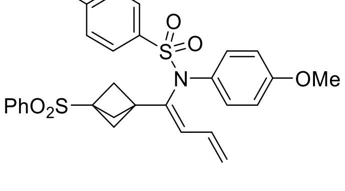

[11] J. Wu, X. Deng, H. Hirao, N. Yoshikai, J. Am. Chem. Soc. 2016, 138, 9105.

Starting from N-VBX **29a** (76 mg, 0.10 mmol) and commercially available tributyl(ethenyl)stannae (59 µL, 0.2 mmol, 2.0 equiv.), **37d** (43 mg, 80 µmol, 80% yield) was obtained as a yellow oil.

R$_f$ = 0.50 (40% EtOAc/pentane).

^{1}H NMR (400 MHz, $CDCl_3$) δ 7.86 – 7.75 (m, 2H, Ar*H*), 7.69 – 7.61 (m, 1H, Ar*H*), 7.58 – 7.50 (m, 4H, Ar*H*), 7.23 – 7.17 (m, 2H, Ar*H*), 7.16 – 7.07 (m, 2H, Ar*H*), 6.80 – 6.70 (m, 2H, Ar*H*), 6.32 (ddd, *J* = 16.8, 10.9, 10.1 Hz, 1H, CHC*H*CH_2), 6.07 (d, *J* = 10.9 Hz, 1H, C*H*CHCH_2), 5.34 (ddd, *J* = 16.8, 1.7, 0.7 Hz, 1H, C=C*H_2*), 5.15 (dd, *J* = 10.1, 1.7 Hz, 1H, C=C*H_2*), 3.79 (s, 3H, OC*H_3*), 2.41 (s, 3H, ArC*H_3*), 2.06 (s, 6H, H_{BCP}).

^{13}C NMR (101 MHz, $CDCl_3$) δ 158.7, 144.0, 137.5, 136.8, 136.3, 133.9, 132.3, 132.3, 131.8, 129.6, 129.3, 128.7, 127.9, 121.8, 114.4, 55.6, 52.5, 51.1, 42.1, 21.7.

One carbon not resolved.

IR (ν_{max}, cm^{-1}) 3067 (w), 3003 (w), 2967 (w), 2922 (w), 2882 (w), 2840 (w), 1602 (w), 1506 (m).

HRMS (ESI/QTOF) m/z: $[M + Na]^+$ Calcd for $C_{29}H_{29}NNaO_5S_2^+$ 558.1379; Found 558.1383.

(Z)-N-(4-methoxyphenyl)-N-(1-(3-(phenylsulfonyl)bicyclo[1.1.1]pentan-1-yl)buta-1,3-dien-1-yl)methanesulfonamide (37e)

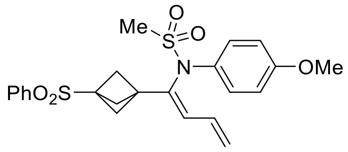

Starting from N-VBX **29b** (68 mg, 0.10 mmol) and commercially available tributyl(ethenyl)stannae (59 µL, 0.20 mmol, 2.0 equiv.), **37e** (26 mg, 57 µmol, 57% yield) was obtained as a white solid.

R$_f$ = 0.38 (40% EtOAc/pentane).

m.p. 115.5–129.2 °C (melt).

^{1}H NMR (400 MHz, $CDCl_3$) δ 7.84 – 7.78 (m, 2H, Ar*H*), 7.68 – 7.60 (m, 1H, Ar*H*), 7.60 – 7.51 (m, 2H, Ar*H*), 7.36 – 7.28 (m, 2H, Ar*H*), 6.90 – 6.79 (m, 2H, Ar*H*), 6.65 (dt, *J* = 17.0, 10.3 Hz, 1H, CHC*H*CH_2), 6.16 (dt, *J* = 10.8, 0.7 Hz, 1H, C*H*CHCH_2), 5.47 (ddd, *J* = 17.1, 1.6, 0.8 Hz, 1H, C=C*H_2*), 5.37 (ddd, *J* = 10.1, 1.6, 0.8 Hz, 1H, C=C*H_2*), 3.80 (s, 3H, OC*H_3*), 3.03 (s, 3H, SO_2C*H_3*), 2.10 (s, 6H, H_{BCP}).

^{13}C NMR (101 MHz, $CDCl_3$) δ 158.4, 136.8, 135.5, 133.9, 132.5, 132.4, 131.0, 129.3, 128.7, 126.4, 122.7, 114.8, 55.6, 52.5, 51.1, 41.9, 40.4.

IR (ν_{max}, cm^{-1}) 3006 (w), 2931 (w), 1508 (m).

HRMS (ESI/QTOF) m/z: $[M + Na]^+$ Calcd for $C_{23}H_{25}NNaO_5S_2^+$ 482.1066; Found 482.1072.

Methyl (Z)-3-(1-((N-(4-methoxyphenyl)-4-nitrophenyl)sulfonamido)-2-phenylvinyl)bicyclo[1.1.1]pentane-1-carboxylate (37f)

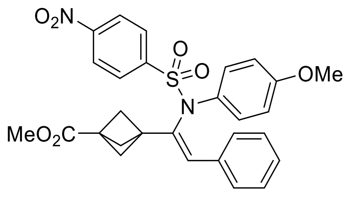

Starting from N-VBX **29f** (71 mg, 0.10 mmol) and commercially available tributyl(phenyl)stannae (65 µL, 0.20 mmol, 2.0 equiv.), **37f** (33 mg, 62 µmol, 62% yield) was obtained as a bright yellow amorphous solid.
R_f = 0.32 (15% EtOAc/pentane).
^{1}H NMR (400 MHz, $CDCl_3$) δ 8.05 – 7.97 (m, 2H, Ar*H*), 7.57 – 7.51 (m, 2H, Ar*H*), 7.38 – 7.32 (m, 2H, Ar*H*), 7.30 – 7.27 (m, 2H, Ar*H*), 7.23 – 7.16 (m, 1H, Ar*H*), 7.15 – 7.07 (m, 2H, Ar*H*), 6.86 – 6.77 (m, 2H, Ar*H*), 6.51 (s, 1H, vinyl*H*), 3.79 (s, 3H, OC*H₃*), 3.66 (s, 3H, OC*H₃*), 2.16 (br s, 6H, H_{BCP}).
^{13}C NMR (101 MHz, $CDCl_3$) δ 170.3, 158.3, 149.9, 145.6, 136.3, 134.3, 132.4, 130.7, 128.9, 128.6, 128.5, 126.2, 123.6, 114.7, 55.6, 53.8, 51.9, 43.5, 36.9.
One aromatic carbon not resolved.
IR (ν_{max}, cm^{-1}) 3104 (w), 3058 (w), 2991 (w), 2966 (m), 2925 (w), 2898 (w), 2835 (w), 1727 (m), 1644 (m), 1626 (m), 1609 (m).
HRMS (ESI/QTOF) m/z: $[M + Na]^+$ Calcd for $C_{28}H_{26}N_2NaO_7S^+$ 557.1353; Found 557.1361.

(Z)-N-(4-methoxyphenyl)-4-methyl-N-(1-(3-phenylbicyclo[1.1.1]pentan-1-yl)buta-1,3-dien-1-yl)benzenesulfonamide (37g)

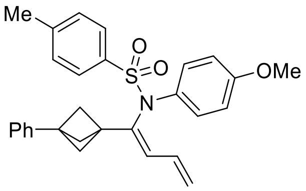

Starting from N-VBX **29g** (69 mg, 0.10 mmol) and commercially available tributyl(ethenyl)stannane (59 µL, 0.20 mmol, 2.0 equiv.), **37g** (30 mg, 63 µmol, 63% yield) was obtained as a white solid.
R_f = 0.46 (15% EtOAc/pentane).
m.p. 92.3–102.8 °C (melt).
^{1}H NMR (400 MHz, $CDCl_3$) δ 7.67 – 7.60 (m, 2H, Ar*H*), 7.31 – 7.11 (m, 9H, Ar*H*), 6.85 – 6.76 (m, 2H, Ar*H*), 6.50 (ddd, *J* = 16.9, 10.9, 10.2 Hz, 1H, CHC*H*CH_2), 6.23 (d, *J* = 10.9 Hz, 1H, C*H*CHCH_2), 5.38 (ddd, *J* = 16.9, 1.8, 0.8 Hz, 1H, C=C*H₂*), 5.19 – 5.11 (m, 1H, C=C*H₂*), 3.79 (s, 3H, OC*H₃*), 2.41 (s, 3H, ArC*H₃*), 2.06 (s, 6H, H_{BCP}).
^{13}C NMR (101 MHz, $CDCl_3$) δ 158.5, 143.6, 140.6, 139.1, 138.1, 133.0, 132.6, 130.8, 129.5, 129.0, 128.3, 128.0, 126.6, 126.2, 120.3, 114.2, 55.5, 54.3, 42.0, 41.1, 21.7.
IR (ν_{max}, cm^{-1}) 3069 (w), 3023 (w), 2975 (m), 2912 (m), 2871 (w), 2839 (w), 1642 (w), 1602 (w), 1504 (s).
HRMS (ESI/QTOF) m/z: $[M + Na]^+$ Calcd for $C_{29}H_{29}NNaO_3S^+$ 494.1760; Found 494.1763.

Methyl (Z)-3-(1-((2-bromophenyl)thio)-2-phenylvinyl)bicyclo[1.1.1]pentane-1-carboxylate (37h)

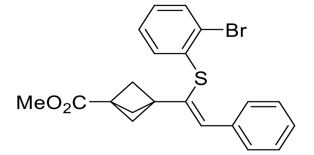

Starting from S-VBX **35b** (176 mg, 0.300 mmol) and commercially available tributyl(phenyl)stannane (103 µL, 0.315 mmol, 1.05 equiv.), **37h** (97.0 mg, 23.3 µmol, 78% yield) was obtained as a yellow oil.

R_f = 0.33 (15% Et_2O/pentane).
^{1}H NMR (400 MHz, $CDCl_3$) δ 7.65 – 7.59 (m, 2H, Ar*H*), 7.52 (dd, *J* = 7.9, 1.3 Hz, 1H, Ar*H*), 7.36 – 7.27 (m, 3H, Ar*H*), 7.22 (dd, *J* = 7.9, 1.7 Hz, 1H, Ar*H*), 7.15 (td, *J* = 7.6, 1.3 Hz, 1H, Ar*H*), 7.04 – 6.97 (m, 2H, Ar*H* + vinyl*H*), 3.65 (s, 3H, OC*H₃*), 2.10 (s, 6H, H_{BCP}).
^{13}C NMR (101 MHz, $CDCl_3$) δ 170.6, 137.4, 136.9, 135.4, 133.2, 131.0, 129.4, 129.4, 128.5, 128.4, 127.6, 127.3, 123.3, 53.5, 51.8, 45.7, 36.4.
IR (ν_{max}, cm^{-1}) 3059 (w), 2992 (m), 2951 (m), 2914 (m), 2878 (m), 1731 (s).
HRMS (ESI/QTOF) m/z: $[M + Na]^+$ Calcd for $C_{21}H_{19}BrNaO_2S^+$ 437.0181; Found 437.0171.

(Z)-Benzyl(2-(4-methoxyphenyl)-1-(3-(phenylsulfonyl)bicyclo[1.1.1]pentan-1-yl)vinyl)sulfane (37i)

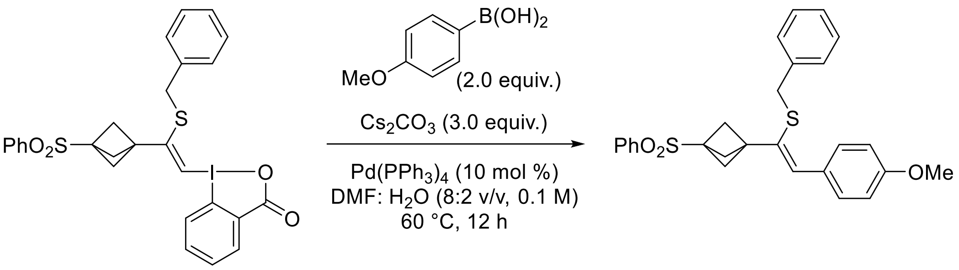

Following a literature procedure,[12] under nitrogen gas, a microwave vial equipped with a stirring bar was charged with S-VBX **35a** (60.3 mg, 0.100 mmol, 1.00 equiv.), $Pd(PPh_3)_4$ (11.7 mg, 10.0 µmol, 10 mol %), Cs_2CO_3 (97.8 mg, 0.300 mmol, 3.00 equiv.), and 4-methoxyboronic acid (30.4 mg, 0.200 mmol, 2.00 equiv.) Then, DMF (0.8 mL) and H_2O (0.2 mL) were added and the resulting mixture was stirred at 60 °C for 12 h. The mixture was cooled to room temperature, diluted with Et_2O (5 mL), and washed with H_2O (3 mL) and brine (3 mL). The organic layer was dried over Na_2SO_4 and concentrated under reduced pressure. The residue was purified by flash column chromatography on silica gel (10% → 50% Et_2O in pentane) to afford **37i** as a yellow oil (19.0 mg, 41.1 µmol, 41% yield). 1:0.23 ratio of regioisomers (Z:E) was observed based on the comparison of the vinyl H (**^{1}H NMR** (400 MHz, $CDCl_3$) δ 6.6(Z) and 6.4(E)). The spectrum was assigned by 2D NMR.
R_f = 0.56 (30% Et_2O/pentane).
^{1}H NMR (400 MHz, $CDCl_3$) δ 7.86 – 7.81 (m, 2H, Ar*H*), 7.72 – 7.67 (m, 2H, Ar*H*), 7.61 – 7.55 (m, 3H, Ar*H*), 7.22 – 7.15 (m, 3H, Ar*H*), 7.04 – 6.98 (m, 2H, Ar*H*), 6.90 – 6.84 (m, 2H, Ar*H*), 6.55 (s, 1H, vinyl*H*), 3.83 (s, 3H, OCH_3), 3.62 (s, 2H, CH_2), 1.97 (s, 6H, H_{BCP}).
^{13}C NMR (101 MHz, $CDCl_3$) δ 159.5, 138.0, 137.0, 134.0, 133.8, 133.3, 130.8, 129.2, 129.1, 128.7, 128.4, 127.7, 127.3, 113.9, 55.4, 51.6, 51.2, 45.4, 37.5.
IR (ν_{max}, cm^{-1}) 3063 (w), 3006 (w), 2957 (w), 2919 (m), 2885 (w), 2849 (w), 1604 (m), 1510 (m).
HRMS (Sicrit plasma/LTQ-Orbitrap) m/z: $[M + H]^+$ Calcd for $C_{27}H_{27}O_3S_2^+$ 463.1396; Found 463.1382.

Preparation of copper species: (bpy)Cu(CF_3)$_3$

[12] W. Ding, J. Chai, C. Wang, J. Wu, N. Yoshikai, *J. Am. Chem. Soc.* **2020**, *142*, 8619–8624.

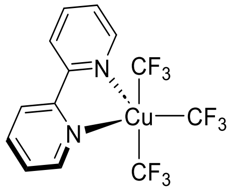

Following a reported procedure,[13] copper(I) iodide (1.14 g, 5.97 mmol, 1.0 equiv.), 2,2'-bipyridine (932 mg, 5.97 mmol, 1.0 equiv.) and silver(I) fluoride (3.03 g, 23.9 mmol, 4.0 equiv.) were added to an oven-dried 50 mL flask in the glove box. Outside the glove box, dry DMF (15.9 mL, 0.38 M) was added and the flask was wrapped with alumina foil. After 30 min of stirring at room temperature, $TMSCF_3$ (5.29 mL, 5.09 g, 35.8 mmol, 6.0 equiv.) was slowly added over 1 h using a syringe pump. The reaction solution was stirred for further 18 h at room temperature before being filtered through a pad of celite. After washing with acetone, the obtained filtrate was concentrated under reduced pressure. Methanol (30 mL) was added and the resulting residue was allowed to crystallize overnight in the freezer at -20 °C. The yellow solid was filtered off and dried under vacuum to yield the titled product (1.49 g, 3.49 mmol, 59% yield).

^{1}H NMR (400 MHz, DMSO) δ 9.23 (dd, *J* = 5.4, 1.6 Hz, 1H), 8.80 (d, *J* = 8.1 Hz, 1H), 8.38 (td, *J* = 7.9, 1.6 Hz, 1H), 7.91 (ddd, *J* = 7.7, 5.2, 1.1 Hz, 1H).

^{13}C NMR (101 MHz, DMSO) δ 149.2, 148.9, 141.0, 127.2, 123.3.

^{19}F NMR (376 MHz, DMSO) δ -23.95 (p, *J* = 9.1 Hz), -36.09 (q, *J* = 9.1 Hz).

Spectroscopic data in accordance with the literature.[13]

General Procedure GP6 for trifluoromethylation

The thermal reactions were performed in Biotage® microwave reaction vials (size: 2.0-5.0 mL) using Carl Roth crimp caps ROTILABO® ND20 with borehole and Butyl/PTFE septum and 10 mm long stirring bars. (phen)$Cu(CF_3)_3$ (64.0 mg, 0.150 mmol, 1.50 equiv.) and the vinylbenziodoxolone (0.100 mmol, 1.00 equiv.) were balanced (outside glove box) into the reaction vial before being capped. The vial was evacuated (under 0.5 mbar) and flushed with nitrogen. This procedure was repeated three times before dry DMF (4.0 mL, 25 mM) was added under nitrogen atmosphere. The reaction vial was transferred to a preheated aluminium block (120 °C) and stirred for 1 h with 900 rpm. After 1 h, the reaction mixture was allowed to cool down to room temperature. Diethyl ether (twice the volume of DMF) was added and the organic layer was washed three times with 5% ammonia solution (removal of copper complexes). The combined organic layers were washed with brine, dried Na_2SO_4, filtrered and evaporated under reduced pressure.

(Z)-*N*-(4-methoxyphenyl)-4-methyl-N-(3,3,3-trifluoro-1-(3-(phenylsulfonyl)bicyclo[1.1.1]pentan-1-yl)prop-1-en-1-yl)benzenesulfonamide (38a)

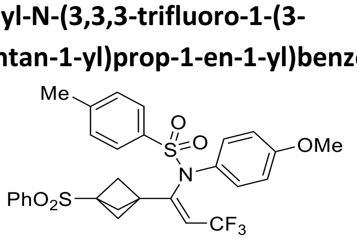

Starting from N-VBX **29a** (75.6 mg, 0.100 mmol), the desired product **38a** was obtained as a yellow oil (44.0 mg, 76.2 µmol, 76% yield).

[13] T. M. Milzarek, J. Waser, *Angew. Chem. Int. Ed.* **2023**, *62*, e202306128.

R_f = 0.69 (50% EtOAc/pentane).

^{1}H NMR (400 MHz, $CDCl_3$) δ 7.84 – 7.72 (m, 2H, Ar*H*), 7.70 – 7.63 (m, 1H, Ar*H*), 7.61 – 7.52 (m, 4H, Ar*H*), 7.23 – 7.14 (m, 4H, Ar*H*), 6.83 – 6.71 (m, 2H, Ar*H*), 5.60 (q, *J* = 7.7 Hz, 1H, vinyl*H*), 3.79 (s, 3H, OCH_3), 2.39 (s, 3H, $ArCH_3$), 2.14 (s, 6H, H_{BCP}).

^{13}C NMR (101 MHz, $CDCl_3$) δ 159.2, 144.5, 136.5, 136.0, 134.1, 130.7, 129.4, 129.1, 128.8, 128.4, 121.3 (q, *J* = 270.9 Hz), 118.73 (q, *J* = 34.5 Hz), 114.6, 55.6, 52.8, 50.8, 42.3, 21.7.

2 quartets are not fully resolved

^{19}F NMR (376 MHz, $CDCl_3$) δ -60.0.

IR ($ν_{max}$, cm^{-1}) 2973 (w), 2967 (w), 2959 (w), 2954 (w), 2945 (w), 2940 (w), 2933 (w), 2929 (w), 2916 (w), 2912 (w), 1669 (w), 1661 (w), 1603 (w), 1508 (m).

HRMS (ESI/QTOF) m/z: $[M + Na]^+$ Calcd for $C_{28}H_{26}F_3NNaO_5S_2^+$ 600.1097; Found 600.1116.

Methyl (Z)-3-(3,3,3-trifluoro-1-(p-tolyloxy)prop-1-en-1-yl)bicyclo[1.1.1]pentane-1-carboxylate (38b)

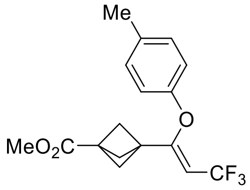

Starting from O-VBX **30d** (101 mg, 0.200 mmol), the desired product **38b** was obtained as a yellow oil (53.1 mg, 0.163 mmol, 81% yield).

R_f = 0.43 in 10% Et_2O/pentane.

^{1}H NMR (400 MHz, $CDCl_3$) δ 7.15 – 7.06 (m, 2H, Ar*H*), 6.91 – 6.83 (m, 2H, Ar*H*), 5.22 (q, *J* = 7.5 Hz, 1H, vinyl*H*), 3.61 (s, 3H, OCH_3), 2.31 (s, 3H, $ArCH_3$), 2.01 (s, 6H, H_{BCP}).

^{13}C NMR (101 MHz, $CDCl_3$) δ 169.5, 159.2 (q, *J* = 5.5 Hz), 133.9, 130.2, 122.81 (q, *J* = 269.9 Hz), 118.4, 104.1 (q, *J* = 34.8 Hz), 53.9, 51.9, 39.6, 37.9, 20.8.

Quartet at δ 159.2 not fully resolved.

^{19}F NMR (376 MHz, $CDCl_3$) δ -57.9.

IR ($ν_{max}$, cm^{-1}) 3041 (w), 2996 (w), 2961 (w), 2887 (w), 1735 (m), 1675 (m).

HRMS (ESI/QTOF) m/z: $[M + H]^+$ Calcd for $C_{17}H_{18}F_3O_3^+$ 327.1203; Found 327.1205.

(Z)-1-Phenyl-3-(3,3,3-trifluoro-1-(*p*-tolyloxy)prop-1-en-1-yl)bicyclo[1.1.1]pentane (38c)

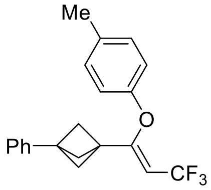

Starting from O-VBX **30h** (52.2 mg, 0.100 mmol), the desired product **38c** was obtained as a colorless oil (30.8 mg, 89.4 µmol, 89% yield).

R_f = 0.68 (5% Et_2O/pentane).

^{1}H NMR (400 MHz, $CDCl_3$) δ 7.29 – 7.23 (m, 2H, Ar*H*), 7.22 – 7.16 (m, 1H, Ar*H*), 7.14 – 7.06 (m, 4H, Ar*H*), 6.96 – 6.90 (m, 2H, Ar*H*), 5.29 (q, *J* = 7.6 Hz, 1H, vinyl*H*), 2.32 (s, 3H, $ArCH_3$), 2.02 (s, 6H, H_{BCP}).

^{13}C NMR (101 MHz, $CDCl_3$) δ 160.3 (q, *J* = 6.1 Hz), 153.7, 139.5, 133.5, 130.1, 128.3, 127.1, 126.0, 123.0 (1, *J* = 268.3 Hz), 118.4, 103.6 (q, *J* = 34.5 Hz), 54.5, 42.1, 38.9, 20.9.

^{19}F NMR (376 MHz, $CDCl_3$) δ -57.9.
HRMS (Sicrit plasma/LTQ-Orbitrap) m/z: $[M + H]^+$ Calcd for $C_{21}H_{20}F_3O^+$ 345.1461; Found 345.1461.
IR (ν_{max}, cm^{-1}) 2981 (w), 2971 (w), 2966 (w), 2915 (w), 2911 (w), 2875 (w), 1684 (w), 1675 (m), 1507 (m).

Methyl (S,Z)-3-(cysteine)-3,3,3-trifluoroprop-1-en-1-yl)bicyclo[1.1.1]pentane-1-carboxylate (38d)

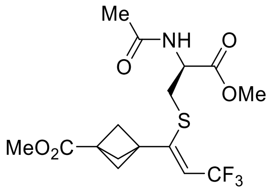

Starting from S-VBX **35c** (57.3 mg, 0.100 mmol), the desired product **38d** was obtained as a yellow oil (22.9 mg, 57.9 µmol, 58% yield).
R$_f$ = 0.51 (10% MeOH/DCM).
^{1}H NMR (400 MHz, $CDCl_3$) δ 6.26 (d, *J* = 7.2 Hz, 1H, N*H*), 5.80 (q, *J* = 7.8 Hz, 1H, vinyl*H*), 4.80 (dt, *J* = 7.2, 4.6 Hz, 1H, NC*H*), 3.77 (s, 3H, OC*H$_3$*), 3.69 (s, 3H, OC*H$_3$*), 3.41 (dd, *J* = 13.5, 4.6 Hz, 1H, SC*H$_2$*), 3.26 (dd, *J* = 13.5, 4.6 Hz, 1H, SC*H$_2$*), 2.27 (s, 6H, H_{BCP}), 2.02 (s, 3H, C=OCH$_3$).
^{13}C NMR (101 MHz, $CDCl_3$) δ 170.3, 169.9, 169.7, 146.7 (q, *J* = 5.4 Hz), 122.3 (q, *J* = 271.1 Hz), 121.7 (q, *J* = 34.7 Hz), 54.1, 53.0, 52.5, 52.0, 44.5, 36.9, 34.5, 23.1.
^{19}F NMR (376 MHz, $CDCl_3$) δ -57.4.
IR (ν_{max}, cm^{-1}) 2994 (w), 2957 (w), 2921 (w), 2884 (w), 1734 (s), 1661 (m).
HRMS (ESI/QTOF) m/z: $[M + Na]^+$ Calcd for $C_{16}H_{20}F_3NNaO_5S^+$ 418.0906; Found 418.0915.

5.2. Hydrogenation

***N*-(4-methoxyphenyl)-4-methyl-*N*-(2-phenyl-1-(3-(phenylsulfonyl)bicyclo[1.1.1]pentan-1-yl)ethyl)benzenesulfonamide (39)**

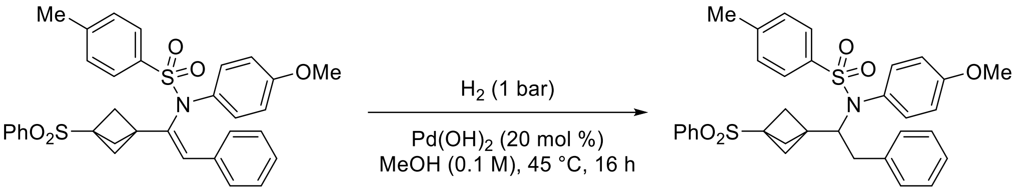

In an oven-dried microwave vial charged with BCP sulfonamide **37c** (63.7 mg, 0.100 mmol, 1.00 equiv.) and $Pd(OH)_2$ (14.0 mg, 20.0 µmol, 20 mol %, 20 wt% Pd) was added methanol (1.0 mL). The mixture was subjected to 1 bar of hydrogen at 45 °C. After 16 h, the mixture was filtered through filter paper and the filtrate was concentrated. The crude was purified by flash column chromatography (30% EtOAc in pentane) to afford the title compound **39** as a white solid (41.8 mg, 71.1 µmol, 71% yield).
R$_f$ = 0.40 (30% EtOAc/pentane).
m.p. 73.9–79.2 °C (melt).
^{1}H NMR (400 MHz, $CDCl_3$) δ 7.80 – 7.71 (m, 2H, Ar*H*), 7.67 – 7.61 (m, 1H, Ar*H*), 7.56 – 7.48 (m, 2H, Ar*H*), 7.39 – 7.34 (m, 2H, Ar*H*), 7.29 – 7.19 (m, 3H, Ar*H*), 7.14 – 7.07 (m, 4H, Ar*H*), 6.94 – 6.86 (m, 2H, Ar*H*), 6.81 – 6.73 (m, 2H, Ar*H*), 4.82 (dd, *J* = 8.5, 6.5 Hz, 1H, NC*H*CH$_2$), 3.82 (s, 3H,

ArOCH_3), 2.77 (dd, J = 14.3, 6.5 Hz, 1H, NCHCH_2), 2.61 (dd, J = 14.3, 8.6 Hz, 1H, NCHCH_2), 2.36 (s, 3H, ArCH_3), 1.92 (dd, J = 9.3, 2.1 Hz, 3H, H_{BCP}), 1.72 (dd, J = 9.3, 2.1 Hz, 3H, H_{BCP}).
^{13}C NMR (101 MHz, $CDCl_3$) δ 159.9, 143.4, 137.5, 137.4, 136.8, 133.8, 133.7, 129.4, 129.3, 129.2, 128.9, 128.8, 128.8, 128.7, 128.0, 127.6, 127.0, 114.1, 59.6, 55.6, 52.0, 50.7, 42.3, 37.9, 21.6.
HRMS (APCI/QTOF) m/z: $[M + Na]^+$ Calcd for $C_{33}H_{33}NNaO_5S_2^+$ 610.1692; Found 610.1691.
IR (ν_{max}, cm^{-1}) 3059 (w), 3002 (w), 2963 (w), 2923 (w), 2892 (w), 2839 (w), 1733 (w), 1509 (m).

Methyl 3-(2-phenyl-1-(p-tolyloxy)ethyl)bicyclo[1.1.1]pentane-1-carboxylate (40_

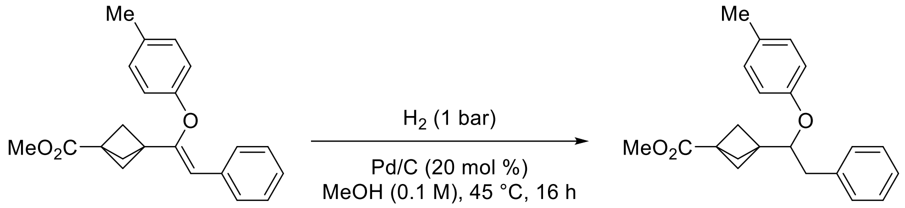

In an oven-dried microwave vial charged with O-VBX **37b** (44.2 mg, 0.132 mmol, 1.00 equiv.) and Pd/C (28.1 mg, 20 µmol, 20 mol %, 10 wt% Pd) was added methanol (1.3 mL). The mixture was subjected to 1 bar of hydrogen at 45 °C. After 16 h, the mixture was filtered through filter paper and the filtrate was concentrated. The crude was purified by flash column chromatography (10% EtOAc in pentane) to afford the title compound **40** as colorless oil (34.0 mg, 0.101 mmol, 76% yield).
R_f = 0.47 (10% EtOAc/pentane).
^{1}H NMR (400 MHz, $CDCl_3$) δ 7.30 – 7.24 (m, 4H, ArH), 7.22 – 7.17 (m, 1H, ArH), 7.02 – 6.97 (m, 2H, ArH), 6.72 – 6.66 (m, 2H, ArH), 4.46 (dd, J = 8.1, 5.0 Hz, 1H, OCHCH$_2$), 3.66 (s, 3H, CO_2CH_3), 2.97 (dd, J = 14.2, 8.1 Hz, 1H, OCHCH_2), 2.84 (dd, J = 14.2, 5.0 Hz, 1H, OCHCH_2), 2.26 (s, 3H, ArCH_3), 2.04 – 1.94 (m, 6H, H_{BCP}).
^{13}C NMR (101 MHz, $CDCl_3$) δ 170.5, 156.9, 138.3, 130.4, 129.9, 129.4, 128.5, 126.5, 116.2, 77.7, 51.8, 50.9, 42.1, 38.6, 38.0, 20.6.
HRMS (nanochip-ESI/LTQ-Orbitrap) m/z: $[M + Na]^+$ Calcd for $C_{22}H_{24}NaO_3^+$ 359.1618; Found 359.1628.
IR (ν_{max}, cm^{-1}) 3027 (w), 2989 (m), 2950 (m), 2922 (m), 2882 (w), 1735 (s).

5.3. Oxidation

Methyl 3-(3-phenyl-2-(*p*-tolyloxy)oxiran-2-yl)bicyclo[1.1.1]pentane-1-carboxylate (41)

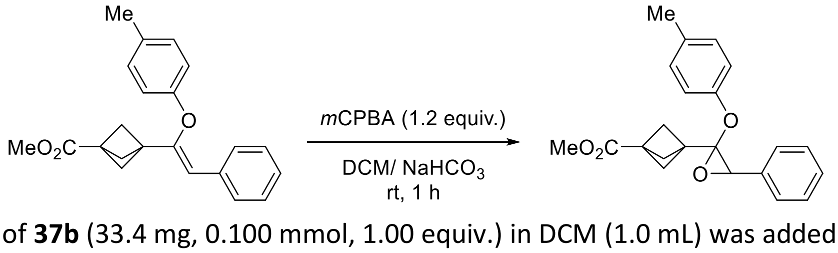

To a solution of **37b** (33.4 mg, 0.100 mmol, 1.00 equiv.) in DCM (1.0 mL) was added sat. $NaHCO_3$ (1.0 mL) and then 3-chloroperbenzoic acid (20.7 mg, 70-75%, 0.120 mmol, 1.20 equiv.). The resulting mixture was stirred at room temperature for 1 h. A saturated aqueous solution of $NaHCO_3$ (1 mL) was added to the reaction mixture and it was stirred for 10 min. A saturated aqueous solution of $NaHCO_3$ (3 mL) was added to the reaction mixture and it was stirred for 10 min. The organic layers were extracted three times with DCM. The combined extracts were

washed with brine and dried over Na_2SO_4, and concentrated under reduced pressure to afford **41** as yellowish oil (35.0 mg, 0.100 mmol, 100% yield).

^{1}H NMR (400 MHz, $CDCl_3$) δ 7.42 – 7.34 (m, 2H, Ar*H*), 7.34 – 7.28 (m, 3H, Ar*H*), 7.10 – 6.97 (m, 4H, Ar*H*), 3.94 (s, 1H, OC*H*), 3.64 (s, 3H, OC*H₃*), 2.28 (s, 3H, ArC*H₃*), 2.12 (dd, *J* = 9.4, 1.9 Hz, 3H, H_{BCP}), 2.00 (dd, *J* = 9.4, 1.9 Hz, 3H, H_{BCP}).

^{13}C NMR (101 MHz, $CDCl_3$) δ 169.9, 153.6, 133.4, 132.4, 129.9, 128.5, 128.1, 127.6, 118.1, 85.1, 61.5, 51.9, 51.8, 40.0, 37.7, 20.7.

HRMS (ESI/QTOF) m/z: [M + H]$^+$ Calcd for $C_{22}H_{23}O_4^+$ 351.1591; Found 351.1591.

IR (ν_{max}, cm^{-1}) 2995 (m), 2958 (w), 2923 (m), 2884 (w), 1731 (s), 1612 (w).

Methyl 3-(2-acetoxy-2-phenylacetyl)bicyclo[1.1.1]pentane-1-carboxylate (42)

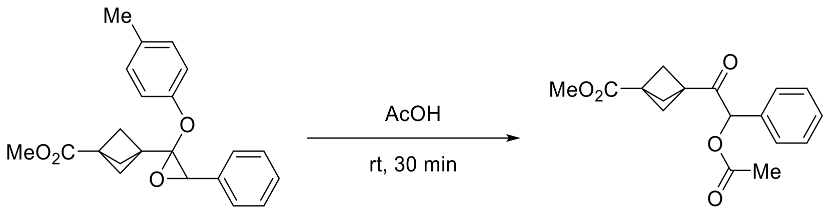

A solution of 3-ethyl-2-(p-tolyloxy)-2-((p-tolyloxy)methyl)oxirane **41** (35.0 mg, 0.100 mmol, 1.0 equiv.) in acetic acid (0.5 mL) was stirred at room temperature for 30 minutes. The excess of acetic acid was removed by co-evaporation with toluene. The crude product was purified by preparatory reverse phase HPLC (t_R = 17-18 min, gradient: 5-95% MeCN in 25 min) to yield the desired product as colourless oil (19.5 mg, 64.5 µmol, 65% yield).

^{1}H NMR (400 MHz, $CDCl_3$) δ 7.44 – 7.38 (m, 3H, Ar*H*), 7.38 – 7.32 (m, 2H, Ar*H*), 6.11 (s, 1H, COC*H*), 3.64 (d, *J* = 1.0 Hz, 3H, OC*H₃*), 2.20 – 2.10 (m, 9H, COC*H₃* + H_{BCP}).

^{13}C NMR (101 MHz, $CDCl_3$) δ 199.9, 170.4, 169.6, 132.6, 129.9, 129.3, 129.0, 78.7, 53.4, 52.0, 42.2, 38.2, 20.8.

HRMS (Sicrit plasma/LTQ-Orbitrap) m/z: [M + H]$^+$ Calcd for $C_{17}H_{19}O_5^+$ 303.1227; Found 303.1227.

IR (ν_{max}, cm^{-1}) 3006 (w), 2954 (w), 2925 (w), 2887 (w), 1739 (s), 1722 (s).

Methyl (Z)-3-(1-((2-bromophenyl)sulfonyl)-2-phenylvinyl)bicyclo[1.1.1]pentane-1-carboxylate (43)

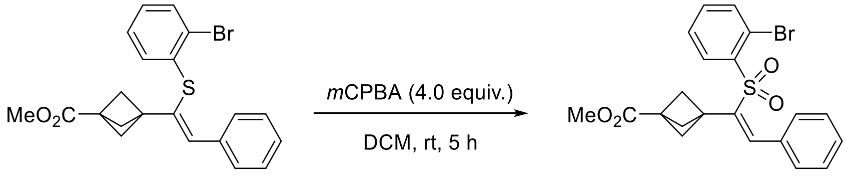

To a solution of **37h** (41.5 mg, 0.100 mmol, 1.00 equiv.) in DCM (1 mL) was added 3-chloroperbenzoic acid (69.0 mg, 0.400 mmol, 1.20 equiv.). The resulting mixture was stirred at room temperature for 5 h. A saturated aqueous solution of $NaHCO_3$ (3 mL) was added to the reaction mixture and it was stirred for 10 min. The organic layers were extracted three times with DCM. The combined extracts were washed with brine and dried over Na_2SO_4, filtered and concentrated under reduced pressure. The crude product was purified by flash column chromatography (10% → 50% EtOAc in pentane) to afford vinyl sulfone **43** (41.4 mg, 92.5 µmol, 93% yield) as a yellow oil, which solidified upon standing.

R_f = 0.31 (30% EtOAc/pentane).

^{1}H NMR (400 MHz, $CDCl_3$) δ 7.64 (dd, *J* = 7.9, 1.8 Hz, 1H, Ar*H*), 7.46 (dd, *J* = 7.9, 1.2 Hz, 1H, Ar*H*), 7.16 (td, *J* = 7.6, 1.7 Hz, 1H, Ar*H*), 7.12 – 7.01 (m, 6H, Ar*H*), 7.00 (s, 1H, vinyl*H*), 3.71 (s, 3H, OCH_3), 2.46 (s, 6H, H_{BCP}).
^{13}C NMR (101 MHz, $CDCl_3$) δ 170.1, 140.8, 139.8, 138.3, 134.7, 133.9, 133.1, 131.5, 128.7, 128.5, 127.8, 127.2, 120.7, 54.5, 51.9, 41.2, 38.4.
HRMS (APCI/QTOF) m/z: $[M + H]^+$ Calcd for $C_{21}H_{20}BrO_4S^+$ 447.0260; Found 447.0252.
IR ($ν_{max}$, cm^{-1}) 3060 (w), 2994 (m), 2952 (w), 2919 (w), 2877 (w), 1727 (s).

6. Crystal structure

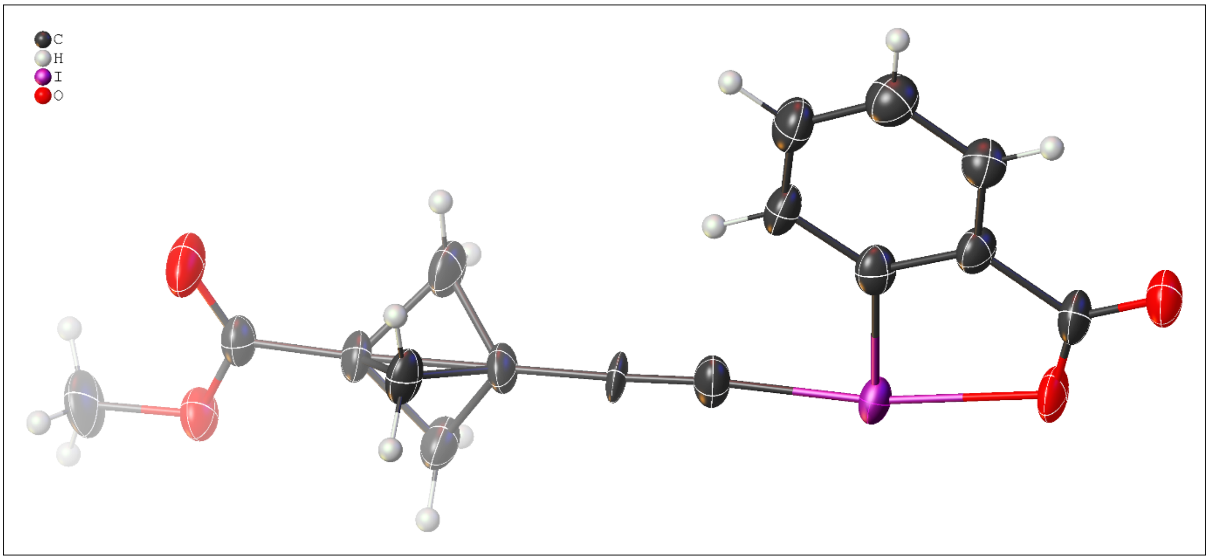

Figure S1. Ellipsoid plot (probability level 50%) of **EBX.2**

Compound	EBX.2
Formula	$C_{16}H_{13}O_4I$
$D_{calc.}$/ g cm^{-3}	1.802
m/mm^{-1}	17.361
Formula Weight	396.16
Colour	colourless
Shape	plate-shaped
Size/mm^3	0.24×0.12×0.01
T/K	140.00(10)
Crystal System	triclinic
Space Group	$P\bar{1}$
a/Å	7.3072(2)
b/Å	8.5375(2)
c/Å	12.3946(7)
a/°	106.552(3)
b/°	98.948(4)
g/°	91.684(2)
V/Å^3	729.96(5)
Z	2
Z'	1
Wavelength/Å	1.54184
Radiation type	CuKa
Q_{min}/°	3.776
Q_{max}/°	74.766
Measured Refl's.	2919
Indep't Refl's	2919
Refl's I≥2s(I)	2704
R_{int}	n/a
Parameters	192
Restraints	0
Largest Peak/e Å^{-3}	1.933
Deepest Hole/e Å^{-3}	-2.361
GooF	1.042
wR_2 (all data)	0.1823
wR_2	0.1764
R_1 (all data)	0.0689
R_1	0.0657

Crystals were grown by preparing solution of **EBX.2** in MeOH/ MeCN by slow diffusion over 2-3 days.

Analysis of the crystal: A suitable crystal with dimensions 0.24 × 0.12 × 0.01 mm^3 was selected and mounted on an XtaLAB Synergy R, DW system, HyPix-Arc 150 diffractometer. The crystal was kept at a steady T = 140.00(10) K during data collection. The structure was solved with the **ShelXT** 2018/2 (Sheldrick, 2015) solution program using dual methods and by using **Olex2** 1.5 (Dolomanov et al., 2009) as the graphical interface. The model was refined with **ShelXL** 2019/3 (Sheldrick, 2015) using full matrix least squares minimisation on $\boldsymbol{F^2}$.

Supplementary crystallographic data for this compound have been deposited at Cambridge Crystallographic Data Centre (CCDC **2443049**) and can be obtained free of charge via www.ccdc.cam.ac.uk/data_request/cif.

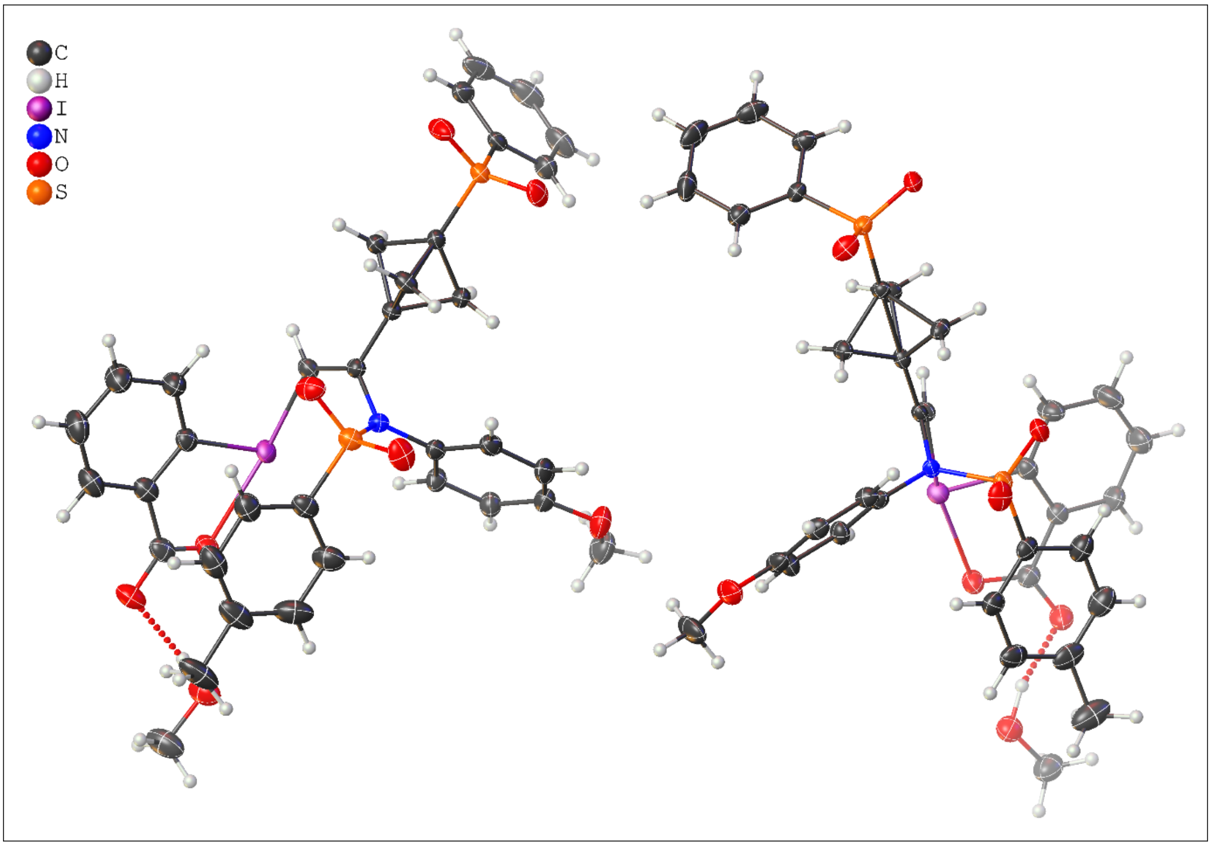

Figure S2. Ellipsoid plot (probability level 50%) of **29a**

**Compound**	**29a**
Formula	$C_{35}H_{34}NO_8S_2I$
$D_{calc.}$/ g cm^{-3}	1.555
m/mm^{-1}	9.082
Formula Weight	787.65
Colour	colourless
Shape	needle-shaped
Size/mm^3	0.34×0.03×0.02
T/K	139.99(10)
Crystal System	triclinic
Space Group	$P\bar{1}$
a/Å	8.16580(13)
b/Å	16.7718(3)
c/Å	25.4221(4)
a/°	77.6525(13)
b/°	86.6449(13)
g/°	81.8643(13)
V/Å^3	3365.48(9)
Z	4
Z'	2
Wavelength/Å	1.54184
Radiation type	Cu*K*[illegible]
Q_{min}/°	1.780
Q_{max}/°	74.225
Measured Refl's.	60077
Indep't Refl's	13131
Refl's I≥2*s*(I)	10821
R_{int}	0.0506
Parameters	861
Restraints	0
Largest Peak/e Å^{-3}	1.749
Deepest Hole/e Å^{-3}	-1.576
GooF	1.066
wR_2 (all data)	0.0882
wR_2	0.0845
R_1 (all data)	0.0475
R_1	0.0360

Crystals were grown by preparing solution of **29a** in MeOH by slow diffusion over 2-3 days.

Analysis of the crystal: A suitable crystal with dimensions 0.34 × 0.03 × 0.02 mm^3 was selected and mounted on an XtaLAB Synergy R, DW system, HyPix-Arc 150 diffractometer. The crystal was kept at a steady *T* = 139.99(10) K during data collection. The structure was solved with the **ShelXT** 2018/2 (Sheldrick, 2015) solution program using dual methods and by using **Olex2** 1.5 (Dolomanov et al., 2009) as the graphical interface. The model was refined with **ShelXL** 2019/3 (Sheldrick, 2015) using full matrix least squares minimisation on $\boldsymbol{F}^2$.

Supplementary crystallographic data for this compound have been deposited at Cambridge Crystallographic Data Centre (CCDC **2443048**) and can be obtained free of charge via www.ccdc.cam.ac.uk/data_request/cif.

7. DSC experiment of EBX.2

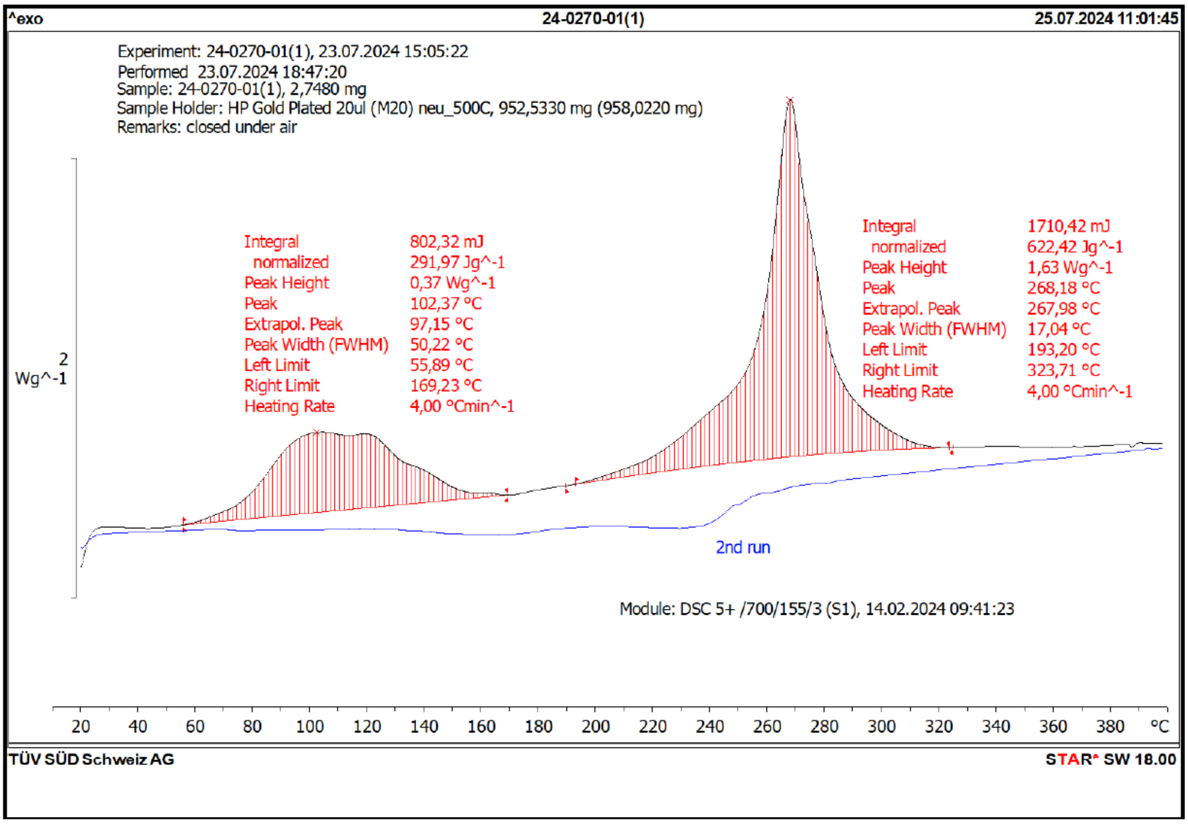

Figure S3. DSC spectrum of **EBX.2** measured at TÜV SÜD Schweiz AG.

8. Spectra of novel compounds

¹H NMR (400 MHz, $CDCl_3$) of compound **12**

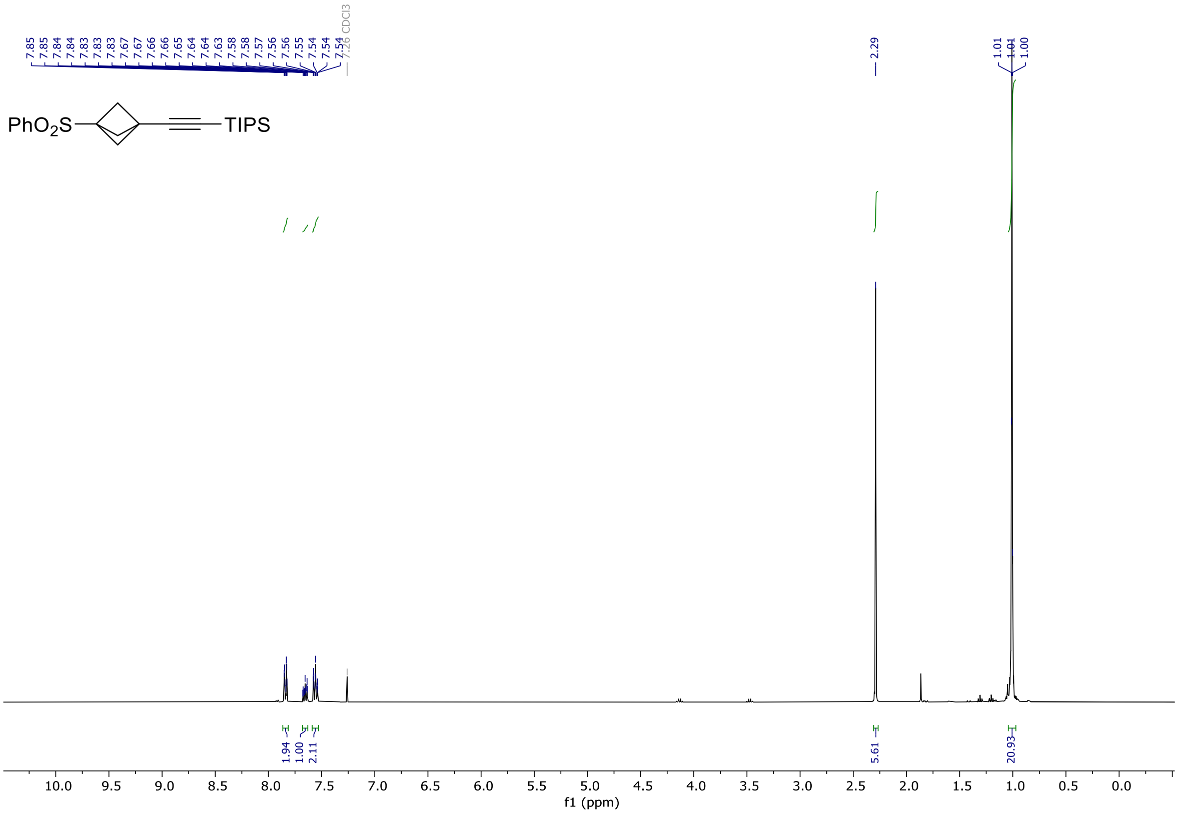

¹³C NMR (101 MHz, $CDCl_3$) of compound **12**

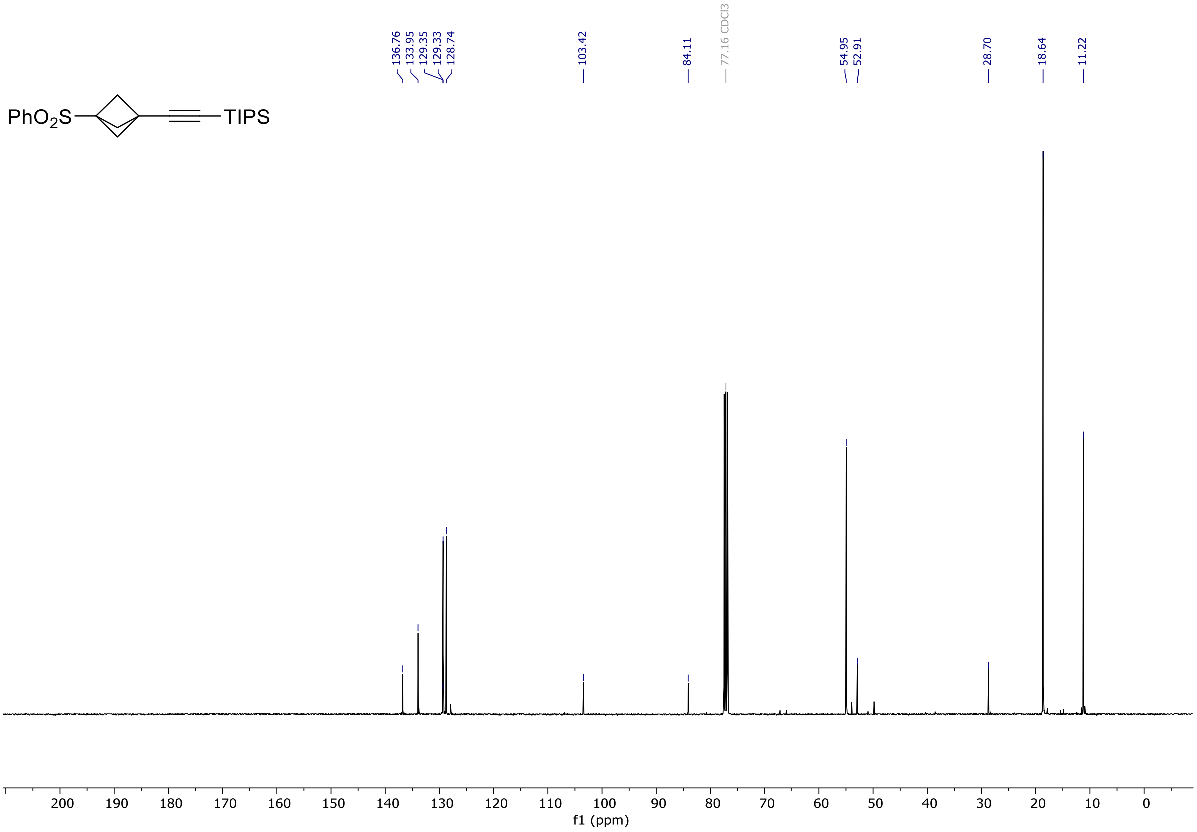

^{1}H NMR (400 MHz, $CDCl_3$) of compound **13**

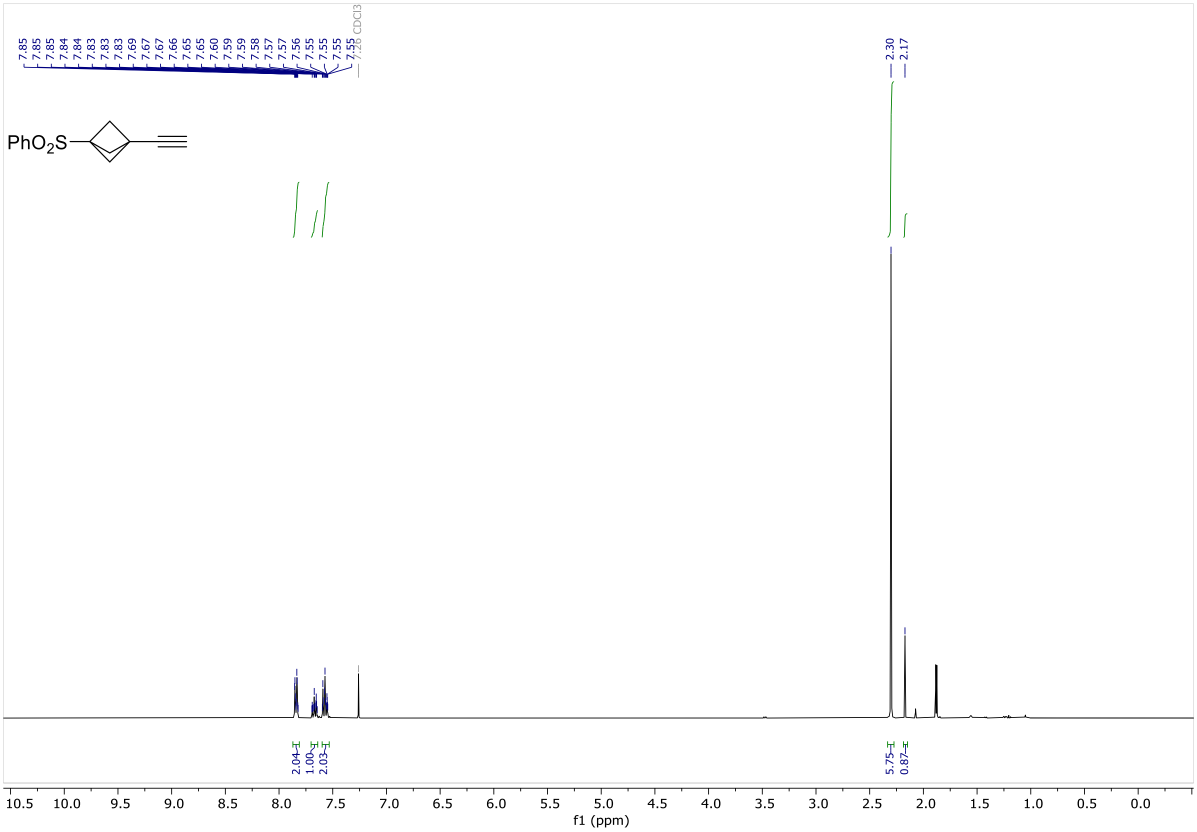

^{13}C NMR (101 MHz, $CDCl_3$) of compound **13**

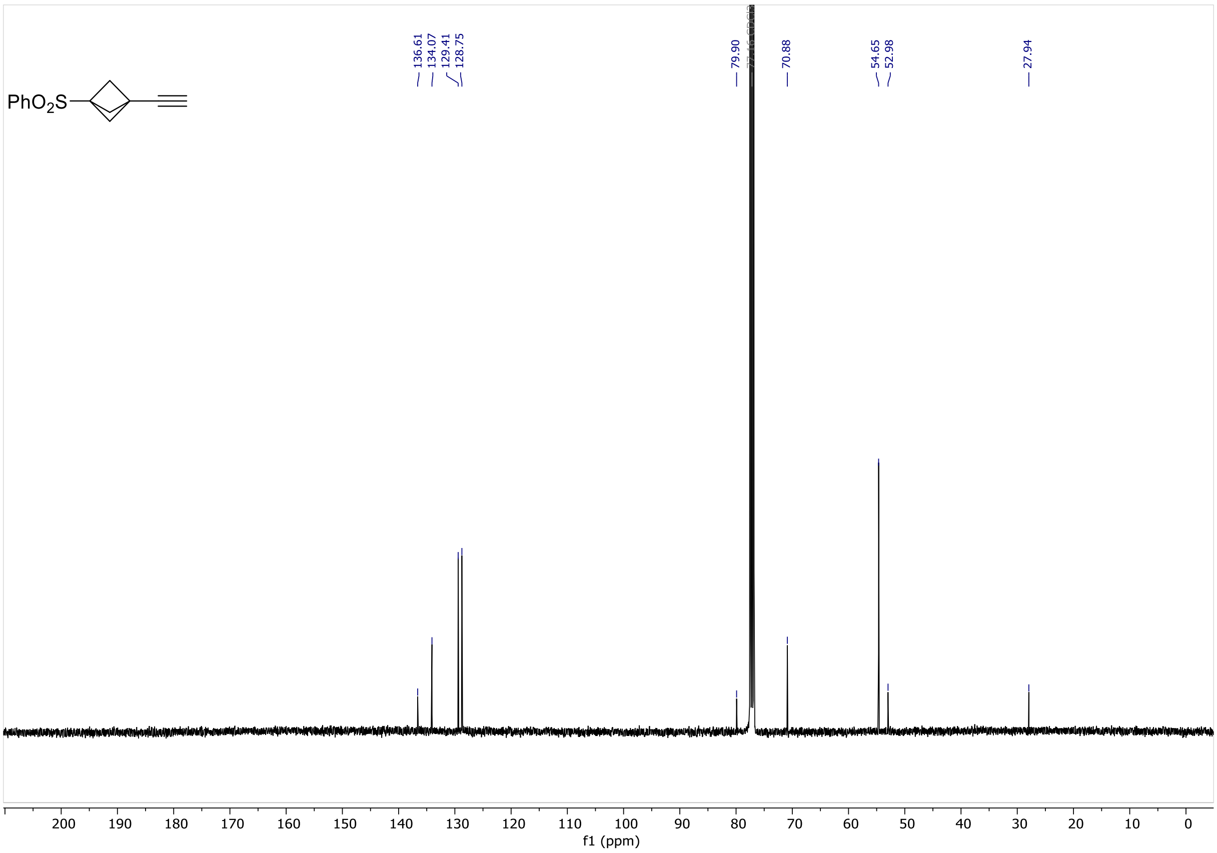

^{1}H NMR (400 MHz, $CDCl_3$) of compound **14**

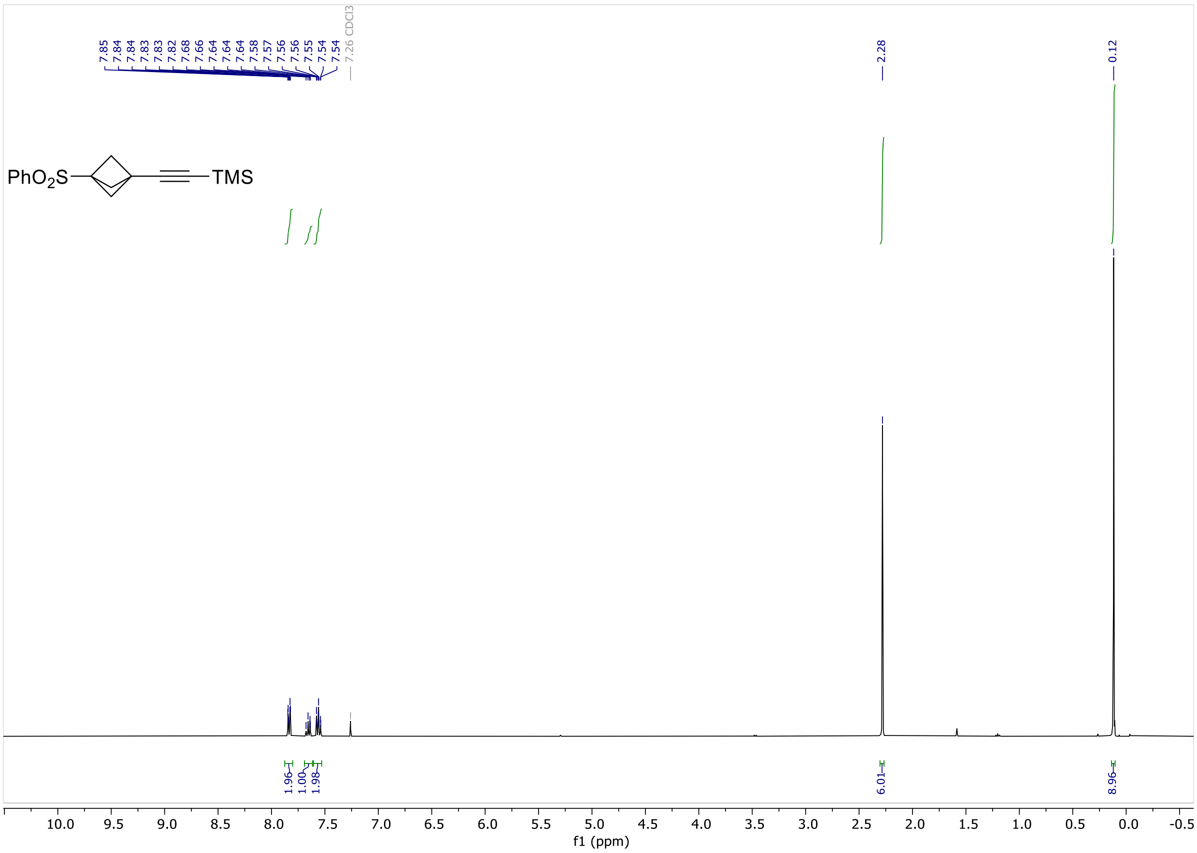

^{13}C NMR (101 MHz, $CDCl_3$) of compound **14**

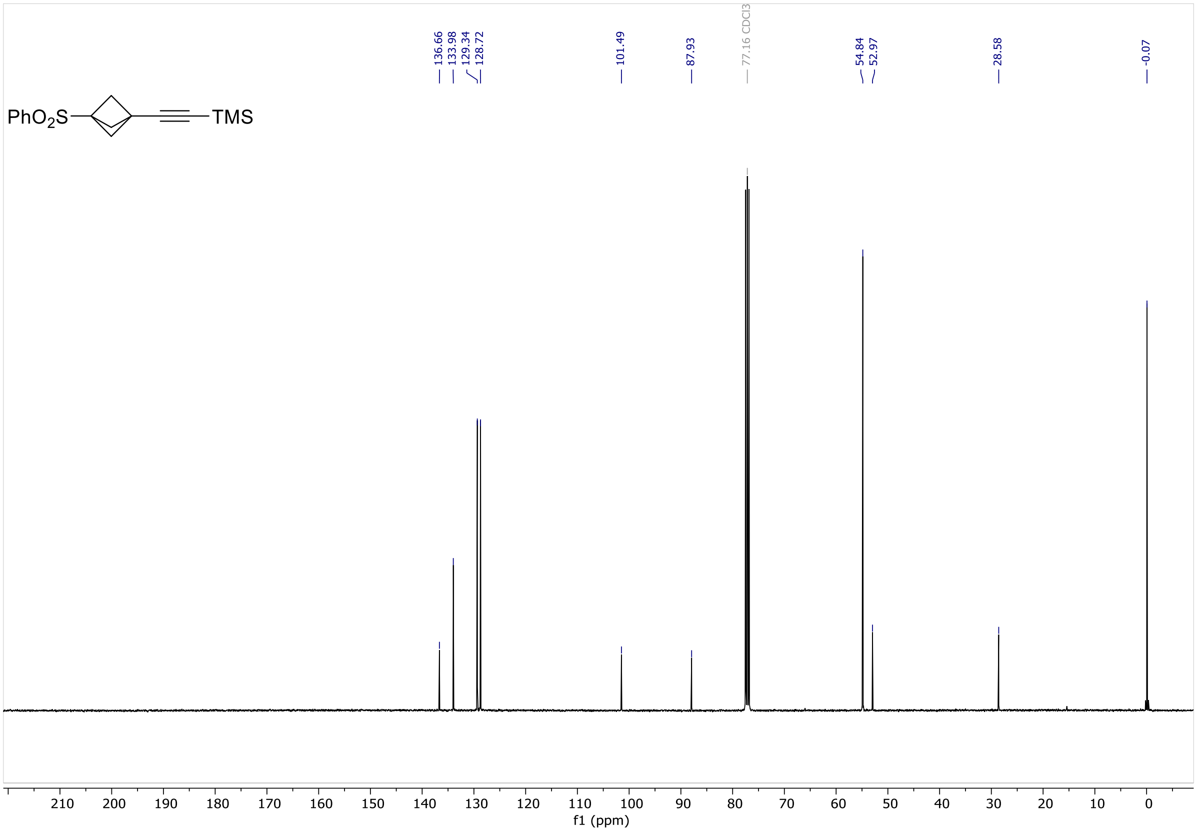

^{1}H NMR (400 MHz, $CDCl_3$) of **EBX.1**

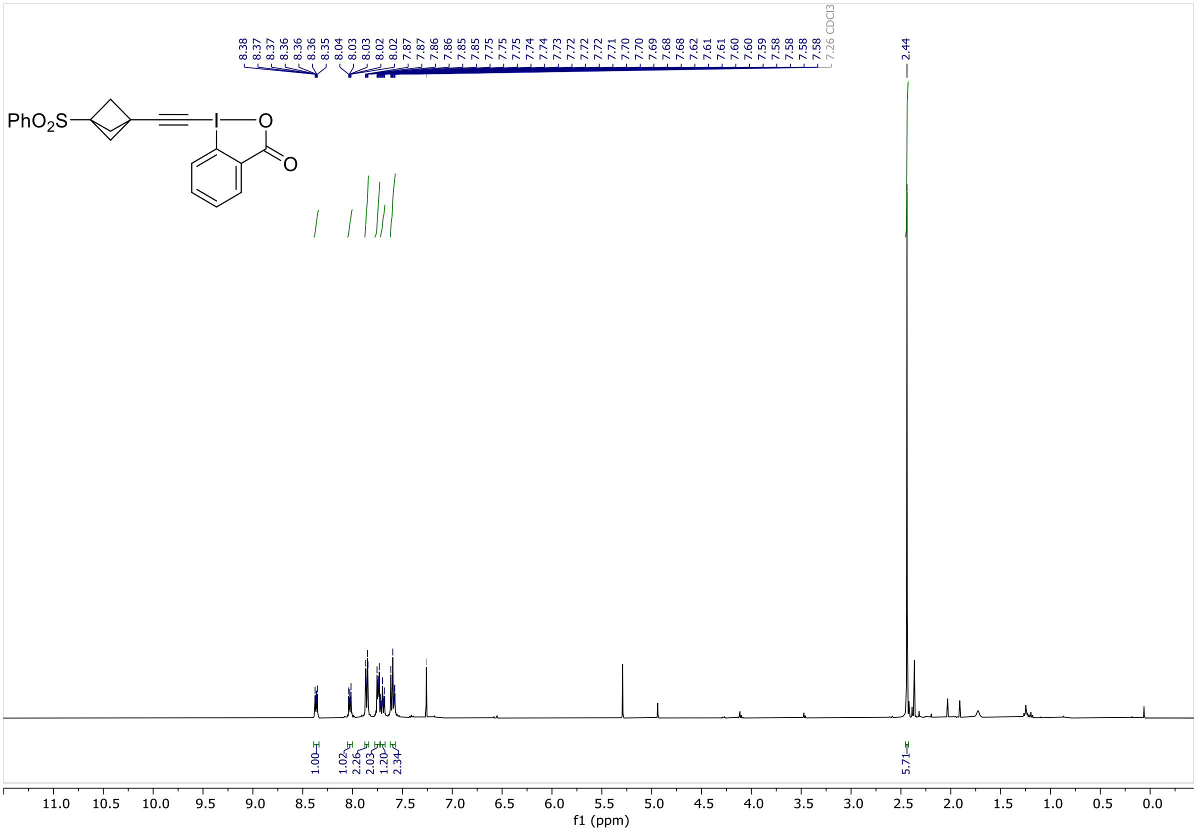

^{13}C NMR (101 MHz, $CDCl_3$) of **EBX.1**

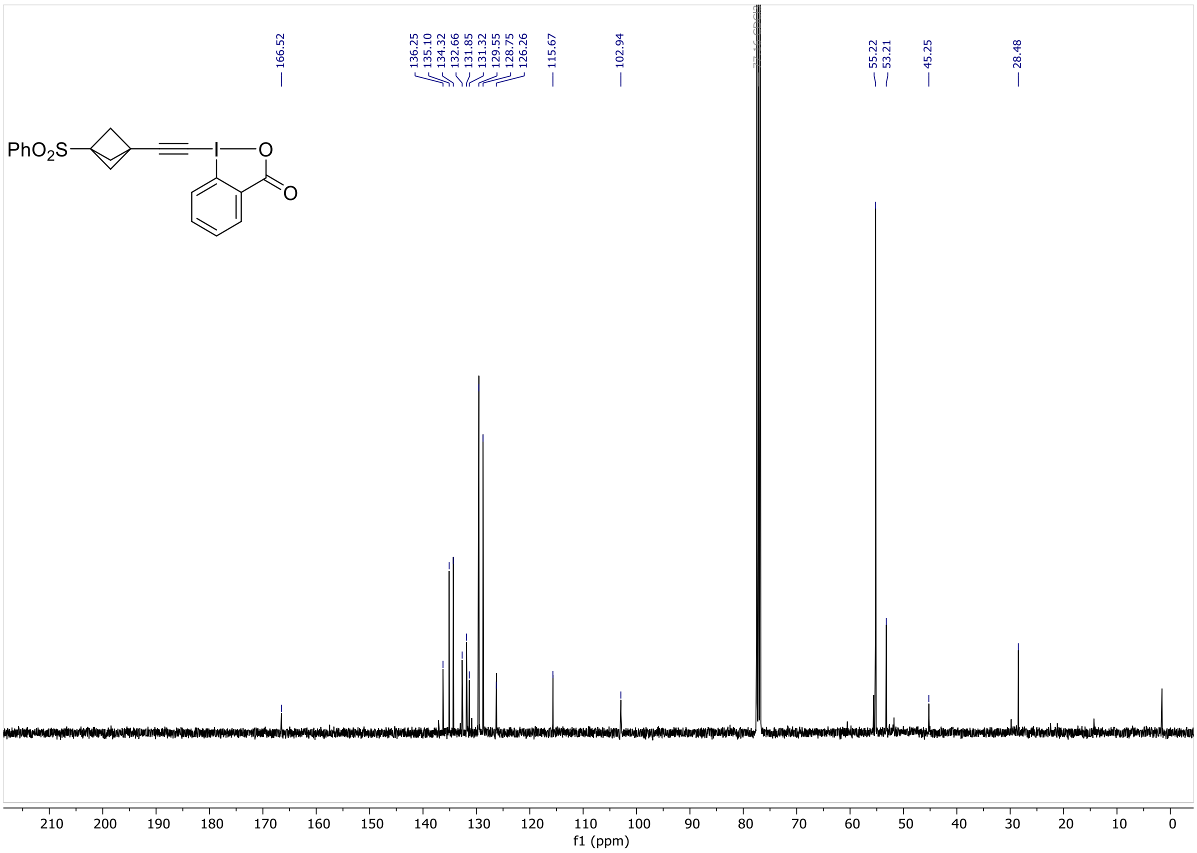

^{1}**H NMR** (400 MHz, $CDCl_3$) of **EBX.2**

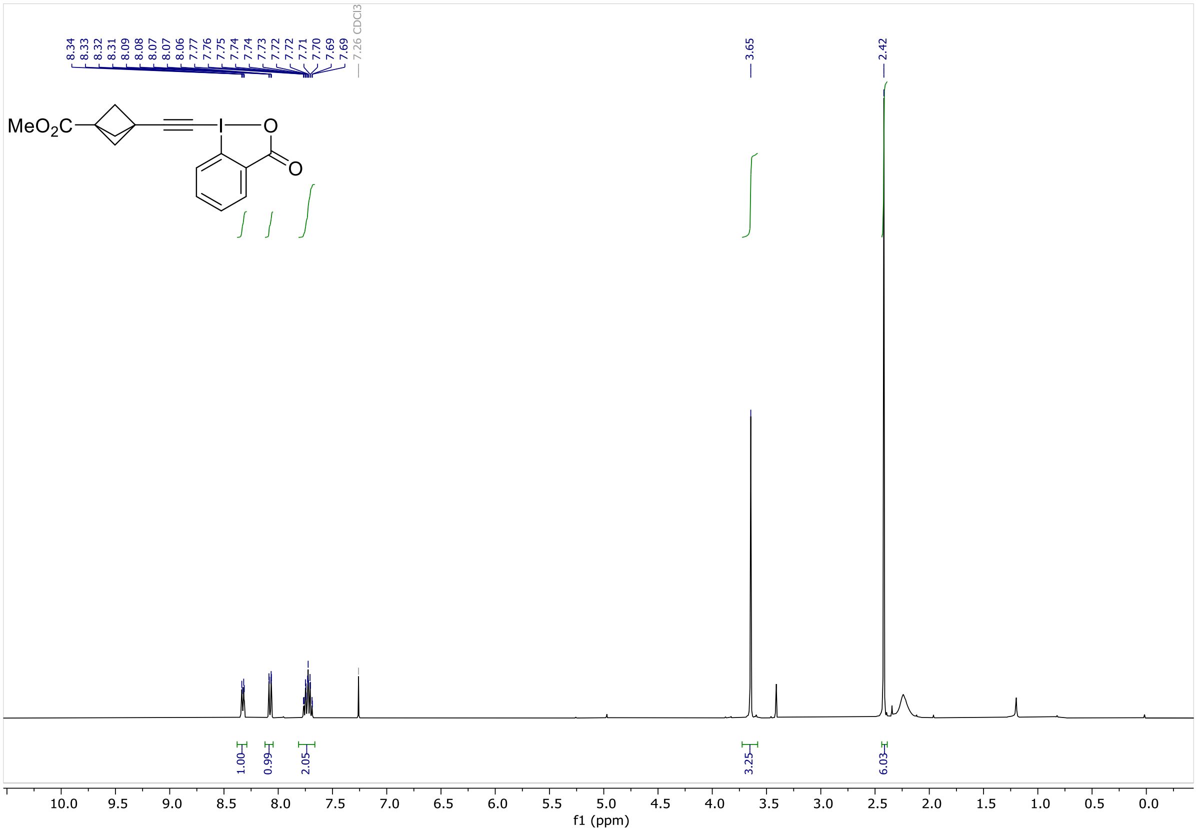

^{13}**C NMR** (101 MHz, $CDCl_3$) of **EBX.2**

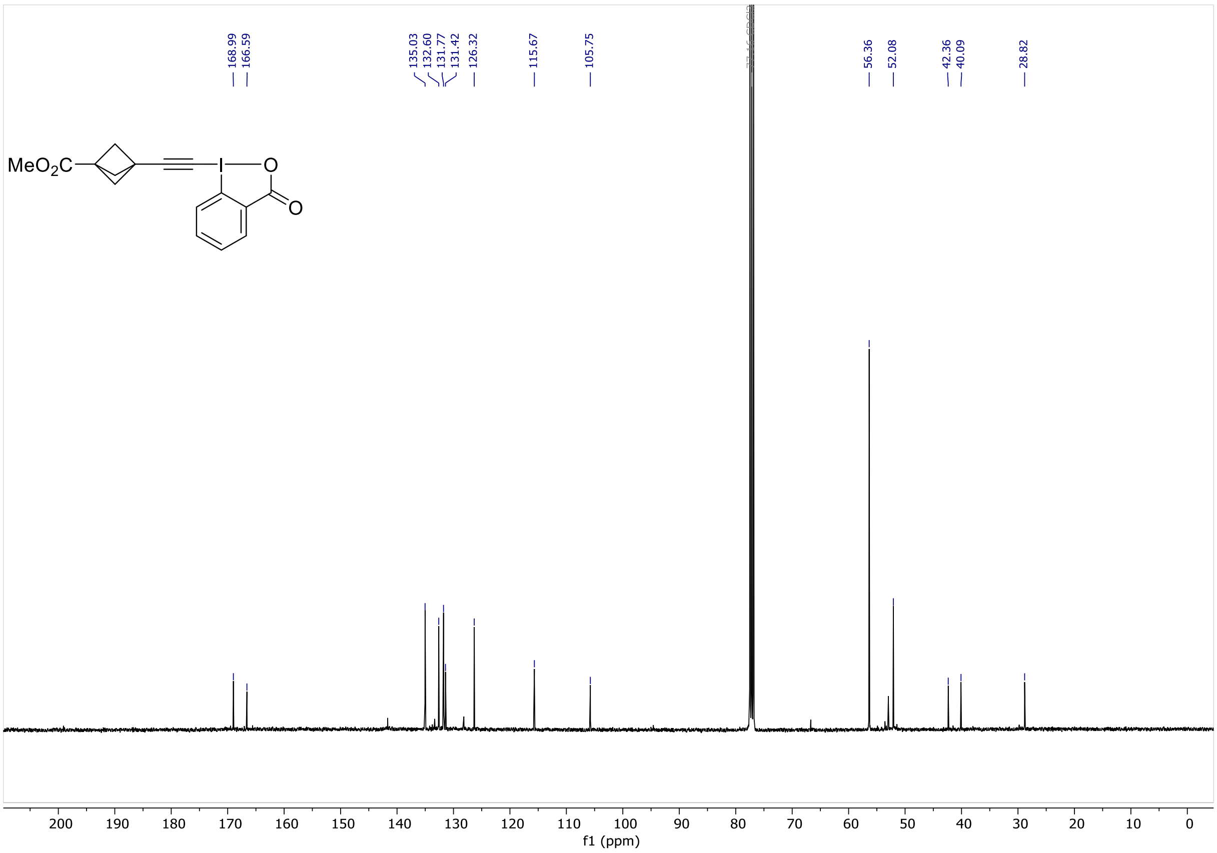

^{1}H NMR (400 MHz, $CDCl_3$) of compound **23**; it was not possible to separate out from some impurity

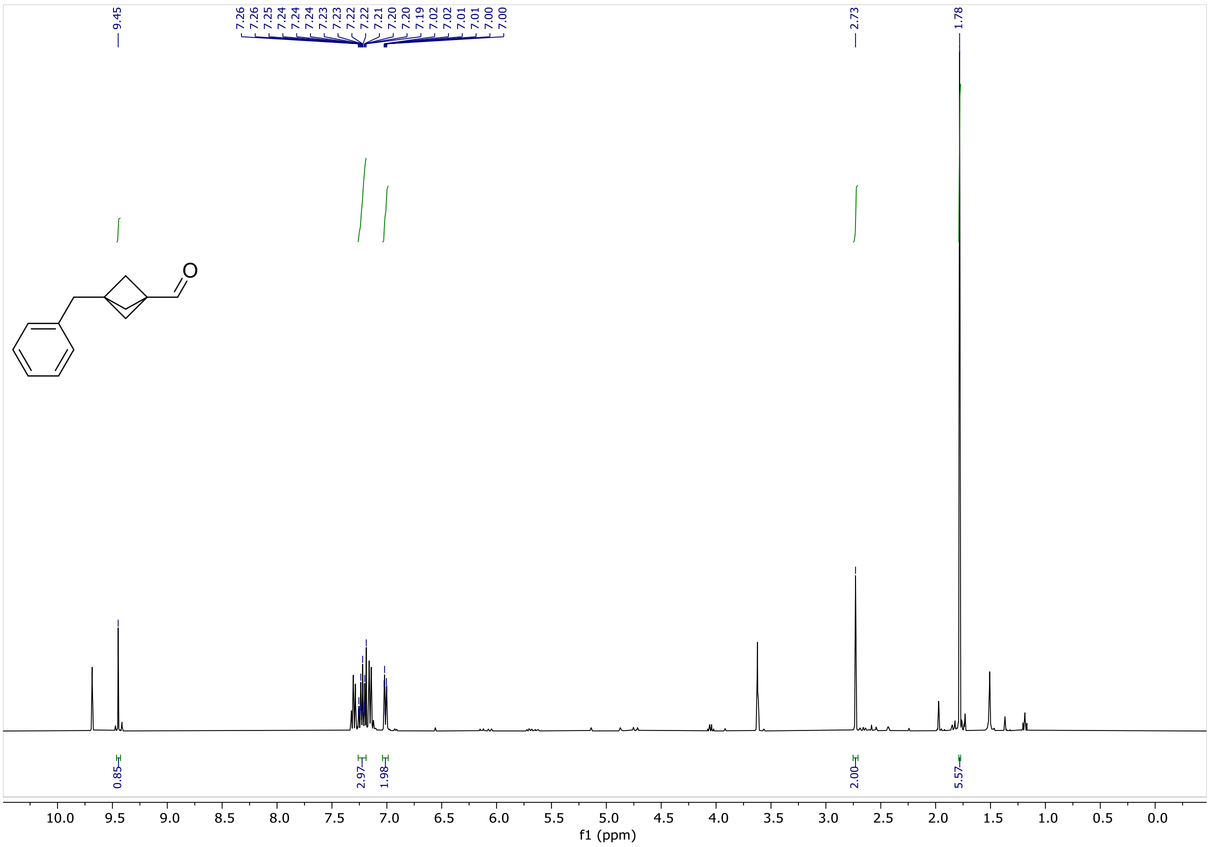

^{13}C NMR (101 MHz, $CDCl_3$) of compound **23**; it was not possible to separate out from some impurity

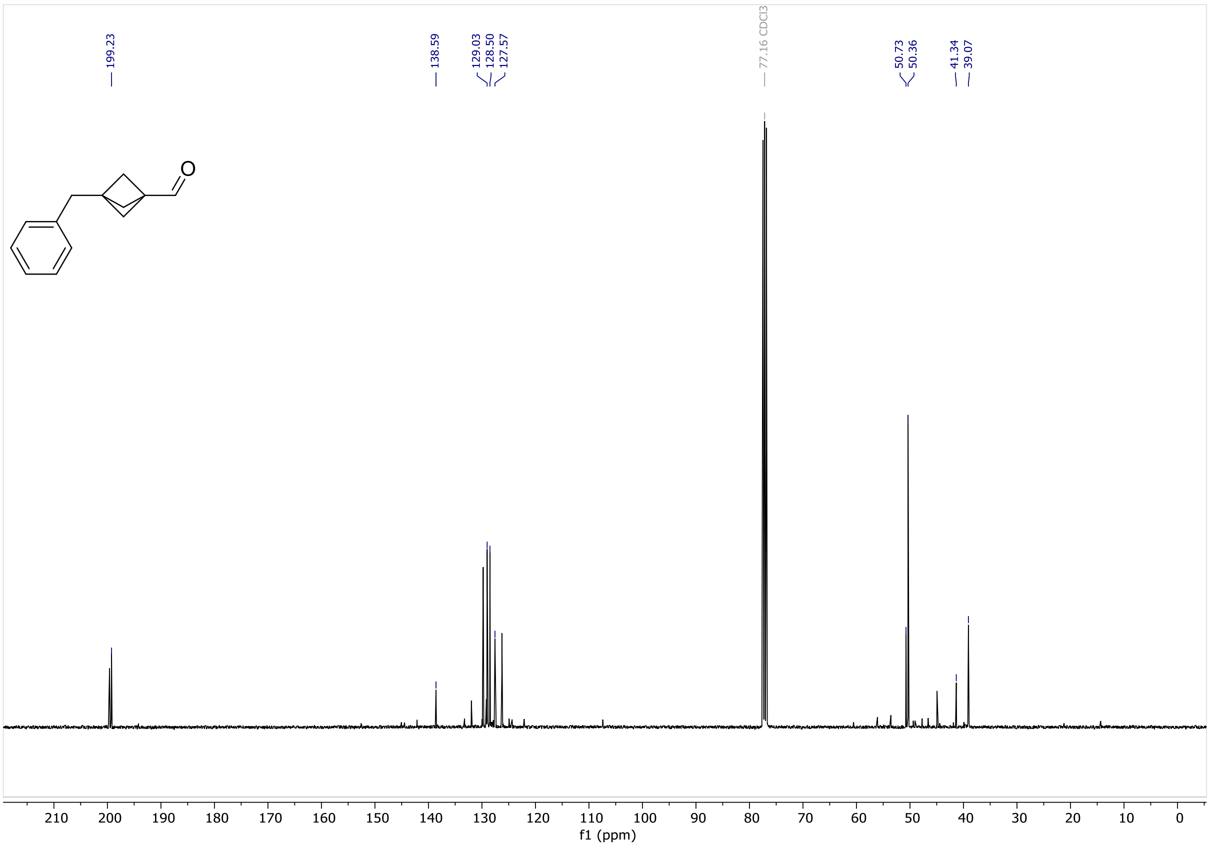

[1]**H NMR** (400 MHz, $CDCl_3$) of compound **25**; it was not possible to separate out from some impurity

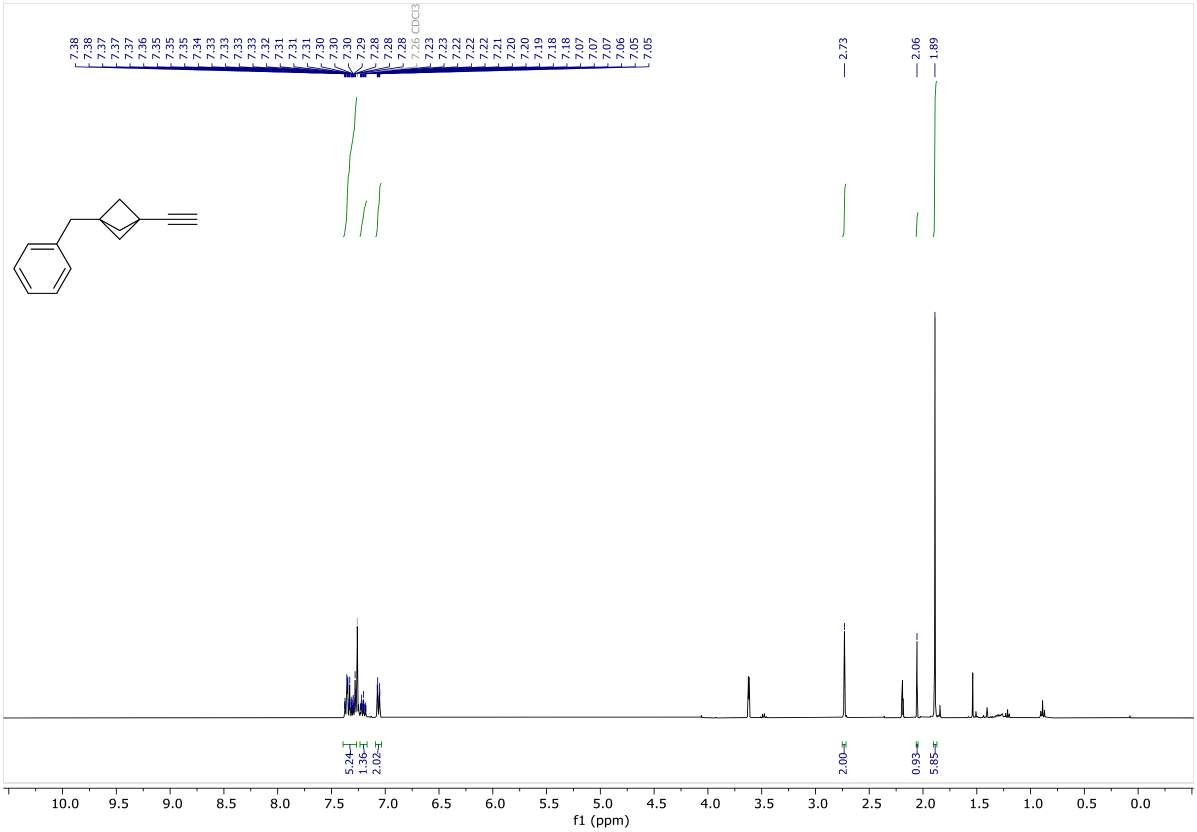

[13]**C NMR** (101 MHz, $CDCl_3$) of compound **25**; it was not possible to separate out from some impurity

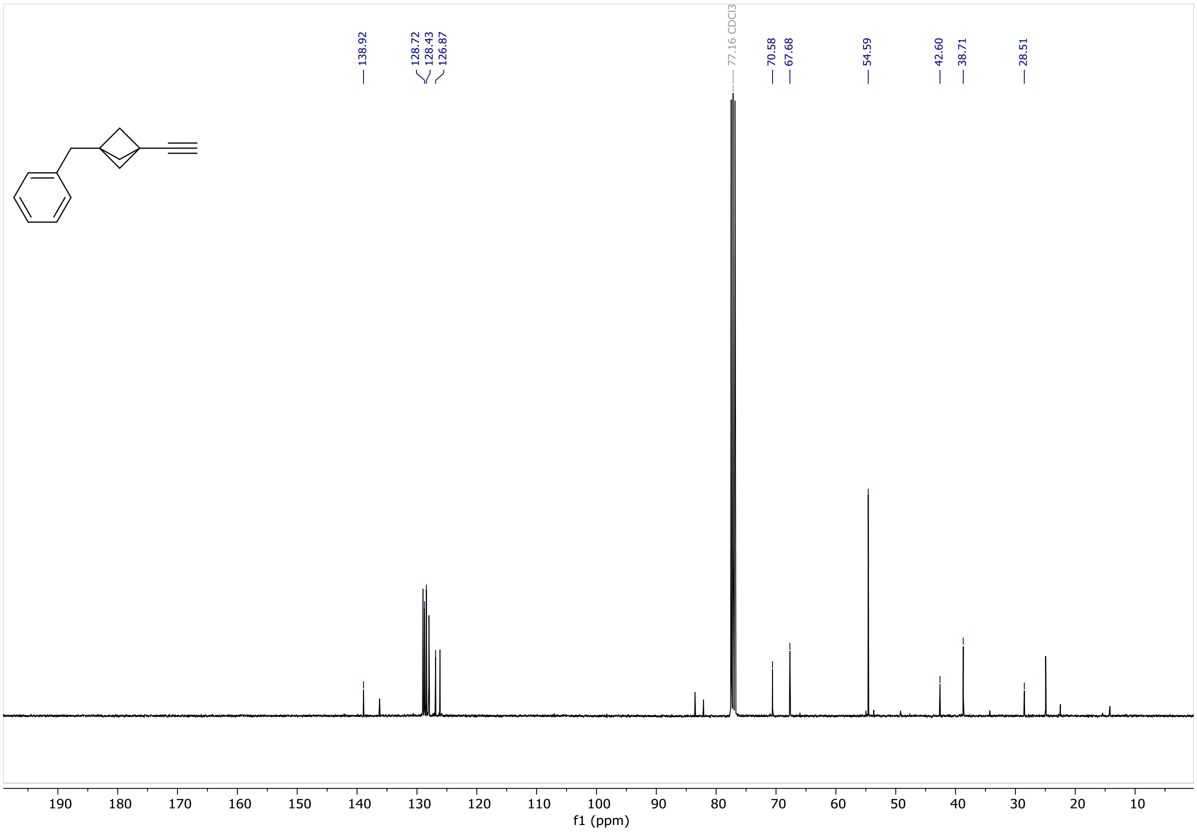

^{1}H NMR (400 MHz, $CDCl_3$) of compound **26**

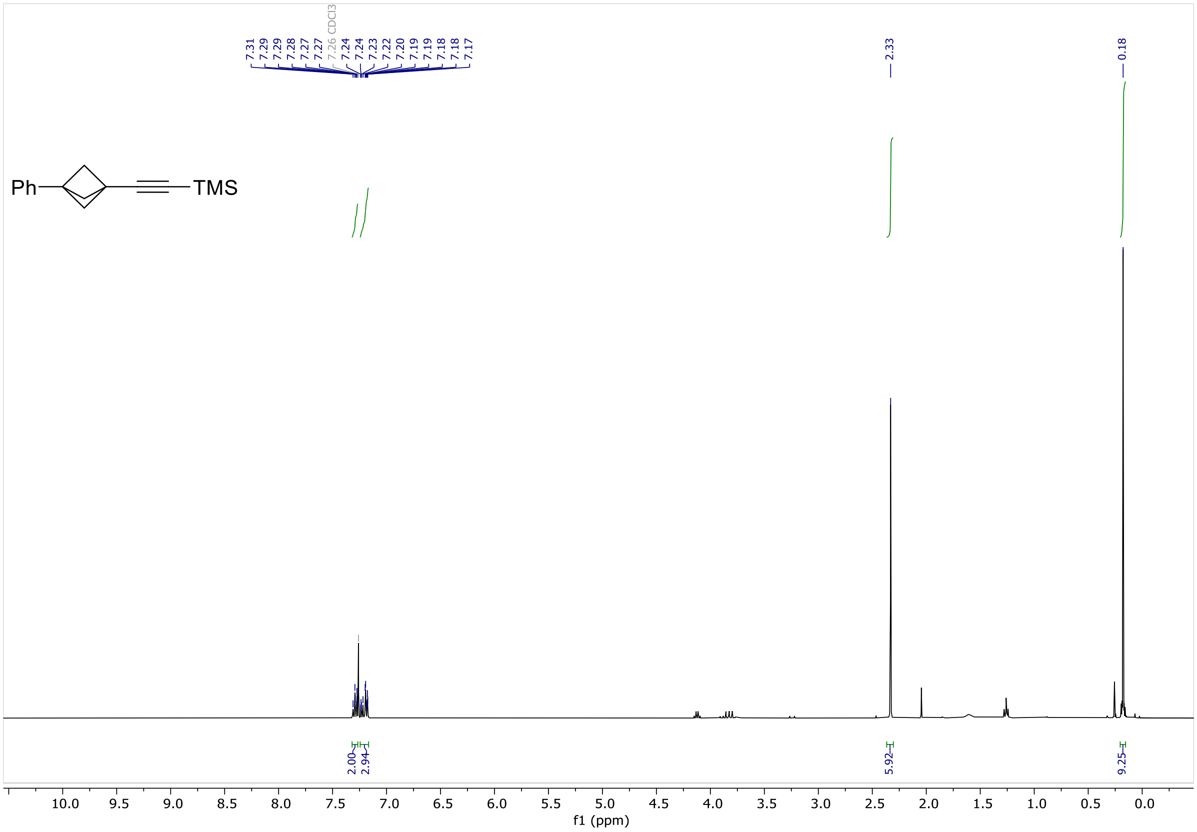

^{13}C NMR (101 MHz, $CDCl_3$) of compound **26**

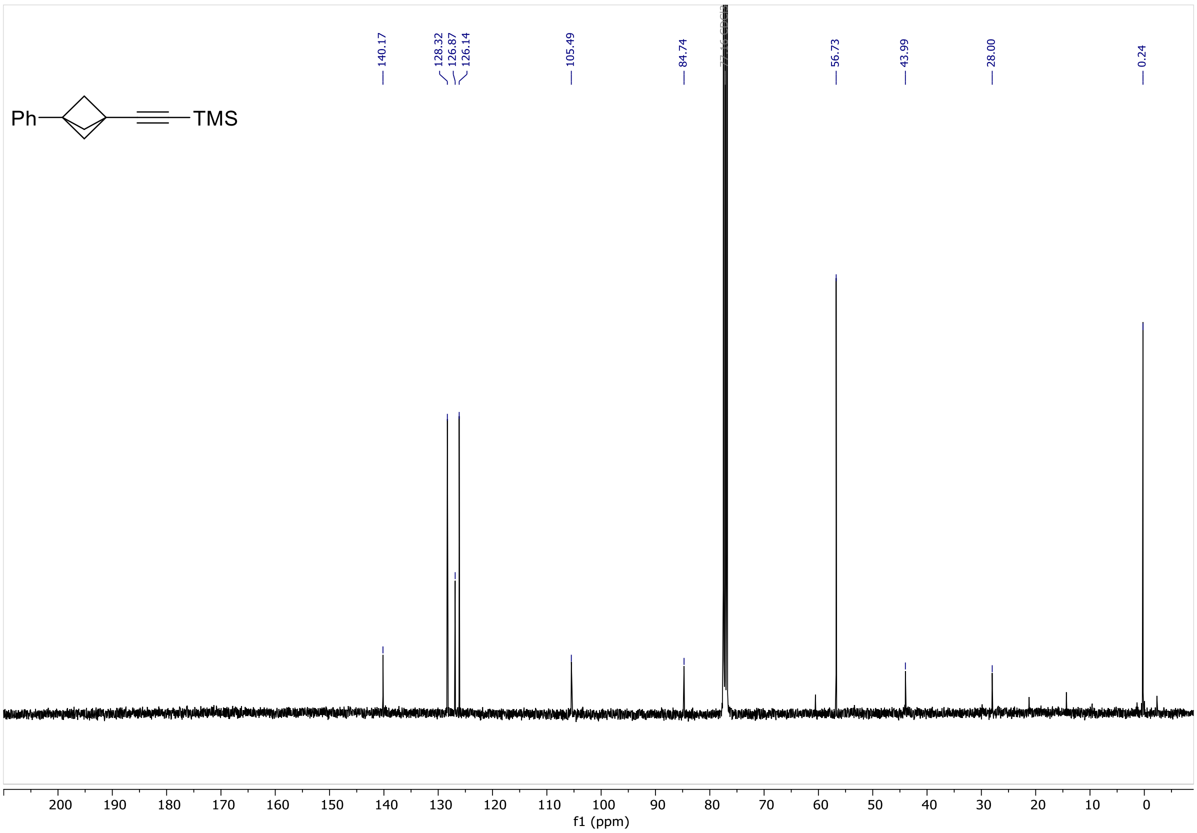

^{1}H NMR (400 MHz, $CDCl_3$) of compound **27**

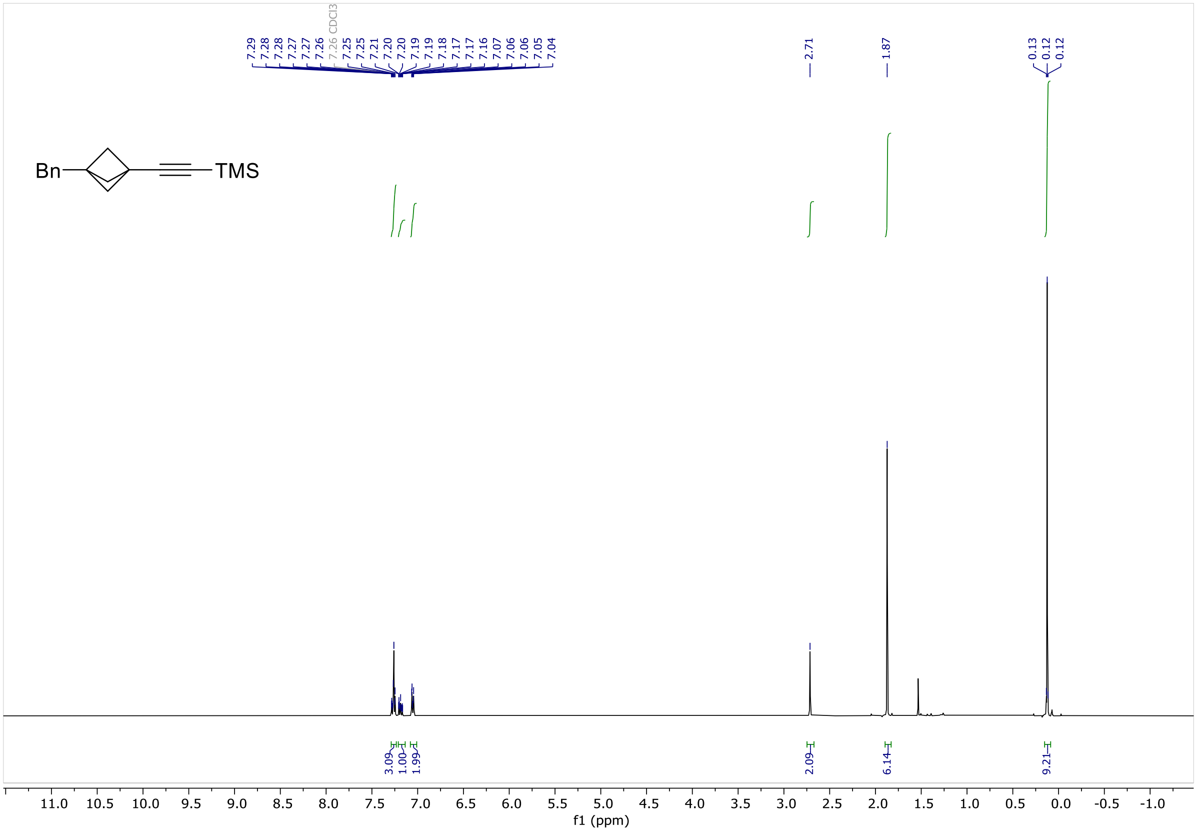

^{13}C NMR (101 MHz, $CDCl_3$) of compound **27**

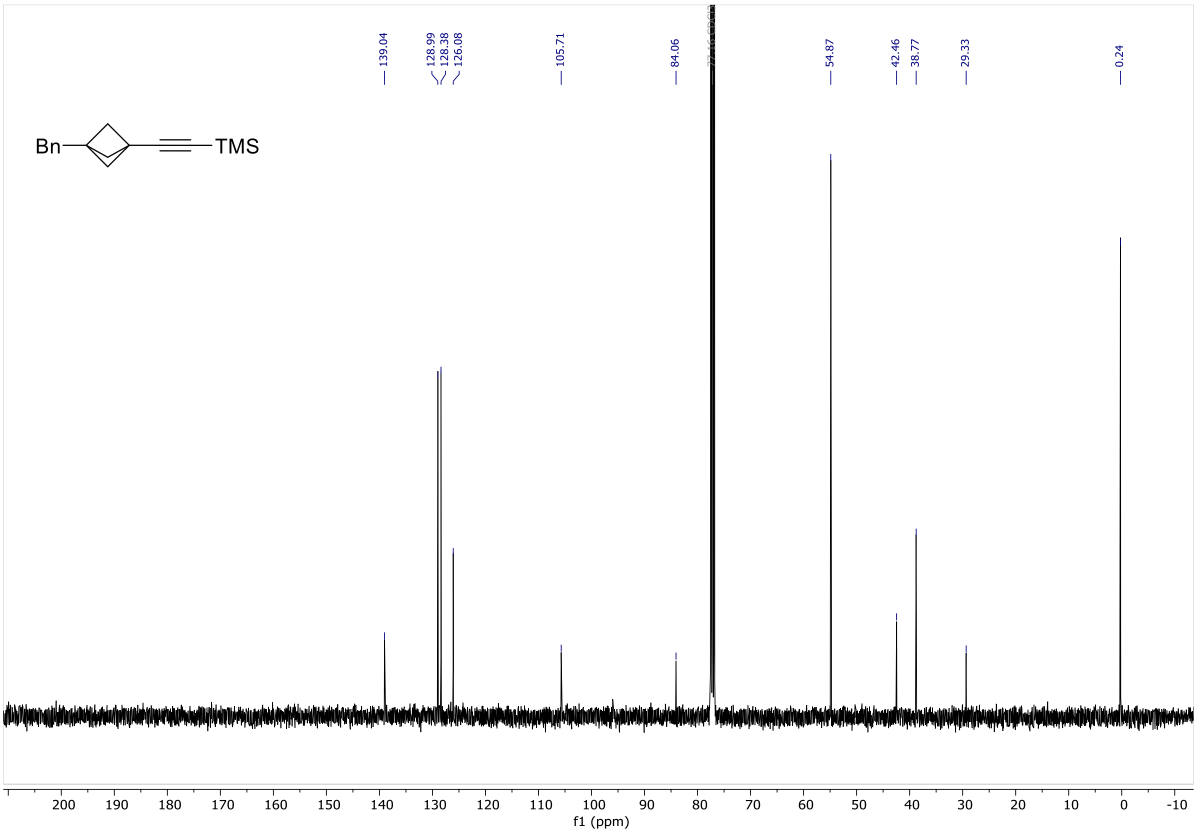

^{1}H NMR (400 MHz, $CDCl_3$) of **EBX.3**

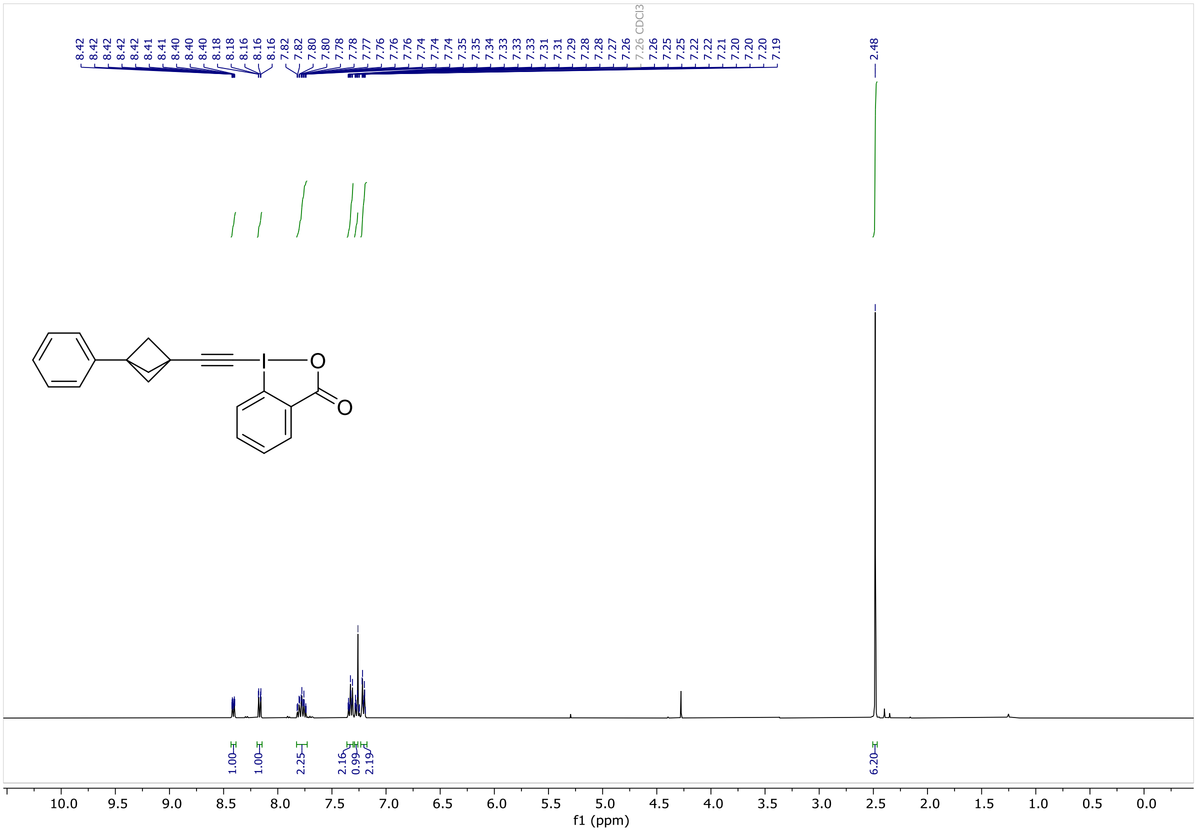

^{13}C NMR (101 MHz, $CDCl_3$) of **EBX.3**

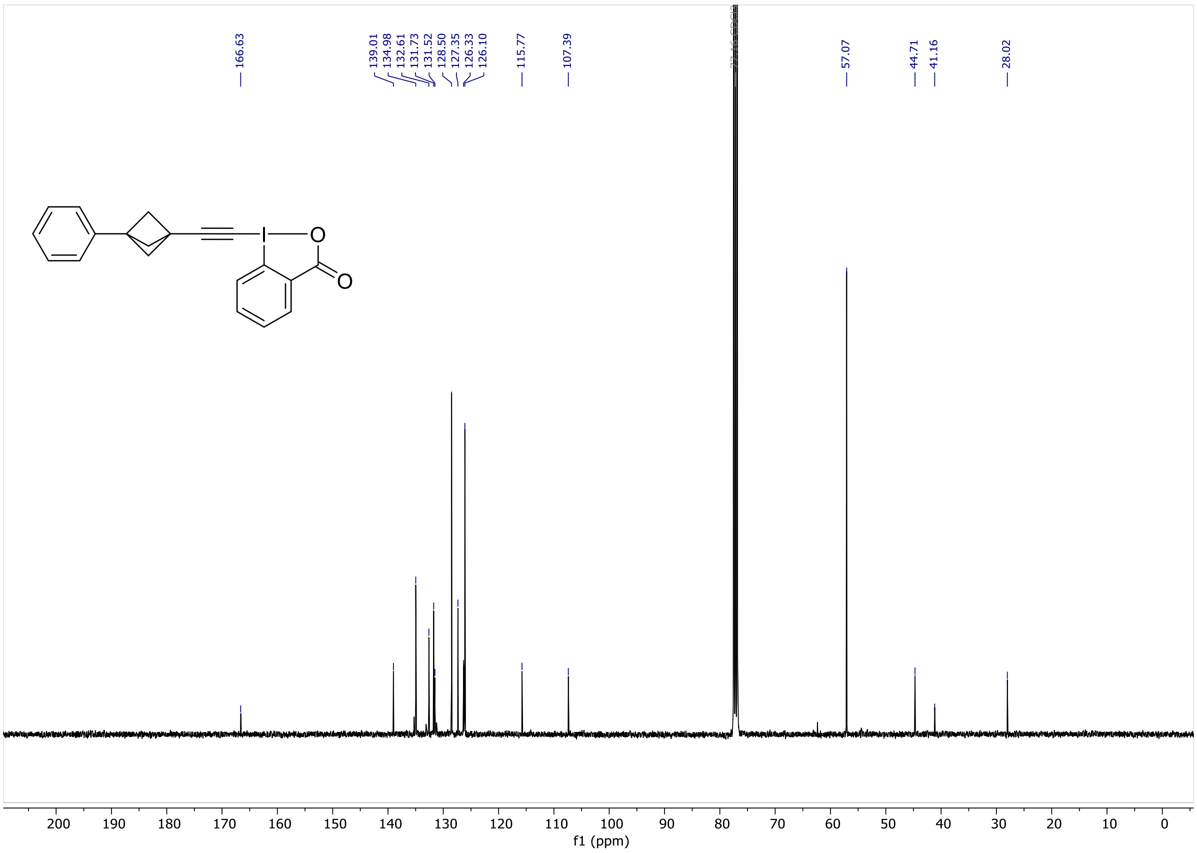

^{1}H NMR (400 MHz, $CDCl_3$) of **EBX.4**

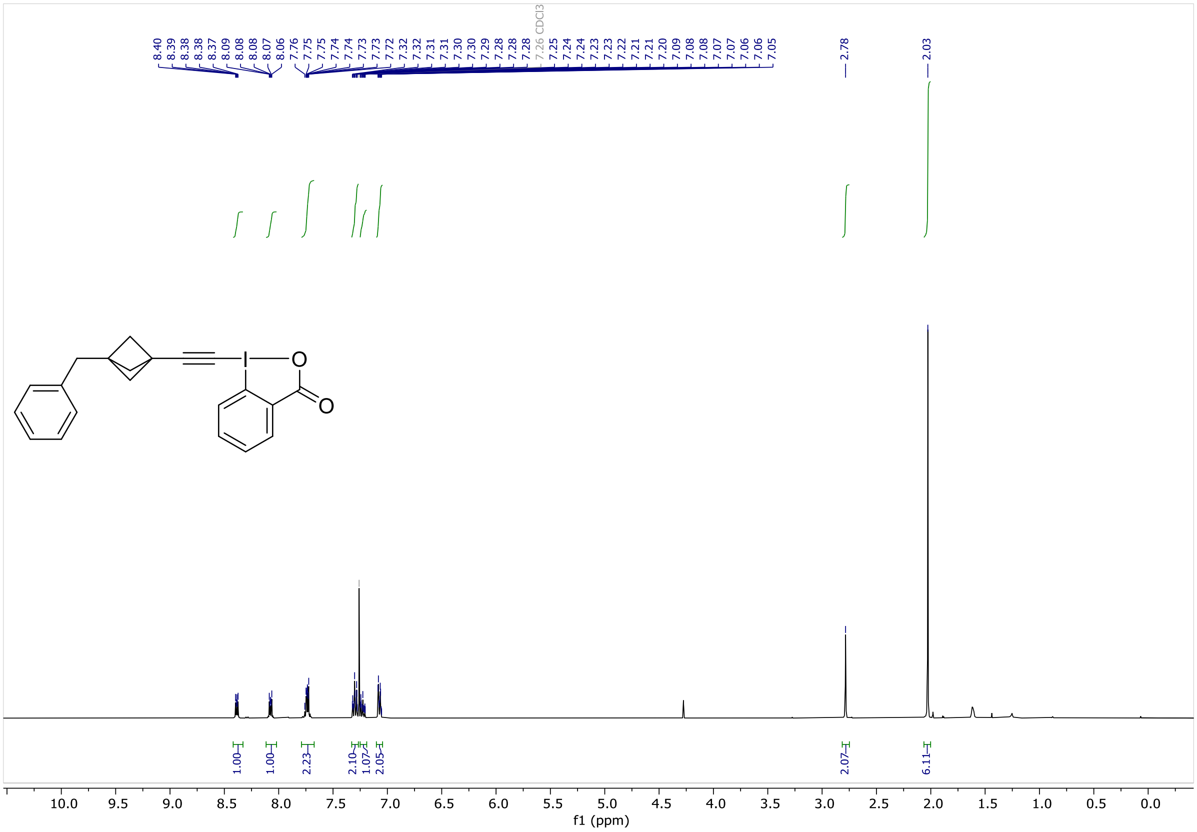

^{13}C NMR (101 MHz, $CDCl_3$) of **EBX.4**

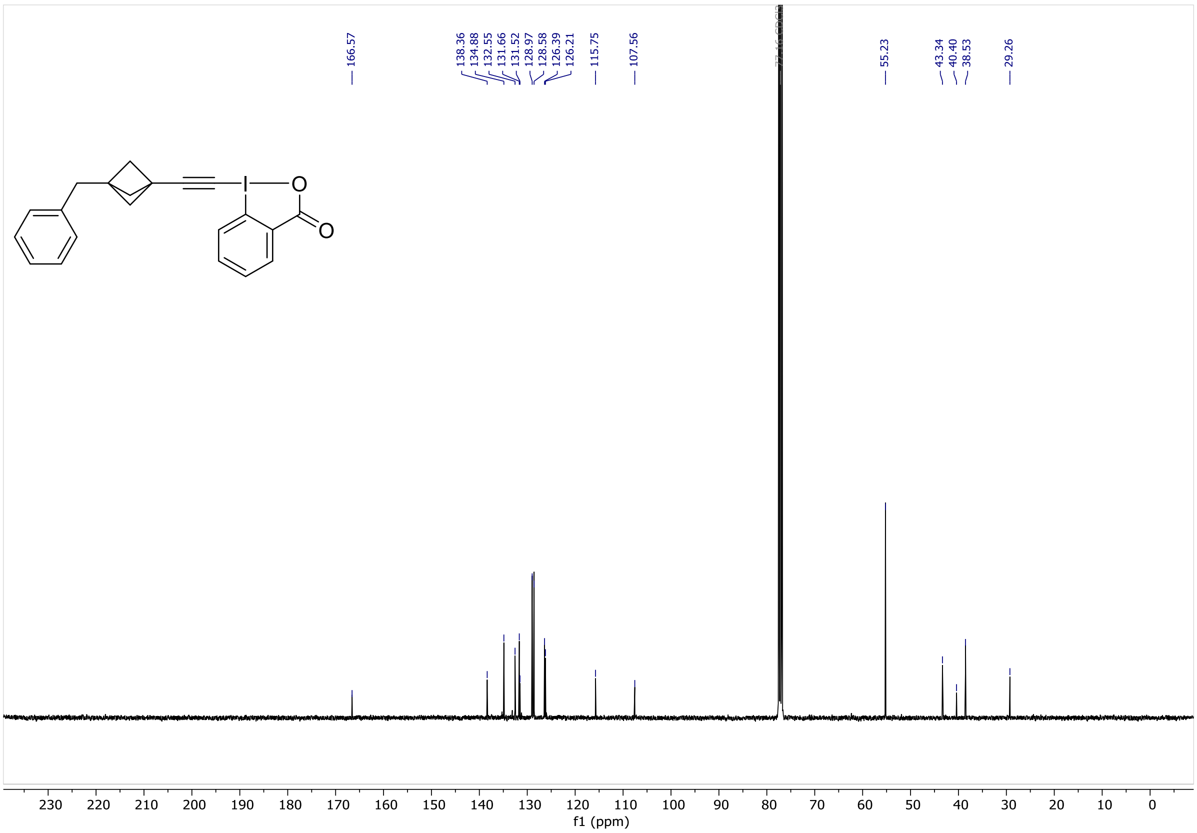

^{1}H NMR (400 MHz, $CDCl_3$) of compound **29a**

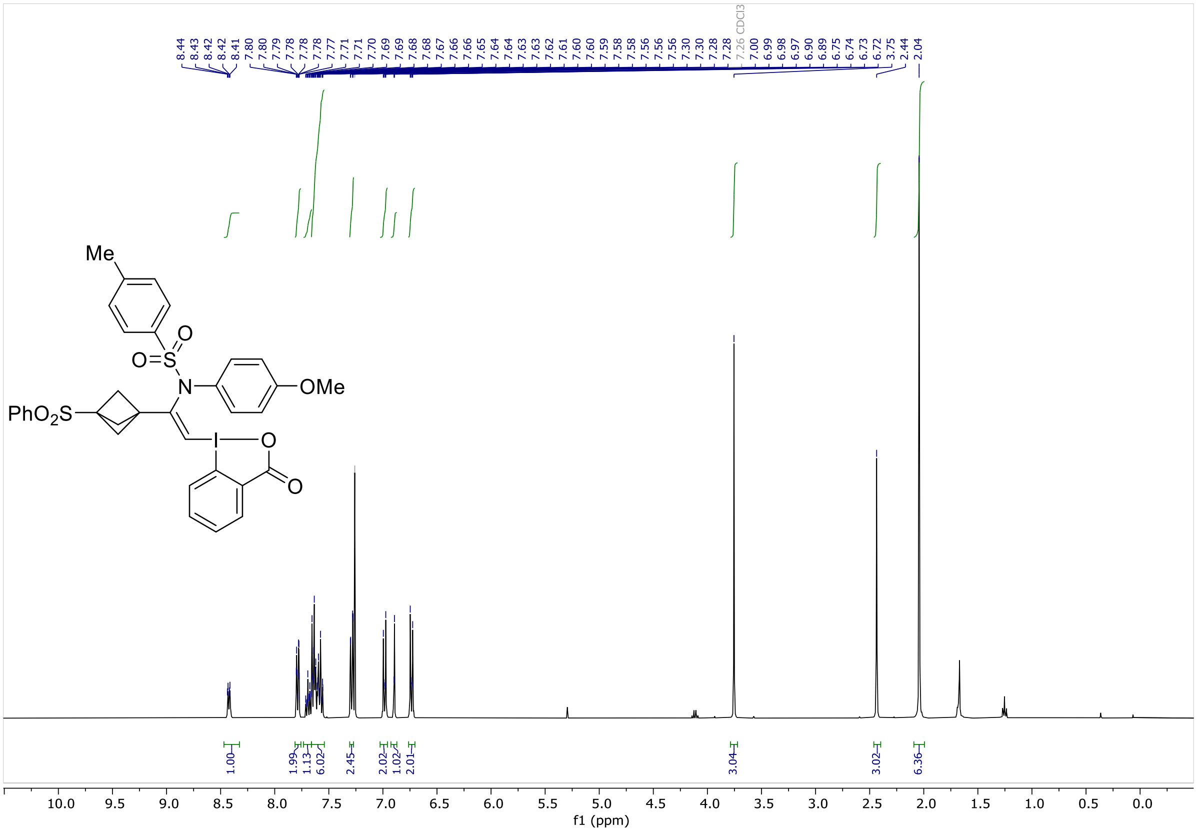

^{13}C NMR (101 MHz, $CDCl_3$) of compound **29a**

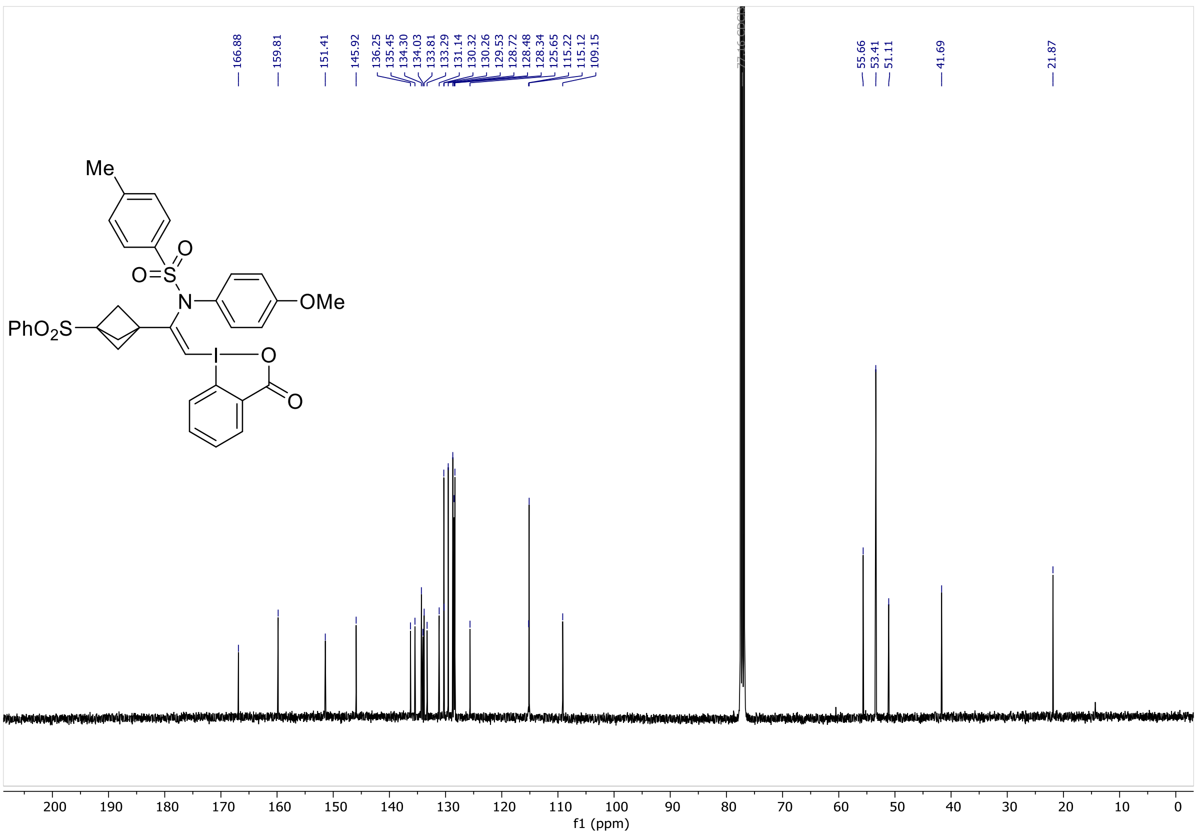

^{1}H NMR (400 MHz, $CDCl_3$) of compound **29b**

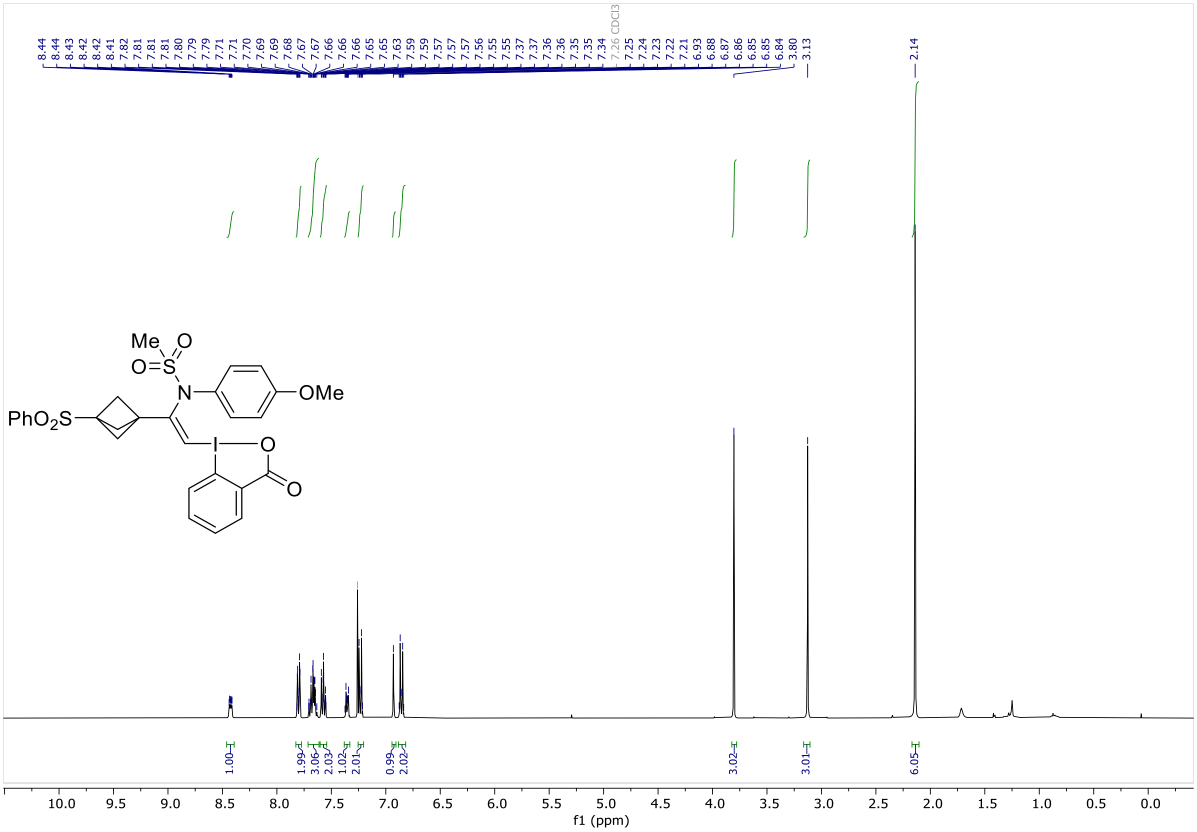

^{13}C NMR (101 MHz, $CDCl_3$) of compound **29b**

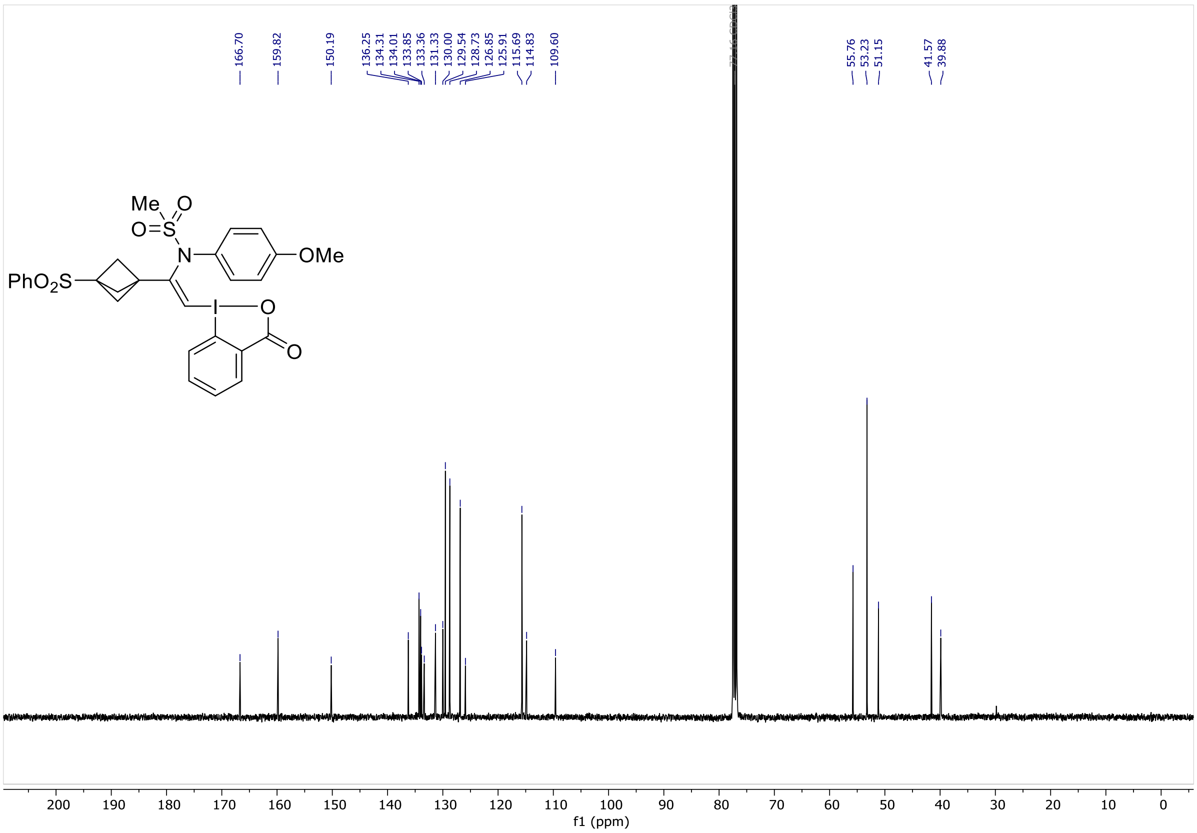

^{1}H NMR (400 MHz, $CDCl_3$) of compound **29c**

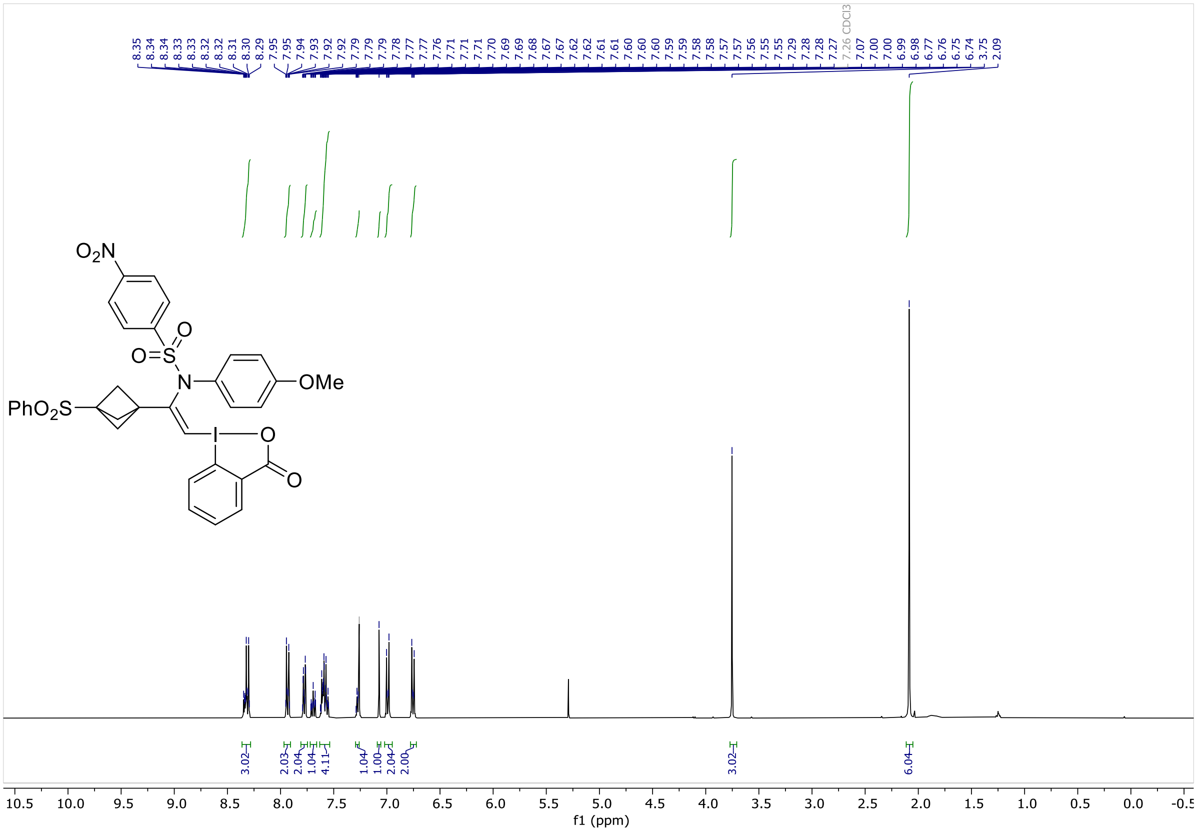

^{13}C NMR (101 MHz, $CDCl_3$) of compound **29c**

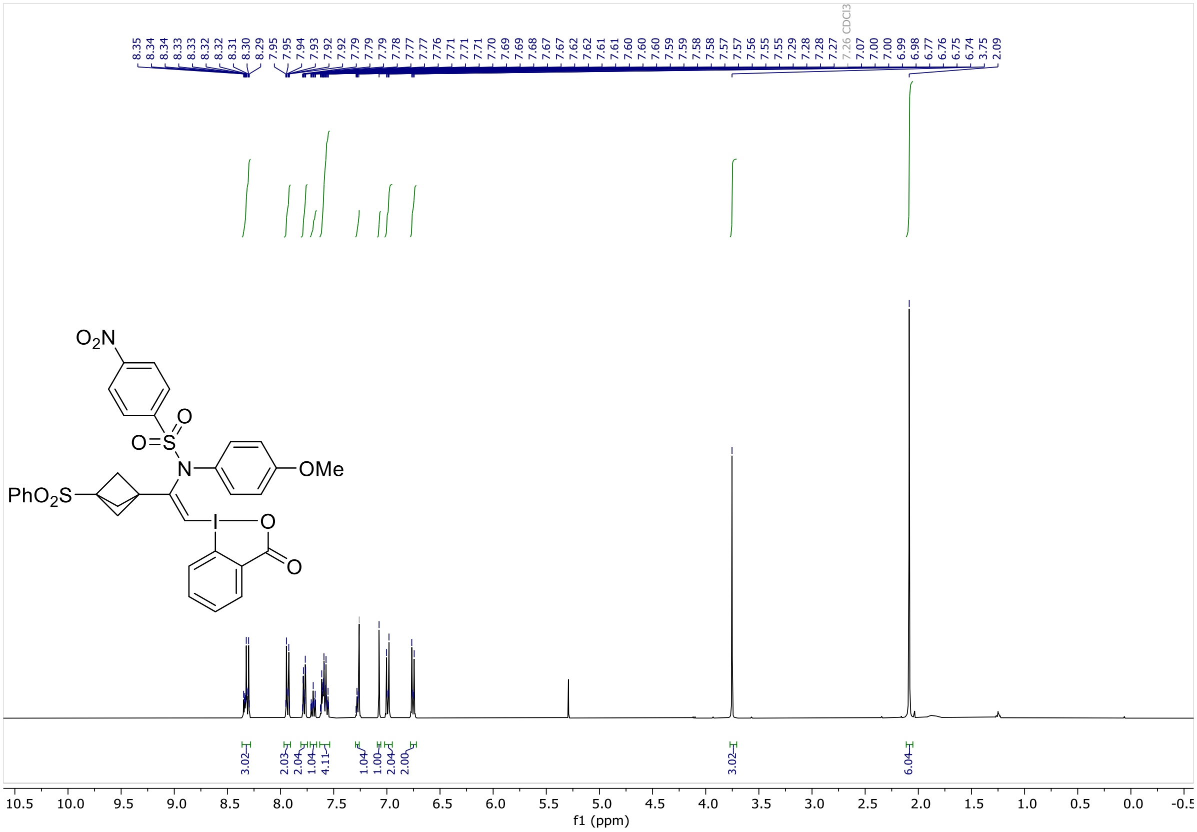

^{1}H NMR (400 MHz, $CDCl_3$) of compound **29d**

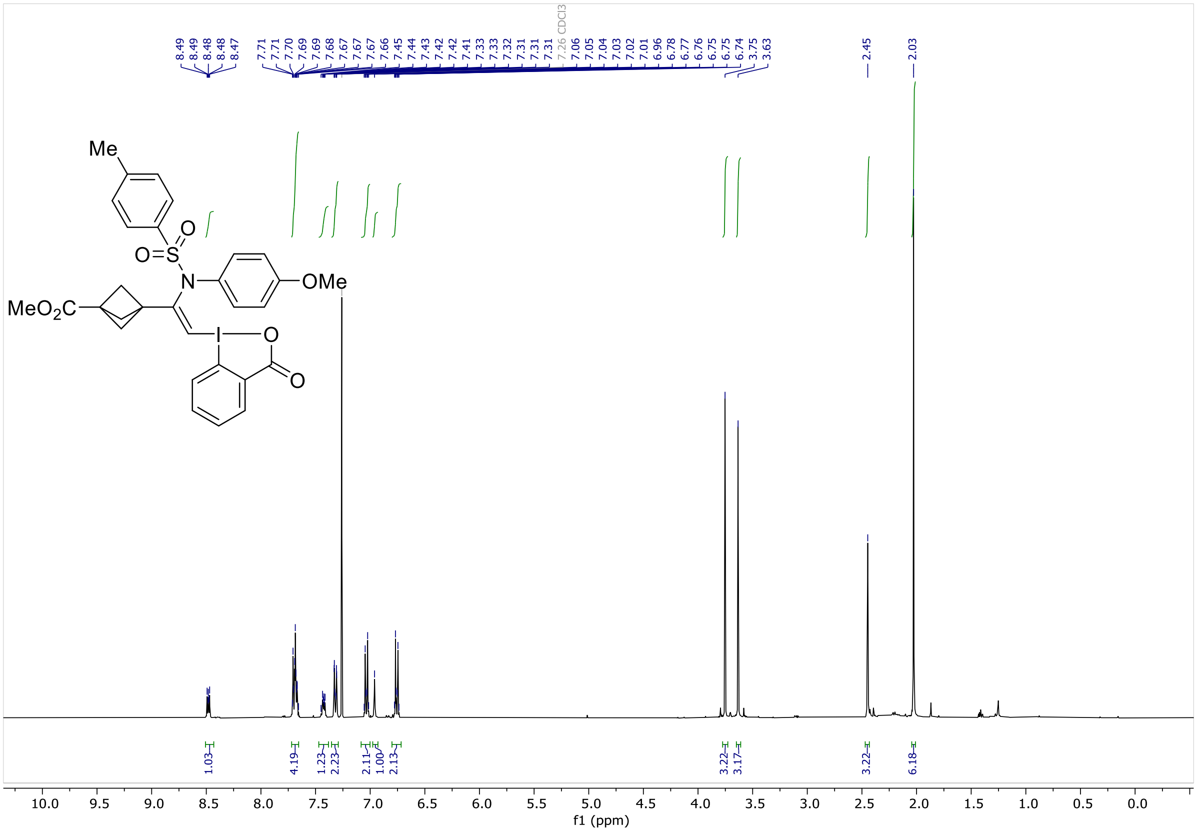

^{13}C NMR (101 MHz, $CDCl_3$) of compound **29d**

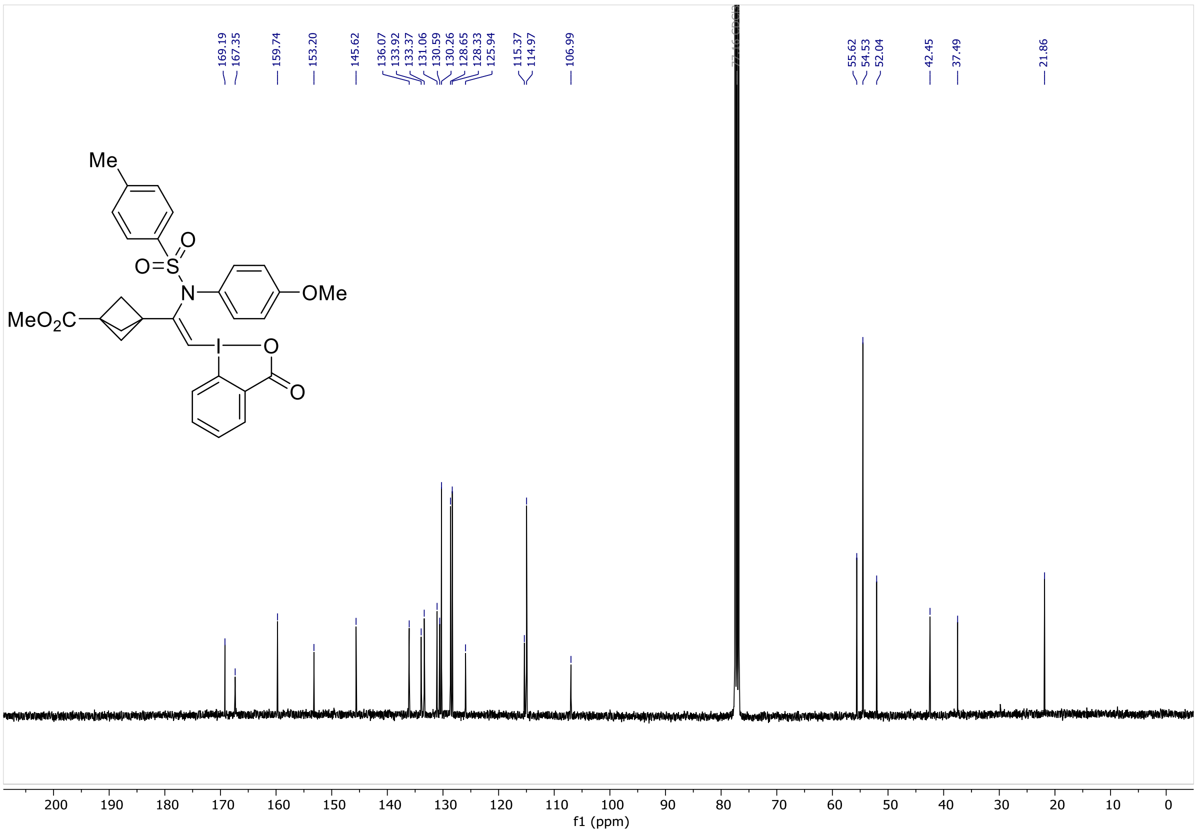

^{1}H NMR (400 MHz, $CDCl_3$) of compound **29e**

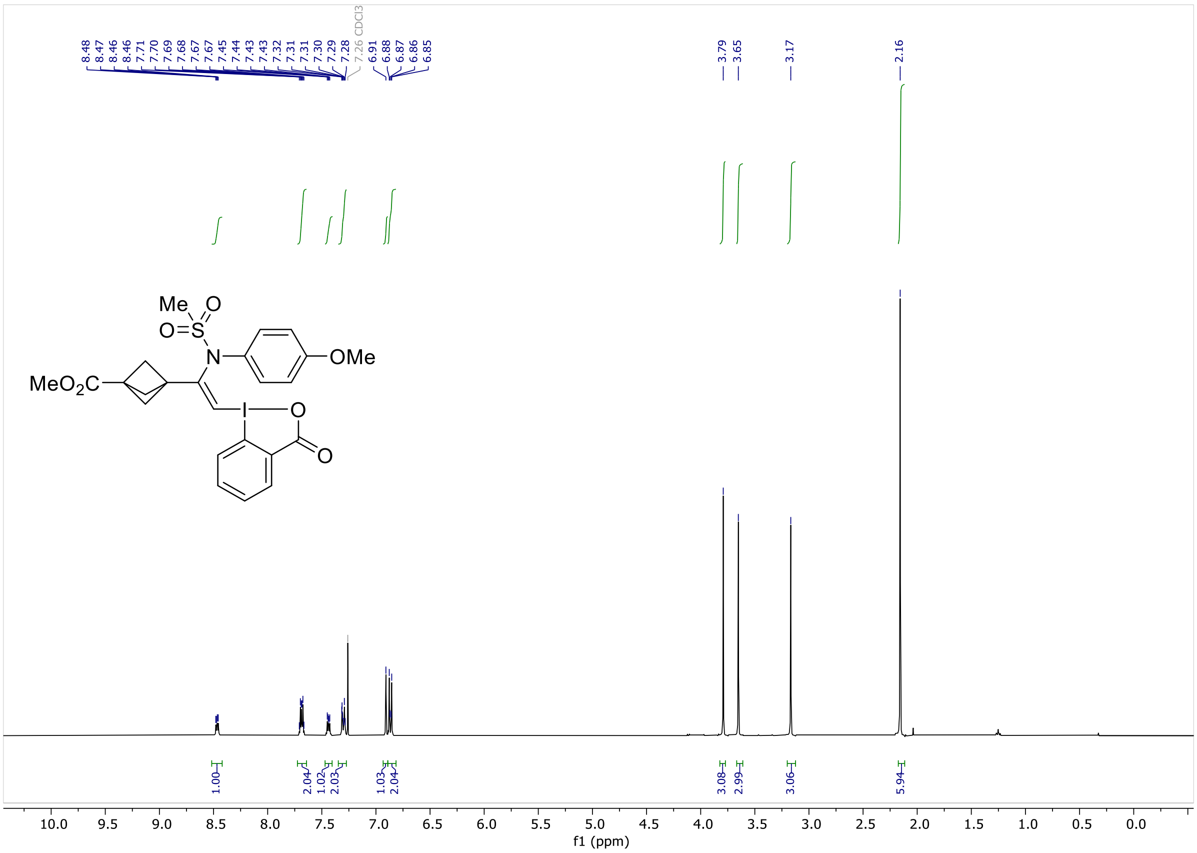

^{13}C NMR (101 MHz, $CDCl_3$) of compound **29e**

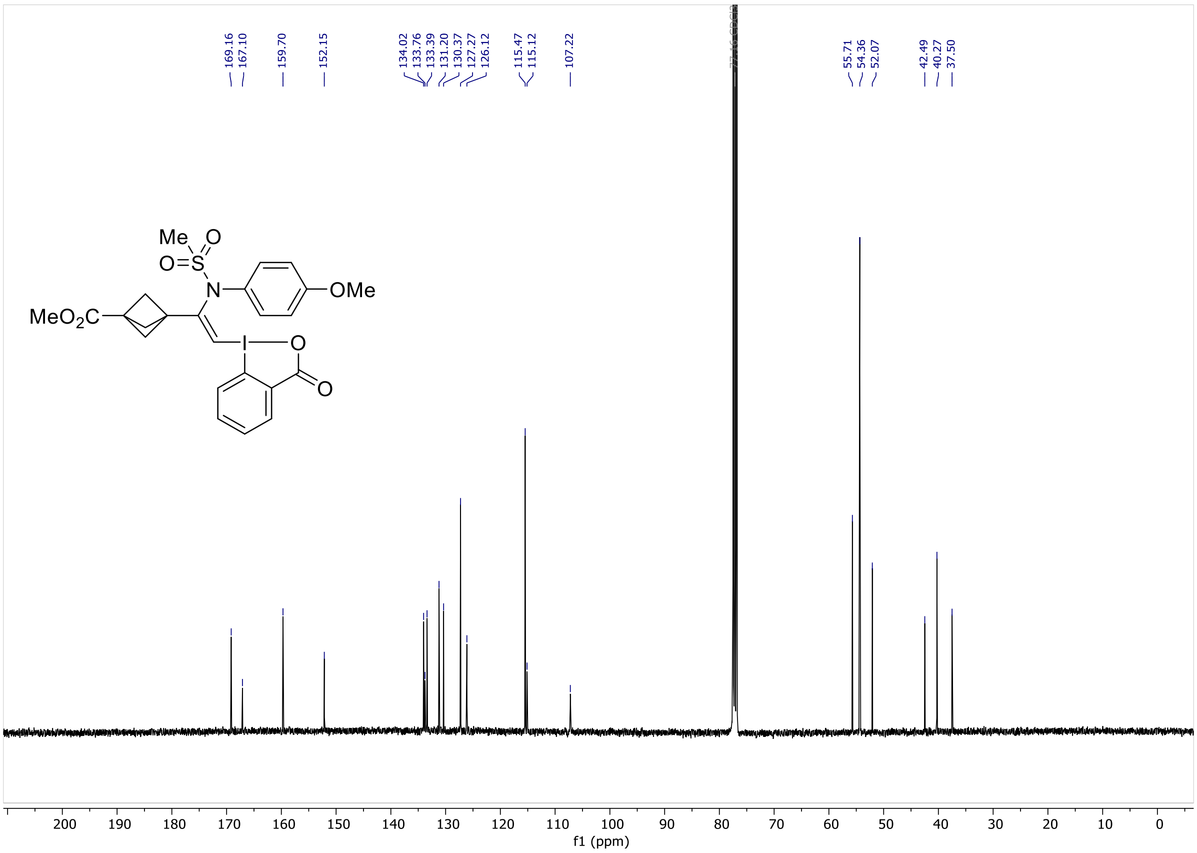

^{1}H NMR (400 MHz, $CDCl_3$) of compound **29f**

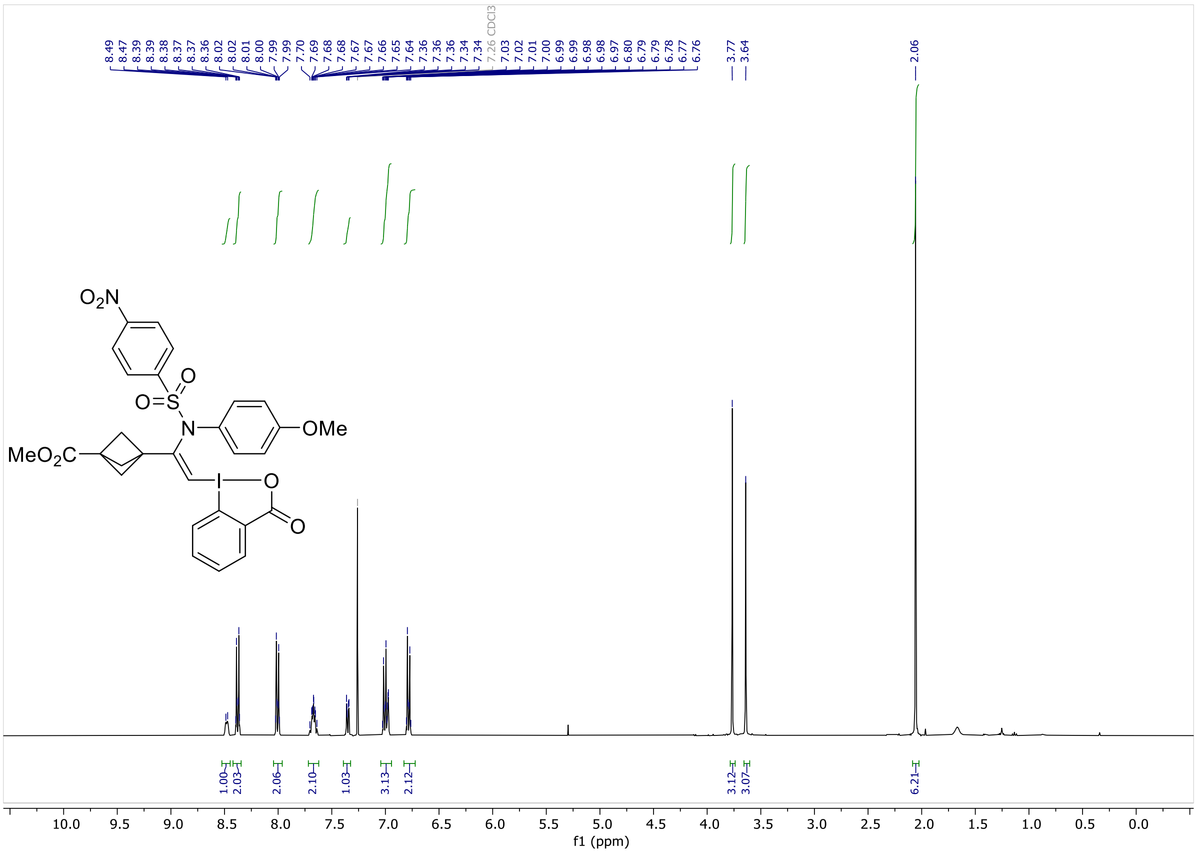

^{13}C NMR (101 MHz, $CDCl_3$) of compound **29f**

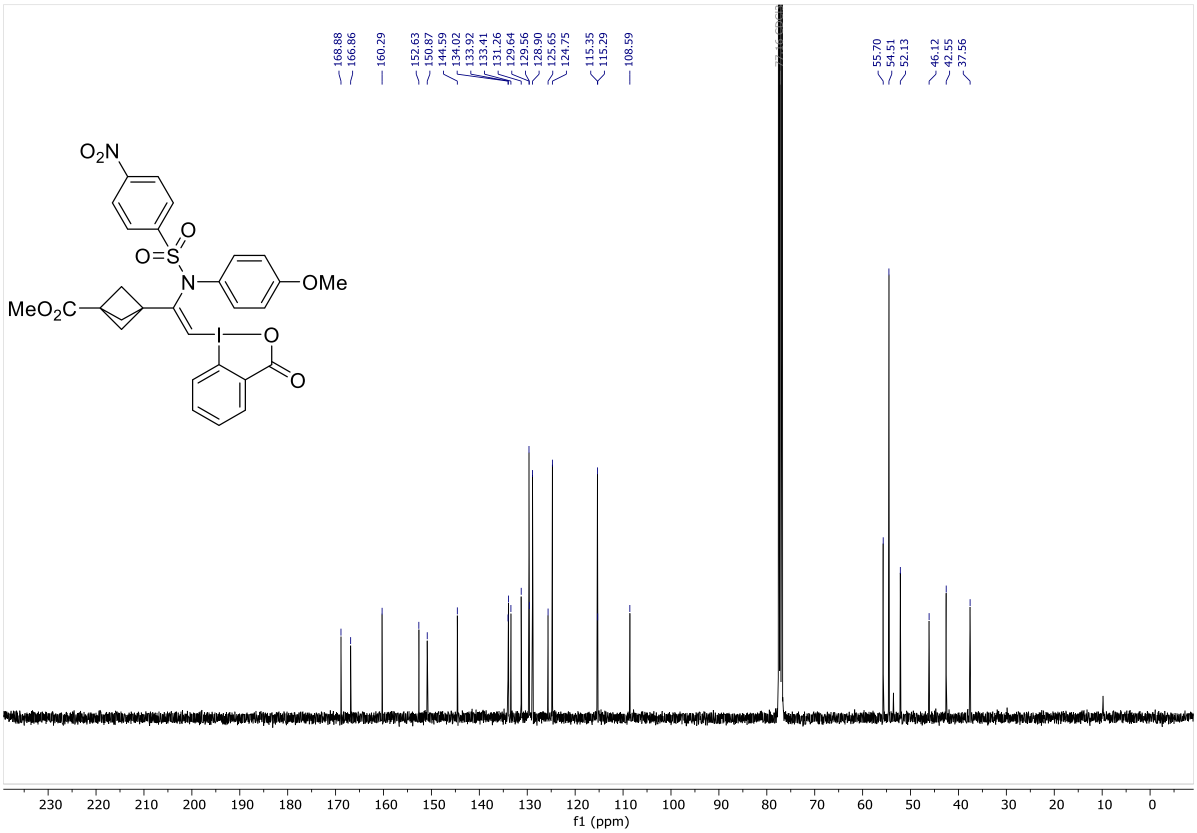

^{1}H NMR (400 MHz, $CDCl_3$) of compound **29g**

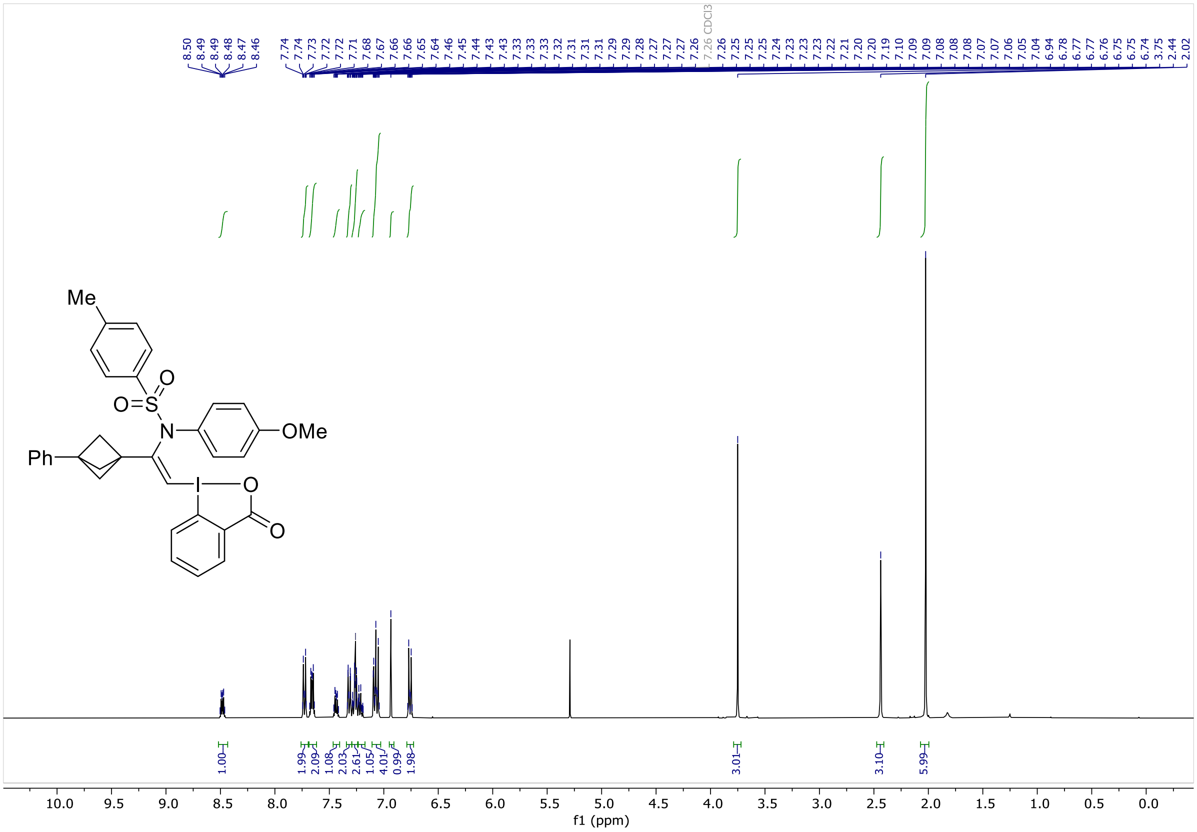

^{13}C NMR (101 MHz, $CDCl_3$) of compound **29g**

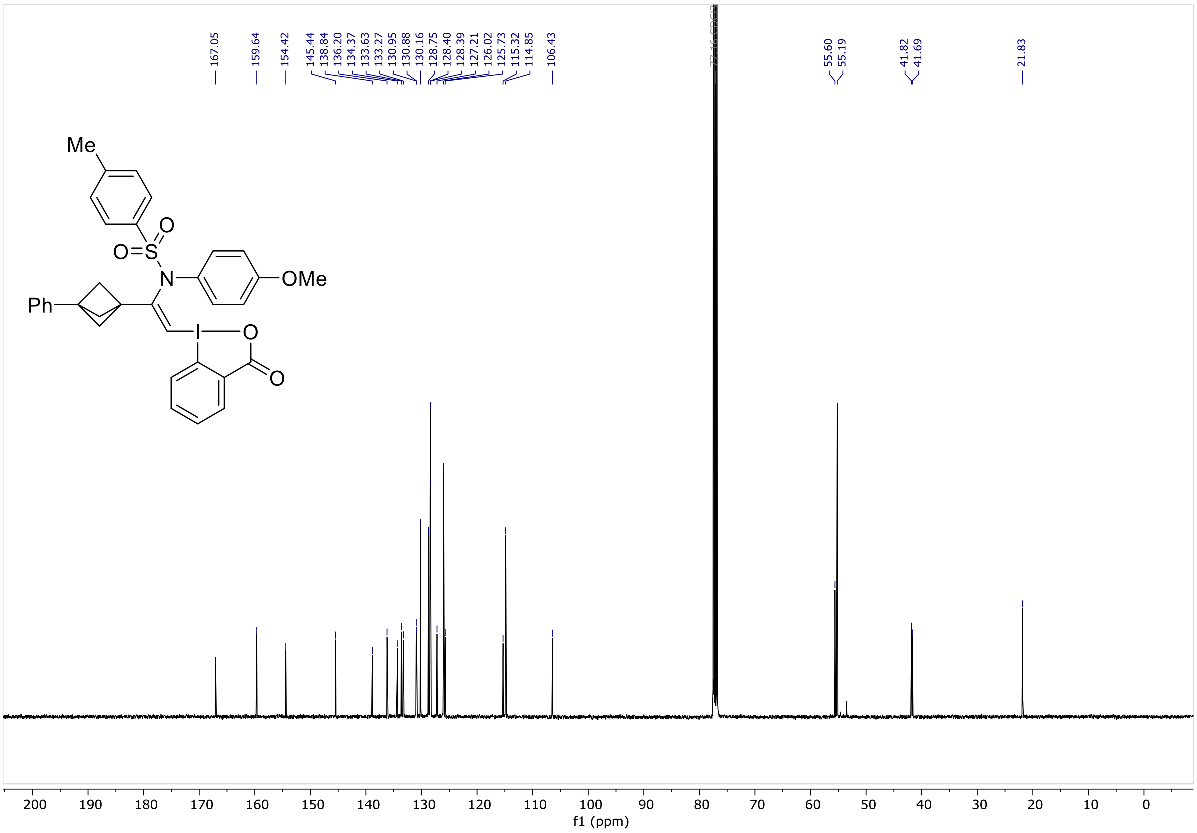

^{1}H NMR (400 MHz, $CDCl_3$) of compound **29h**

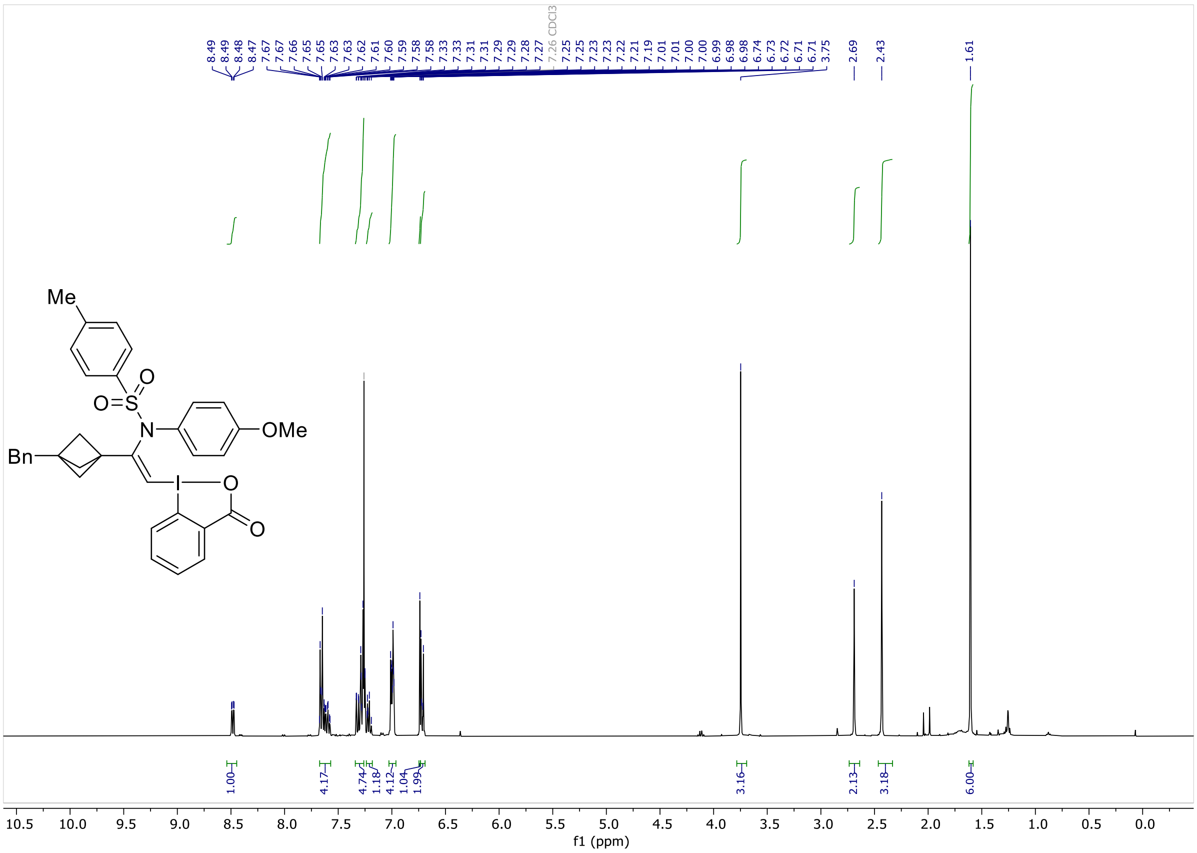

^{13}C NMR (101 MHz, $CDCl_3$) of compound **29h**

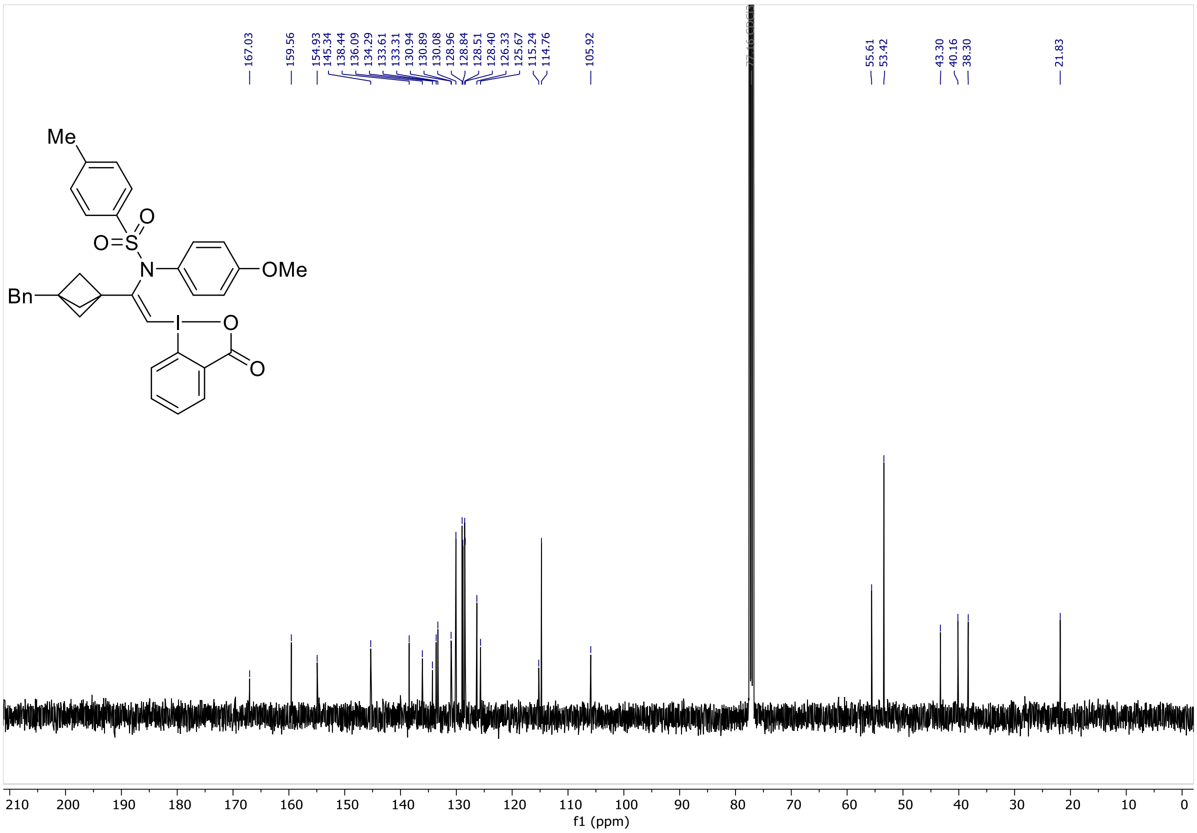

^{1}H NMR (400 MHz, $CDCl_3$) of compound **30a**

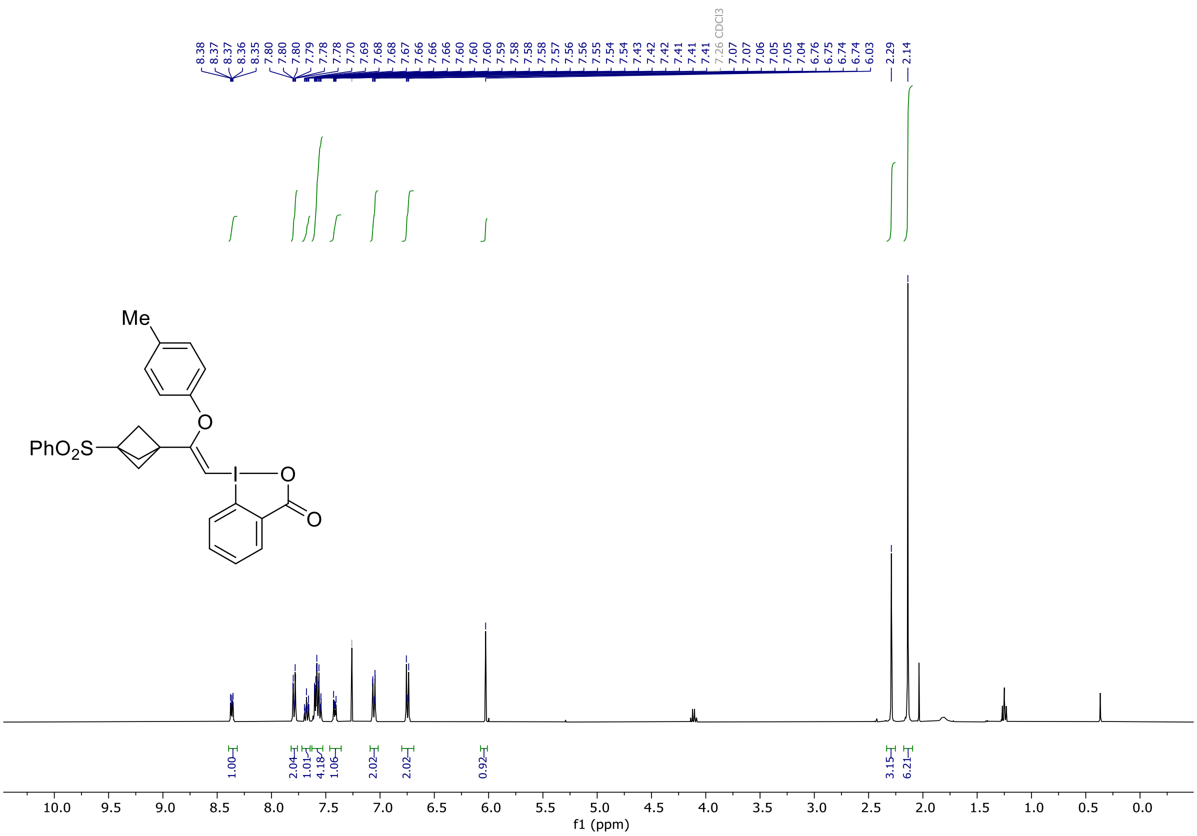

^{13}C NMR (101 MHz, $CDCl_3$) of compound **30a**

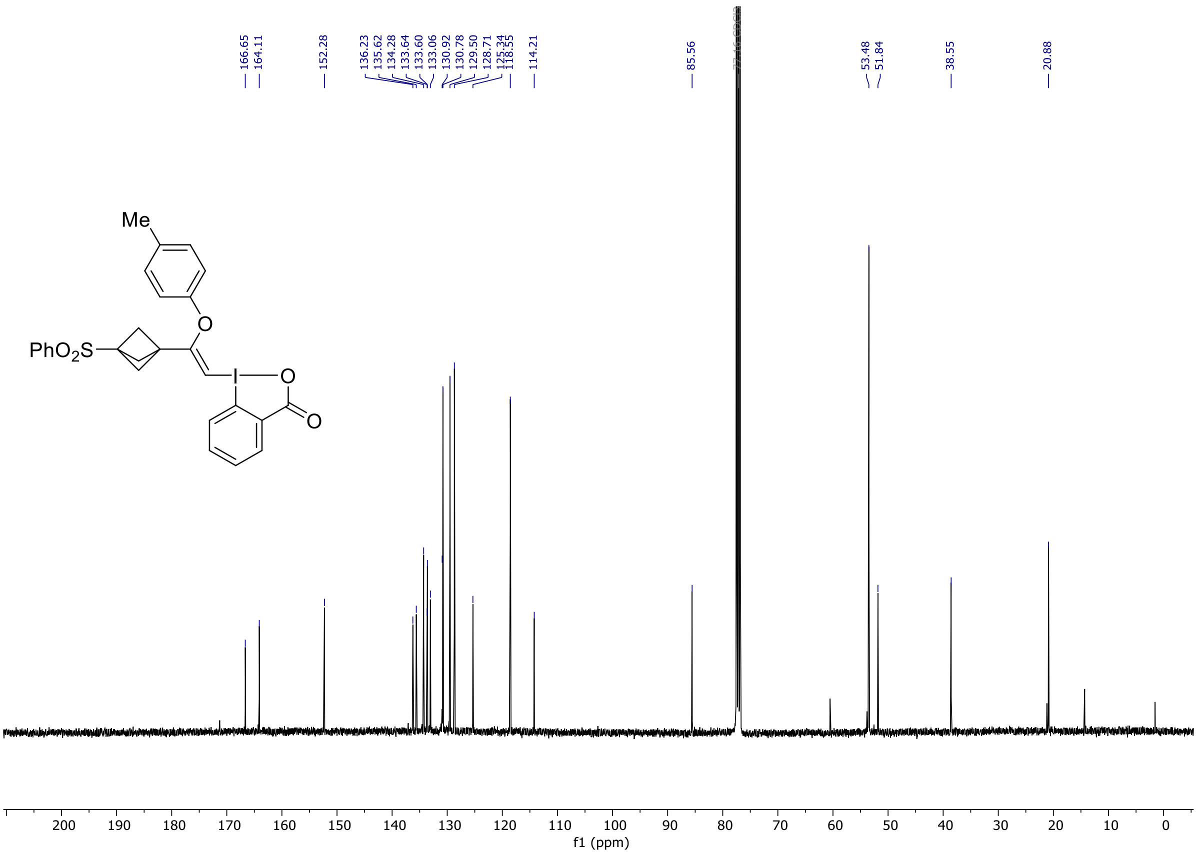

^{1}H NMR (400 MHz, $CDCl_3$) of compound **30b**

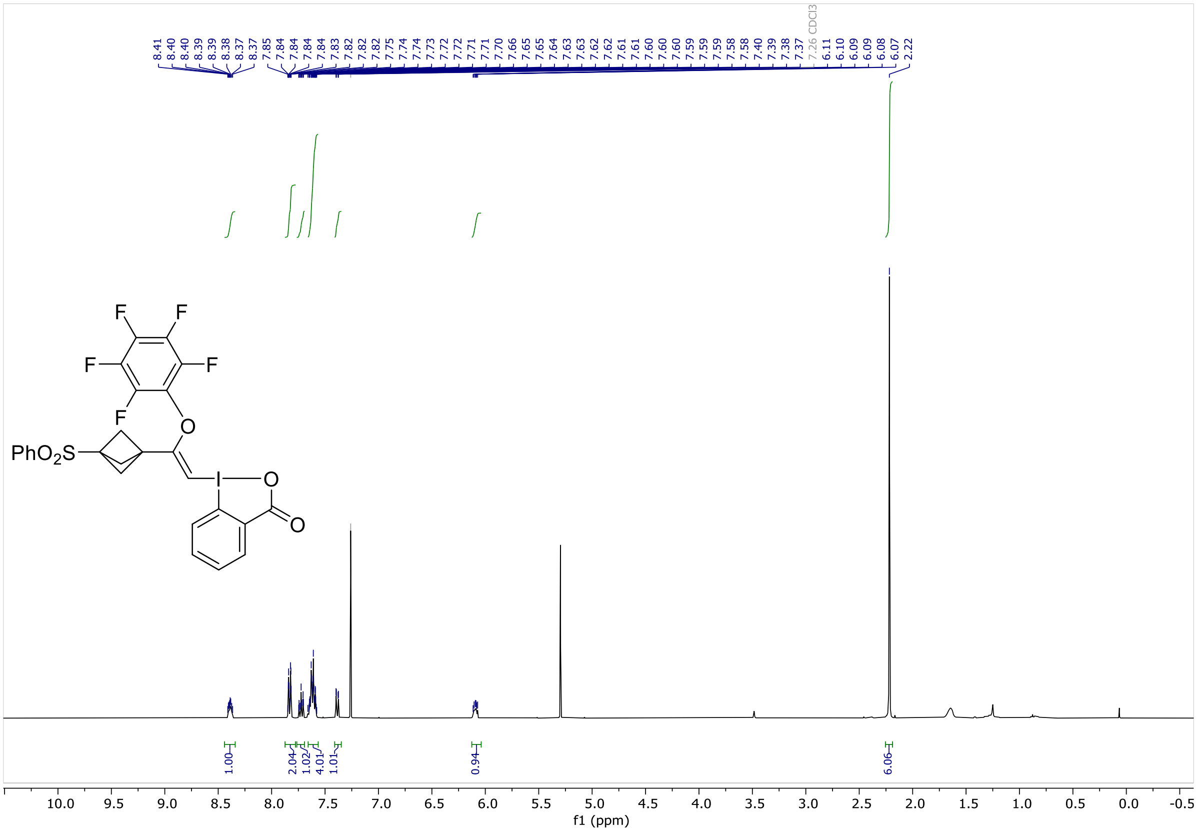

^{13}C NMR (101 MHz, $CDCl_3$) of compound **30b**

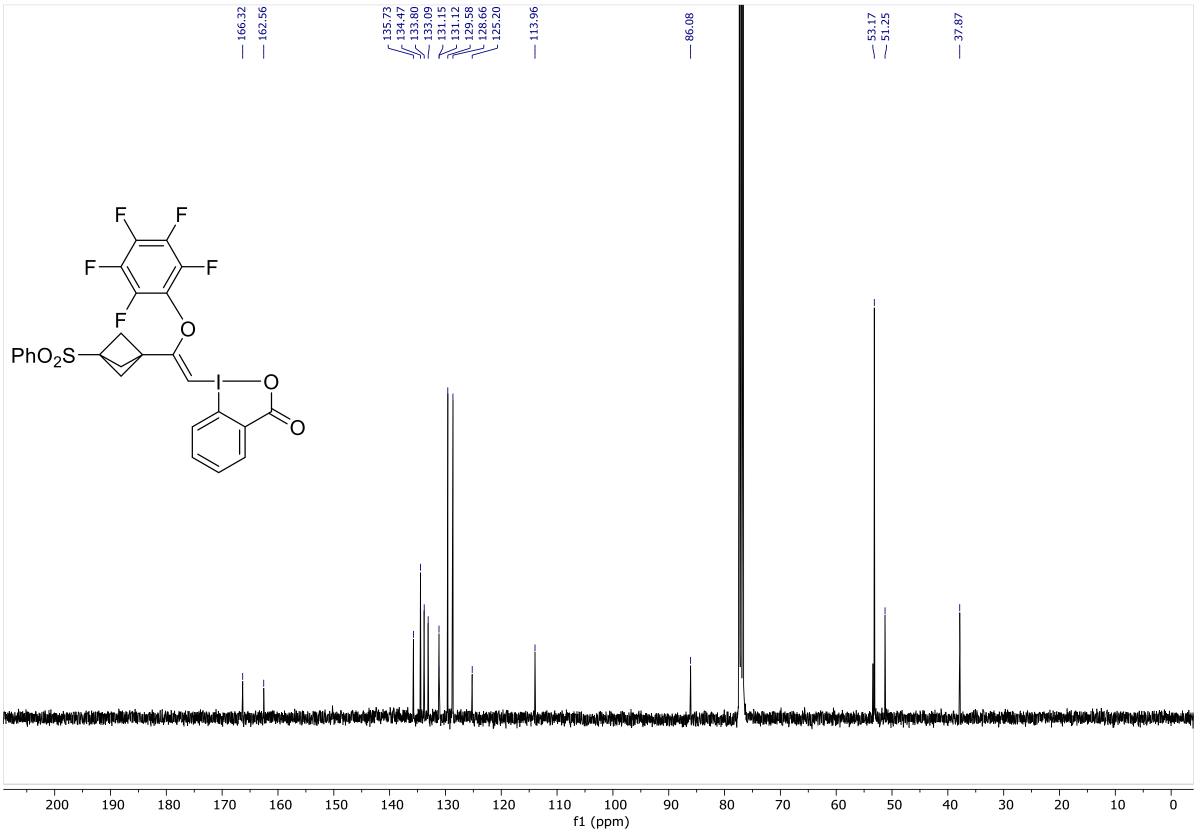

^{19}F NMR (376 MHz, $CDCl_3$) of compound **30b**

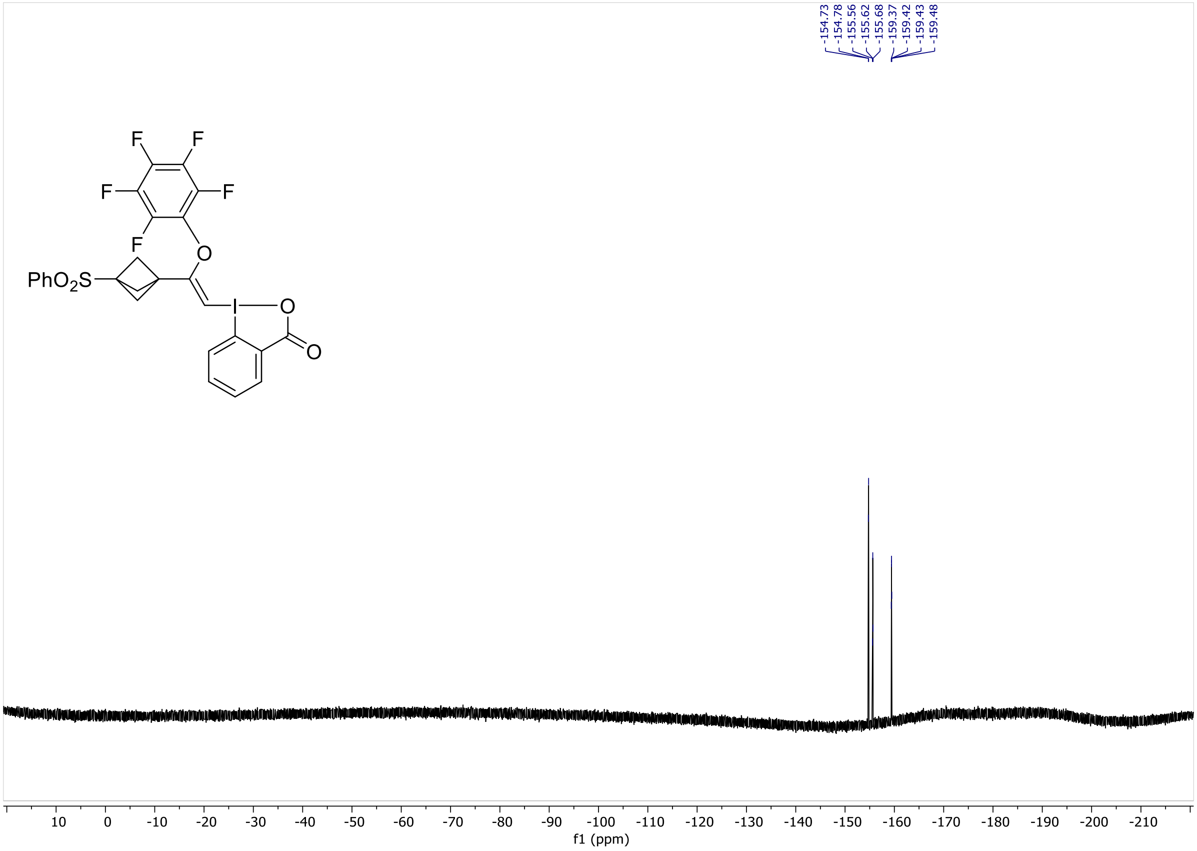

^{1}H NMR (400 MHz, $CDCl_3$) of compound **30c**

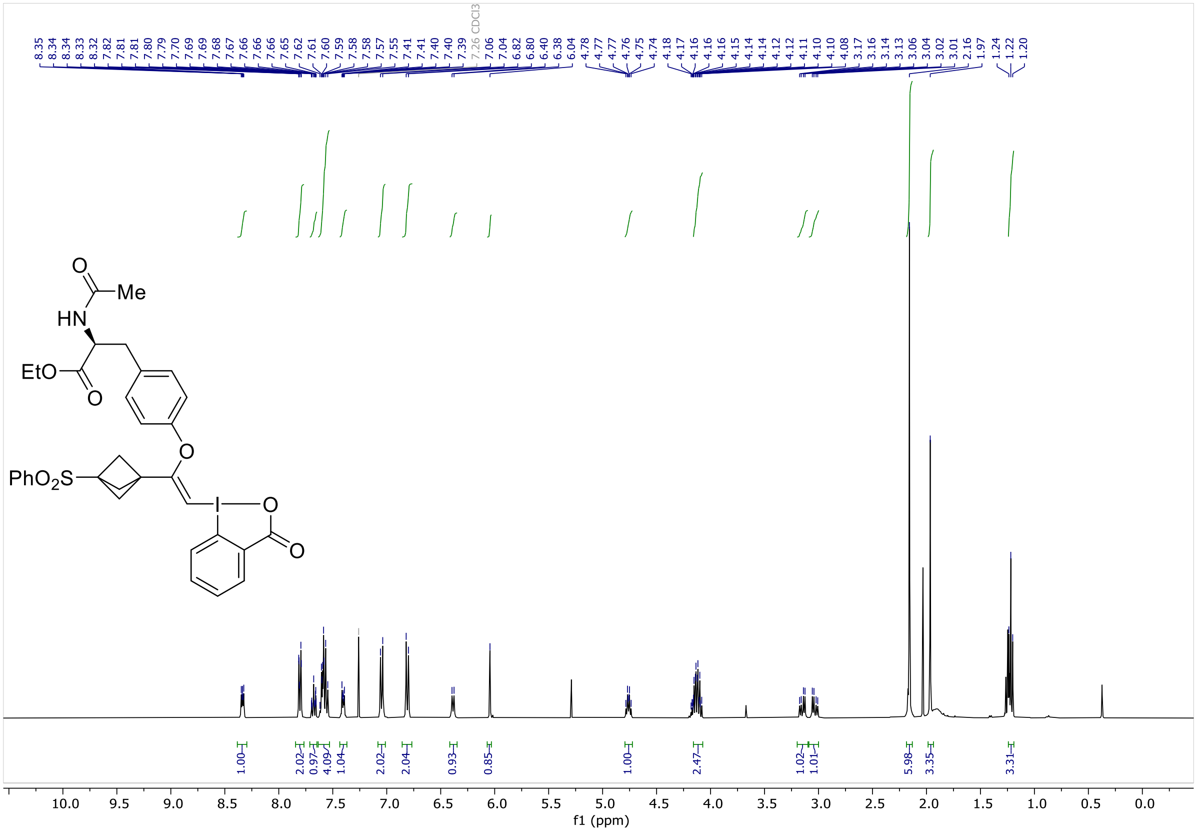

^{13}C NMR (101 MHz, $CDCl_3$) of compound **30c**

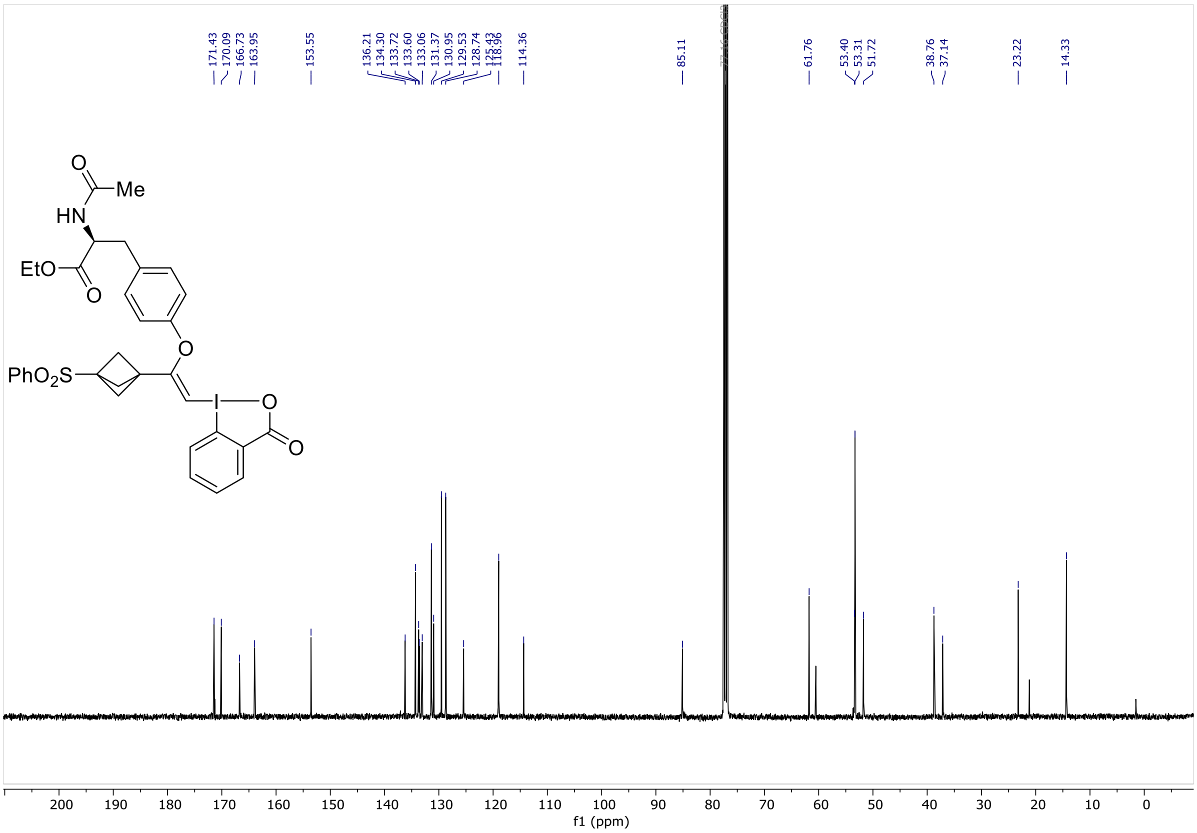

^{1}H NMR (400 MHz, $CDCl_3$) of compound **30d**

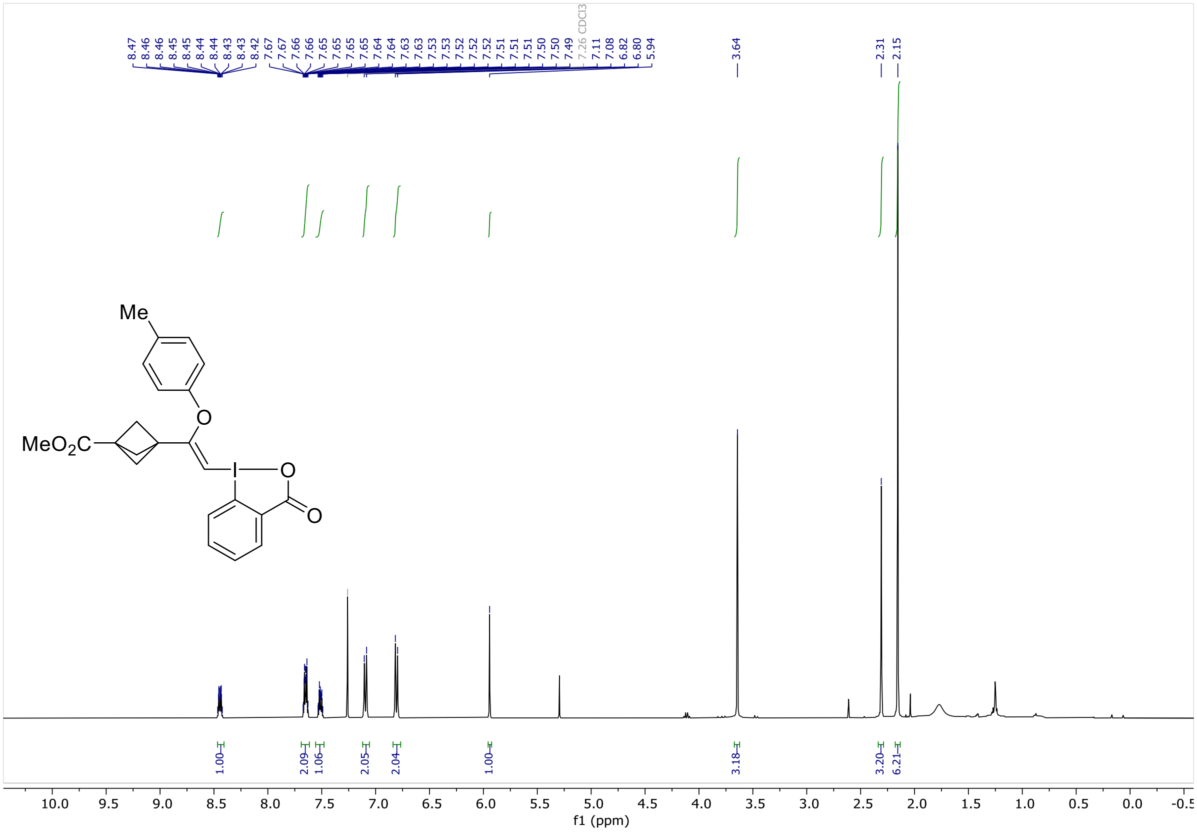

^{13}**C NMR** (101 MHz, $CDCl_3$) of compound **30d**

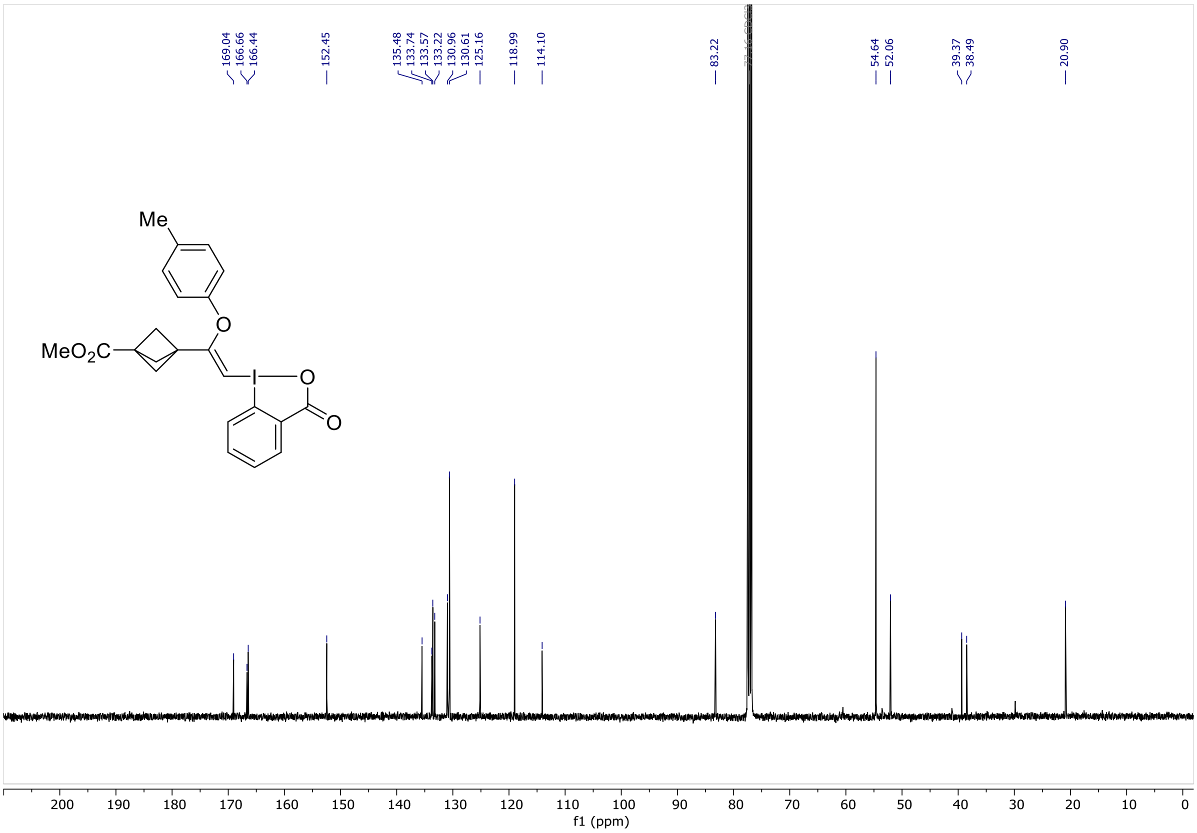

^{1}**H NMR** (400 MHz, $CDCl_3$) of compound **30e**

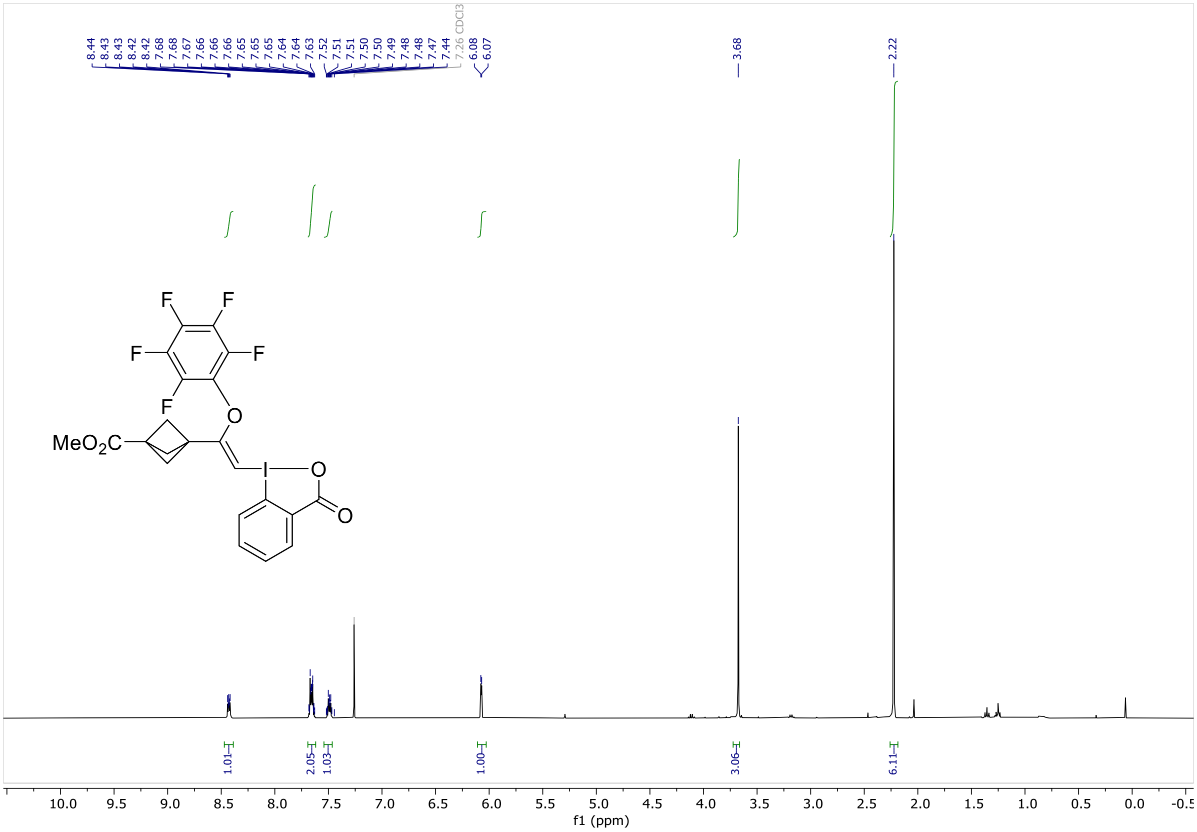

^{13}C NMR (101 MHz, $CDCl_3$) of compound **30e**

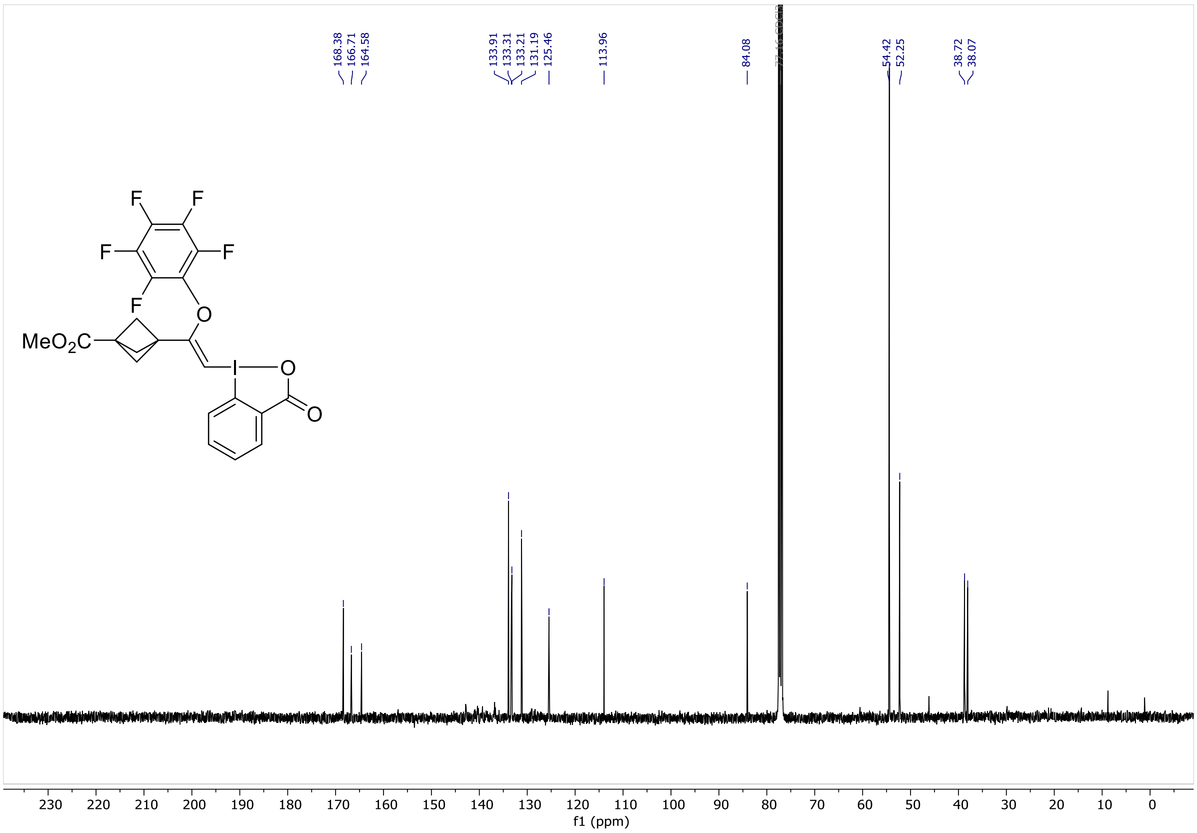

^{19}F NMR (376 MHz, $CDCl_3$) of compound **30e**

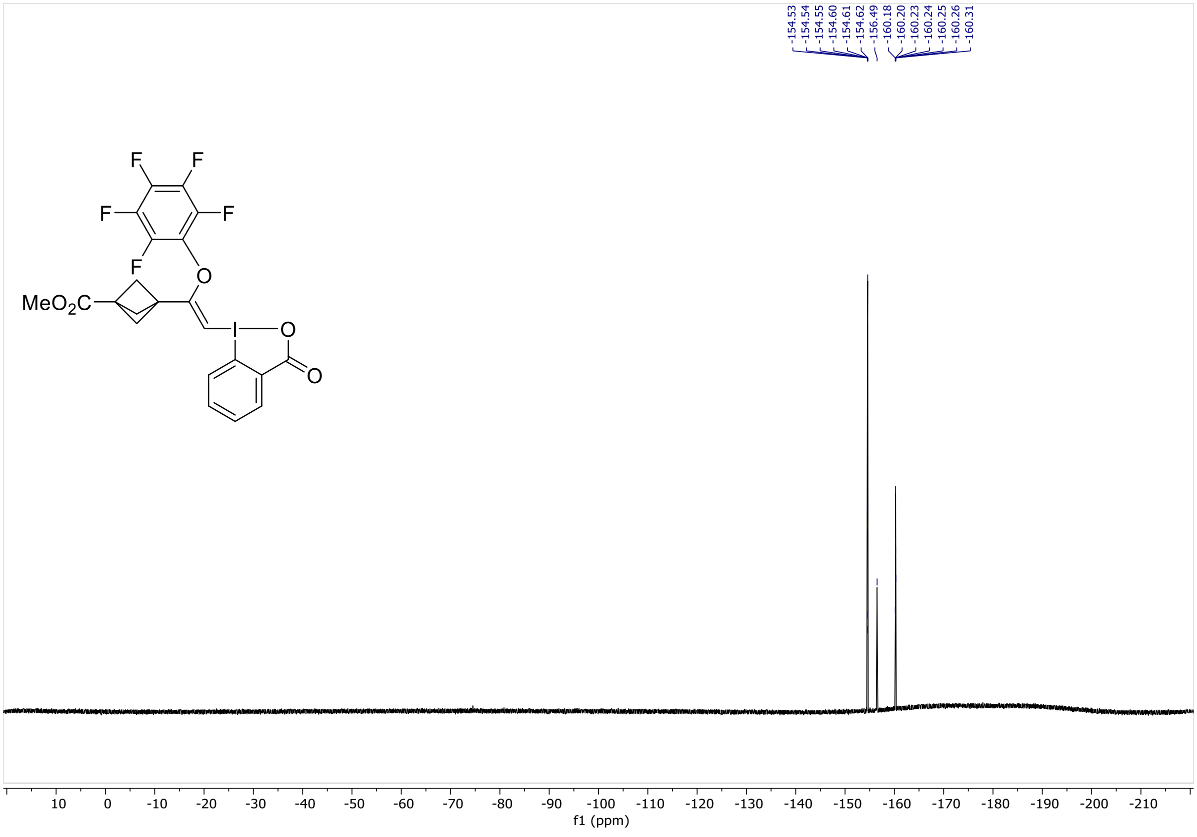

^{1}H NMR (400 MHz, $CDCl_3$/ MeOD) of compound **30f**

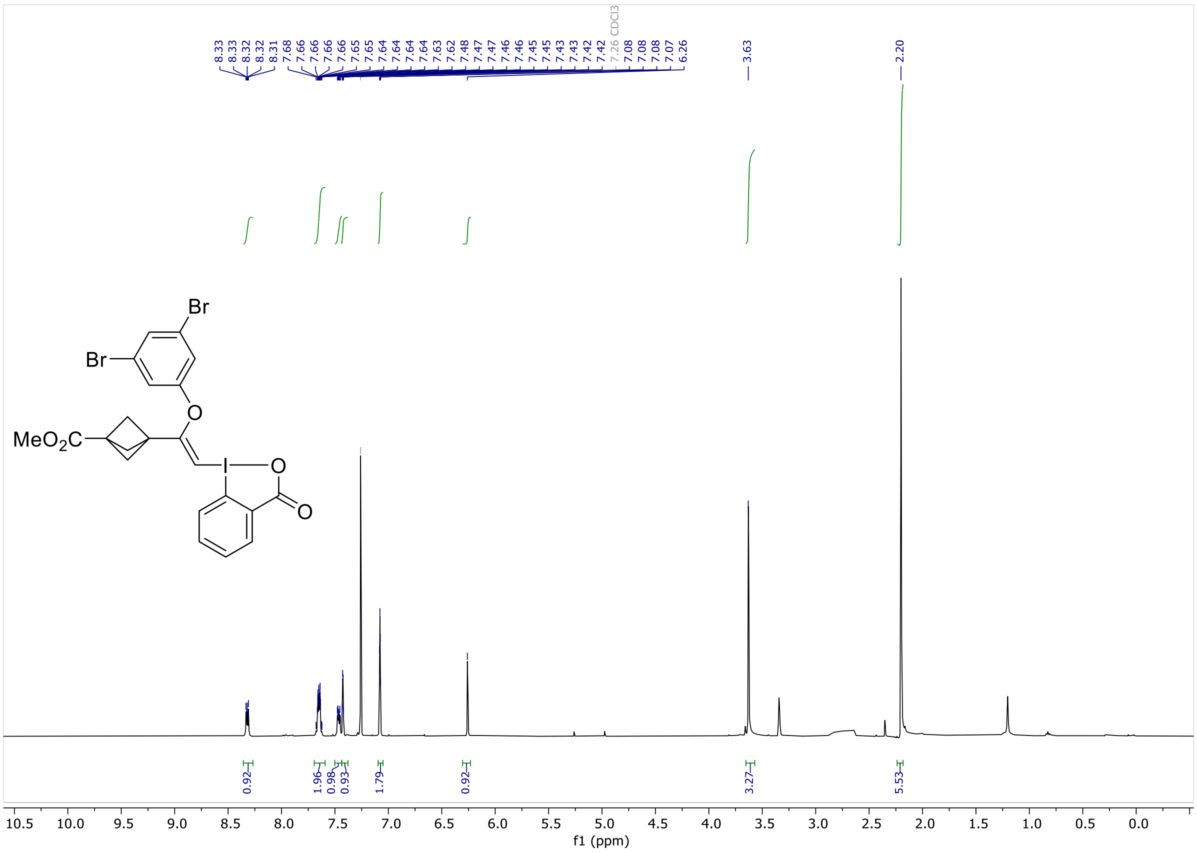

^{13}C NMR (101 MHz, $CDCl_3$/ MeOD) of compound **30f**

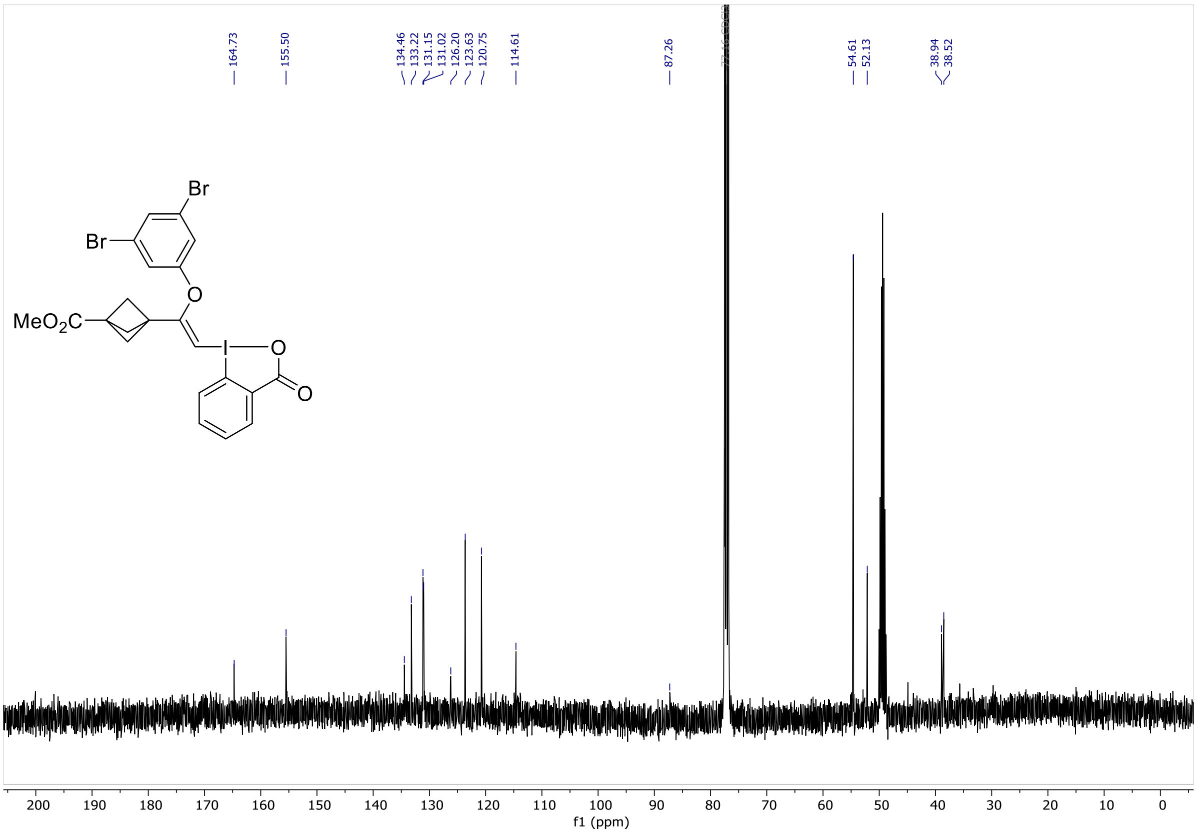

^{1}H NMR (400 MHz, $CDCl_3$) of compound **30g**

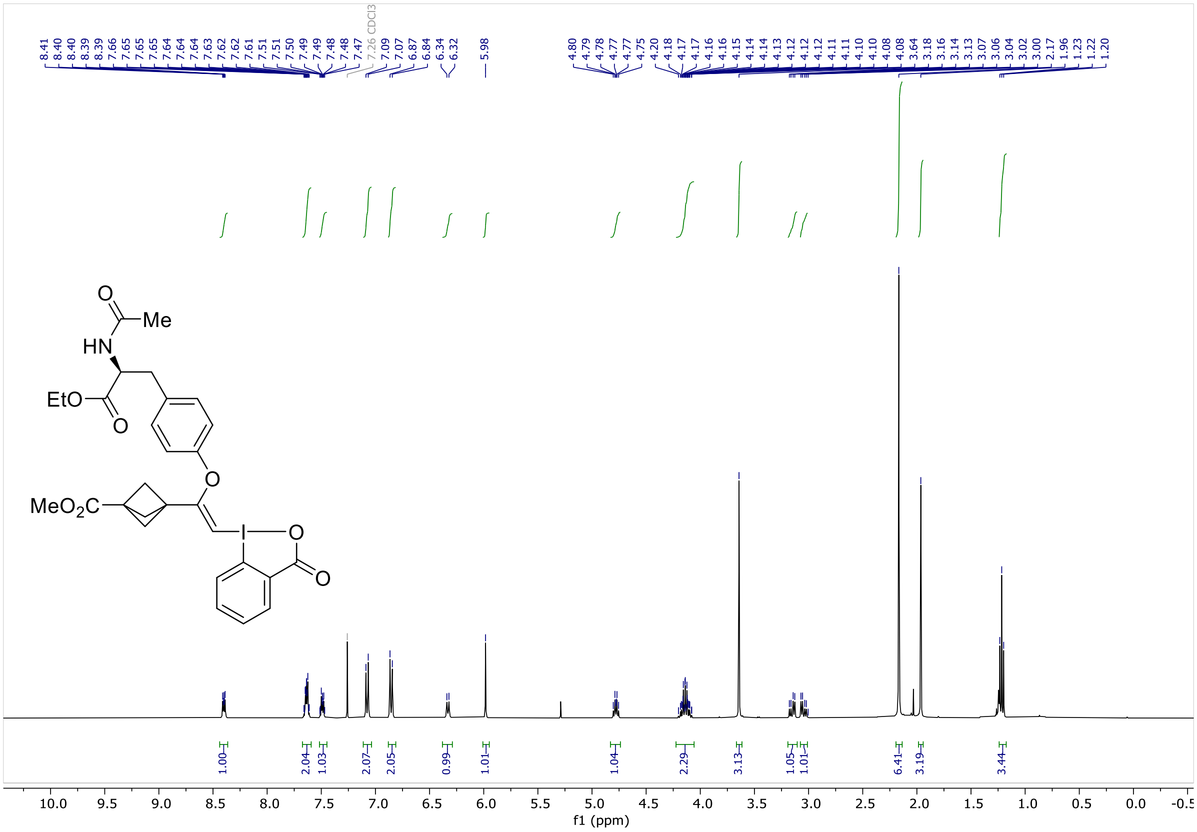

^{13}C NMR (101 MHz, $CDCl_3$) of compound **30g**

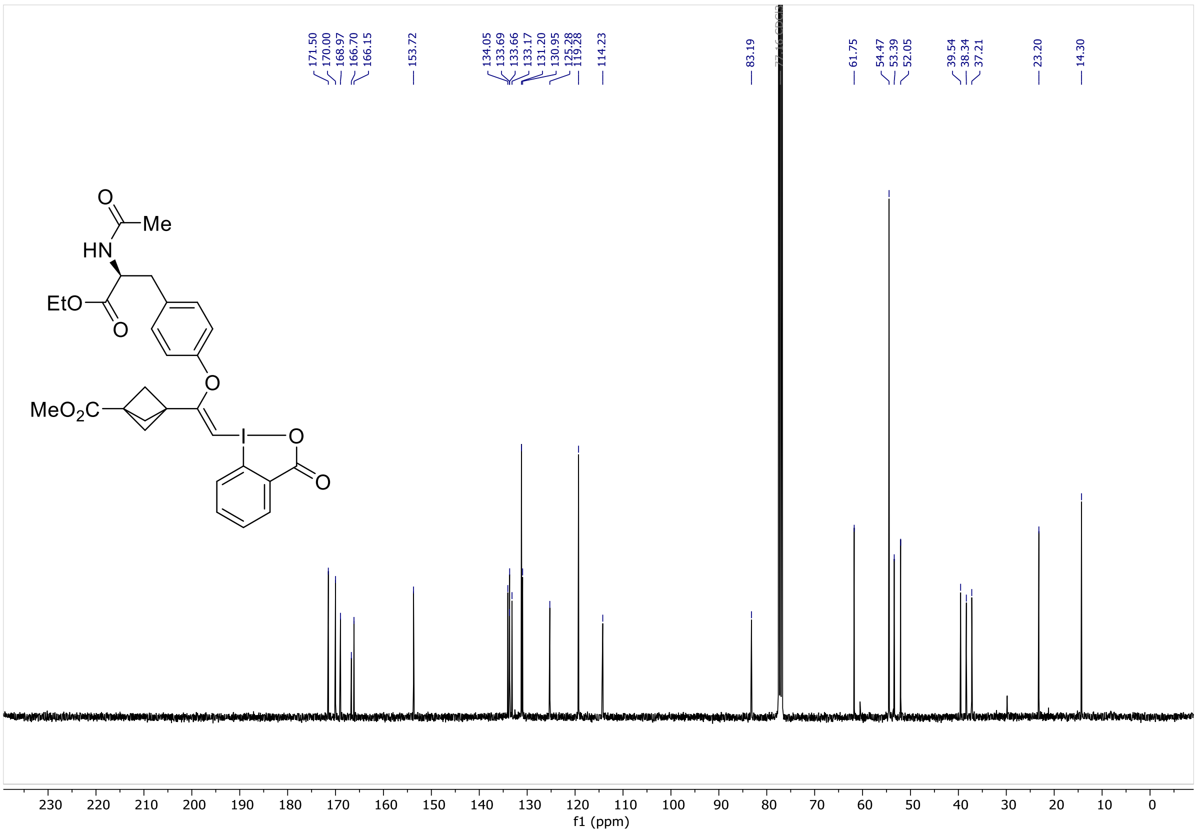

^{1}H NMR (400 MHz, $CDCl_3$) of compound **30h**

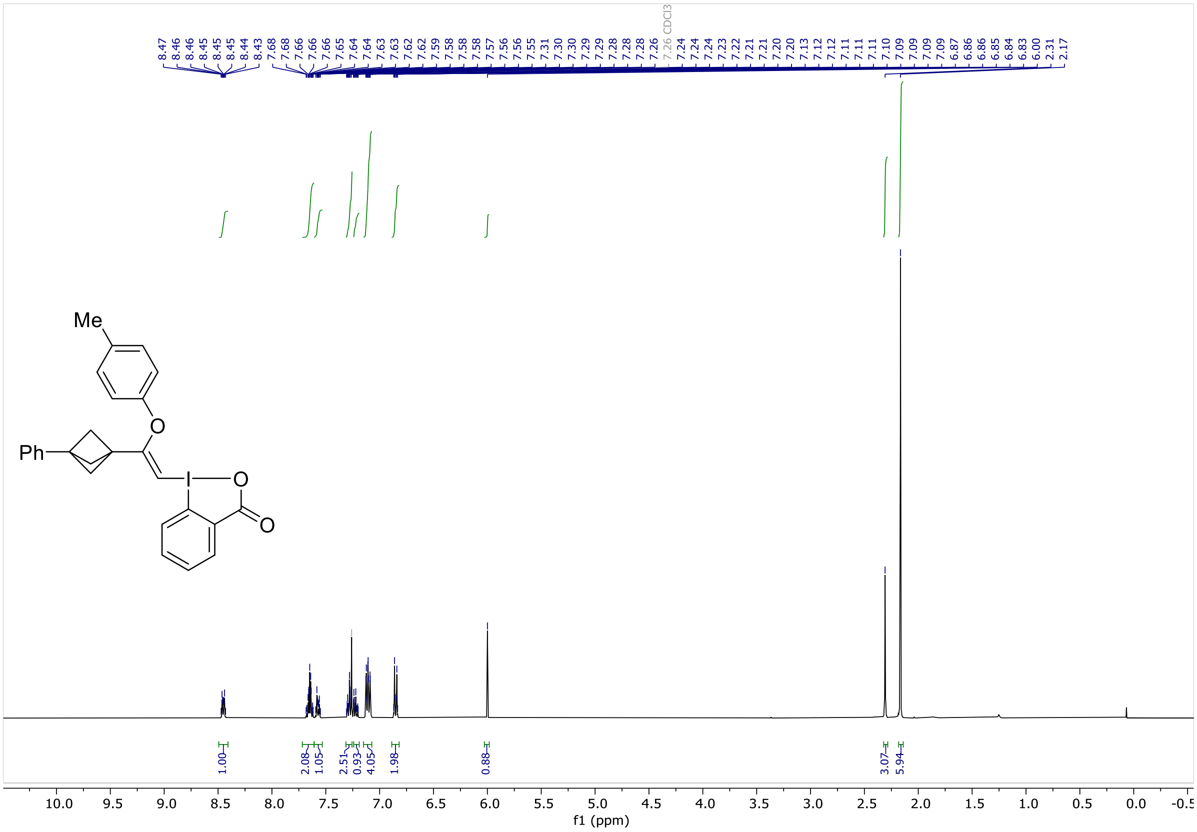

^{13}C NMR (101 MHz, $CDCl_3$) of compound **30h**

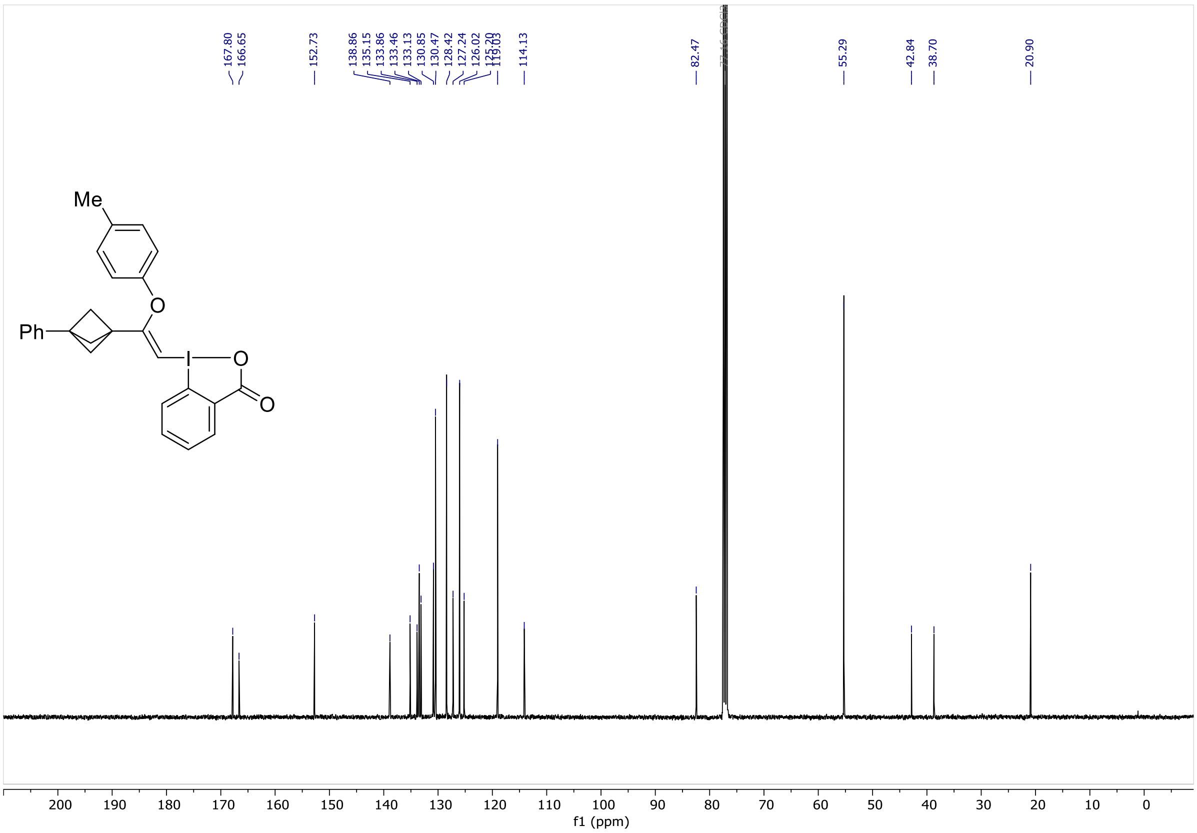

^{1}**H NMR** (400 MHz, $CDCl_3$) of compound **30i**

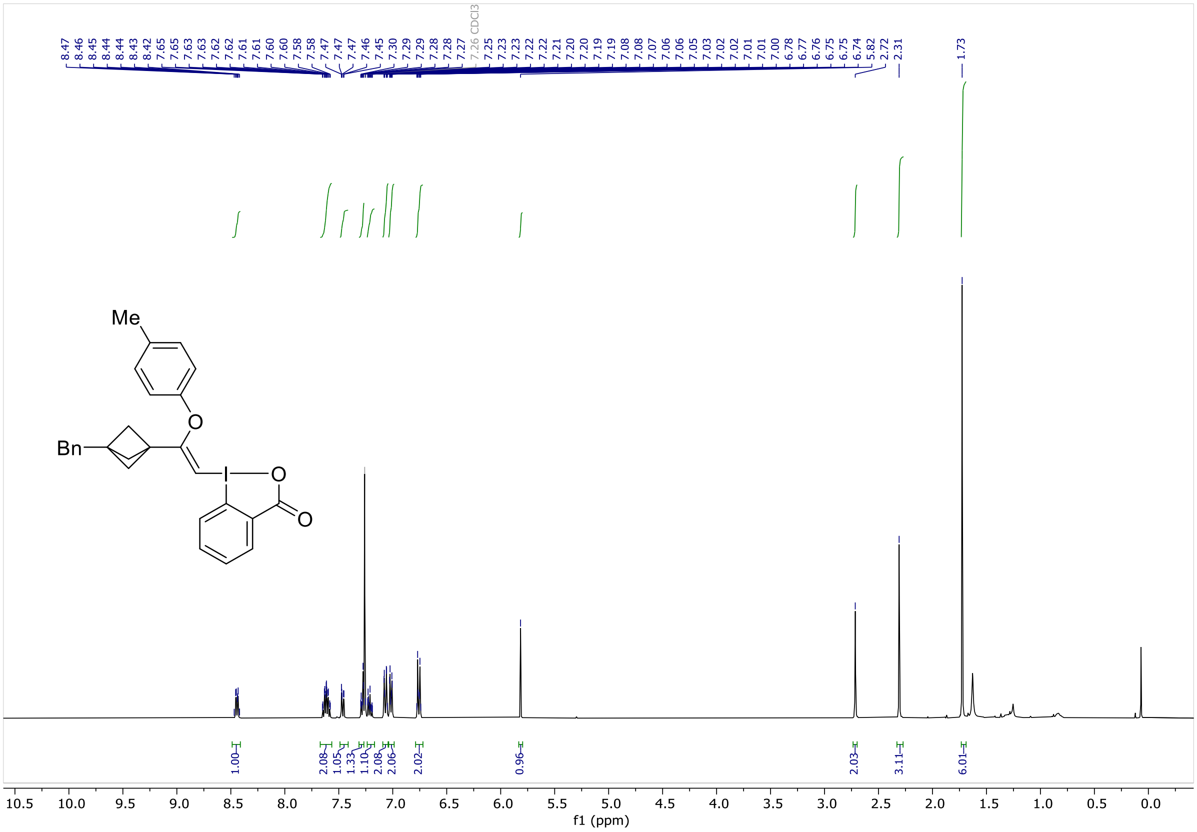

^{13}**C NMR** (101 MHz, $CDCl_3$) of compound **30i**

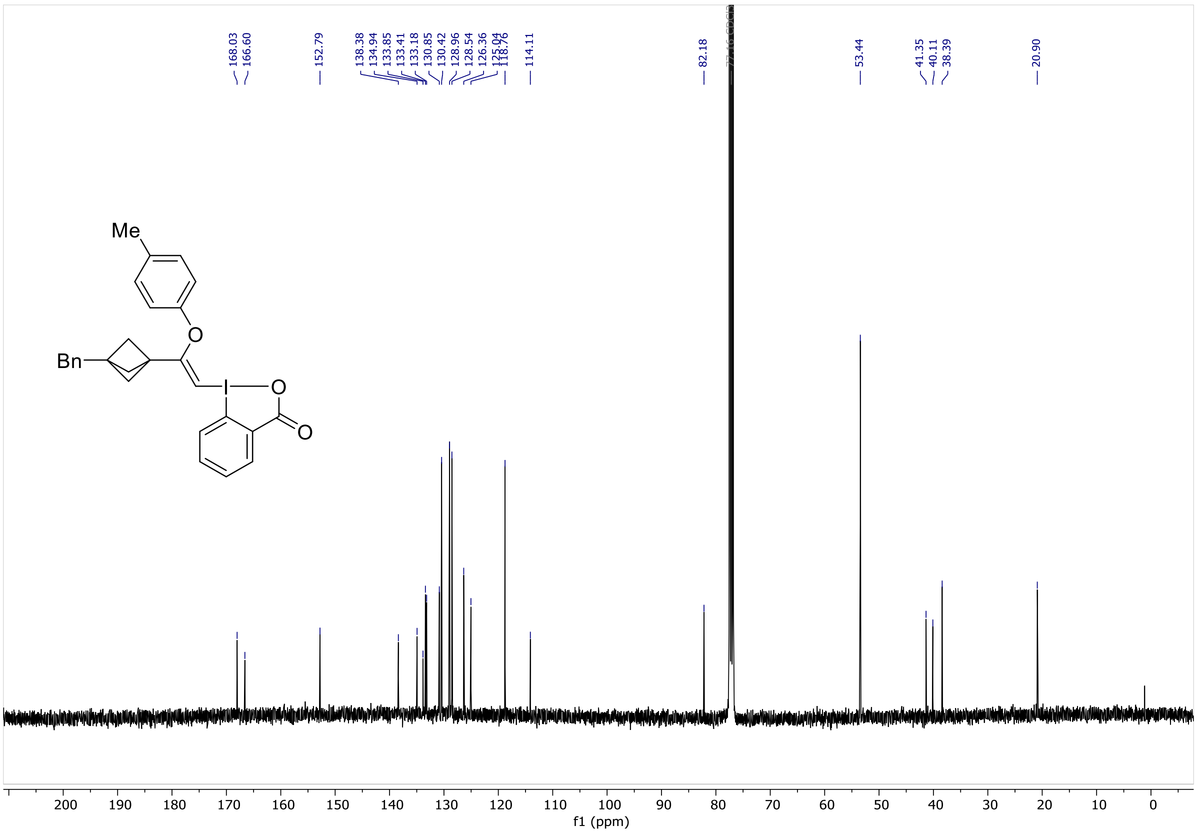

^{1}H NMR (400 MHz, $CDCl_3$/ MeOD) of compound **31**

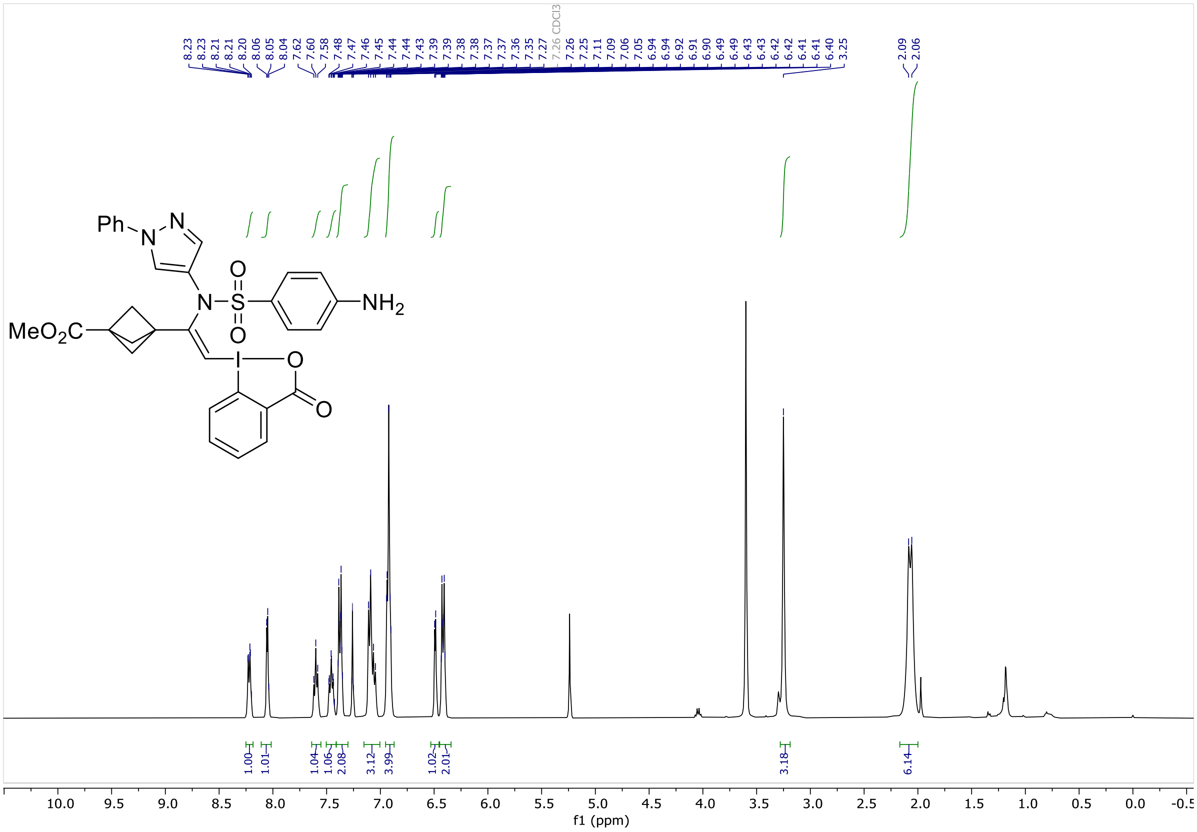

^{13}C NMR (101 MHz, $CDCl_3$/ MeOD) of compound **31**

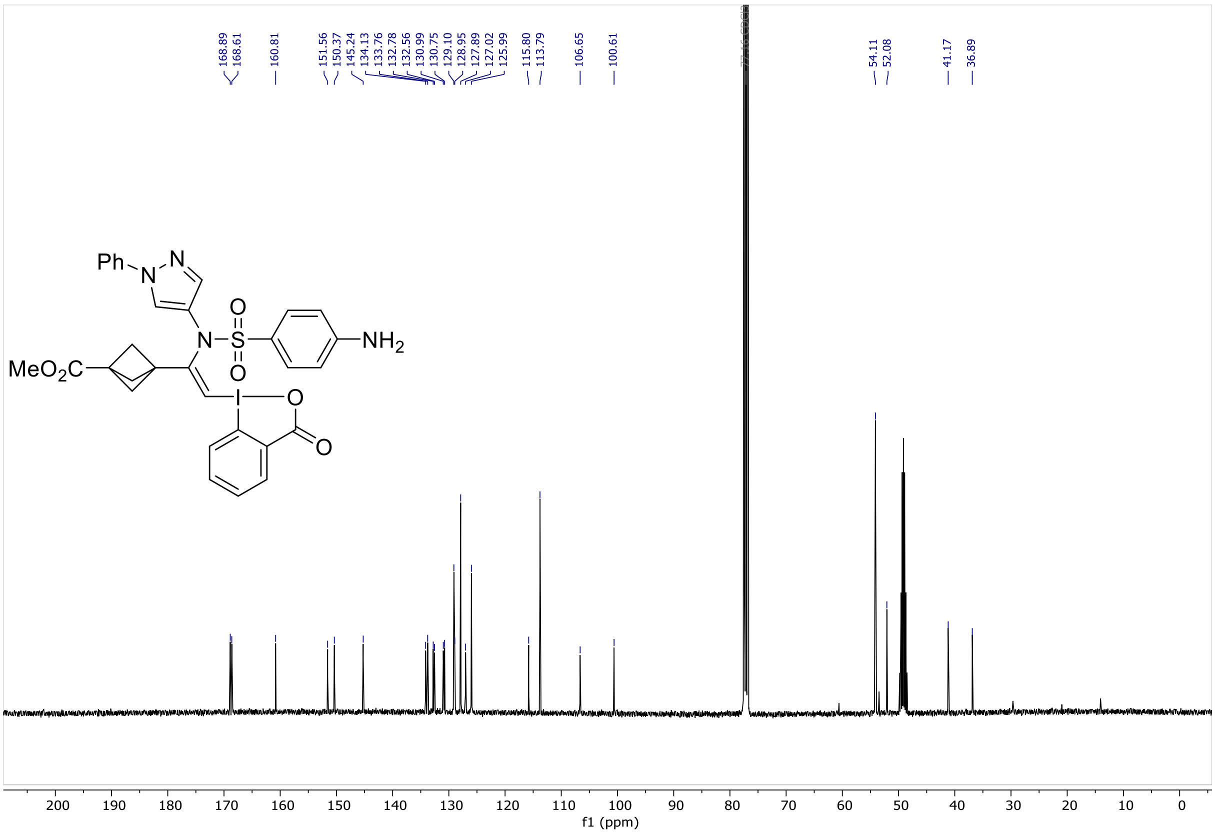

^{1}H NMR (400 MHz, $CDCl_3$) of compound **32**

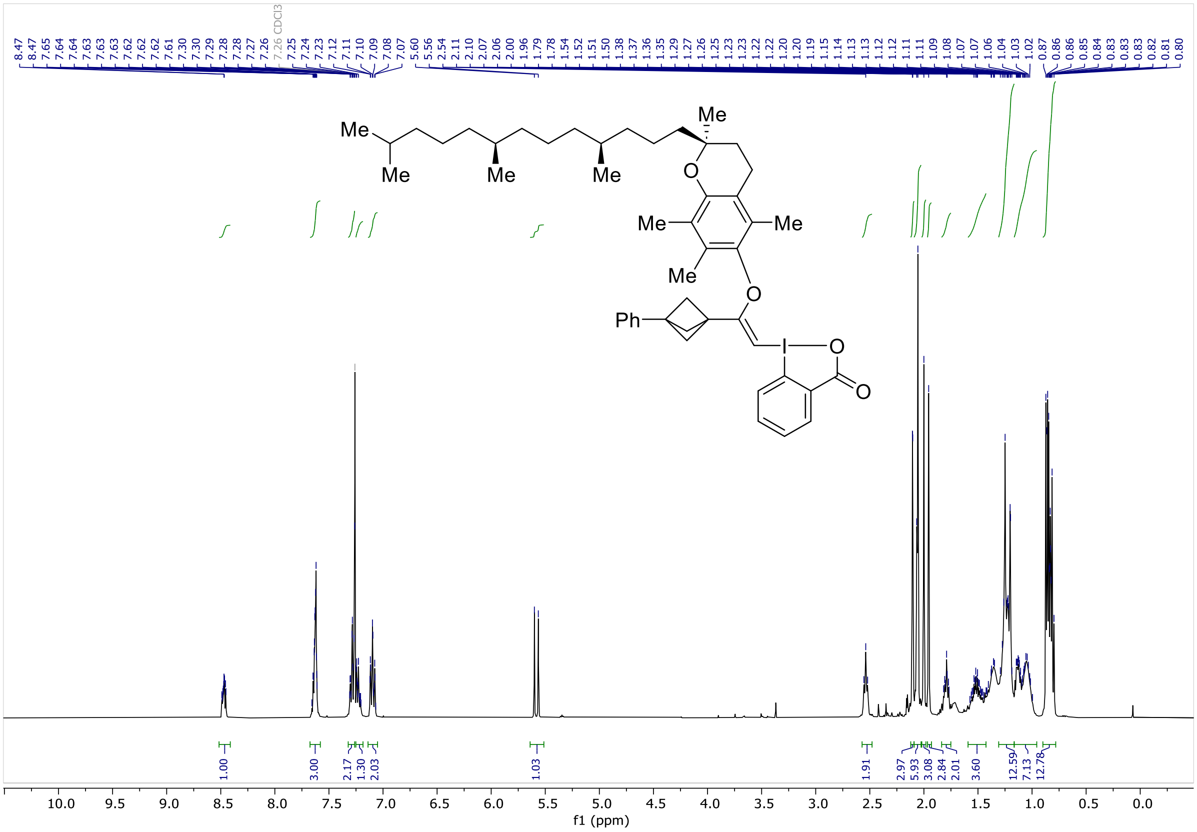

^{13}C NMR (101 MHz, $CDCl_3$) of compound **32**

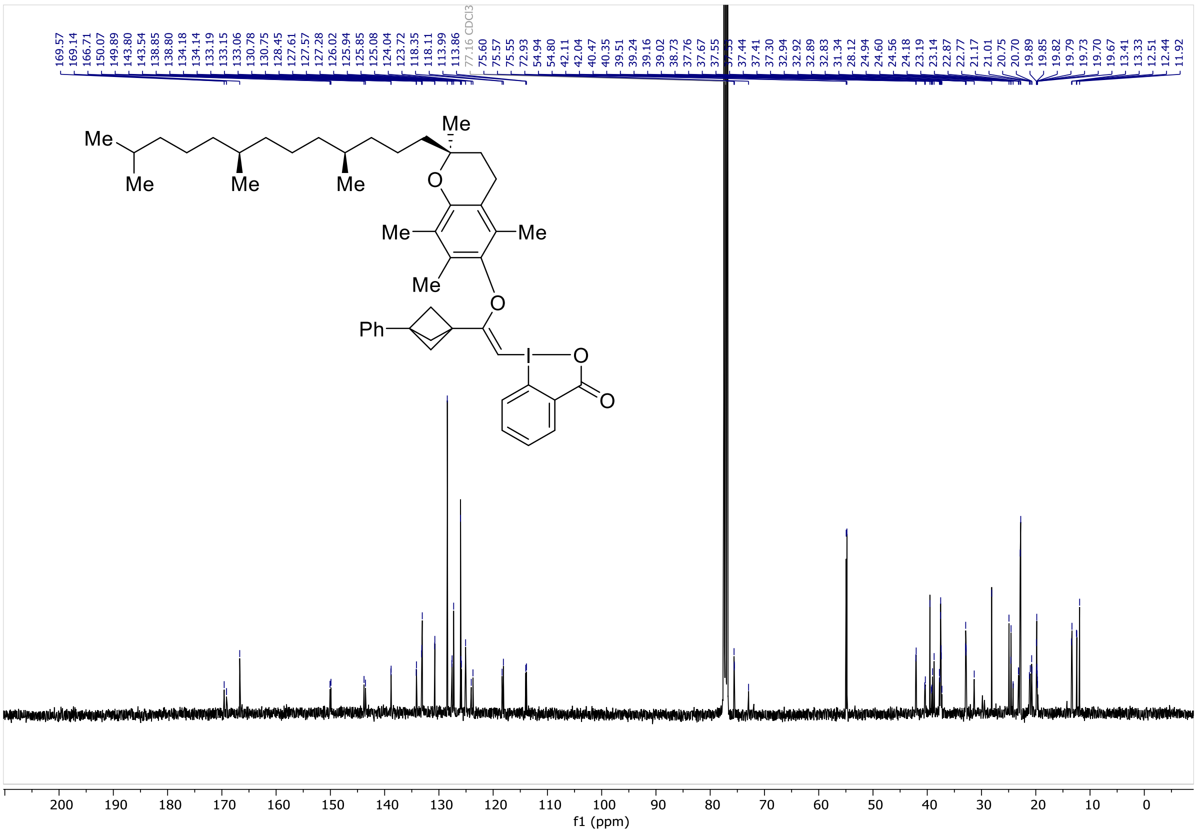

[1]H-[1]H **NOESY** experiment ($CDCl_3$) of compound **32**

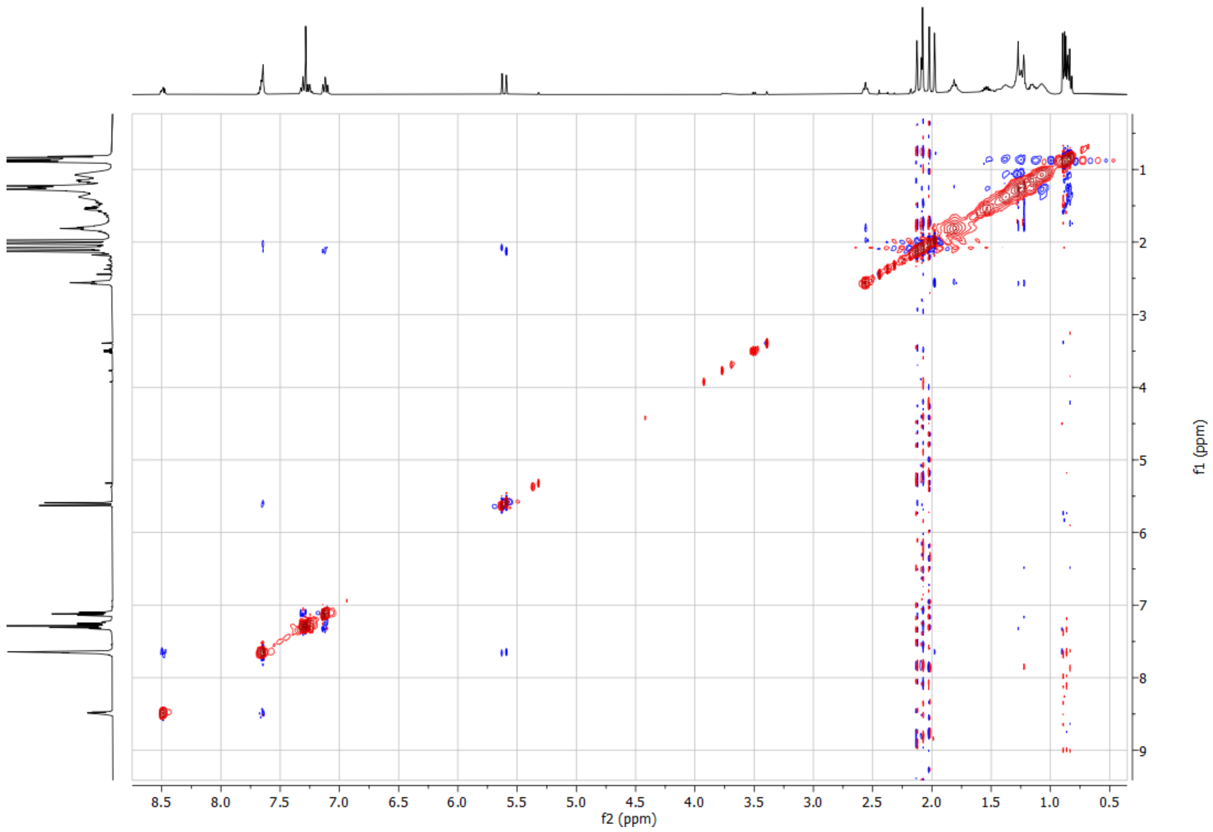

A possible explanation for the NOESY signals could be E/Z isomers.

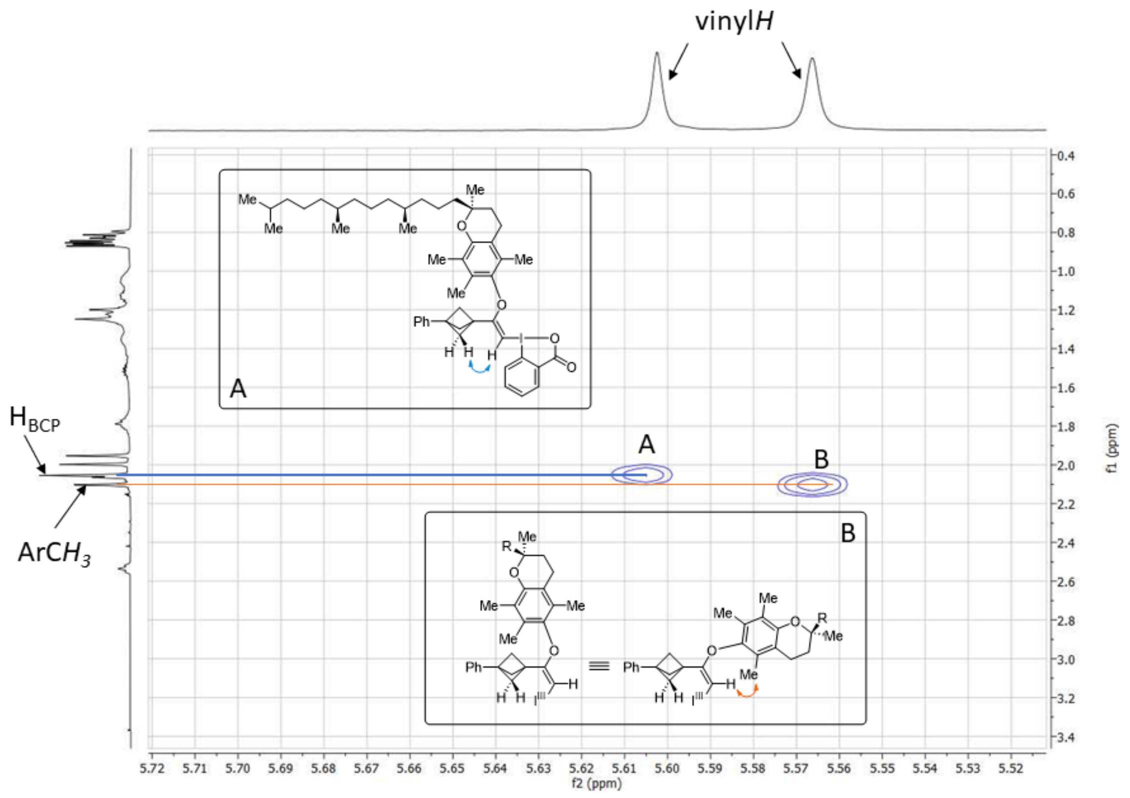

^{1}H NMR (400 MHz, $CDCl_3$) of compound **33**

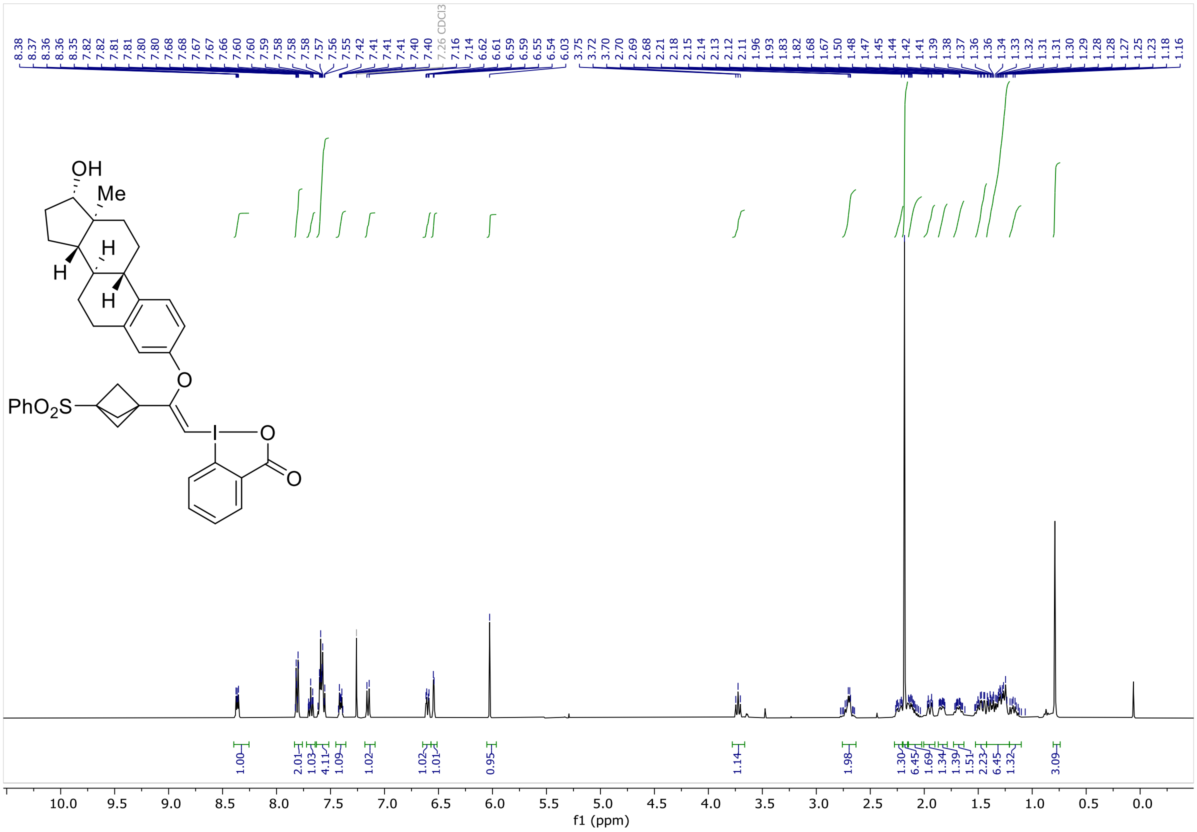

^{13}C NMR (101 MHz, $CDCl_3$) of compound **33**

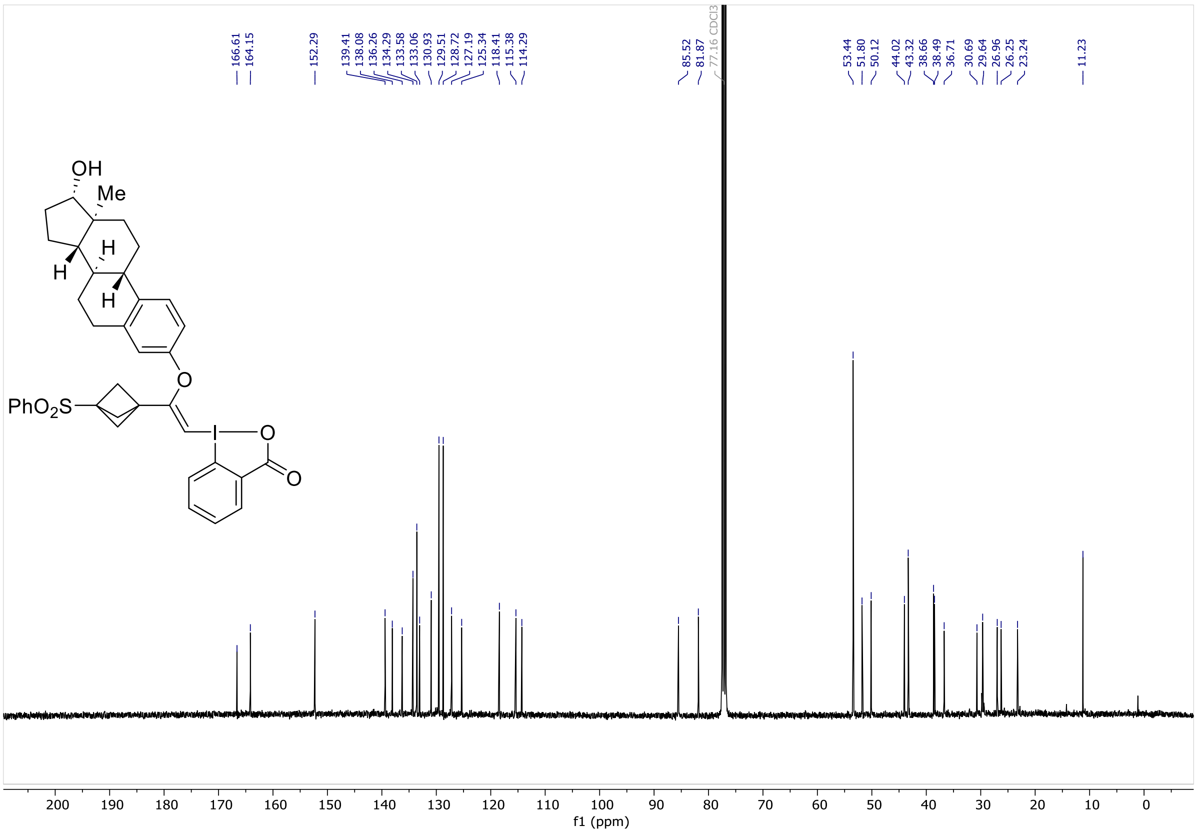

^{1}H NMR (400 MHz, $CDCl_3$) of compound **34**

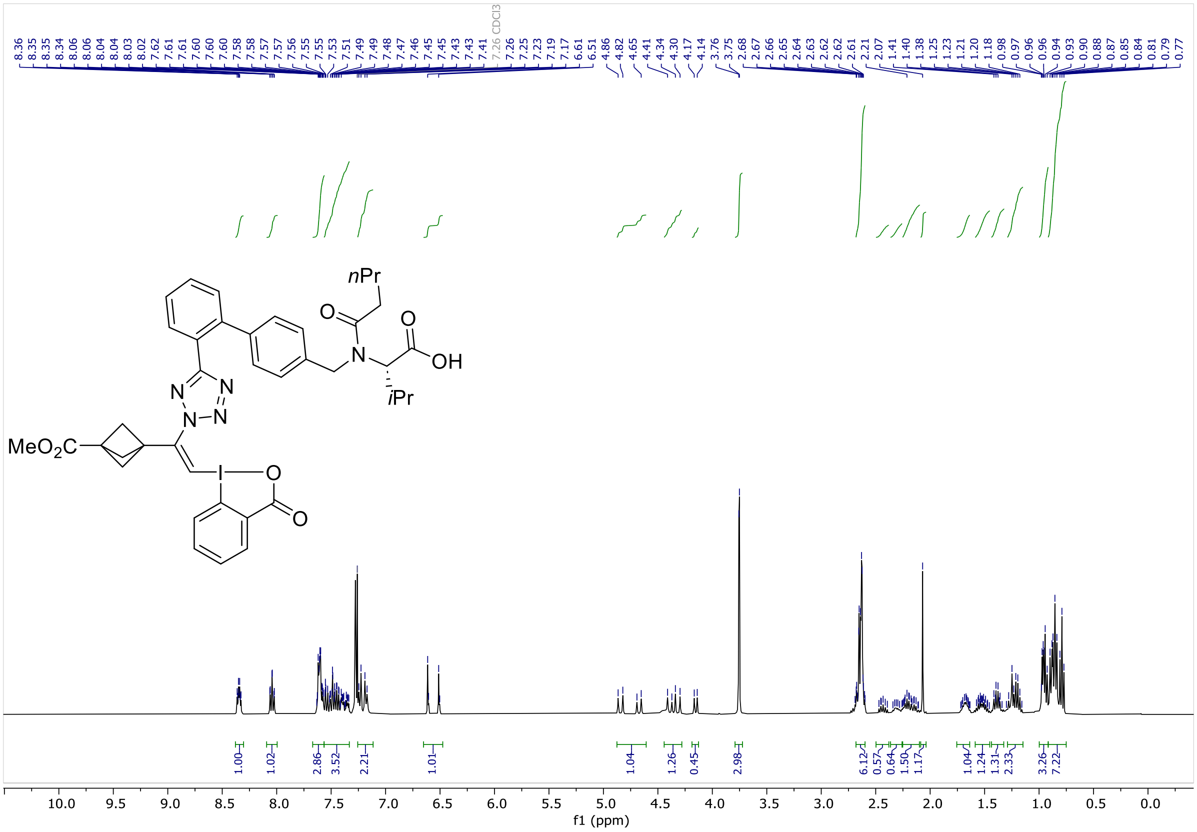

^{13}C NMR (101 MHz, $CDCl_3$) of compound **34**

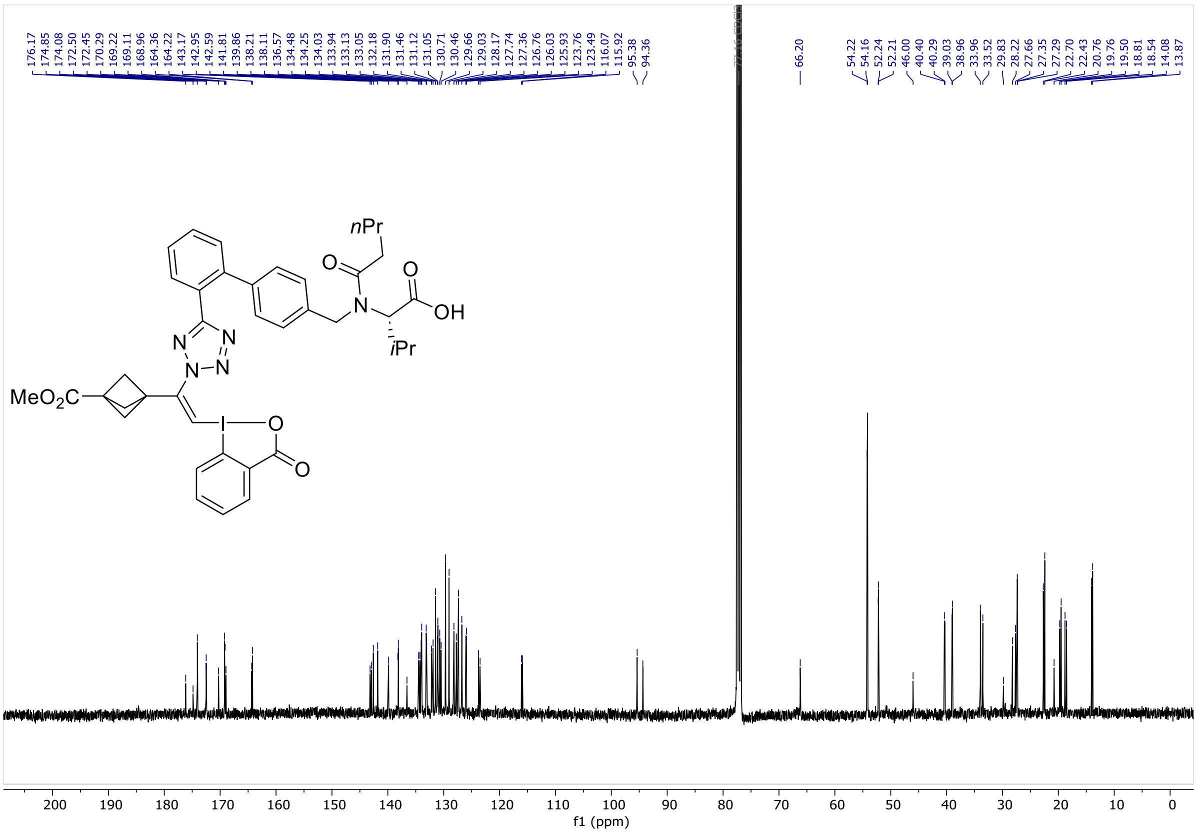

^{1}H NMR (400 MHz, DMSO) of compound **34** at 363 K

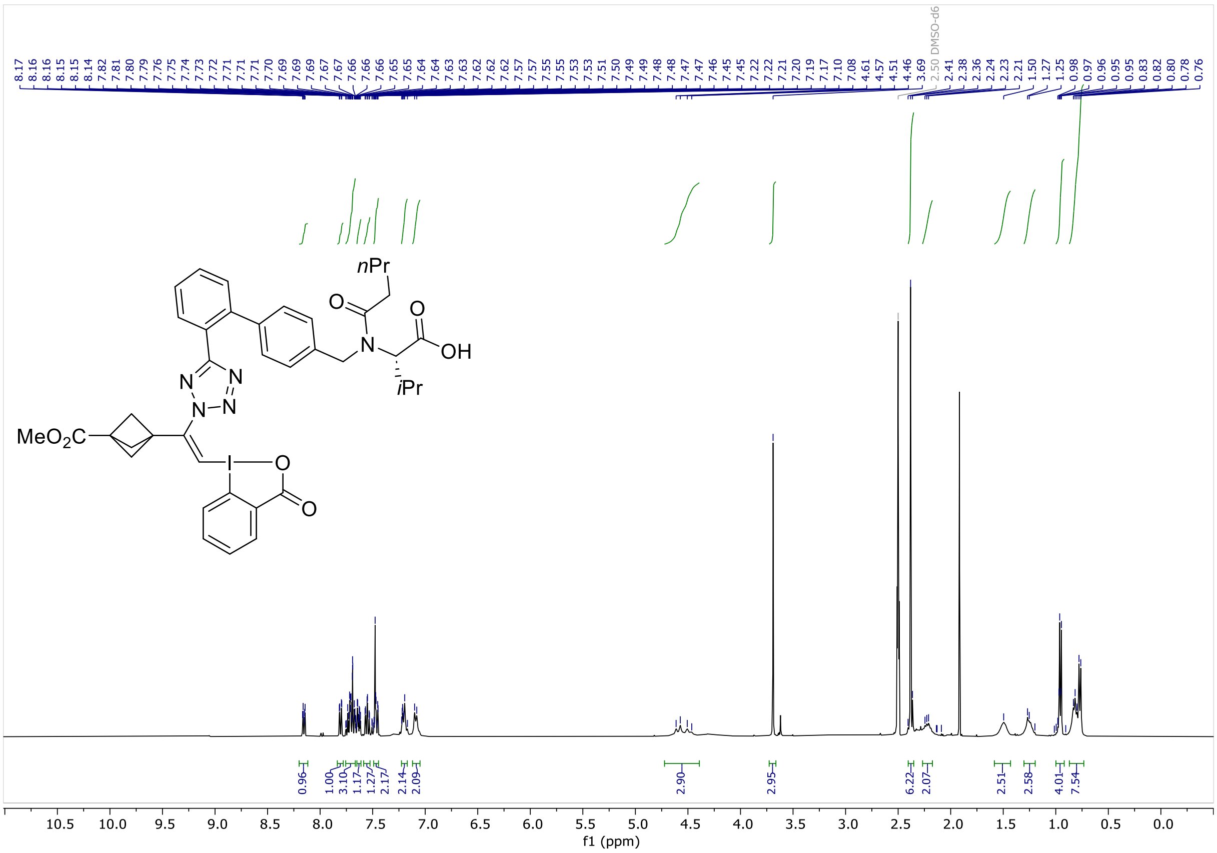

^{13}C NMR (101 MHz, $CDCl_3$) of compound **34** at 363 K

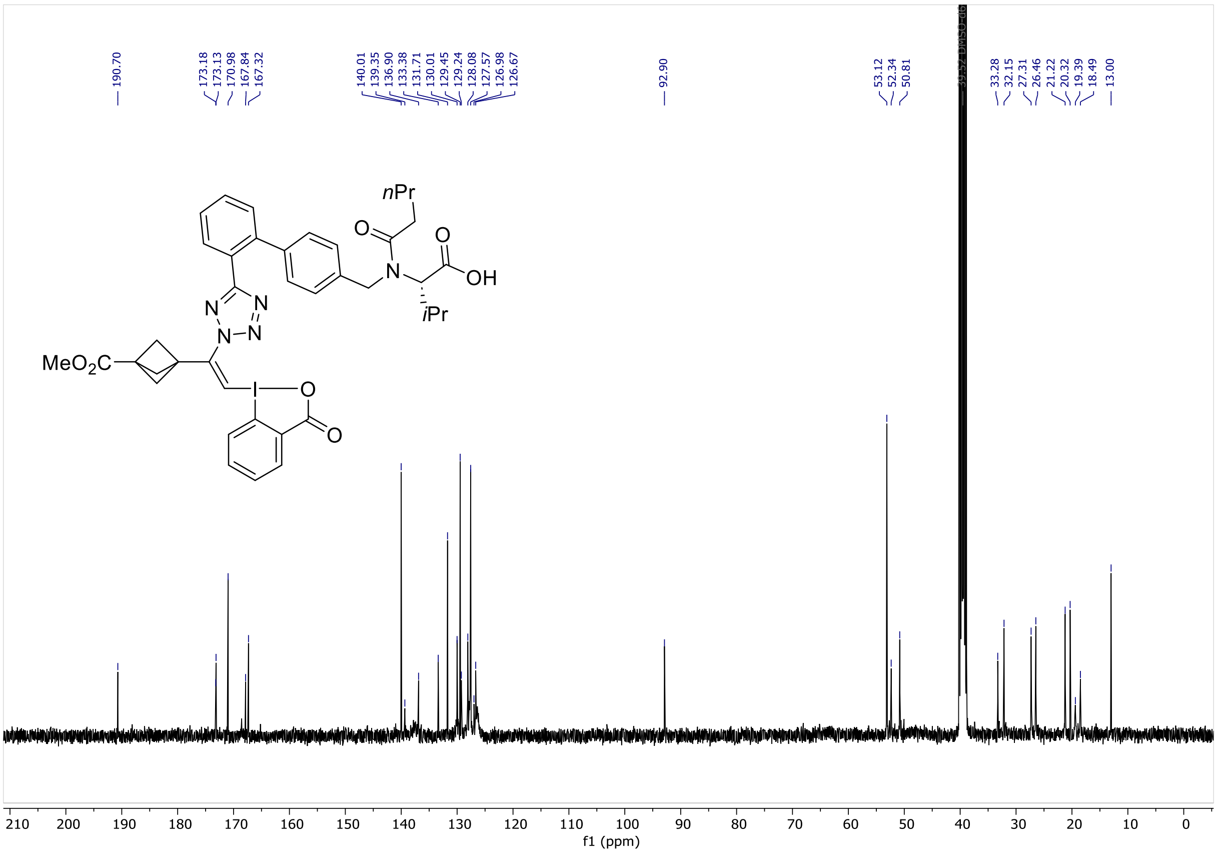

Temperature dependent **¹H NMR** (400 MHz, DMSO) of compound **34**

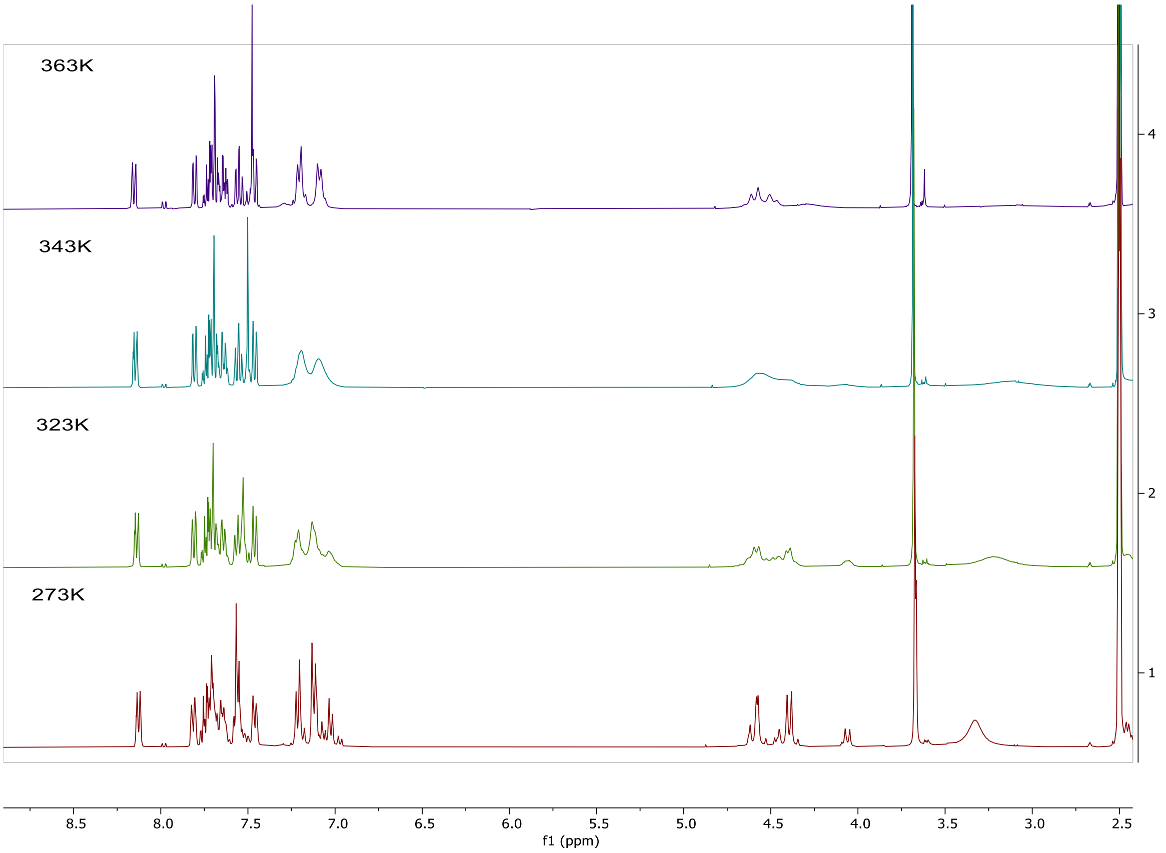

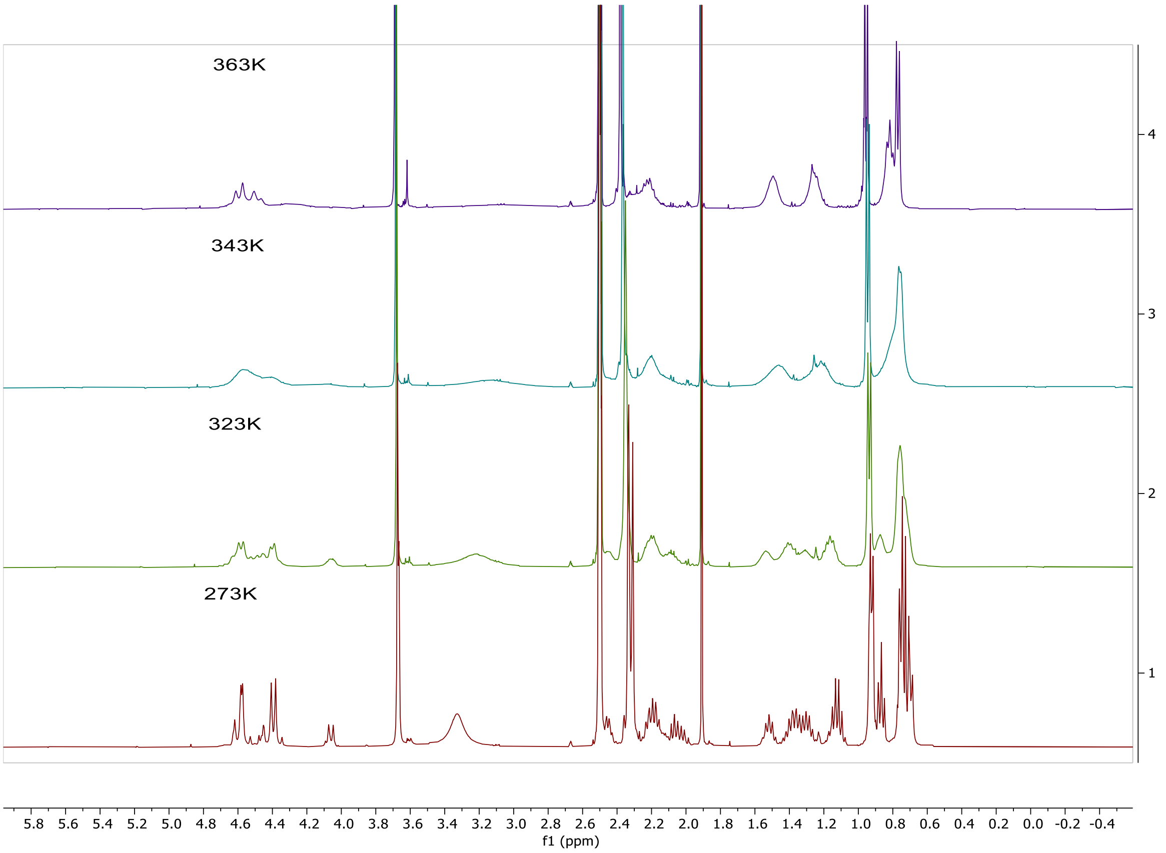

^{1}H NMR (400 MHz, $CDCl_3$) of compound **35a**

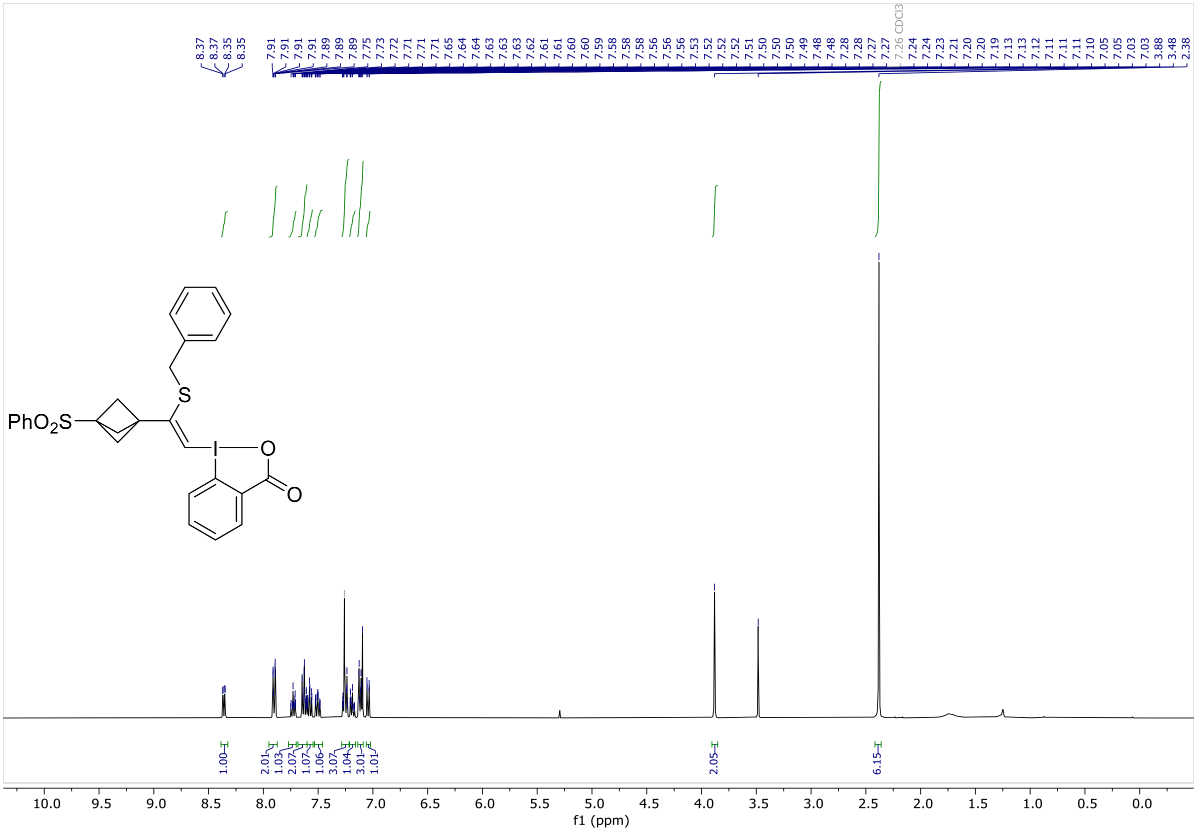

^{13}C NMR (101 MHz, $CDCl_3$) of compound **35a**

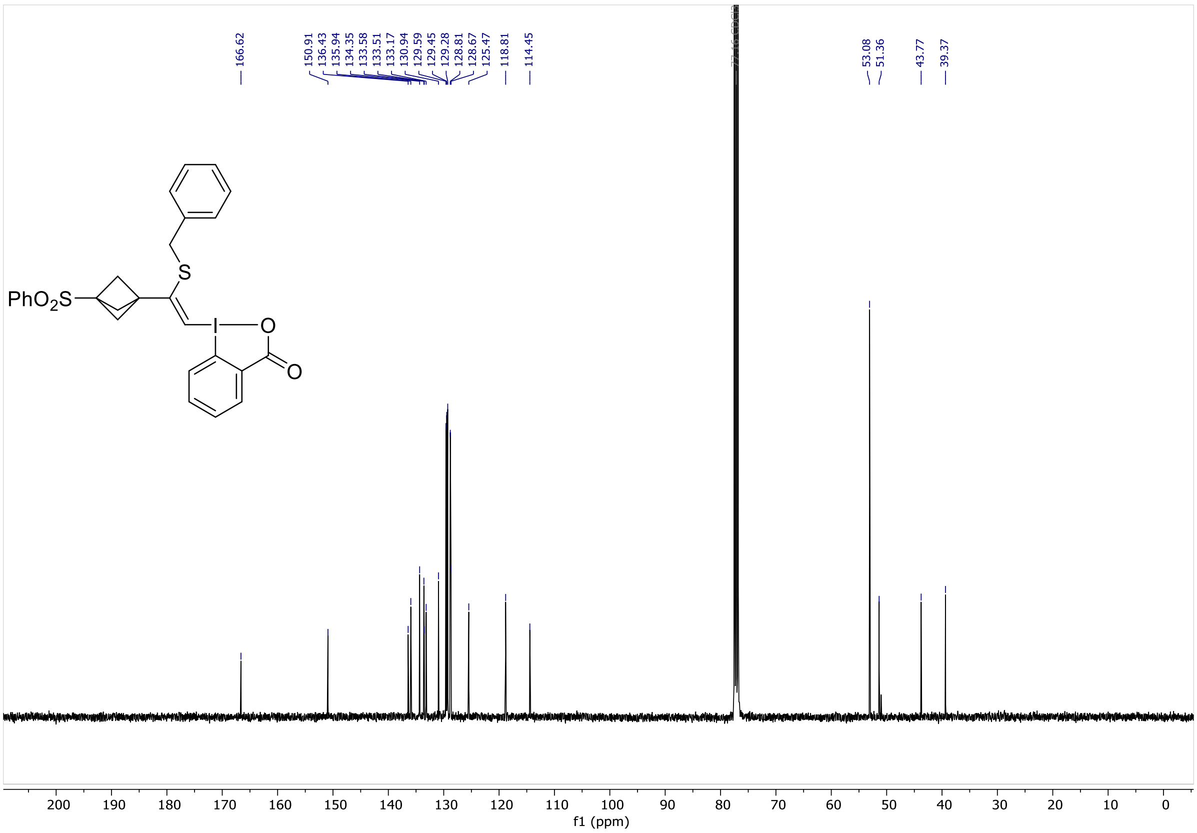

^{1}H NMR (400 MHz, $CDCl_3$) of compound **35b**

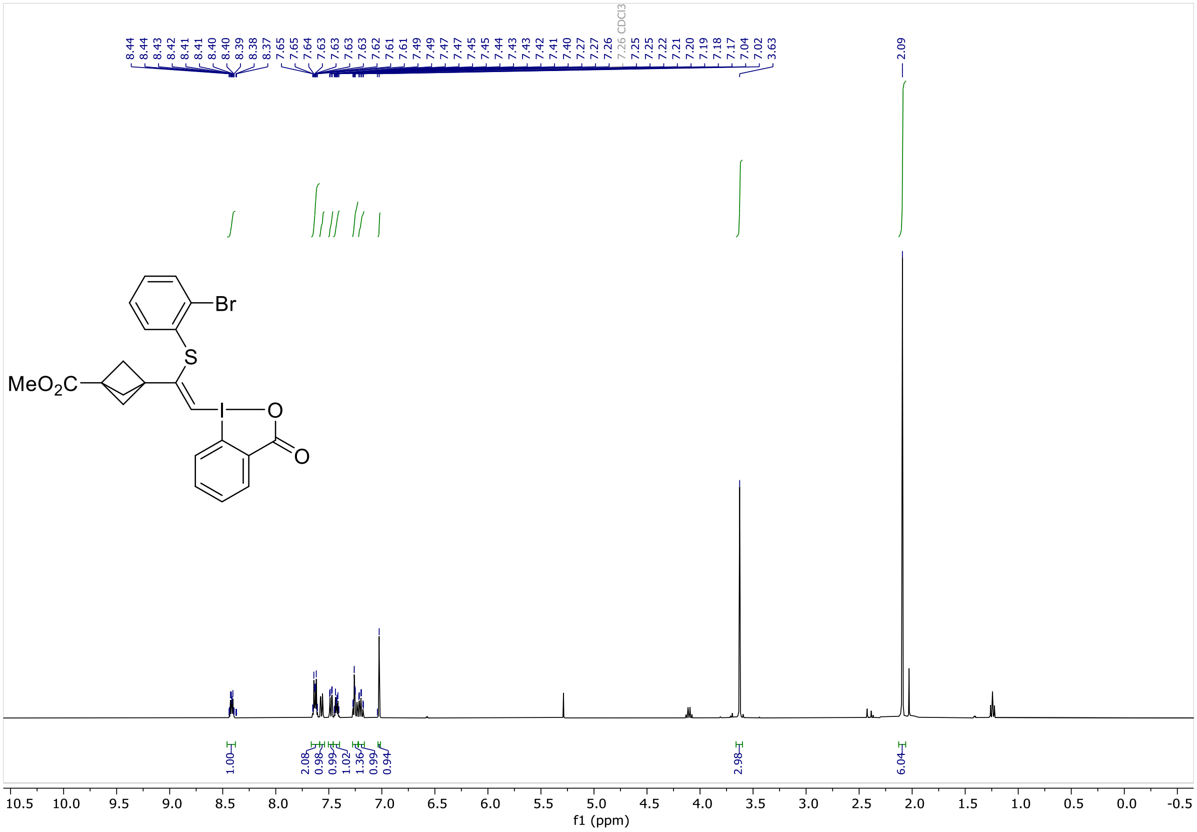

^{13}C NMR (101 MHz, $CDCl_3$) of compound **35b**

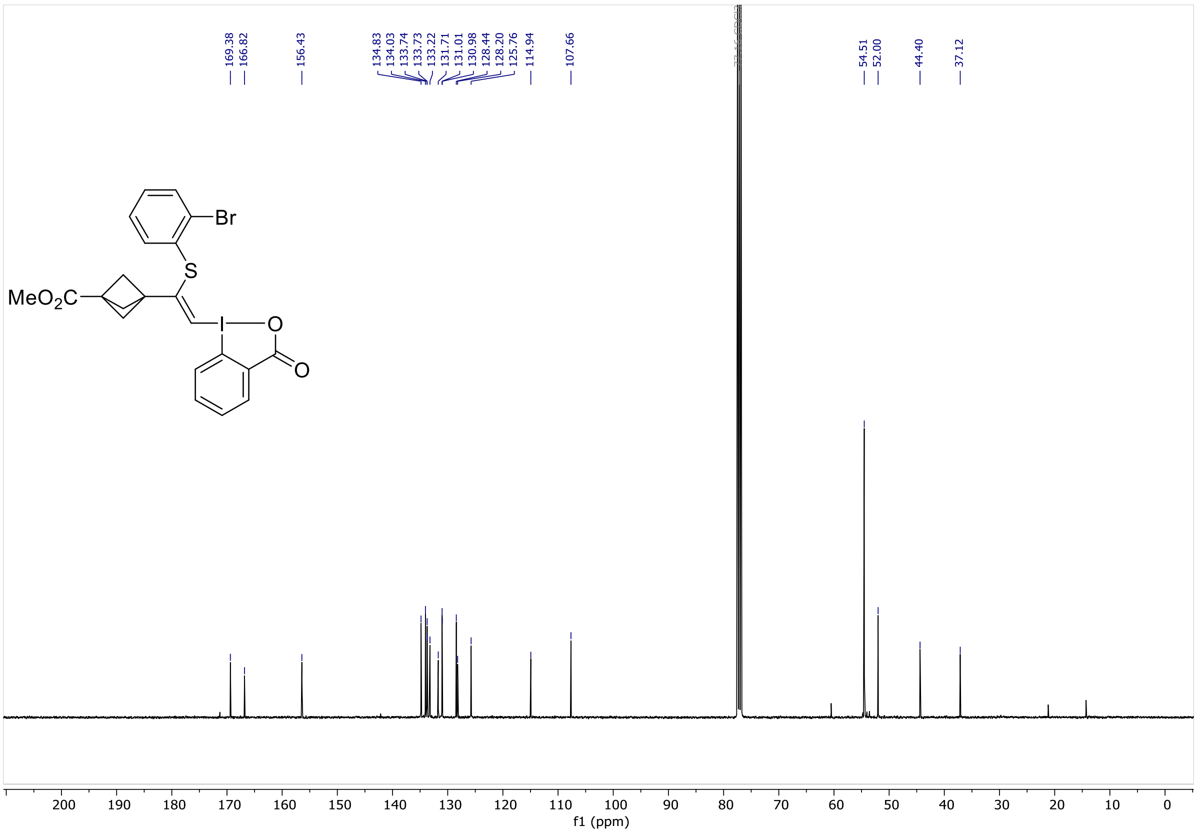

^{1}H NMR (400 MHz, $CDCl_3$) of compound **35c**

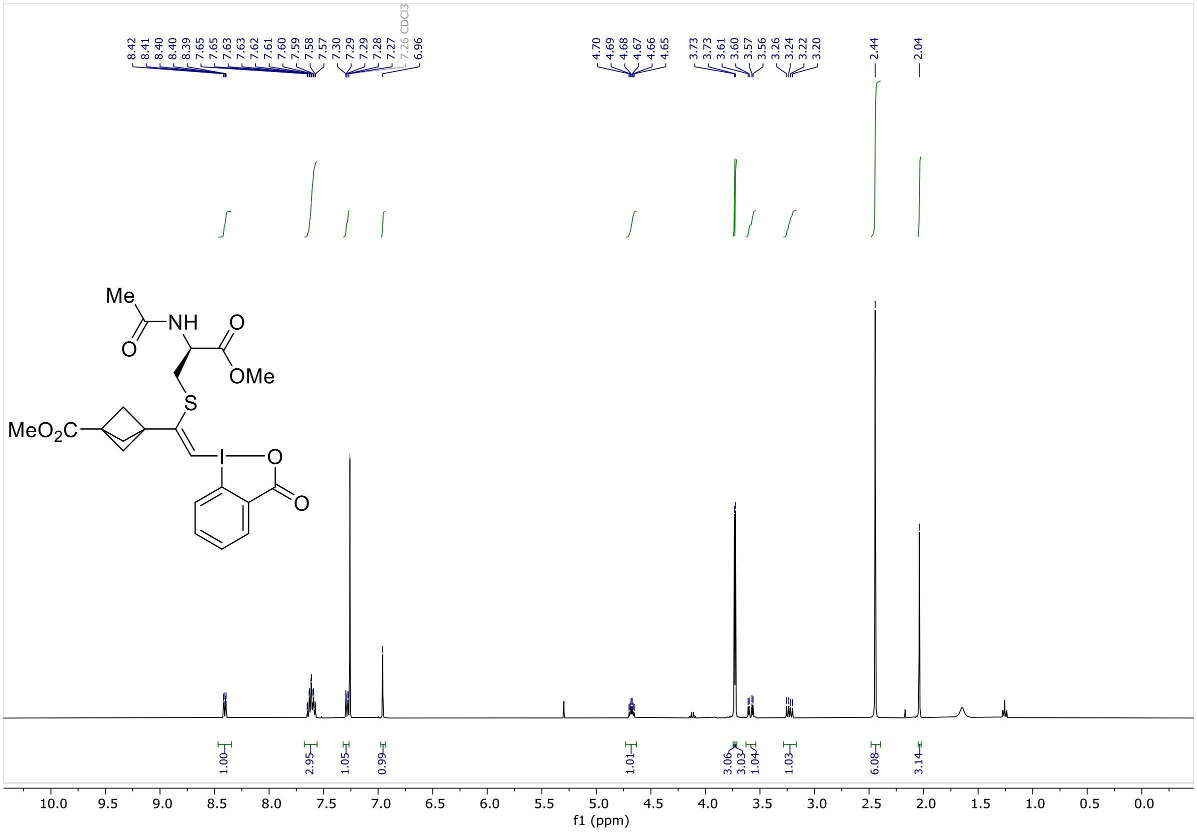

^{13}C NMR (101 MHz, $CDCl_3$) of compound **35c**

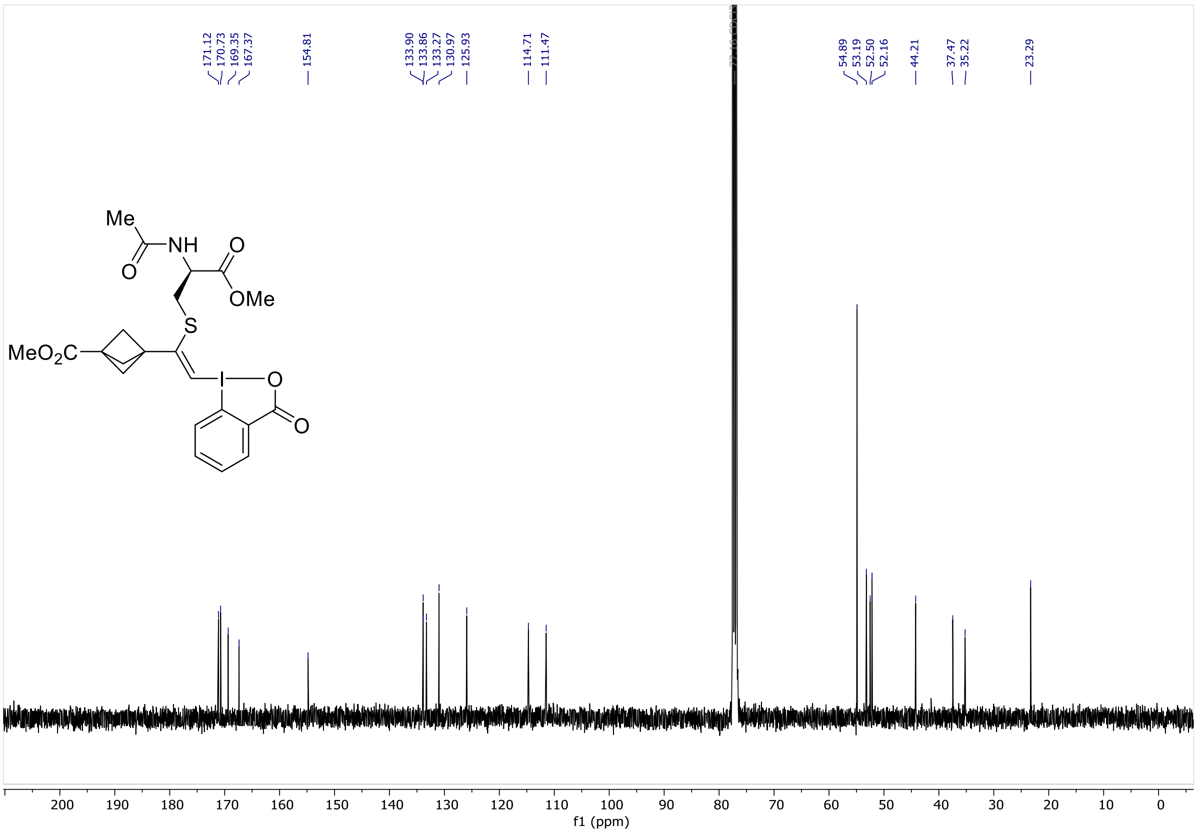

^{1}H NMR (400 MHz, $CDCl_3$) of compound **35d**

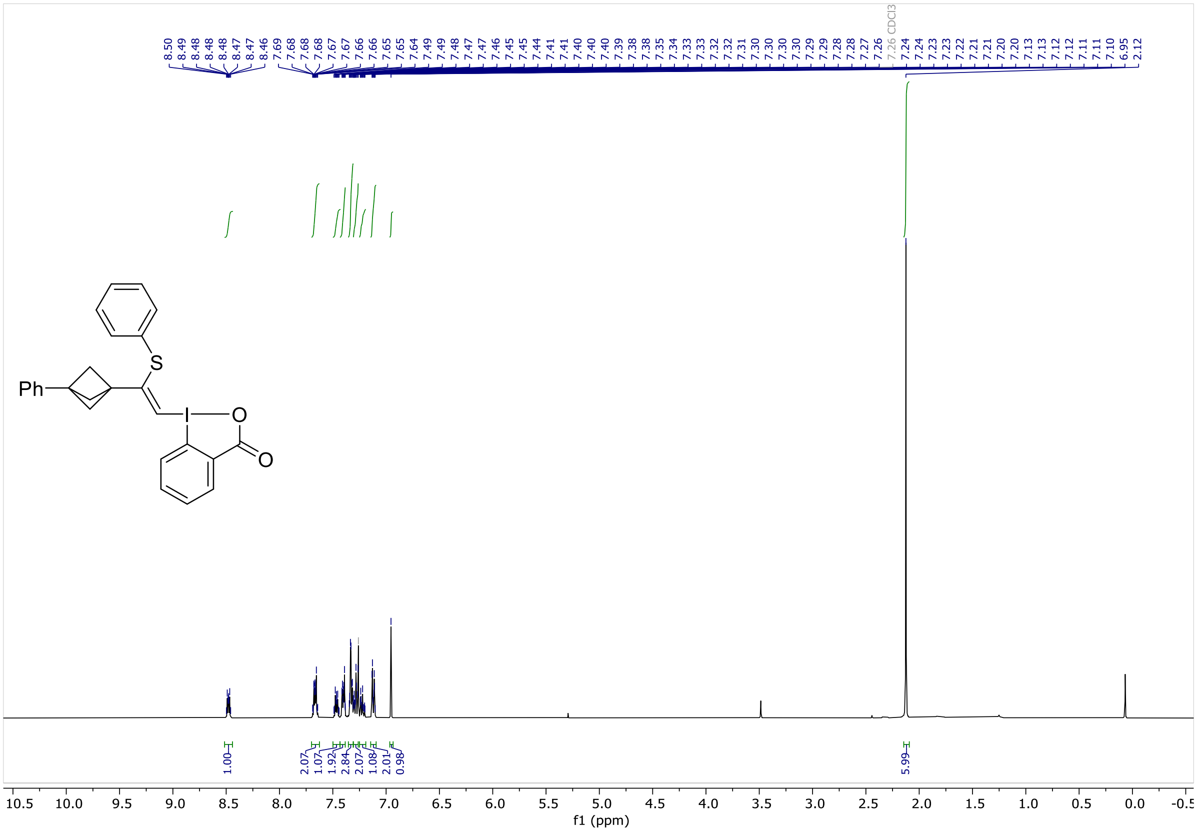

^{13}C NMR (101 MHz, $CDCl_3$) of compound **35d**

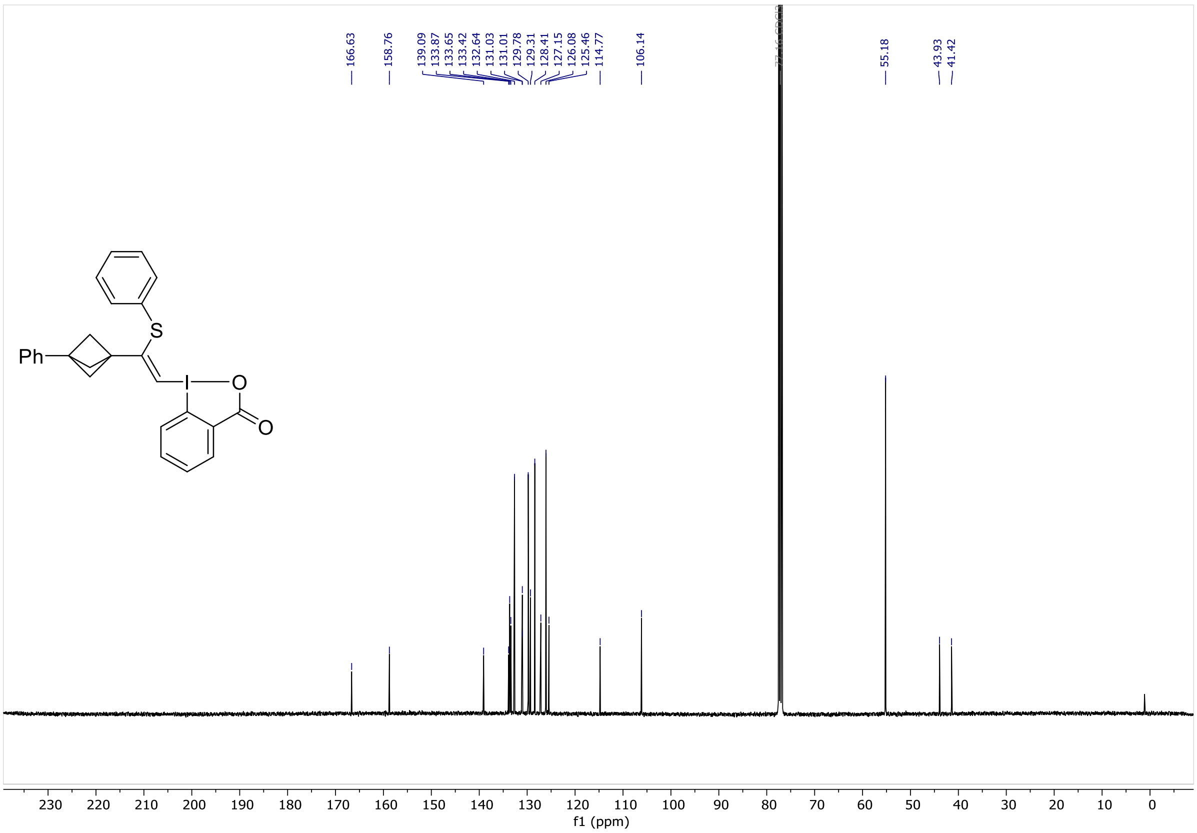

^{1}H NMR (400 MHz, $CDCl_3$) of compound **35e**

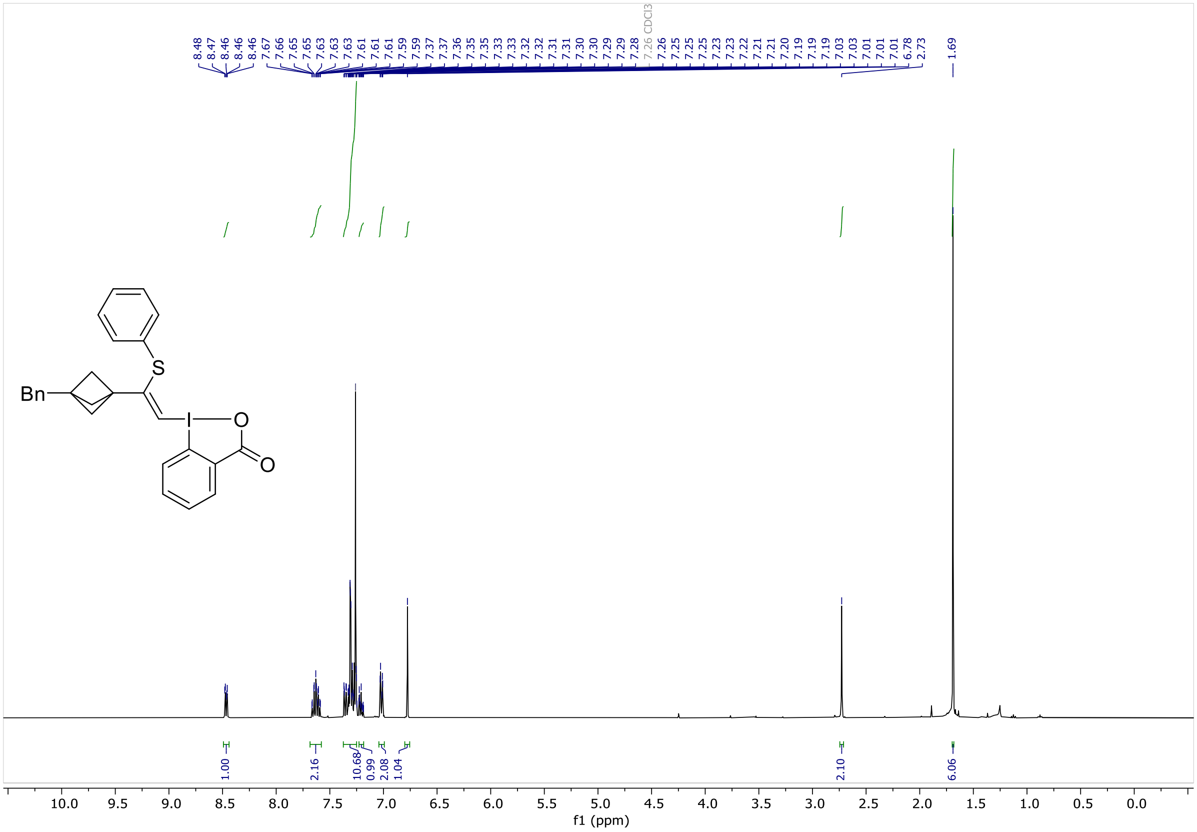

^{13}C NMR (101 MHz, $CDCl_3$) of compound **35e**

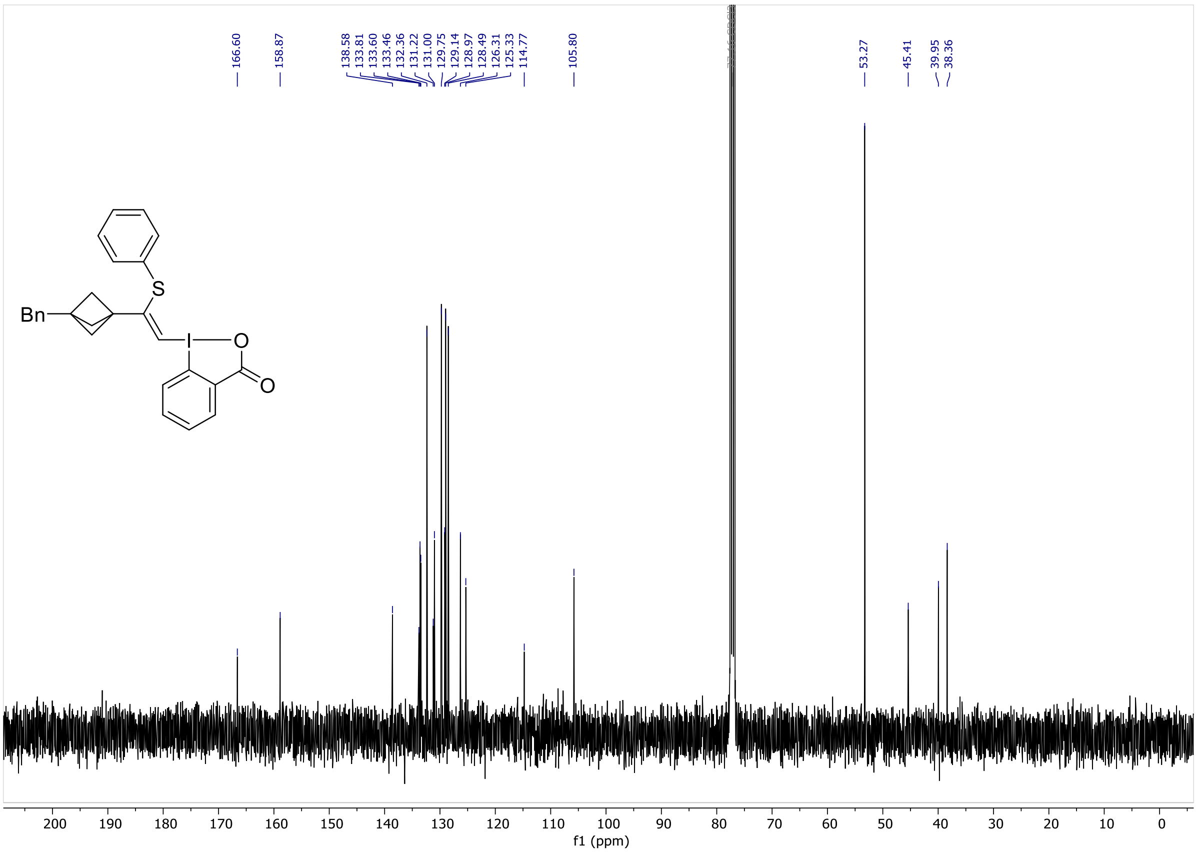

^{1}H **NMR** (400 MHz, $CDCl_3$) of compound **37a**

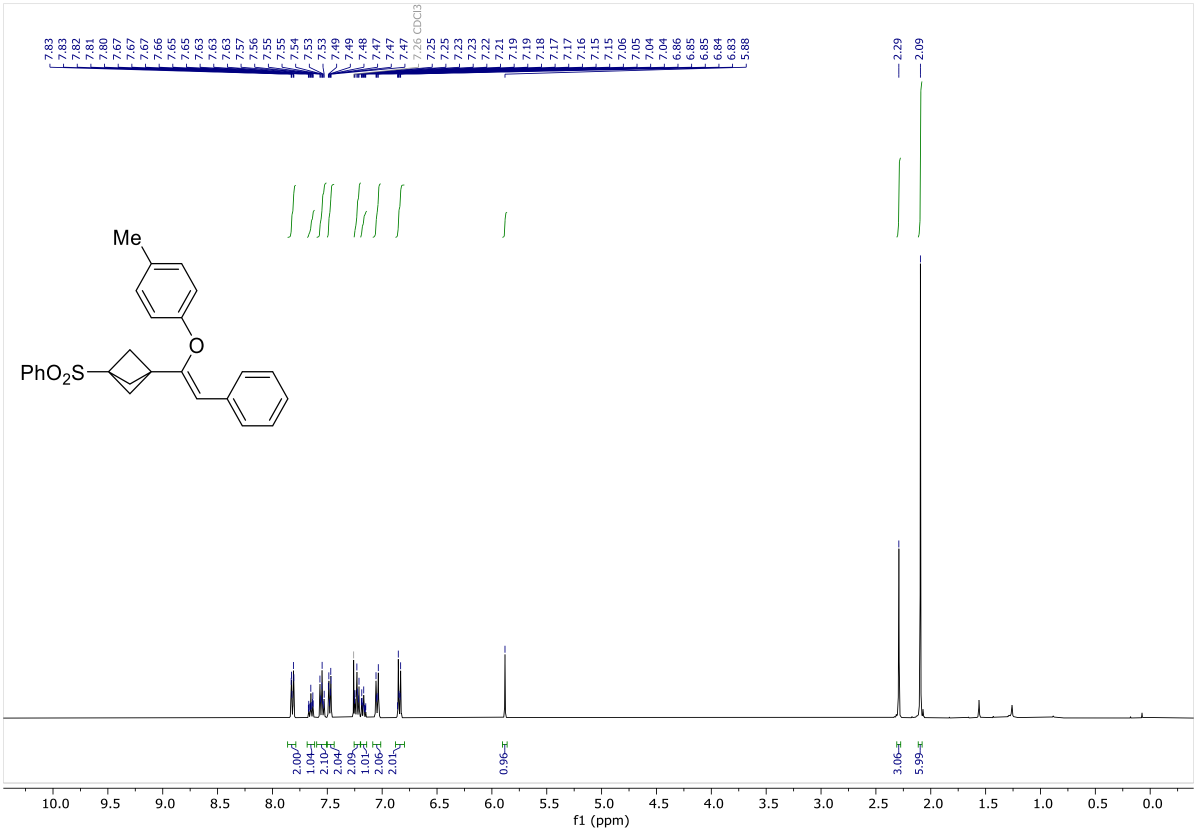

^{13}C **NMR** (101 MHz, $CDCl_3$) of compound **37a**

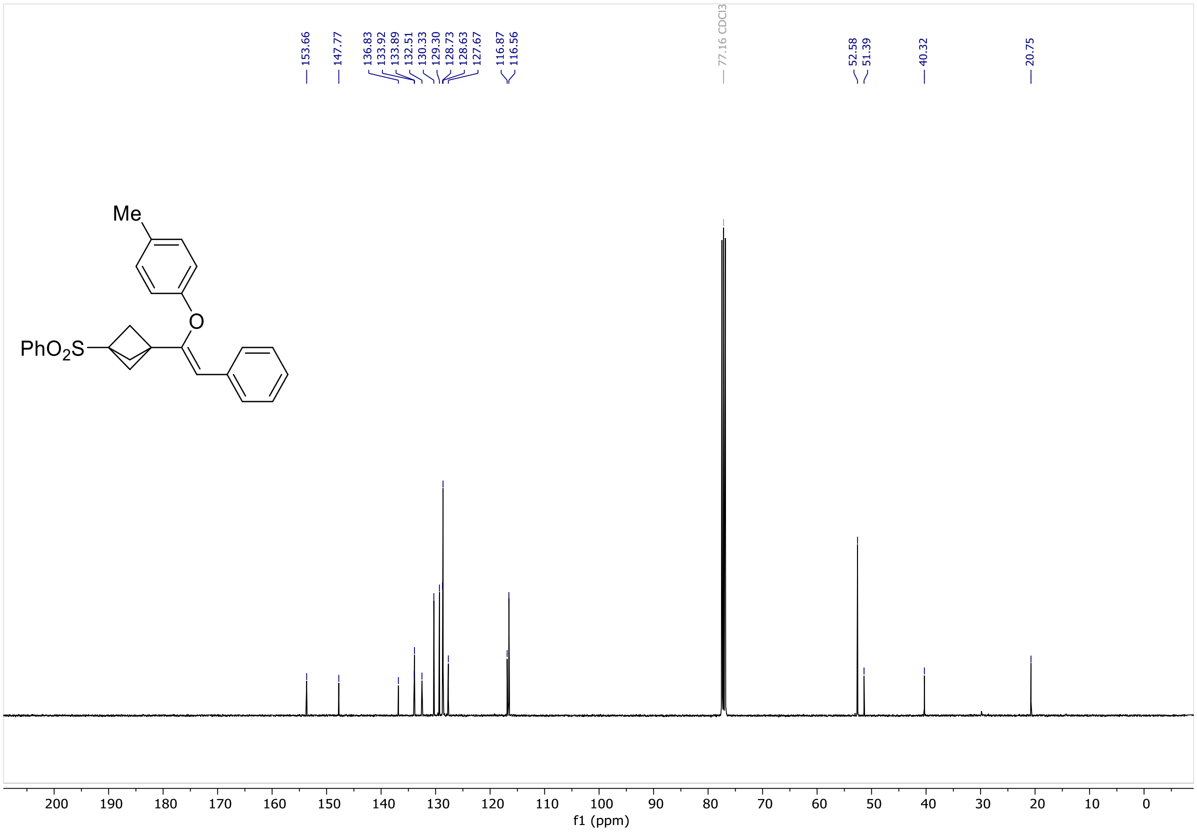

^{1}H NMR (400 MHz, $CDCl_3$) of compound **37b**

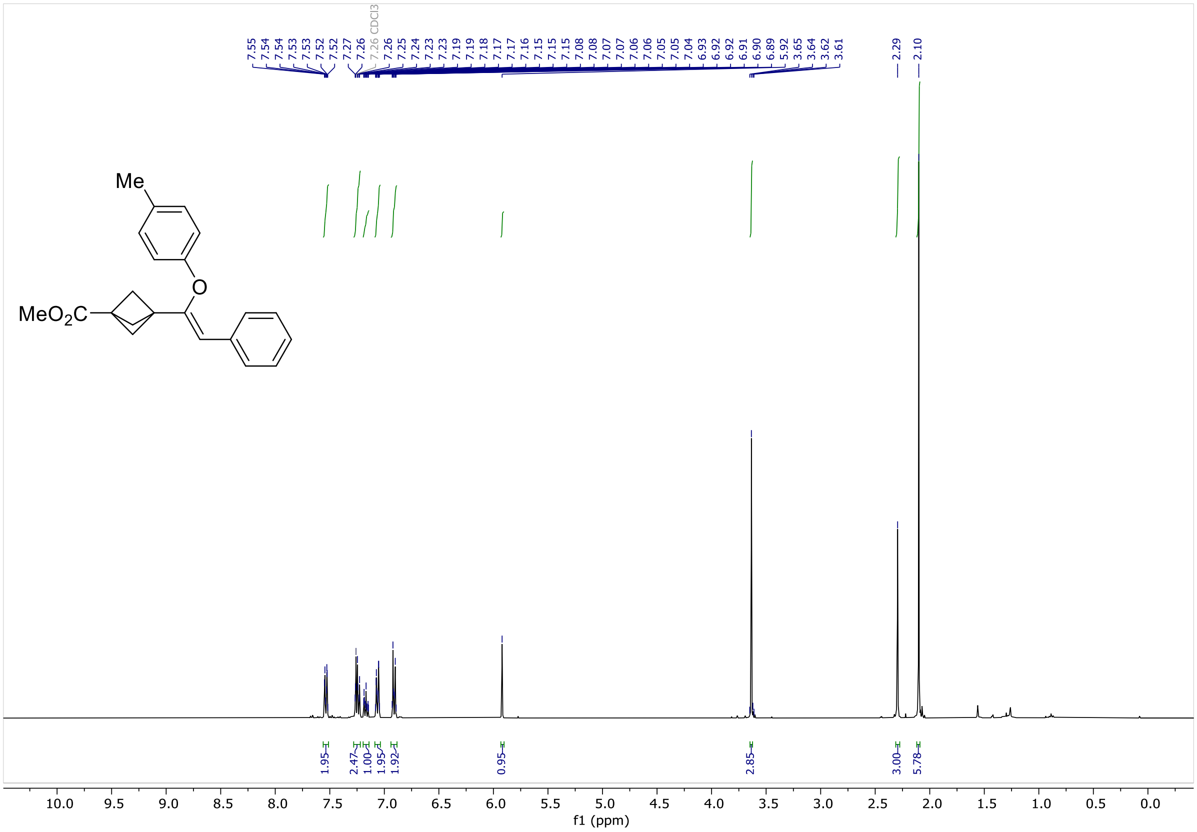

^{13}C NMR (101 MHz, $CDCl_3$) of compound **37b**

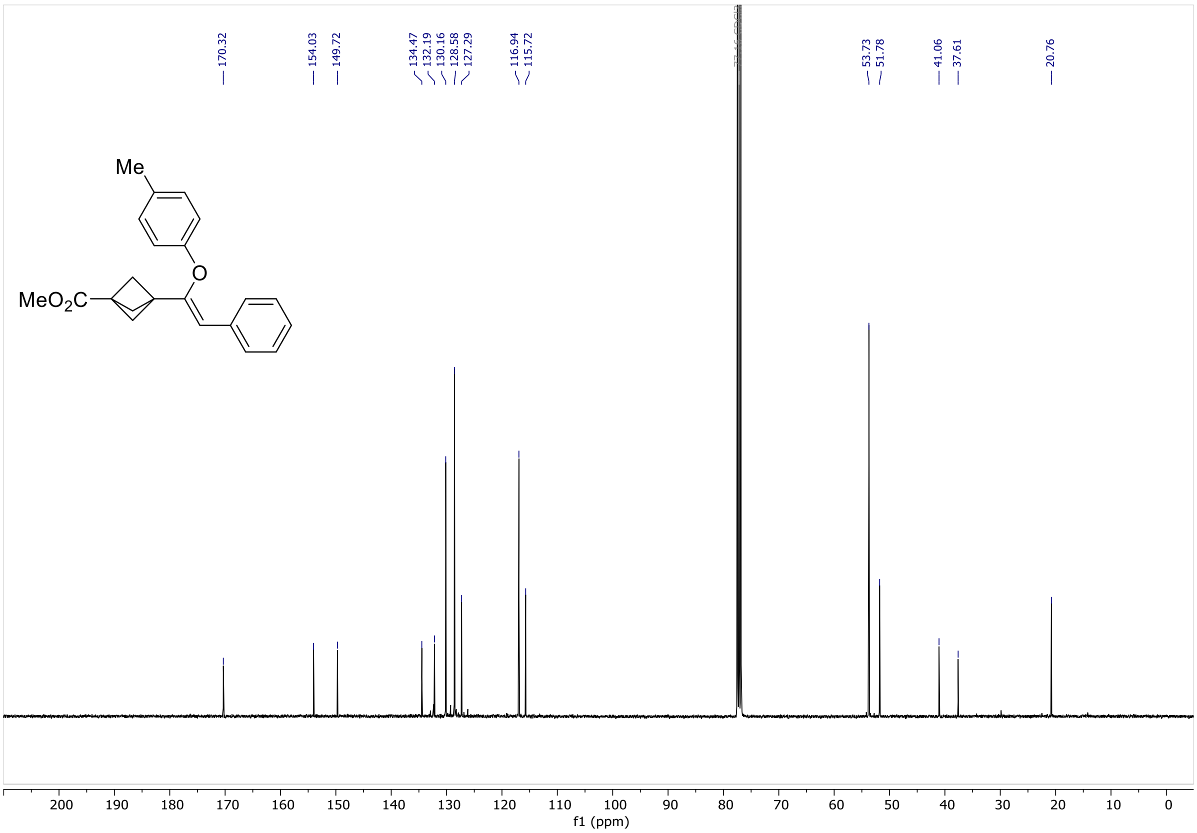

^{1}**H NMR** (400 MHz, $CDCl_3$) of compound **37c**

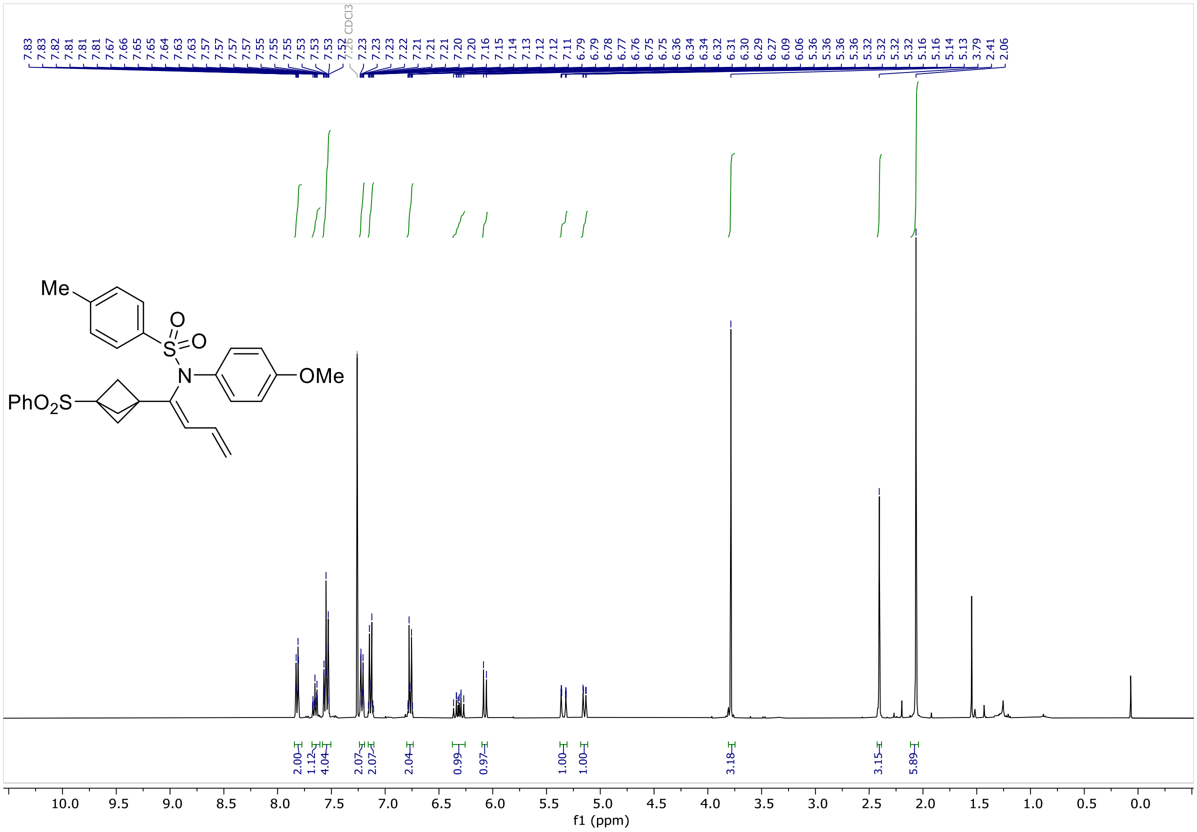

^{13}**C NMR** (101 MHz, $CDCl_3$) of compound **37c**

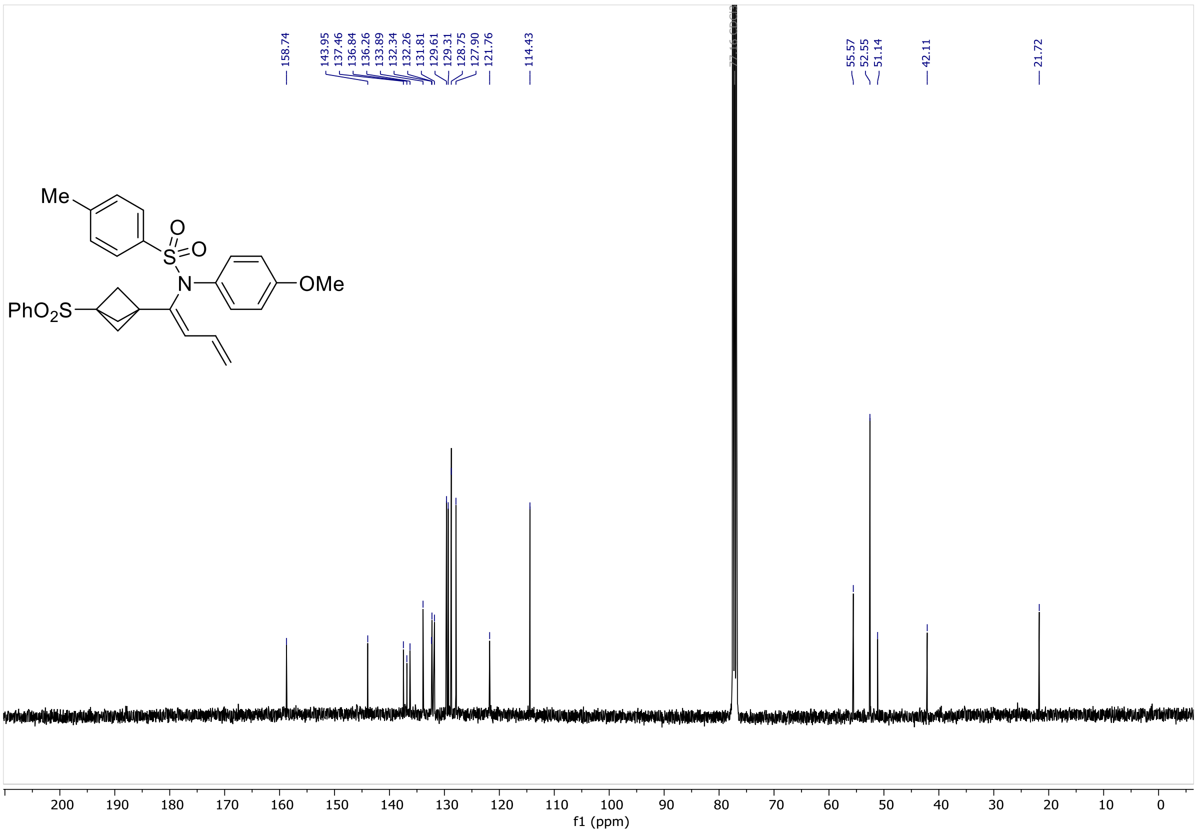

^{1}H NMR (400 MHz, $CDCl_3$) of compound **37d**; it was not possible to separate out from some impurity.

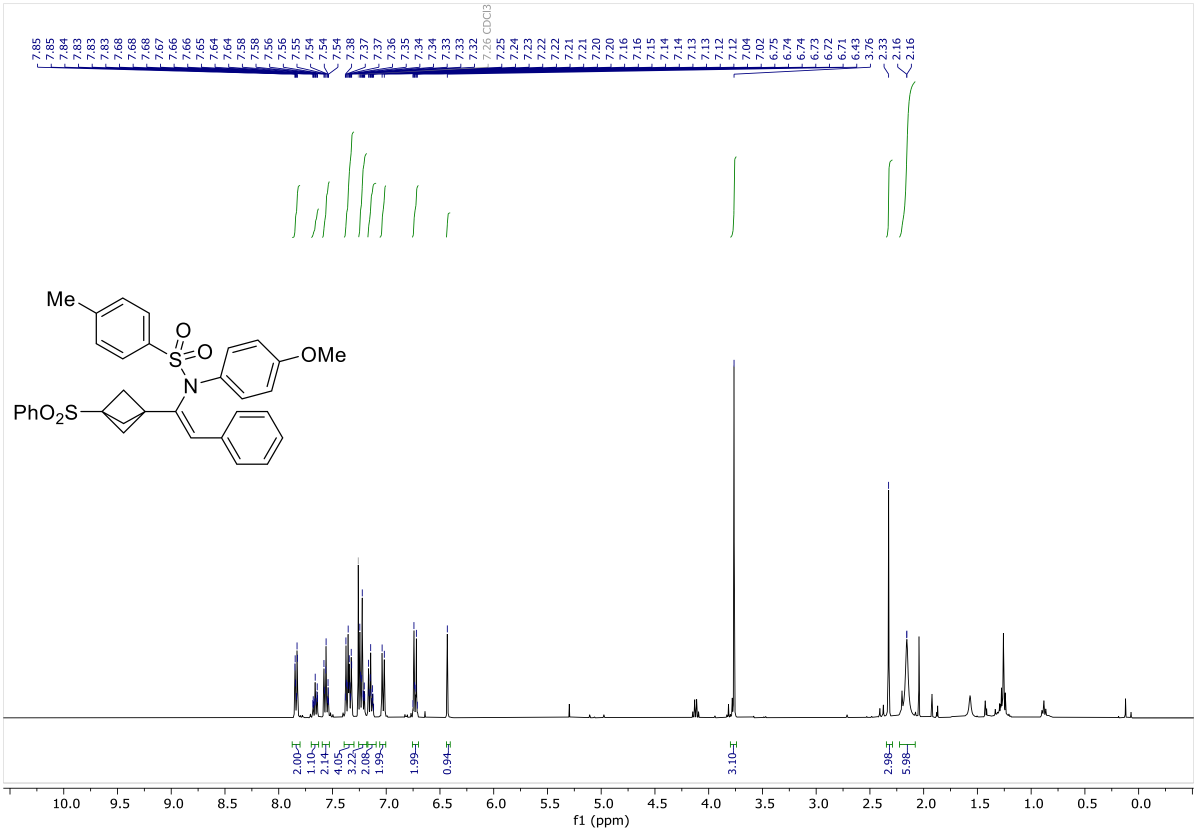

^{13}C NMR (101 MHz, $CDCl_3$) of compound **37d**; it was not possible to separate out from some impurity

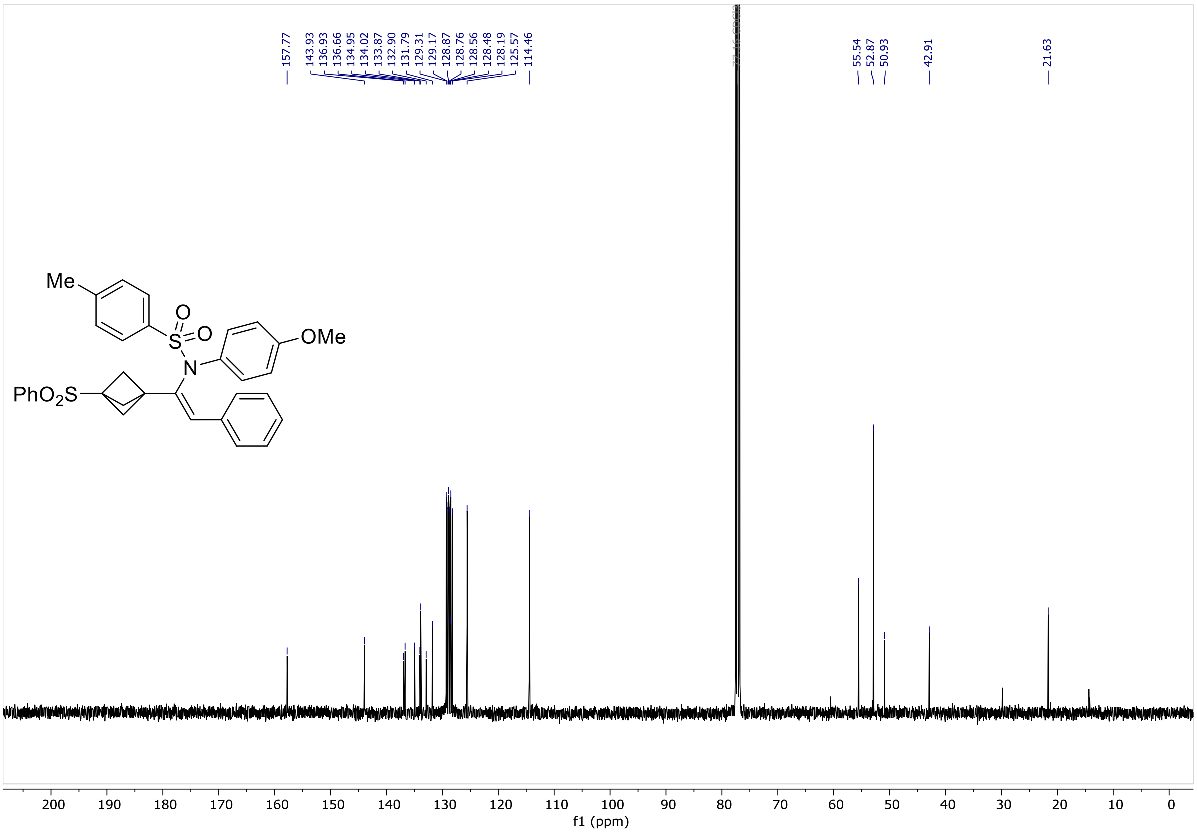

^{1}H NMR (400 MHz, $CDCl_3$) of compound **37e**

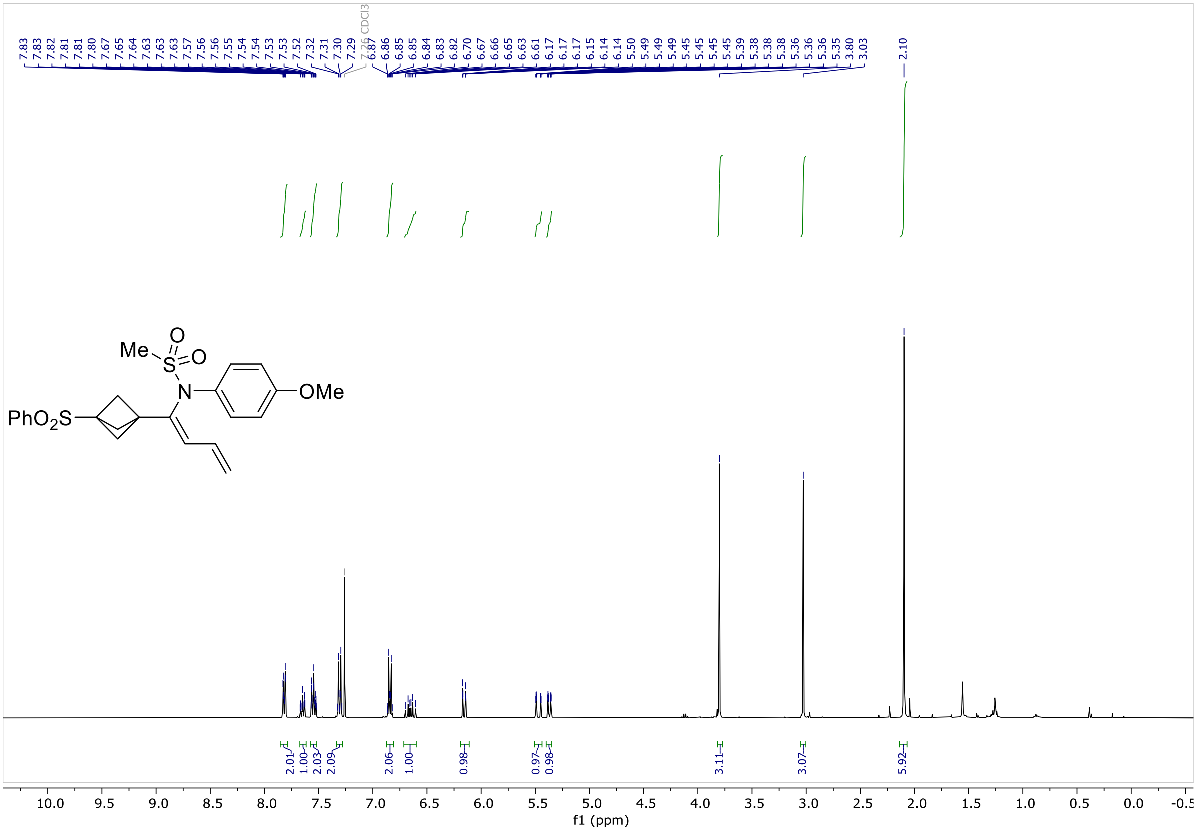

^{13}C NMR (101 MHz, $CDCl_3$) of compound **37e**

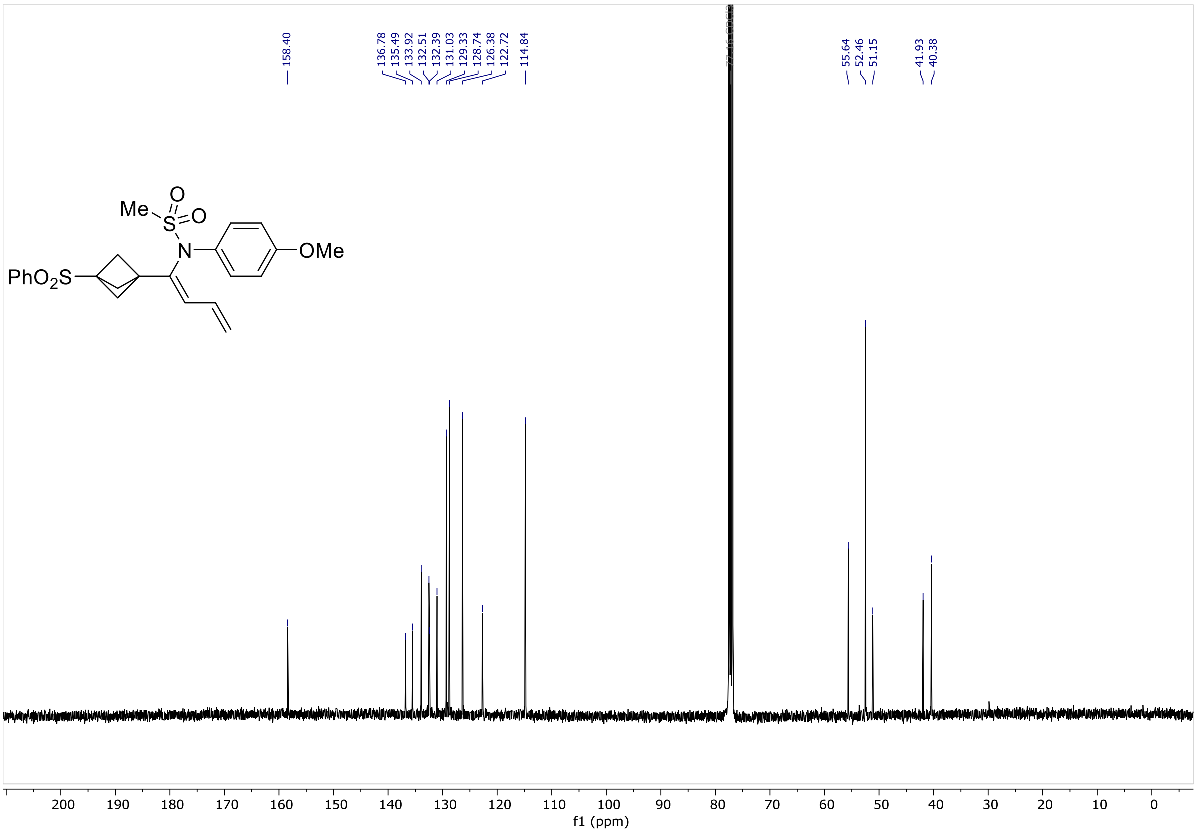

^{1}H NMR (400 MHz, $CDCl_3$) of compound **37f**

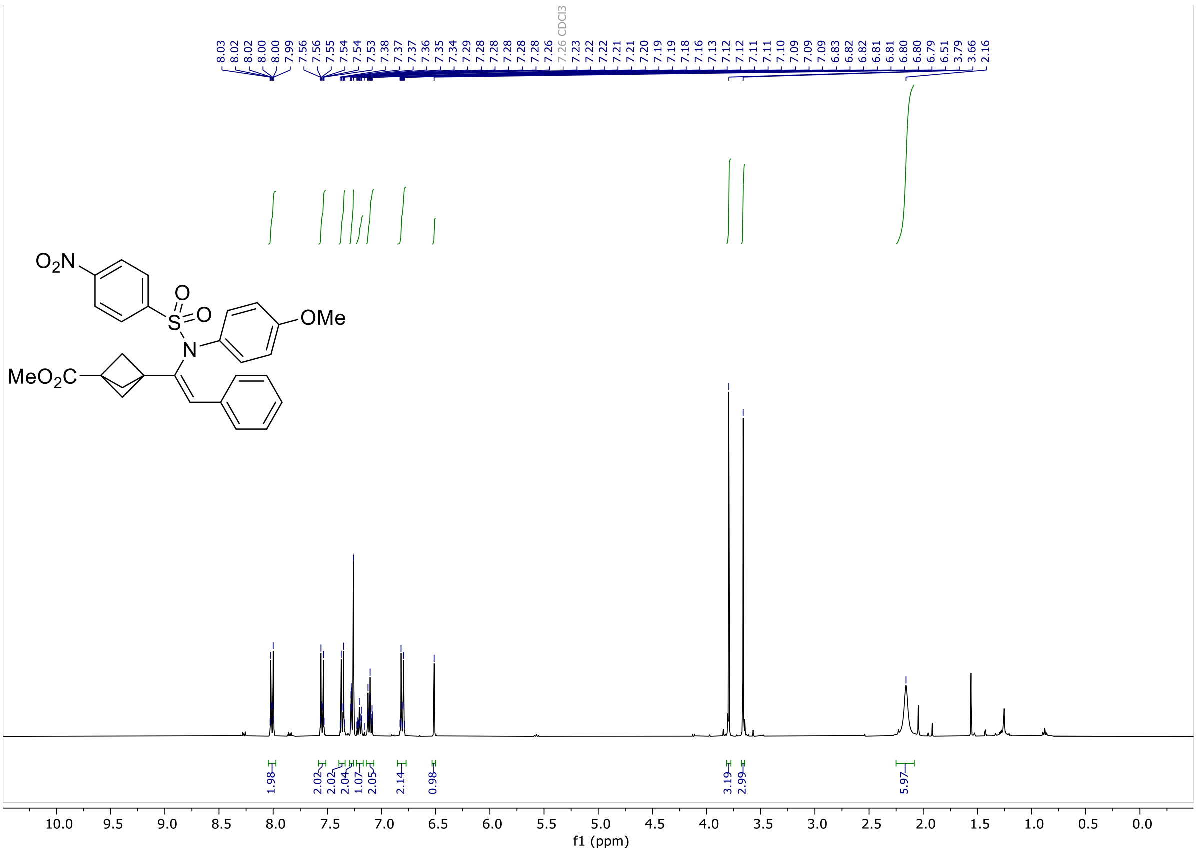

^{13}C NMR (101 MHz, $CDCl_3$) of compound **37f**

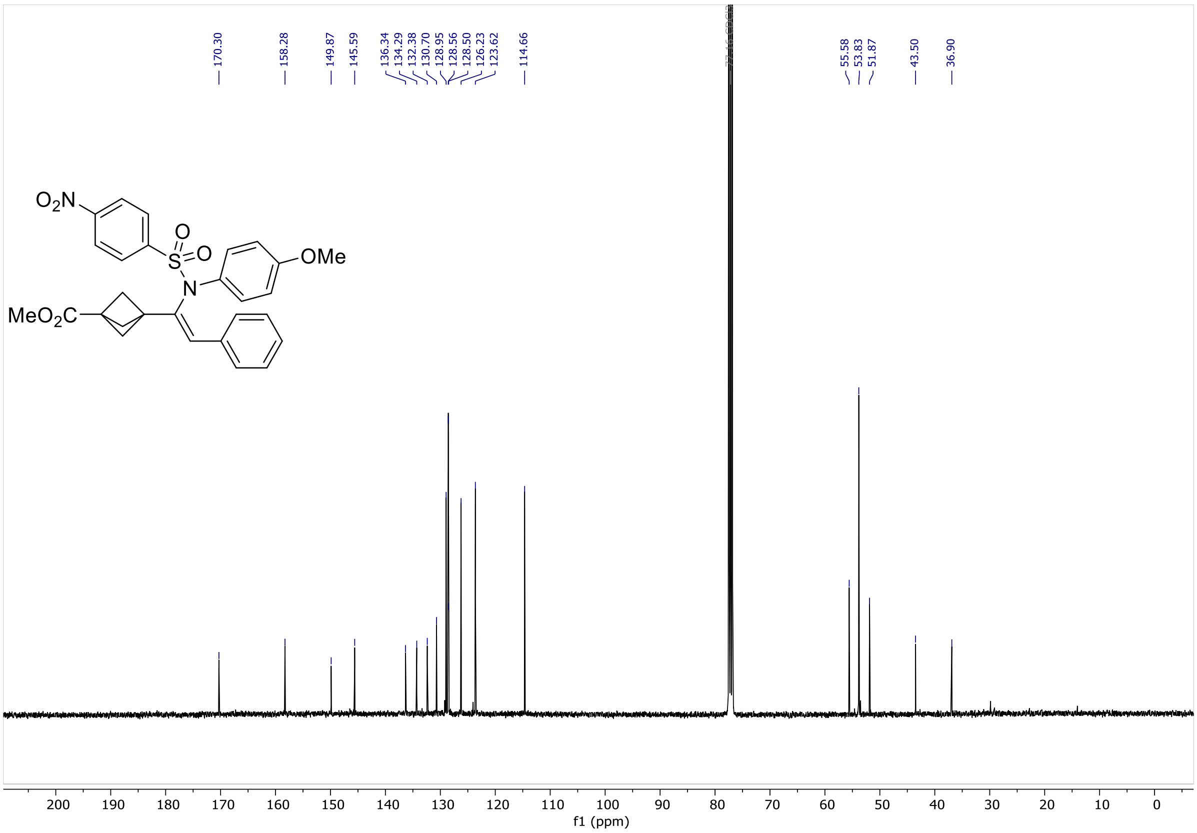

[1]**H NMR** (400 MHz, $CDCl_3$) of compound **37g**

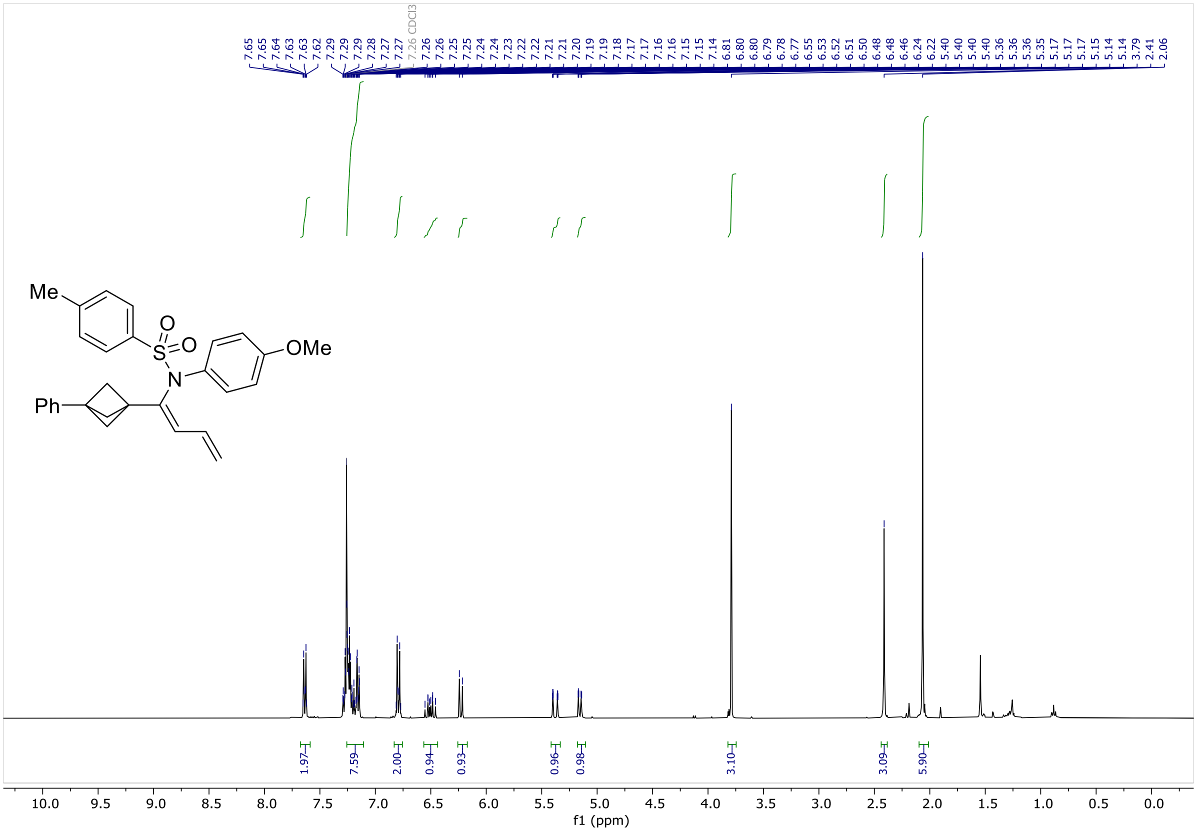

[13]**C NMR** (101 MHz, $CDCl_3$) of compound **37g**

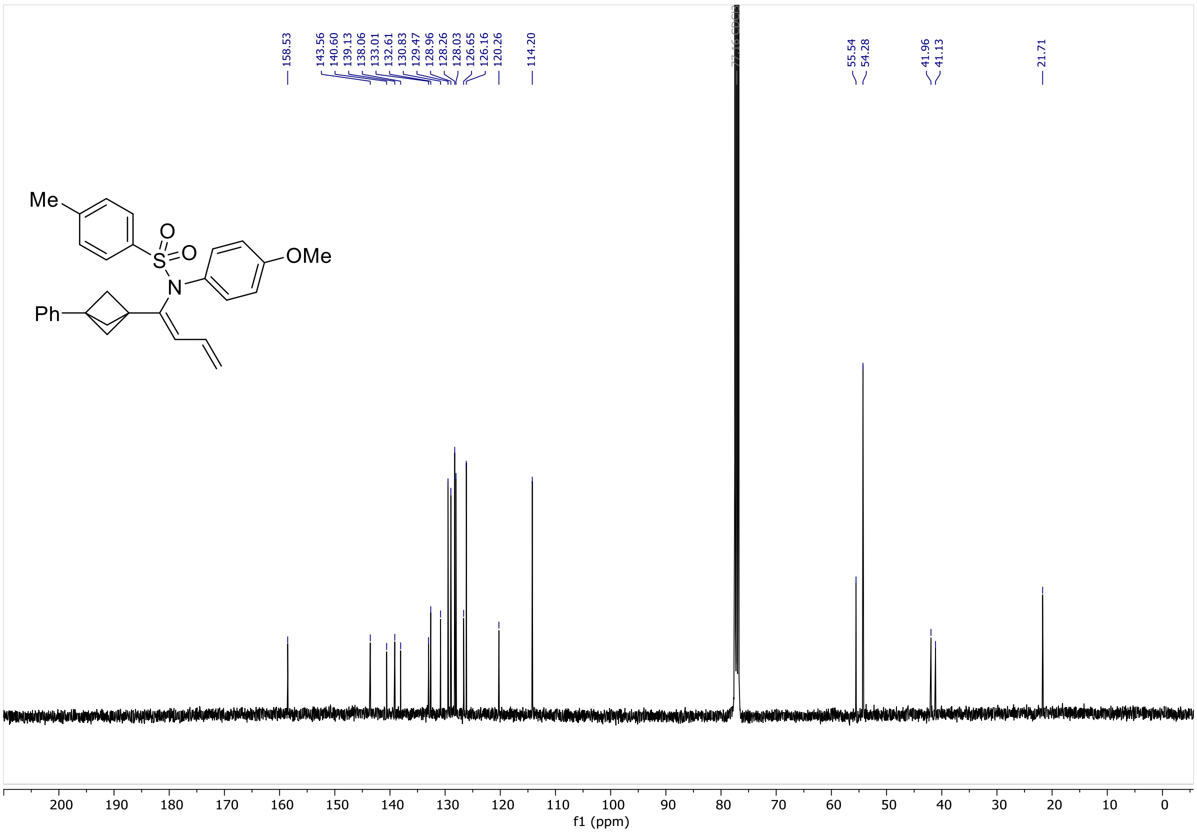

^{1}**H NMR** (400 MHz, $CDCl_3$) of compound **37h**

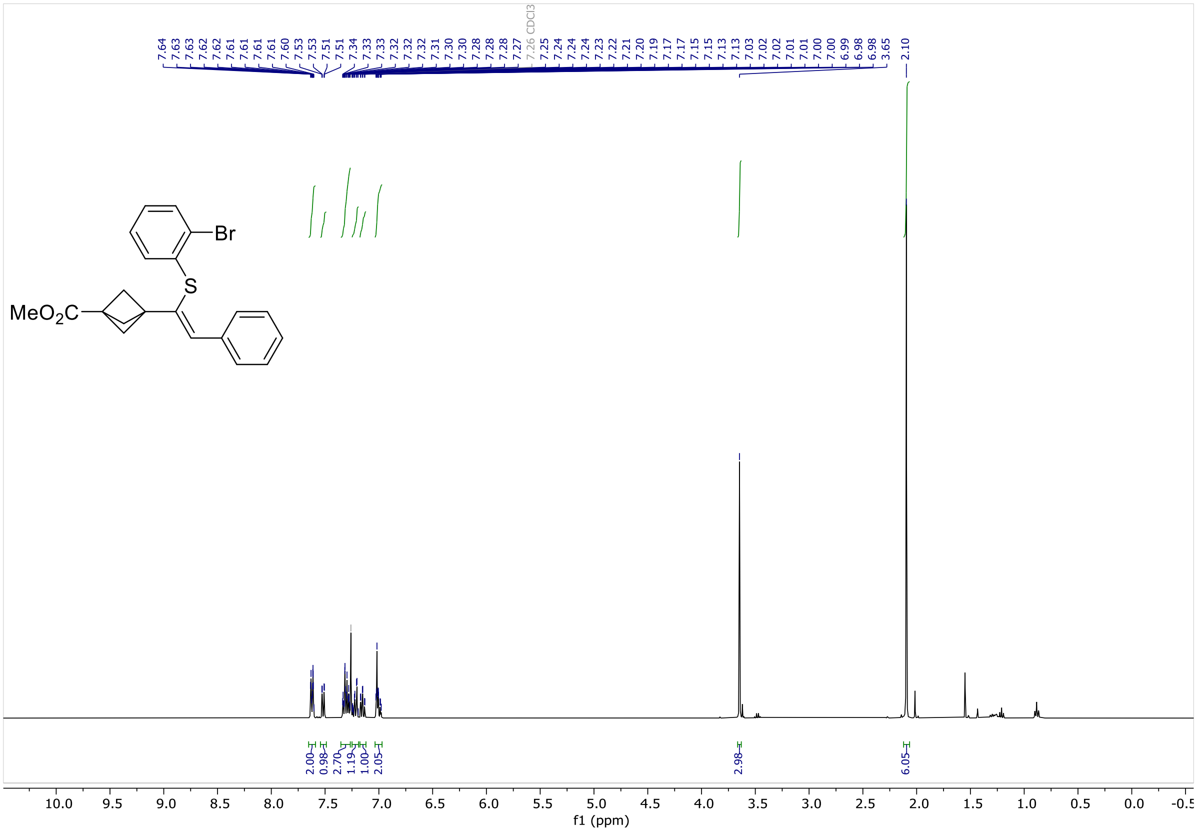

^{13}**C NMR** (101 MHz, $CDCl_3$) of compound **37h**

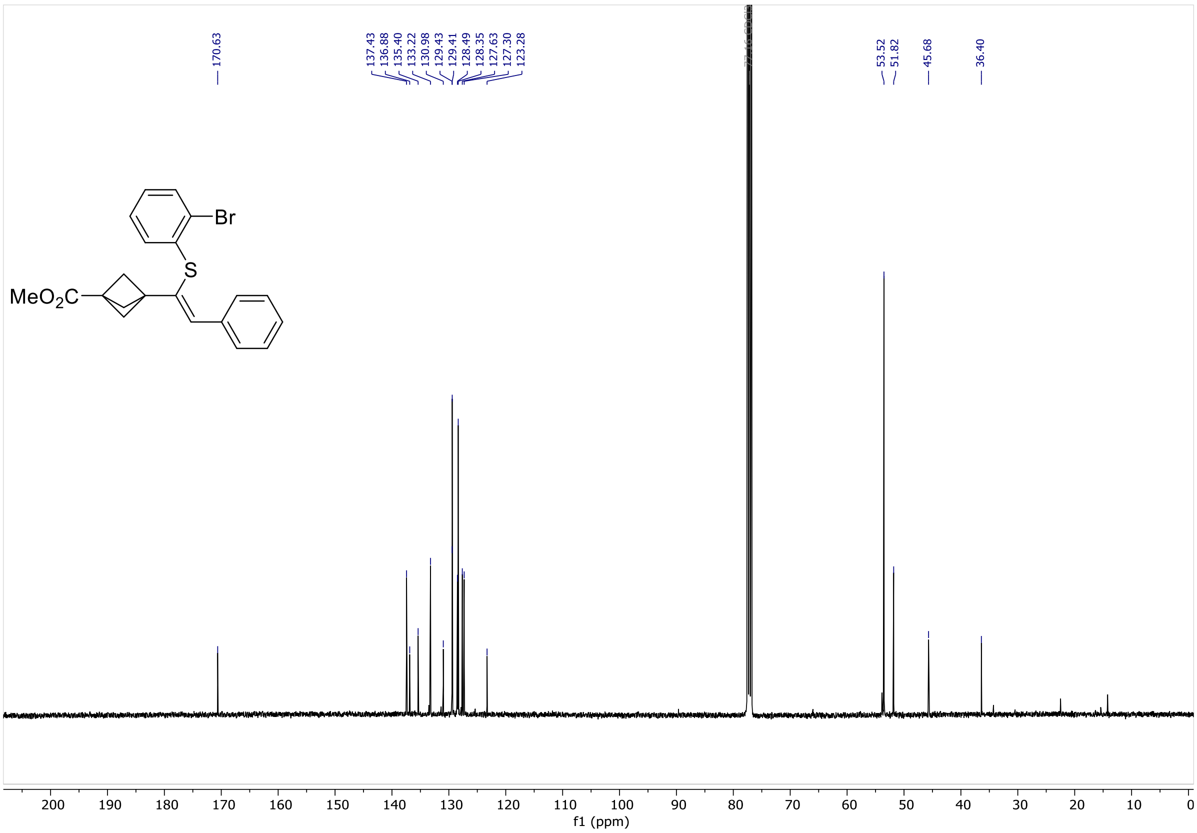

[1]**H NMR** (400 MHz, CDCl$_3$) of compound **37i**; other signals are from E isomer

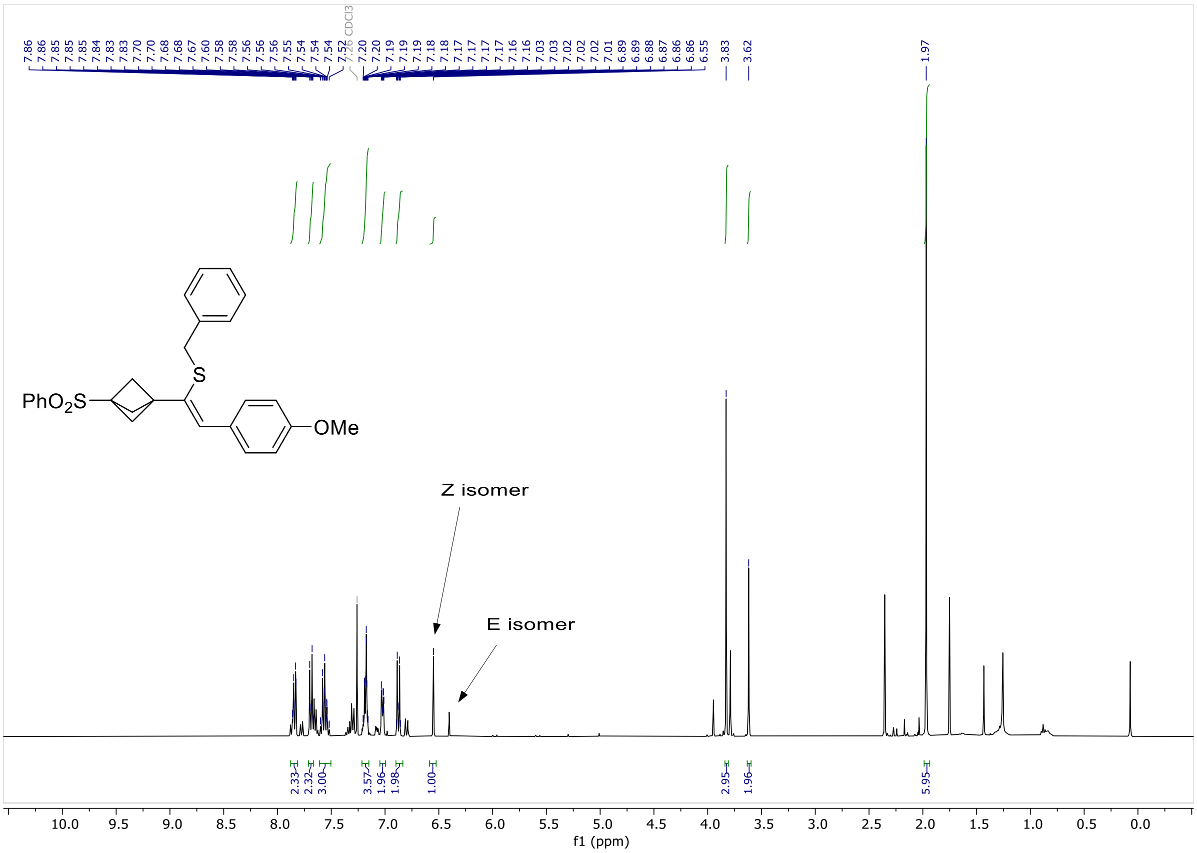

[13]**C NMR** (101 MHz, CDCl$_3$) of compound **37i**; other signals are from E isomer

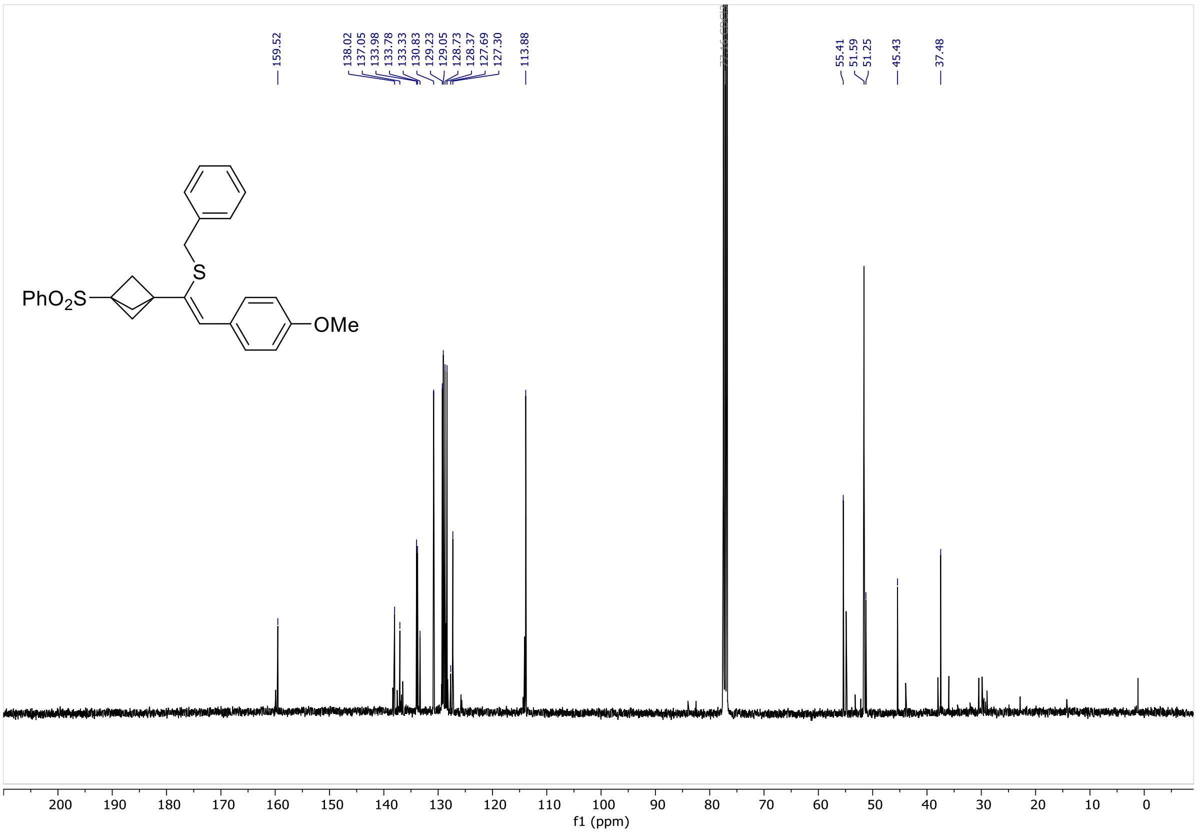

^{1}H NMR (400 MHz, $CDCl_3$) of compound **38a**

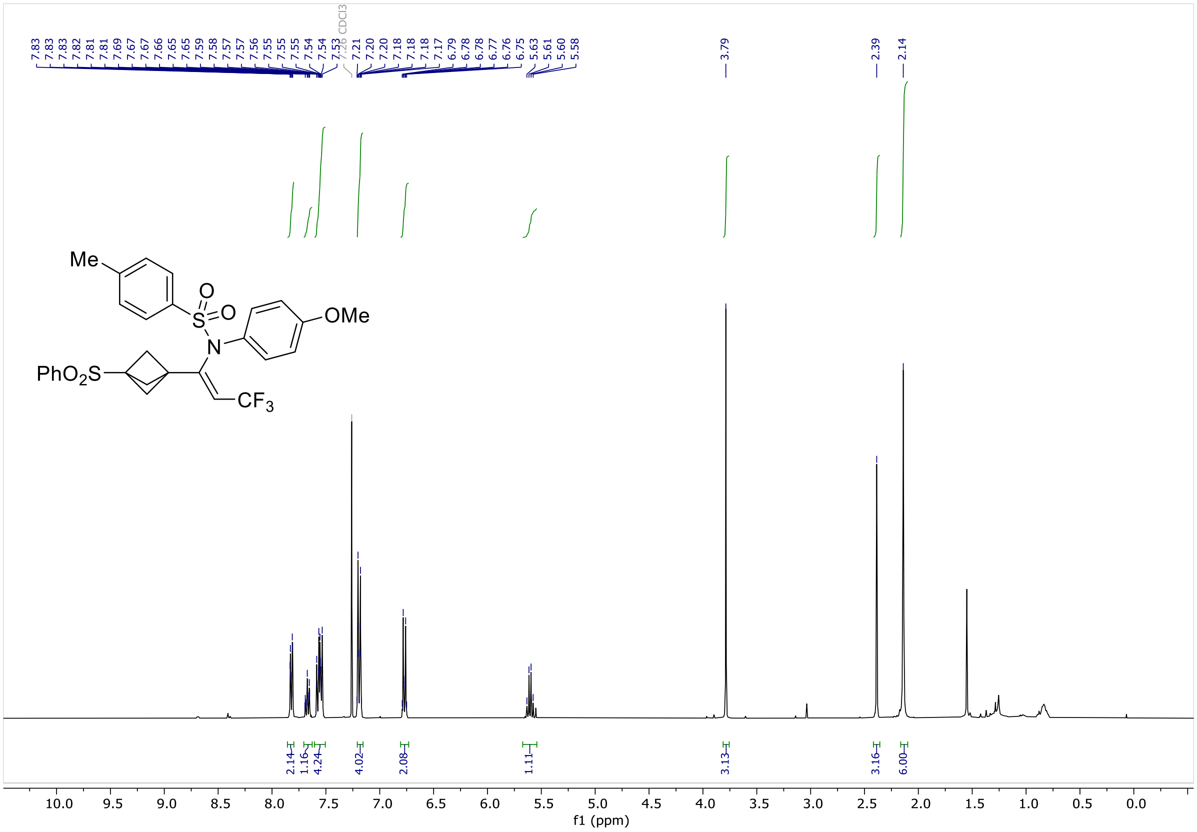

^{13}C NMR (101 MHz, $CDCl_3$) of compound **38a**

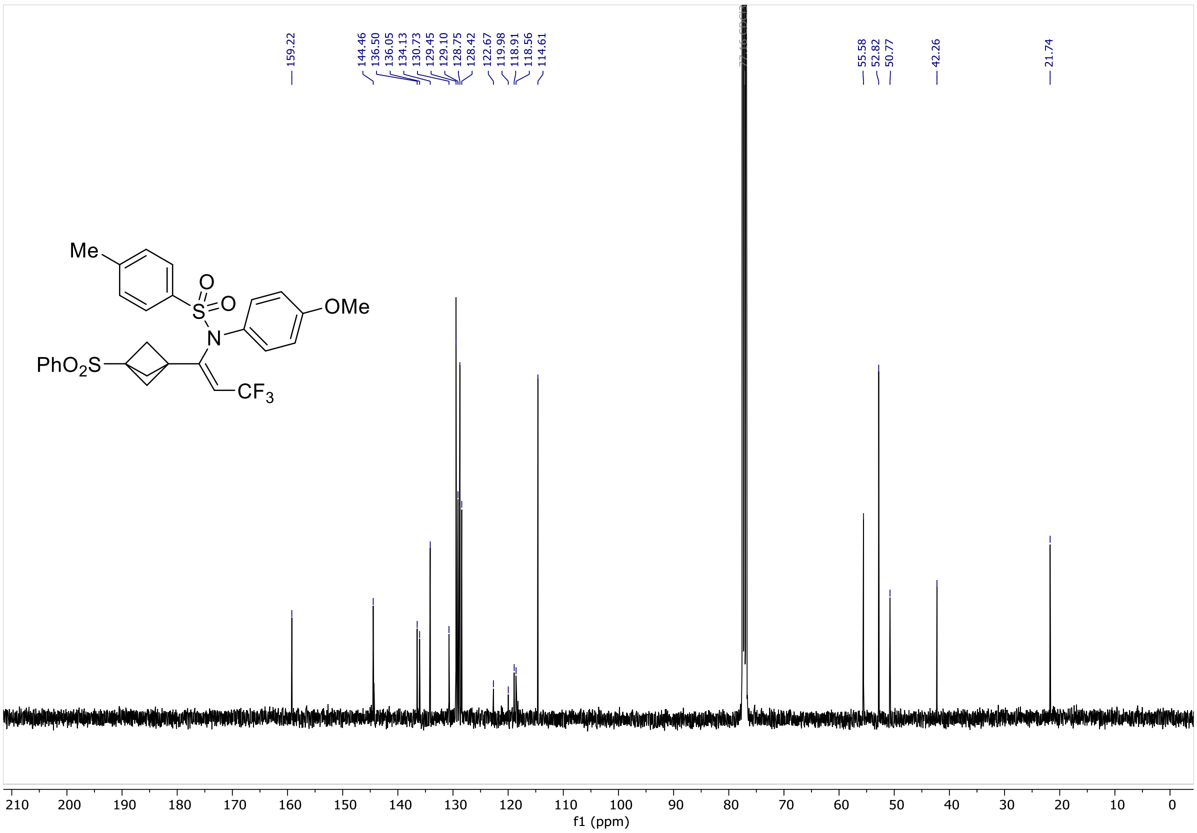

^{19}F NMR (376 MHz, $CDCl_3$) of compound **38a**

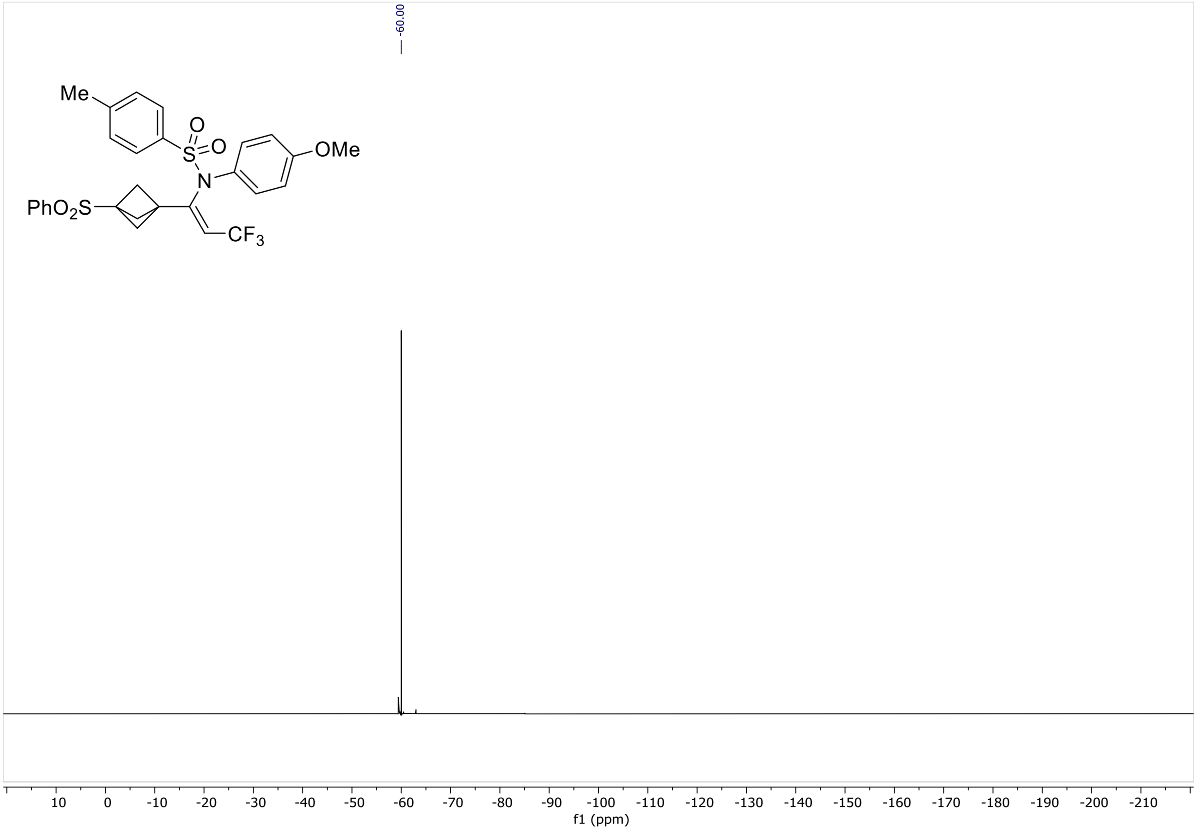

^{1}H NMR (400 MHz, $CDCl_3$) of compound **38b**

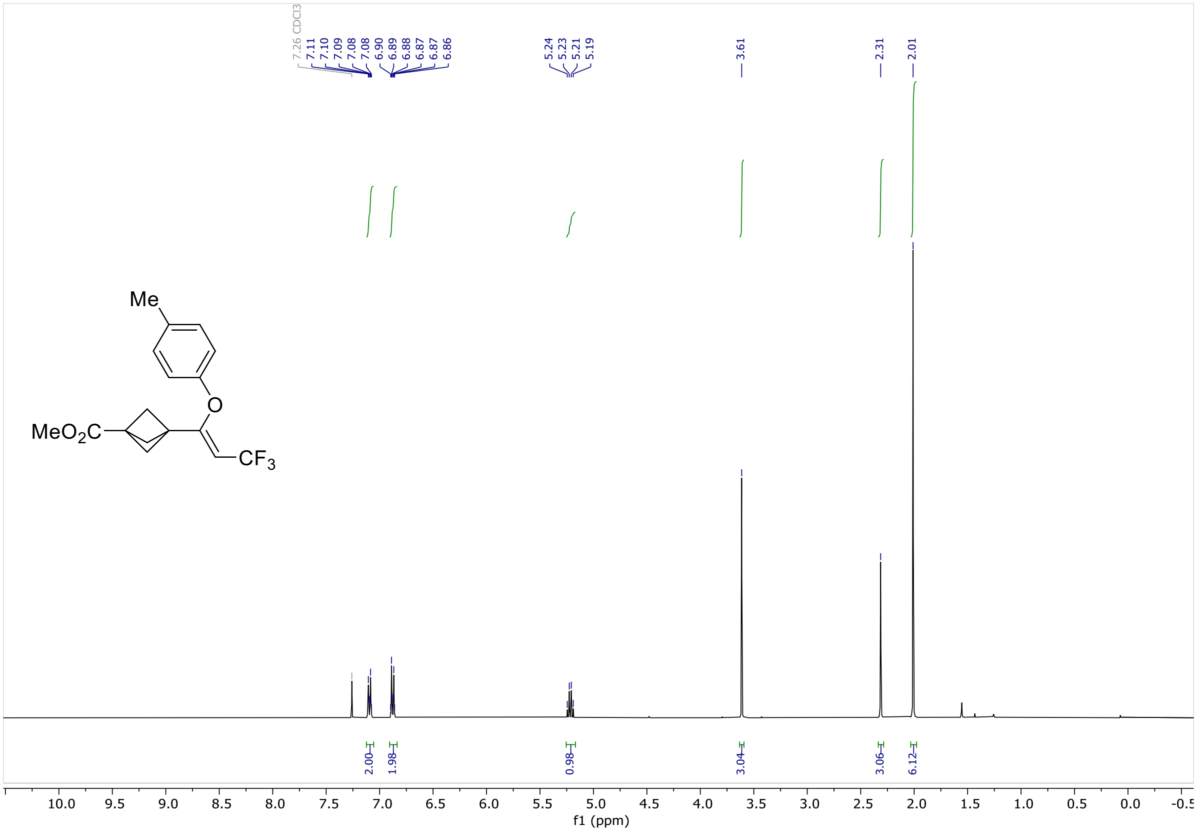

^{13}**C NMR** (101 MHz, $CDCl_3$) of compound **38b**

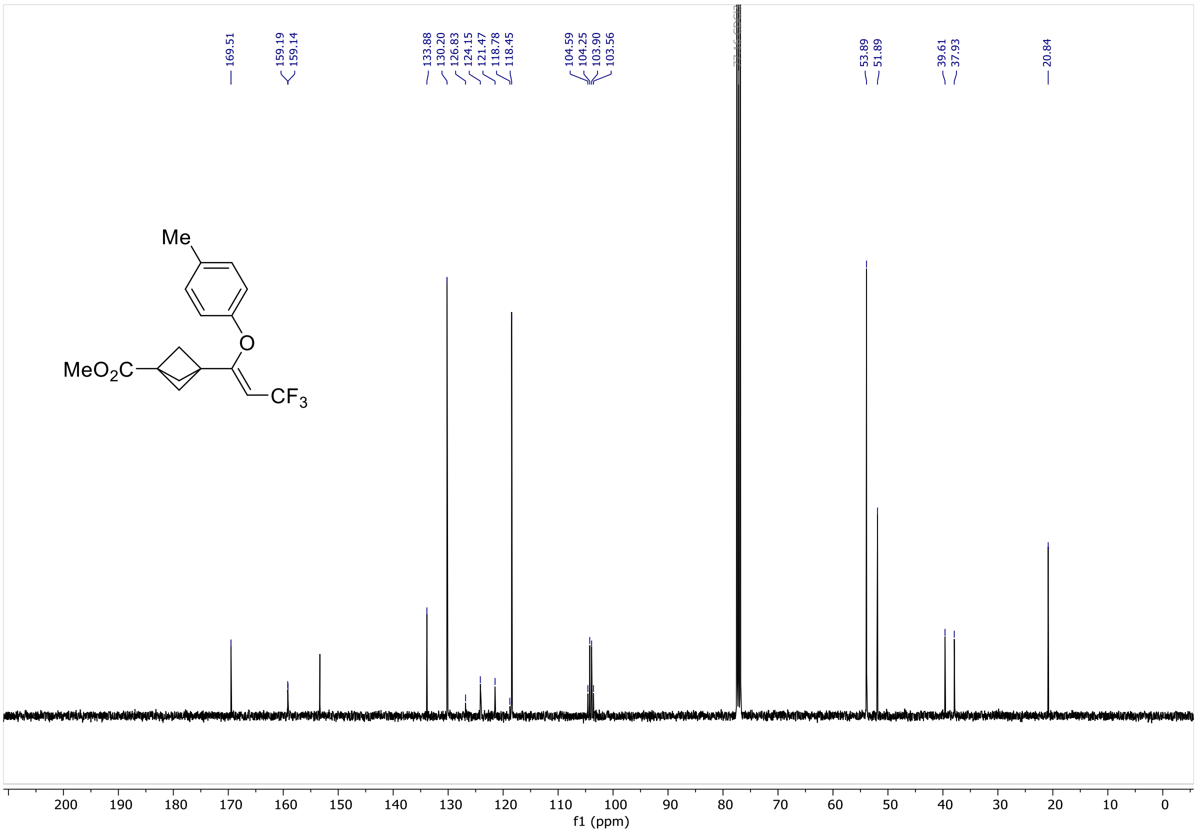

^{19}**F NMR** (376 MHz, $CDCl_3$) of compound **38b**

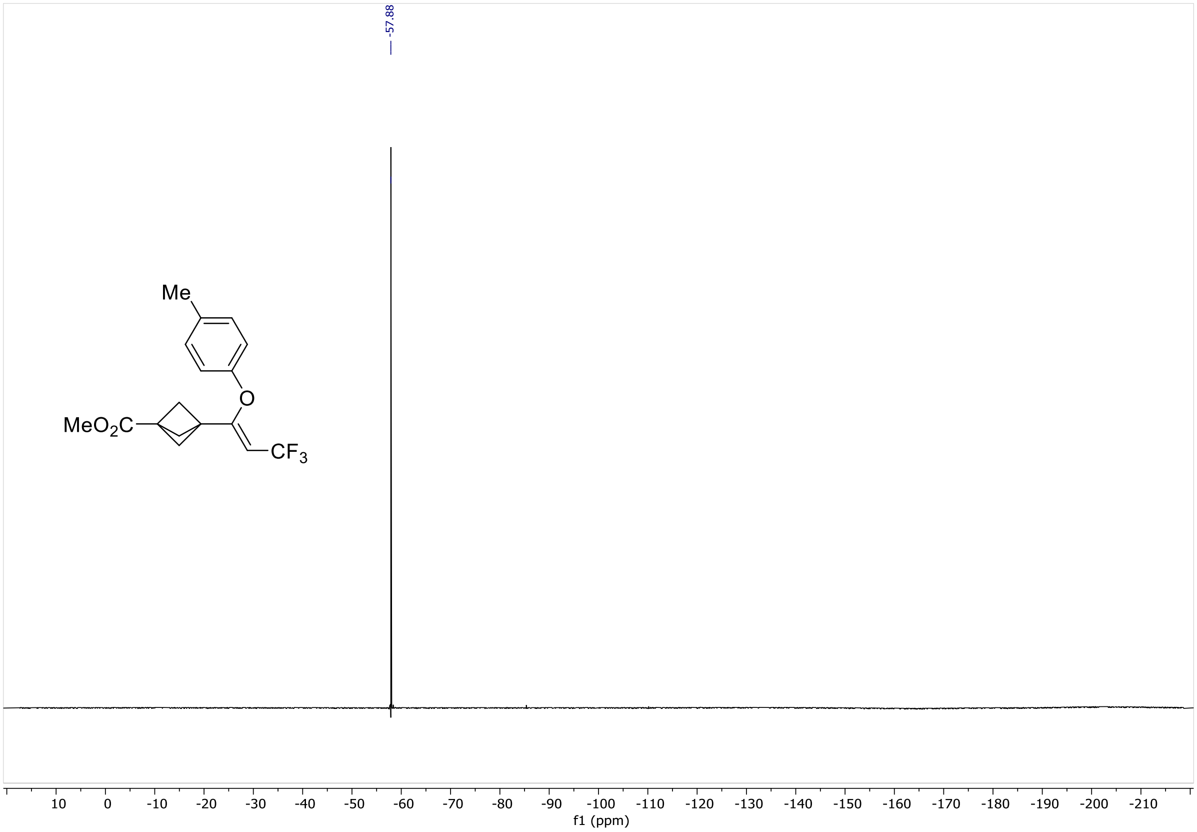

^{1}**H NMR** (400 MHz, $CDCl_3$) of compound **38c**

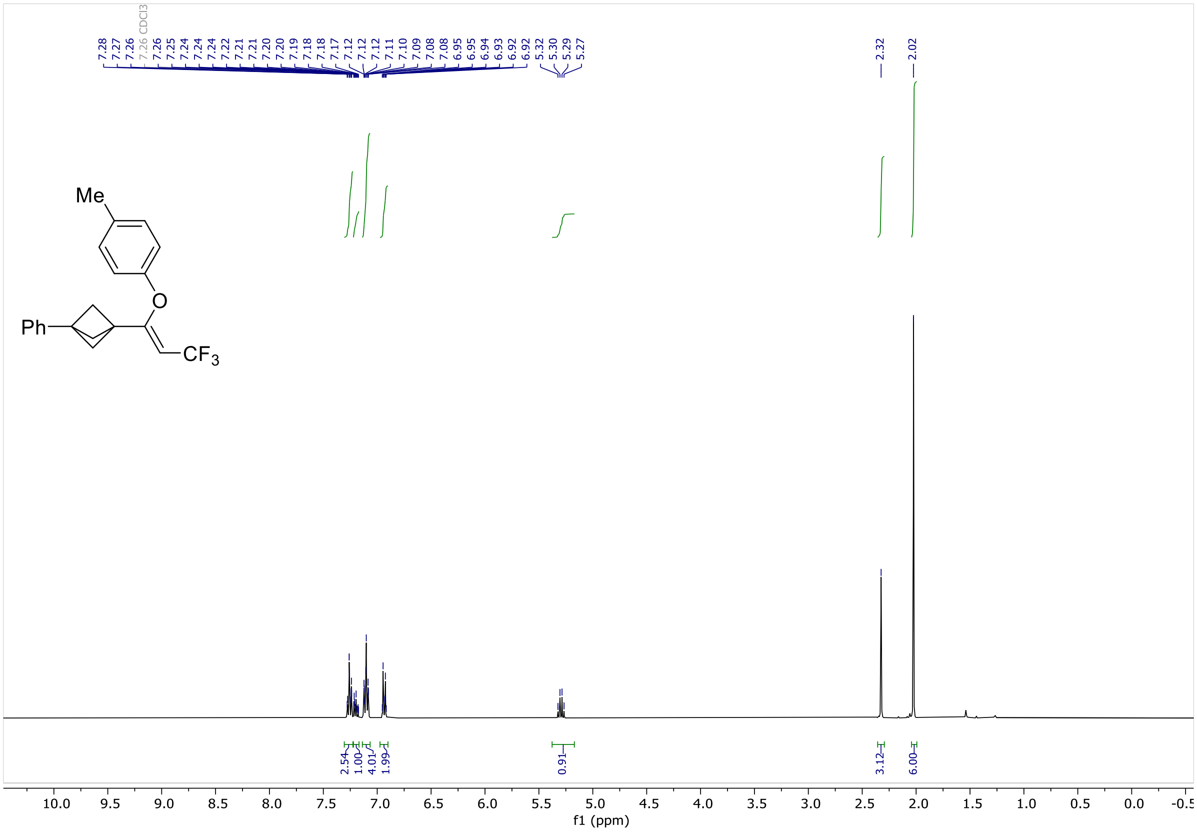

^{13}**C NMR** (101 MHz, $CDCl_3$) of compound **38c**

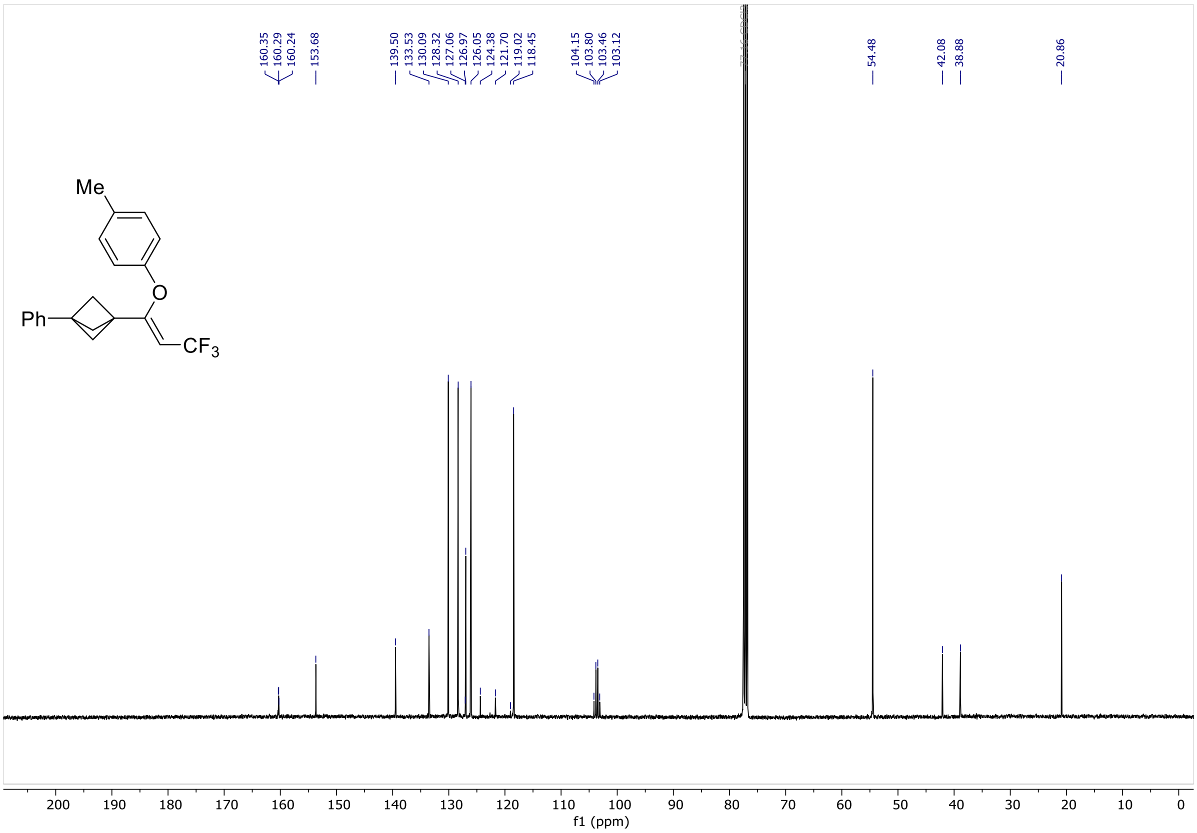

^{19}F NMR (376 MHz, $CDCl_3$) of compound **38c**

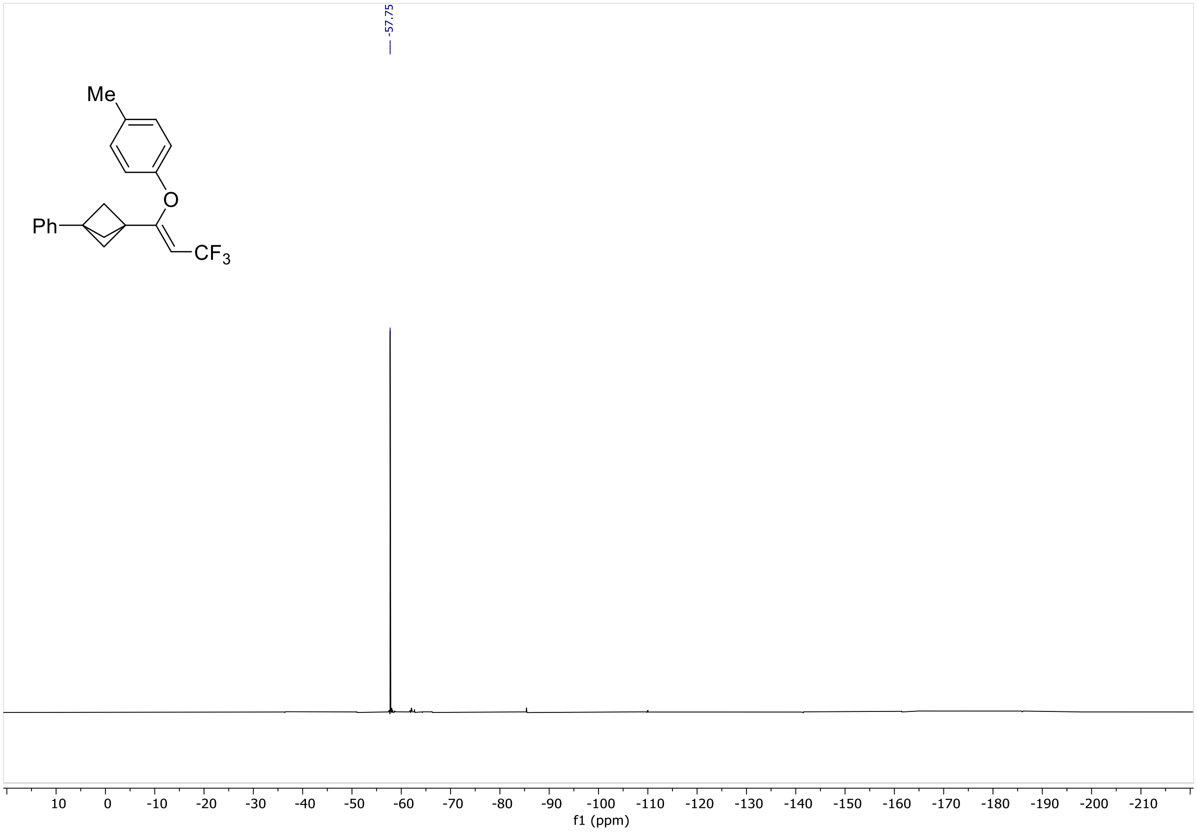

^{1}H NMR (400 MHz, $CDCl_3$) of compound **38d**

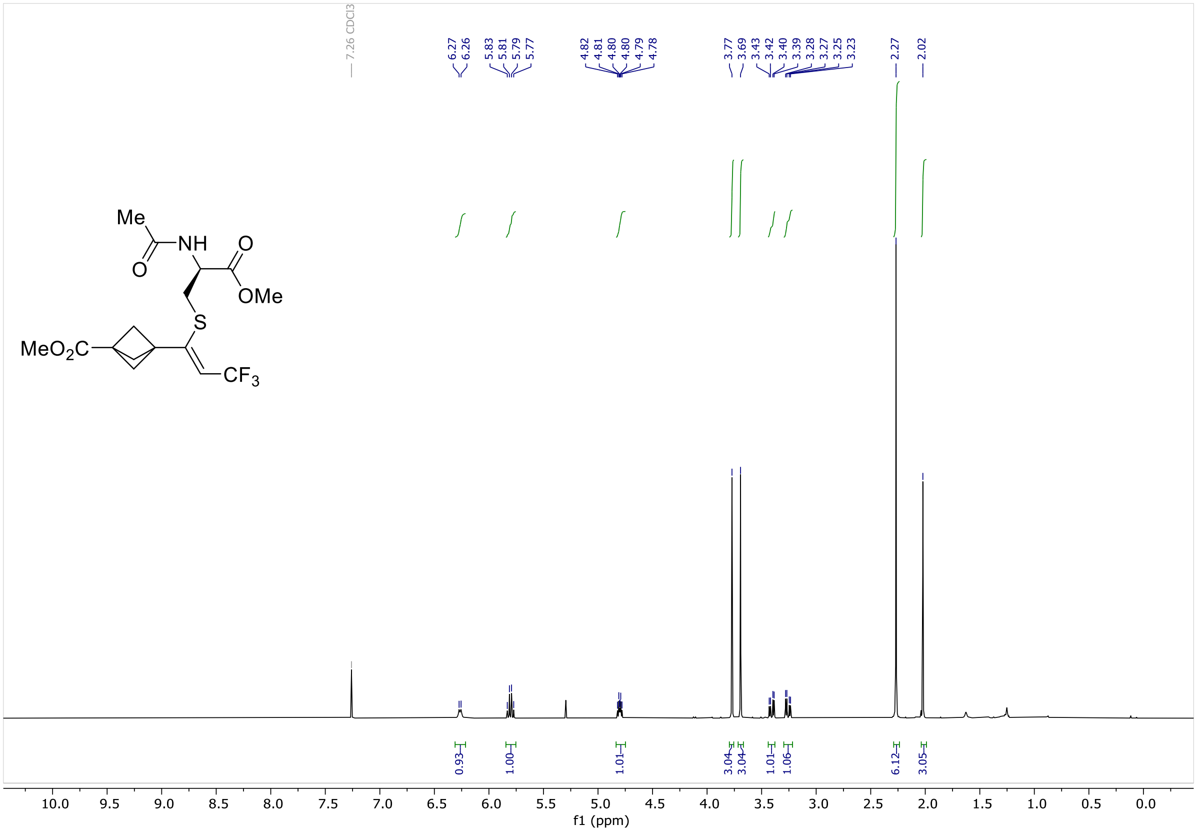

^{13}C NMR (101 MHz, $CDCl_3$) of compound **38d**

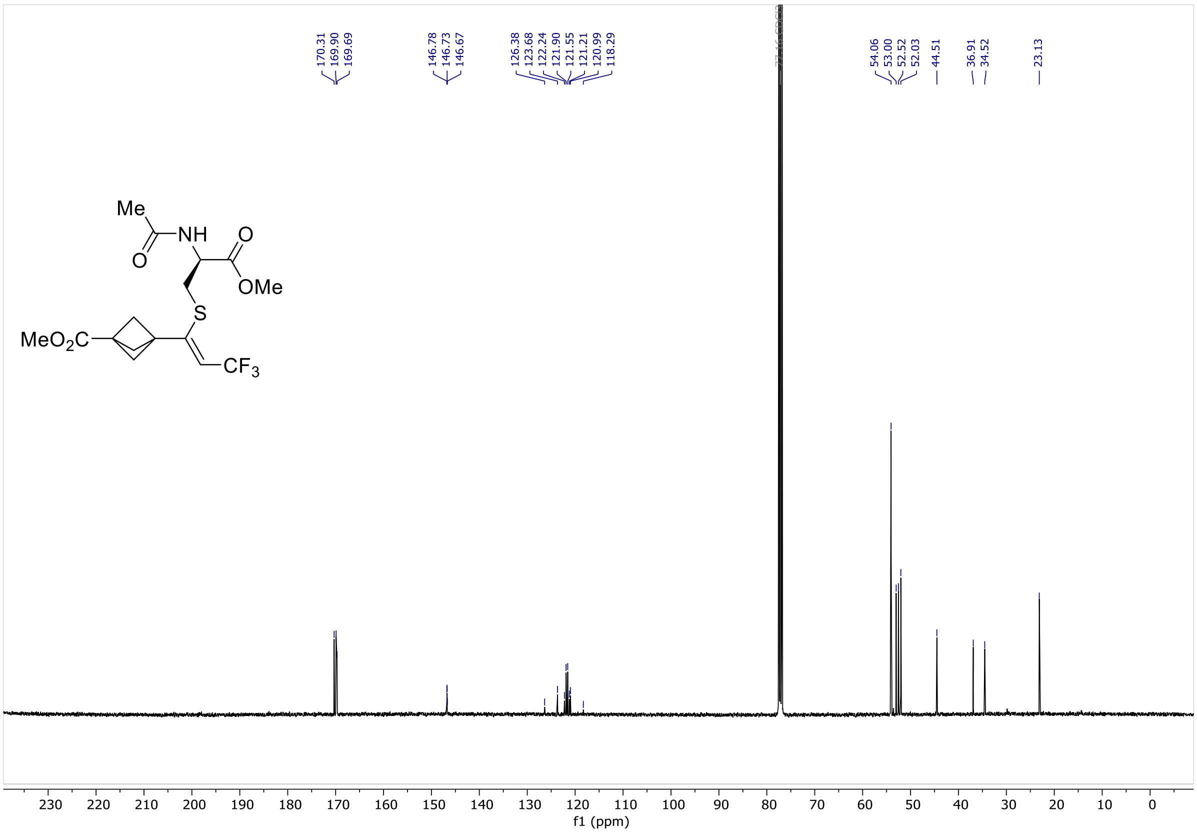

^{19}F NMR (376 MHz, $CDCl_3$) of compound **38d**

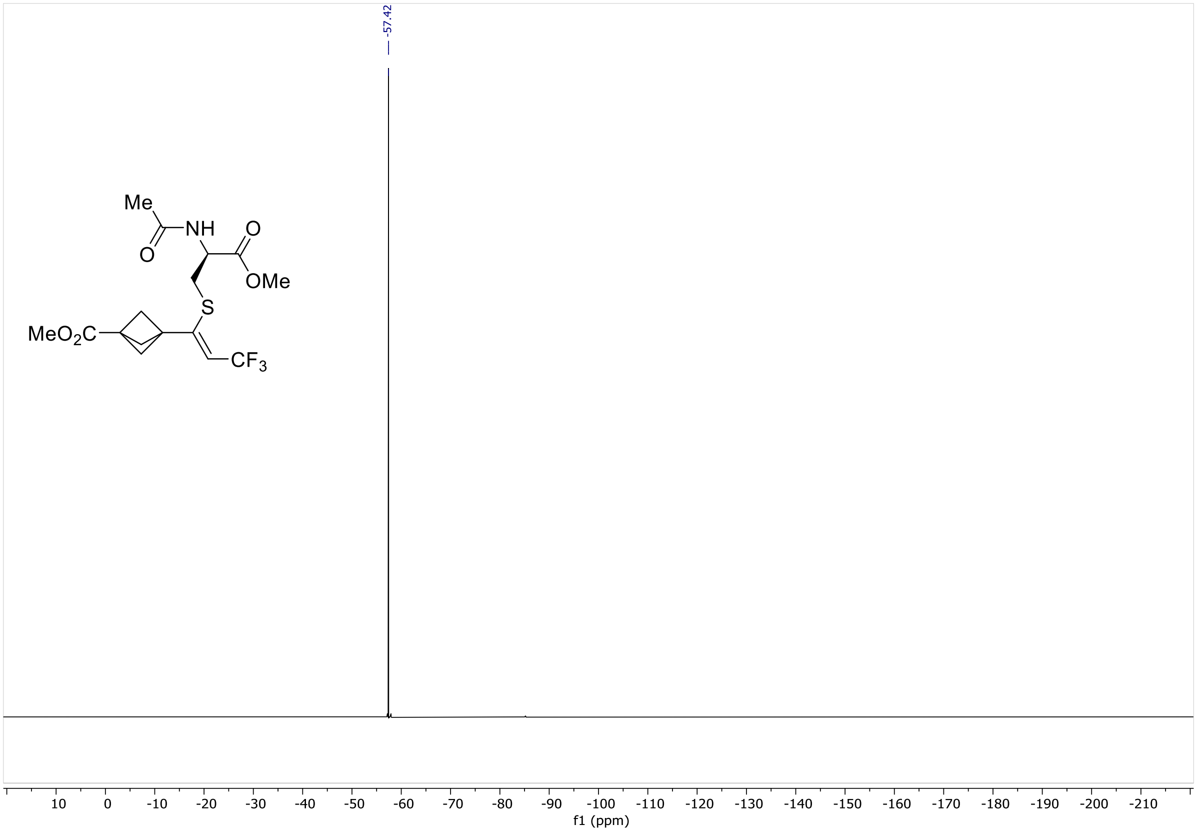

^{1}H NMR (400 MHz, $CDCl_3$) of compound **39**

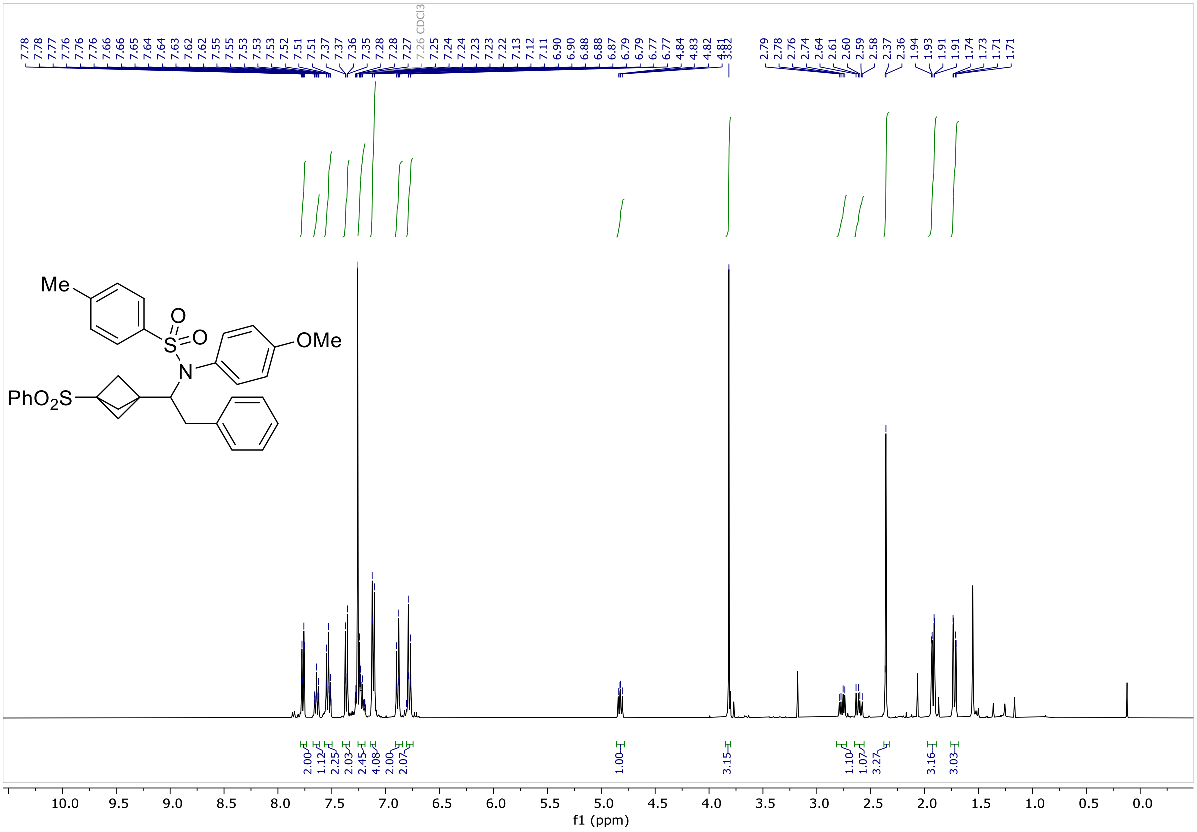

^{13}C NMR (101 MHz, $CDCl_3$) of compound **39**

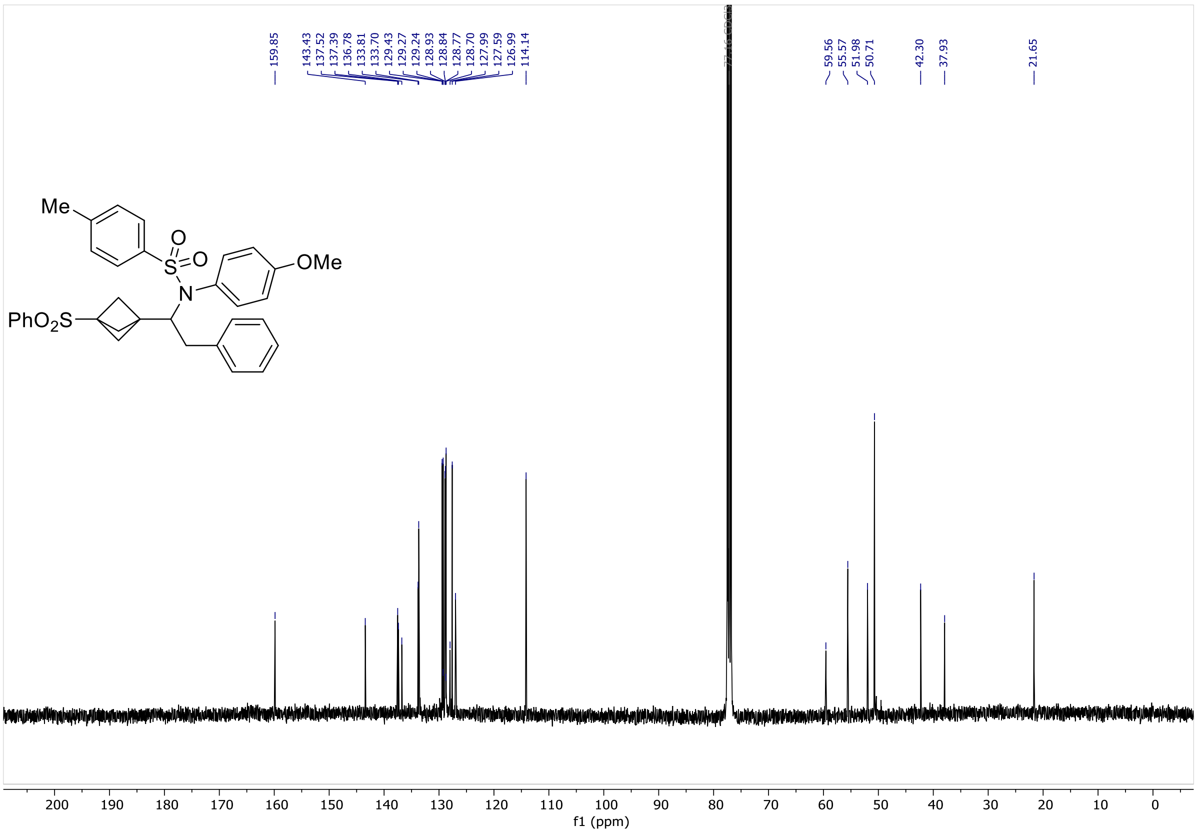

^{1}H NMR (400 MHz, $CDCl_3$) of compound **40**

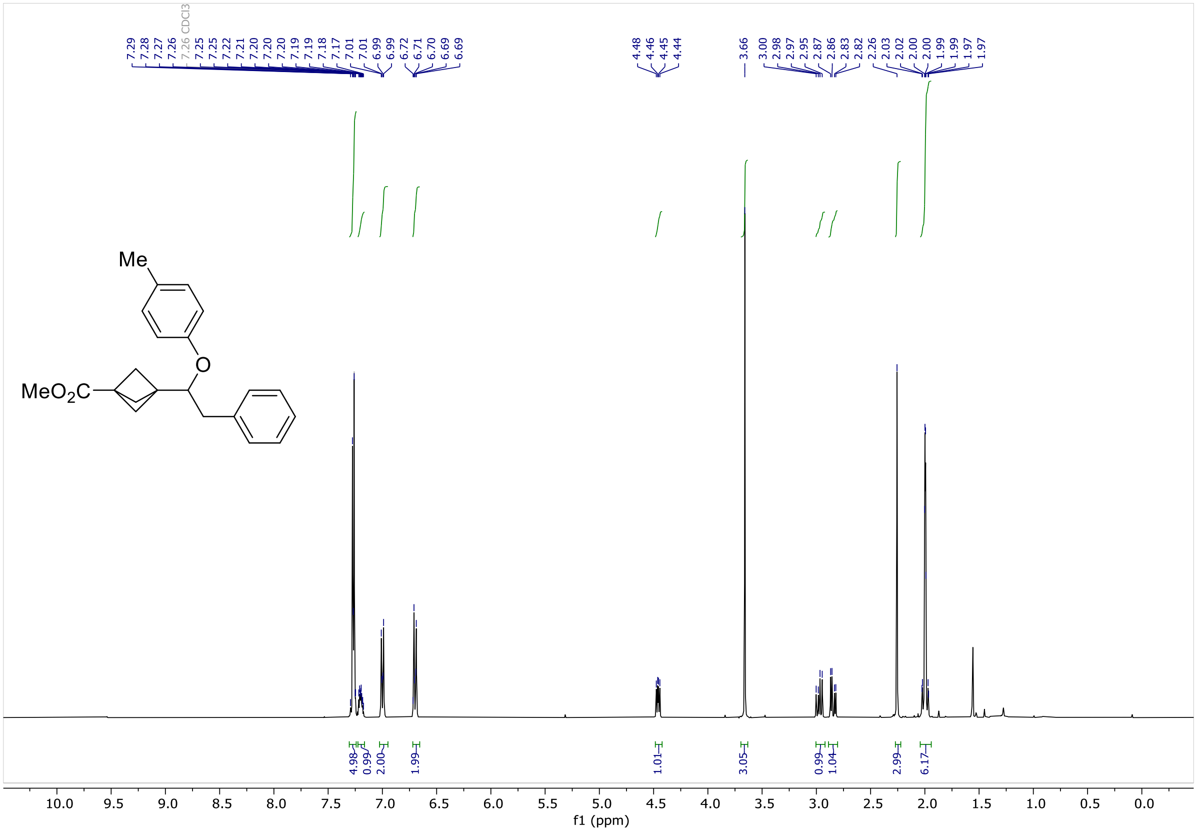

^{13}C NMR (101 MHz, $CDCl_3$) of compound **40**

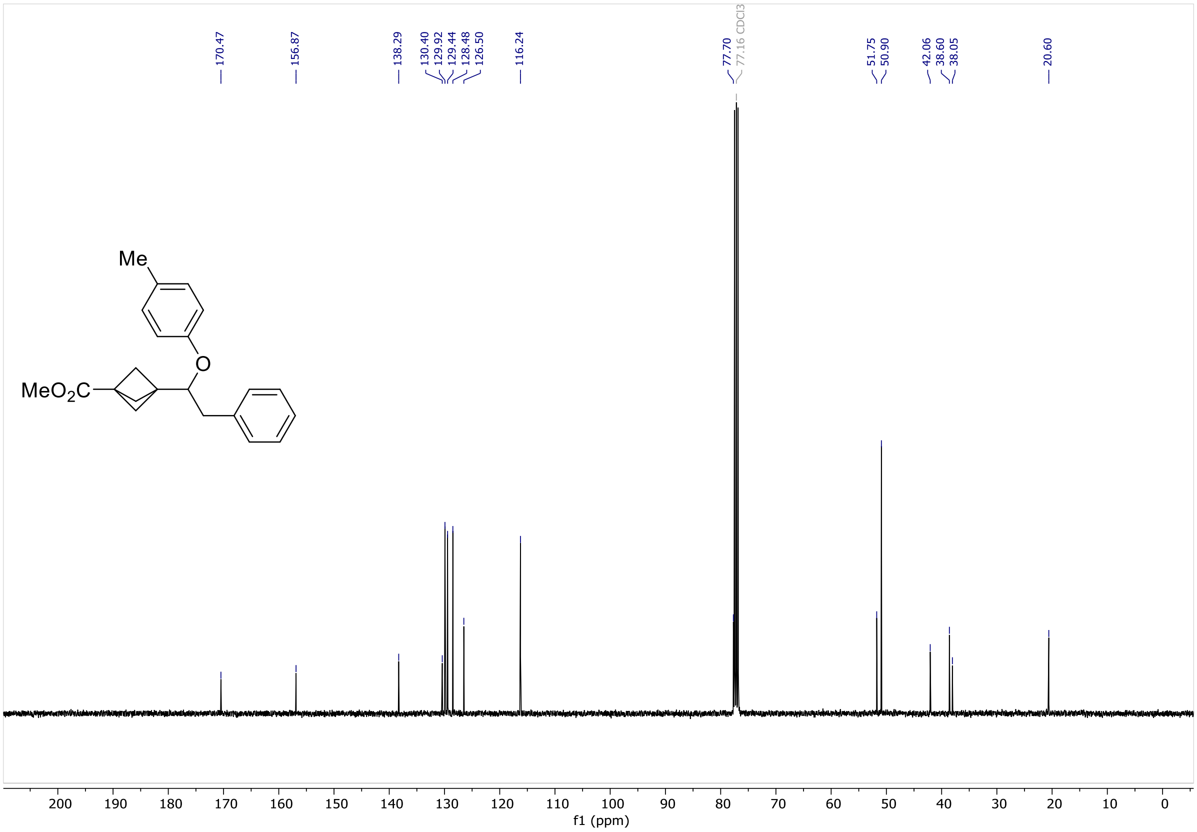

^{1}H NMR (400 MHz, $CDCl_3$) of compound **41**

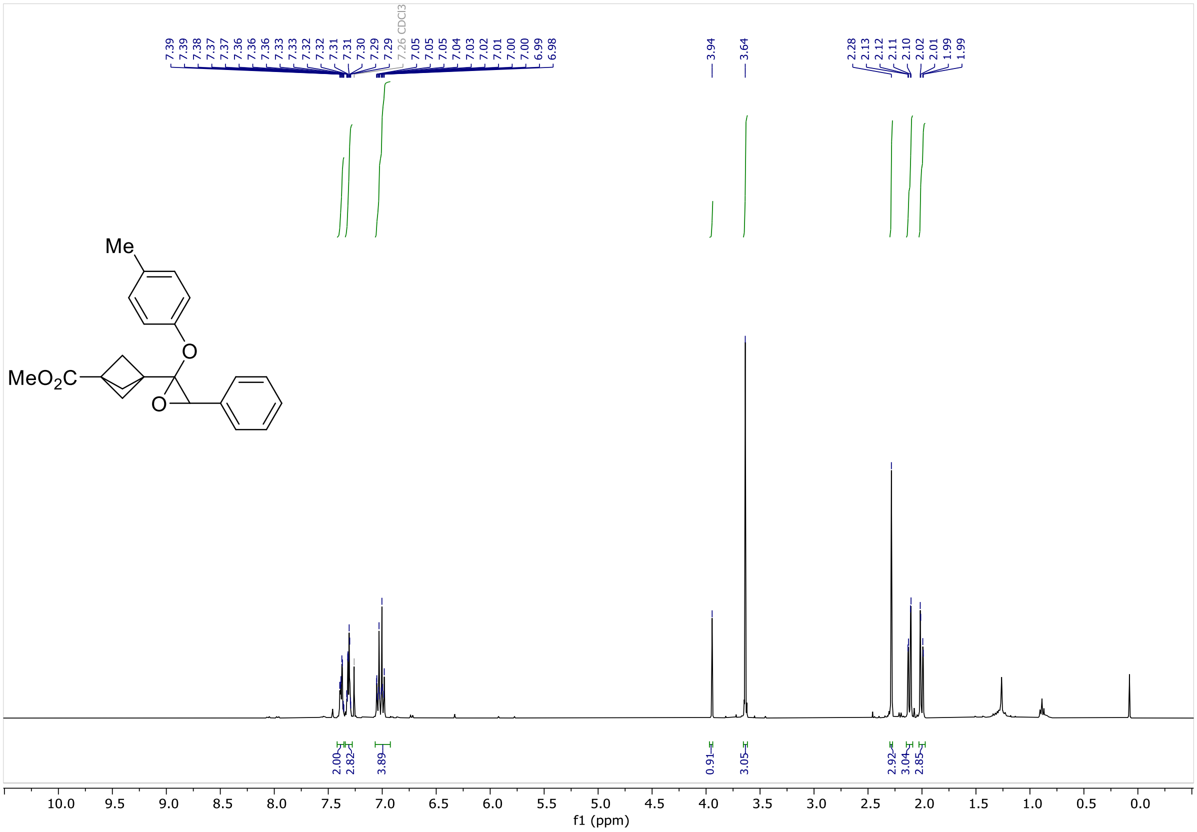

^{13}C NMR (101 MHz, $CDCl_3$) of compound **41**

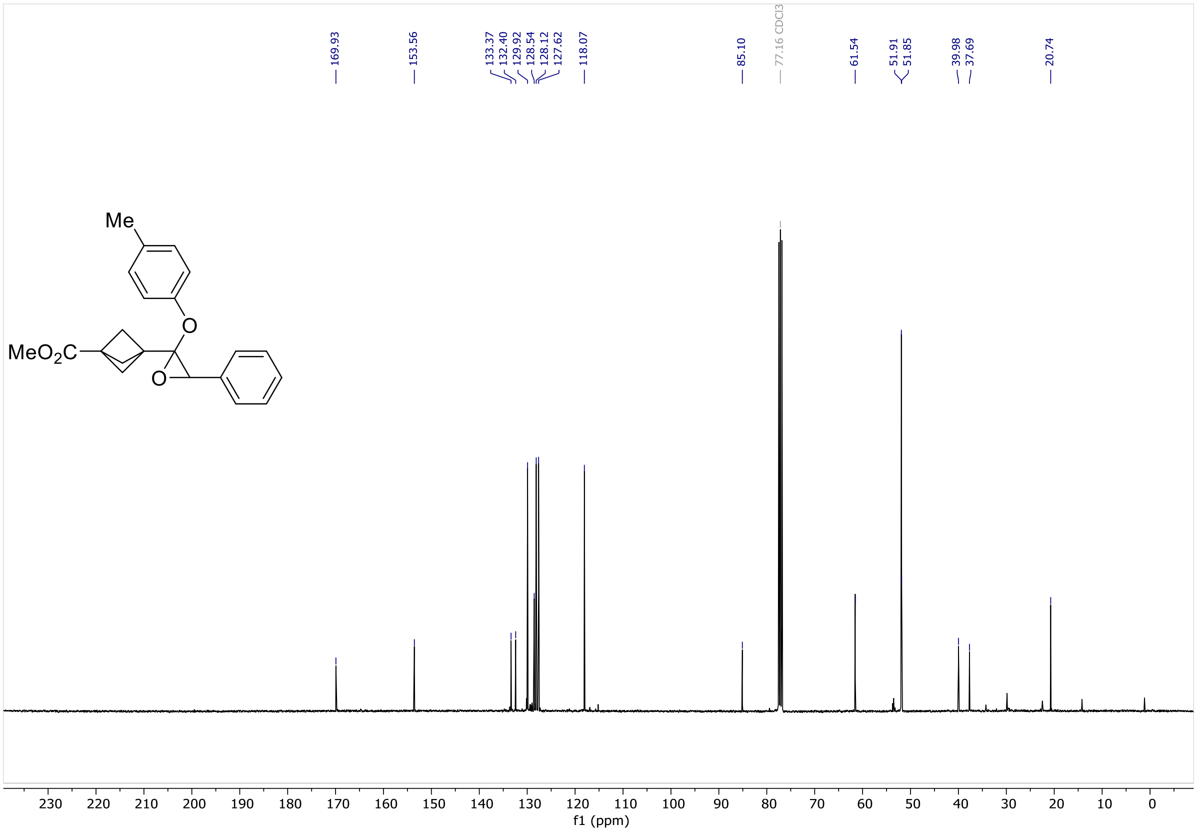

^{1}H NMR (400 MHz, $CDCl_3$) of compound **42**

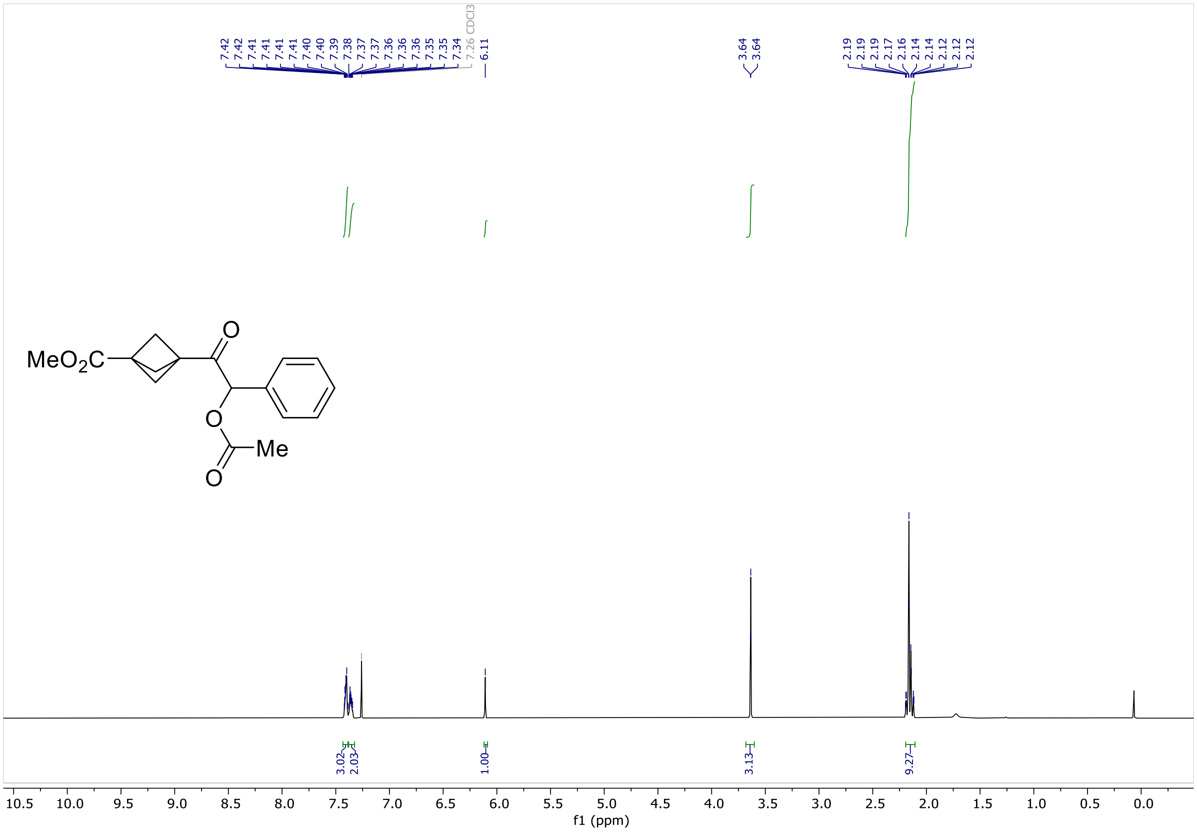

^{13}C NMR (101 MHz, $CDCl_3$) of compound **42**

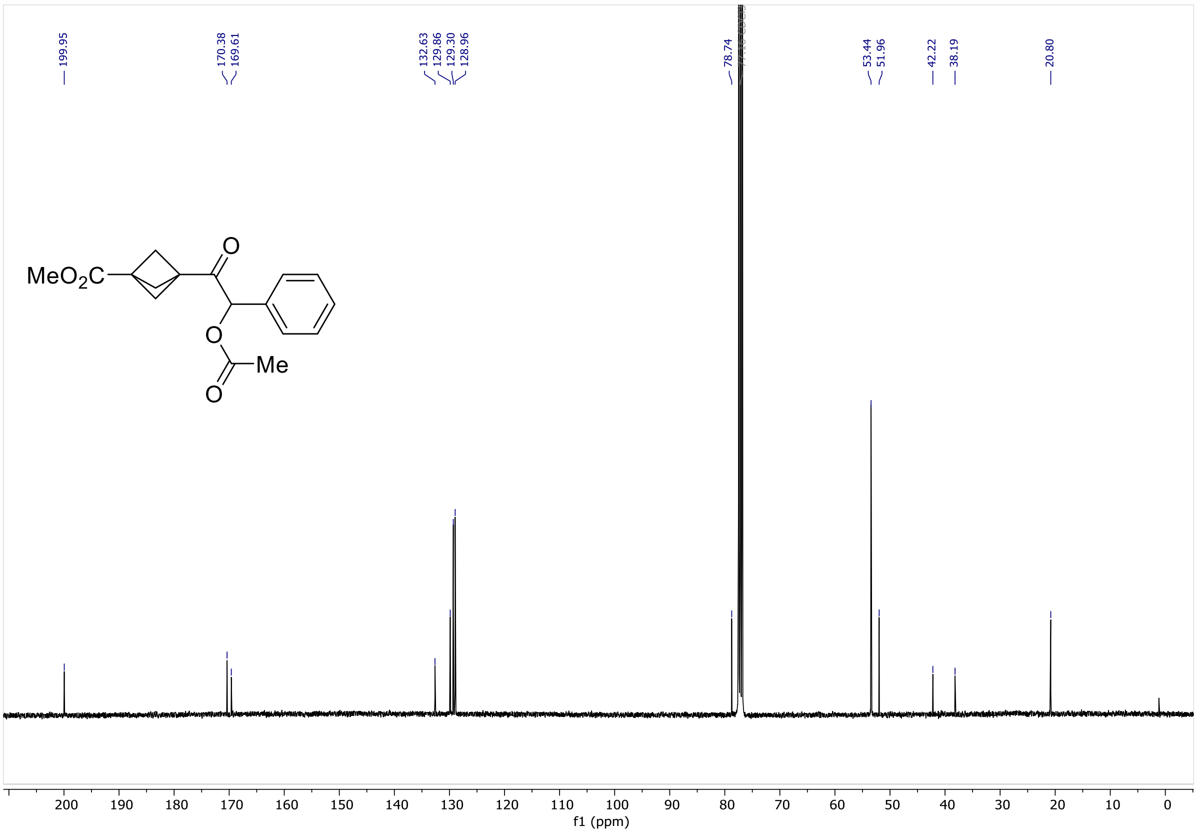

^{1}H NMR (400 MHz, $CDCl_3$) of compound **43**

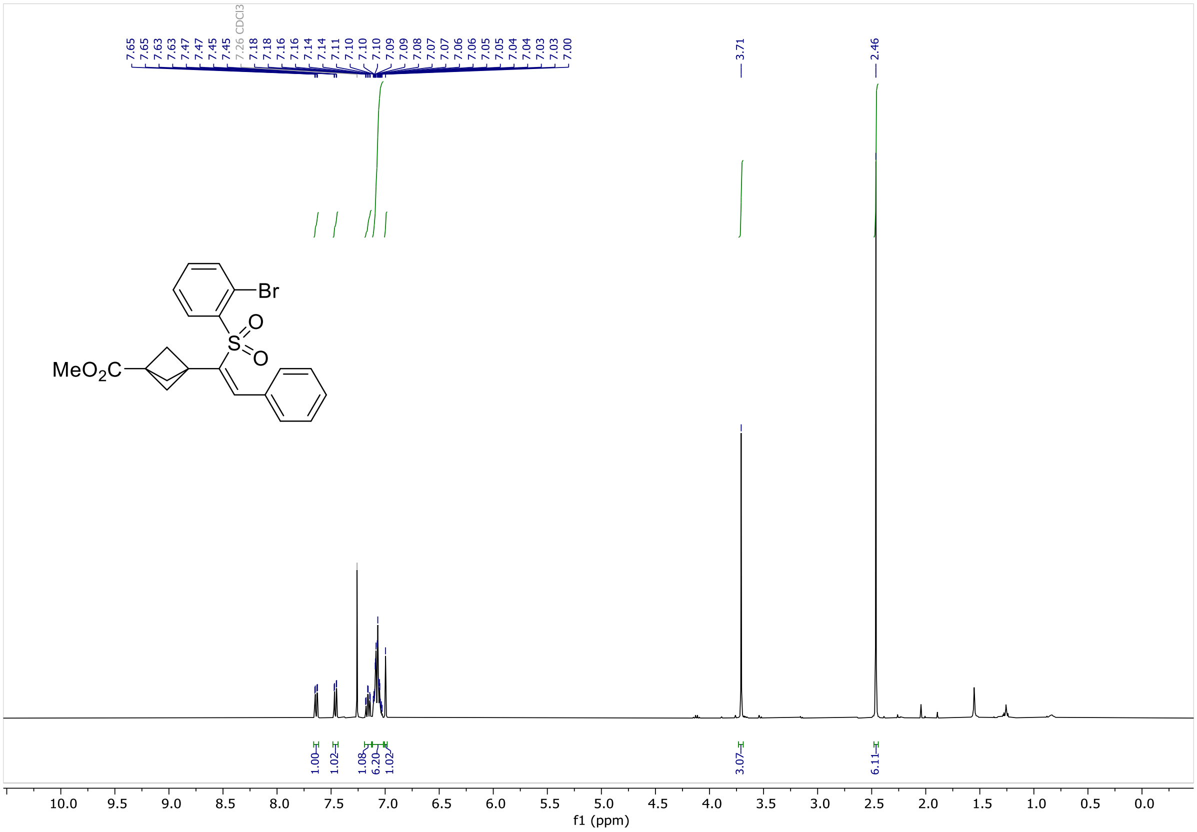

^{13}C NMR (101 MHz, $CDCl_3$) of compound **43**

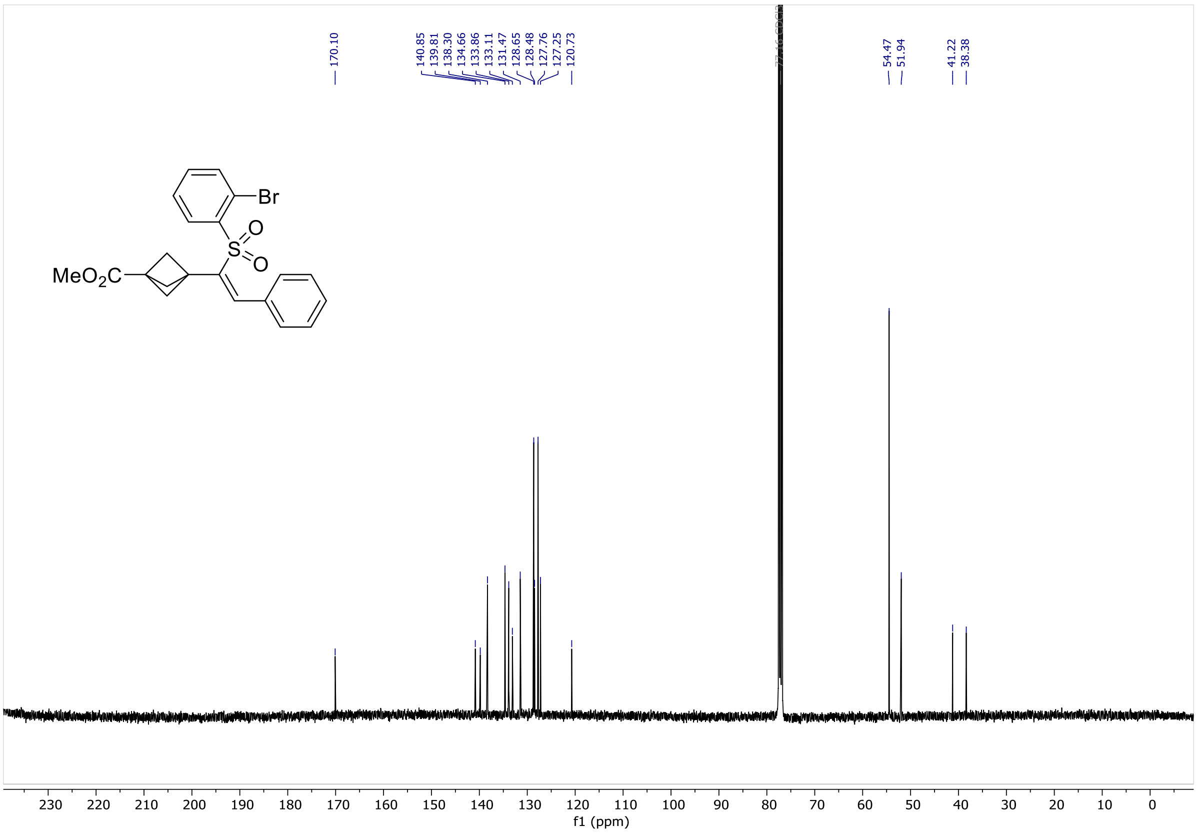

Supplement: Supplementary file 1 — Supporting Infromation [file ANIE-64-e202508404-s001.pdf]
